# Emerging trends and knowledge structure of epilepsy during pregnancy research for 2000–2018: a bibliometric analysis

**DOI:** 10.7717/peerj.7115

**Published:** 2019-06-07

**Authors:** Minglu Wang, Weitao Li, Yuying Tao, Limei Zhao

**Affiliations:** Department of Pharmacy, Shengjing Hospital of China Medical University, Shenyang, Liaoning Province, China

**Keywords:** Epilepsy, Pregnancy, Antiepileptic drugs, Scientometrics, Visualization, CiteSpace

## Abstract

**Background:**

Epilepsy during pregnancy presents a unique set of challenges for pregnant women, the fetus, and the health care community. As research in this area advances rapidly, it is critical to keep up with the emerging trends and key turning points of the development of the domain knowledge. This study aimed to construct a series of science maps to quantitatively and qualitatively evaluate the intellectual landscape and research frontiers in the field of epilepsy during pregnancy research.

**Methods:**

All publications were extracted from the Web of Science Core Collection database. Bibliometric analysis was used to analyze the scientific research outputs, including journals, countries/regions, institutions, authors (cited authors), intellectual base and research hotspots.

**Results:**

A total of 2,225 publications related to epilepsy during pregnancy were identified as published between 2000 and 2018. The overall trend of the number of publications showed a fluctuating growth from 59 articles in 2000 to 198 in 2018. *Neurology* was the leading journal in the field of epilepsy and pregnancy research both in terms of impact factor score (8.055) and *H*-index value (77). The US retained its leading position and exerted a pivotal influence in this area. The University of Melbourne was identified as a good research institution for research collaboration. Prof. Pennell and Tomson have made great achievements in this area, and Prof. Tomson laid a foundation for the development of this domain. The keyword “neonatal seizures” ranked first in research hotspots, and the keyword “autism spectrum disorders (ASD)” ranked first in research frontiers.

**Conclusions:**

Epilepsy during pregnancy is a fascinating and rapid development of subject matter. A more recent emerging trend focused on comprehensive management of pregnant and lactating women, evaluation of the safety and efficacy of newer antiepileptic drugs. The keywords “management issue,” “brain injury,” “meta-analysis,” “in utero exposure,” and “ASD” were the latest research frontiers and should be closely observed.

## Introduction

Epilepsy is one of the most common chronic neurological diseases caused by excessive discharge of brain neurons, and is characterized by recurrent, episodic, and transient central nervous system dysfunction (Fisher et al., 2005, 2014). Pregnancy is a special state that most women experience in their lifetime in which physiological changes can alter the natural course of epilepsy, affect the process of drug disposal in the body and so make therapeutic management more complicated. Women with epilepsy (WWE) are generally considered to account for 0.2–0.4% of all pregnant women (Artama et al., 2005; Viinikainen et al., 2006), whereas population-based cohort studies have shown that the incidence of epilepsy in pregnant women is as high as 0.7% (Katz et al., 2006). Considering that antiepileptic drugs (AEDs) have been widely used for indications other than epilepsy, the proportion of pregnant women and fetuses exposed to AEDs may be higher (Bobo et al., 2012). Maternal mortality for WWE is 10 times higher than for those without the disorder (Edey, Moran & Nashef, 2014). Seizures during pregnancy and in utero exposure to AEDs pose the broad array of challenges for pregnant women, the fetus and the entire healthcare community. On one hand, maternal stress and anxiety, fluctuation in hormone levels, complicated pharmacokinetic changes of AEDs during pregnancy, poor compliance, or self-replacement for medicines with poor efficacy, all of above are pregnancy-related factors that may lead to poor control of epilepsy (Harden & Lu, 2019; Kinney & Morrow, 2016; Sarma et al., 2016). It is indisputable that uncontrolled epileptic seizures could cause adverse pregnancy outcomes in clinical practice through precipitating maternal injuries, intrauterine hypoxia, and poor social conditions. On the other hand, in utero exposure to AEDs is associated with an increased risk of certain fetal congenital malformations (especially neural tube defects) and long-term cognitive and/or motor impairments (Banach et al., 2010; Tomson et al., 2018; Veroniki et al., 2017a). In view of these challenges, extensive research on basic medicine, clinical medicine, and epidemiology related to epilepsy during pregnancy has been widely carried out worldwide, and a large number of papers have been published. With the rapid development of epilepsy during pregnancy research, it is critical to keep up with emerging trends and key turning points of the development of relevant knowledge. Nevertheless, few attempts have been made to systematically analyze these publications.

Scientometrics, as a branch of informatics, has found its application in understanding emerging trends and knowledge structures in some area of research through quantitatively analyzing patterns in the scientific literature (Chen, 2017). CiteSpace is one of the most frequently used science mapping tools, originated from the concept of co-citation analysis, and created to promote the discovery of emerging trends and key turning points of the development of the domain knowledge (Chen, 2006; Cobo et al., 2011; Egghe & Rousseau, 2002). Therefore, this article aimed to identify the theme evolution and emerging trends in the intellectual landscape of research concerning epilepsy during pregnancy through scientometric analysis and visualization display based on science mapping tools.

## Materials and Methods

### Data sources and search strategies

Literatures were retrieved online through the Social Science Citation Index and the Science Citation Index-Expanded of the Web of Science Core Collection (WoSCC) on March 7, 2019. The search terms were used for the following terms (Dataset S1) with a time span ranging from January 01, 1986 to December 31, 2018: ((“convulsion” OR “epilepsy” OR “seizure”) OR (“antiepileptic” OR “anticonvulsant” OR “AED” OR “phenytoin” OR “phenobarbital” OR “divalproex” OR “valproic acid” OR “carbamazepine” OR “oxcarbazepine” OR “levetiracetam” OR “gabapentin” OR “lamotrigine” OR “topiramate”)) AND (“pregnancy” OR “pregnancies” OR “gestation” OR “pregnant” OR “maternal” OR “fetus” OR “foetus” OR “newborn”) AND Language = English, only original articles and reviews were included. All literature retrieval and record downloads were completed within the same day so as to avoid changes in citation counts caused by daily database updates.

### Data collection

Initially, the records retrieved from WoSCC were downloaded, screened, sorted and extracted by two researchers (ML Wang and WT Li) independently, any disagreements were discussed and a consensus reached. Then, these data were converted to txt format and imported into CiteSpace V 5.1.R8 SE, 64bit (Drexel University, Philadelphia, PA, USA) and the Online Analysis Platform of Literature Metrology (http://bibliometric.com/) for data analysis.

### Analysis methods

We reviewed the characteristics of publications by establishing “The WoSCC Literature Analysis Report” online, including distribution of countries/regions, institutions, journals and authors, number of annual publications, citation counts and *H*-index. The impact factor (IF) and quartile in category of journals were obtained by querying the Journal Citation Reports (JCR) 2017 standards. Among these, the number of citations received by a paper is an indicator measuring scientific value (Eyre-Walker & Stoletzki, 2013). The IF is widely regarded as a leading proxy for importance and impact of medical journals in their respective disciplines (Roldan-Valadez et al., 2018). The *H*-index is a comprehensive indicator to quantify the productivity and impact of scientific research outputs of a journal, institute, scholar, and so forth. (Kulasegarah & Fenton, 2010). According to the classification of Clarivate Analytics, the journal ranking in the JCR is based on the IF of the current year, and then divided into four equal parts (25% each) of quartiles (Q) in category as Q1–Q4 (Lahuerta et al., 2017). By combining these kinds of scientific metrics, different aspects of publications (including influence, production and reputation) can be better measured.

In this study, the online analysis platform of Literature Metrology was used to analyze the trend of annual number of publications and growth trends of countries/regions. CiteSpace was used to identify research frontiers and emerging trends in this domain by exploring collaboration networks between authors/institutes/countries, analyzing the association between journals, identifying co-cited references as well as by capturing keywords with strong citation bursts over time. We set the “time slicing” in CiteSpace based on the overall time distribution of publications, and the “years per slice” was set to 1, the “top N per slice” was set to 50, which means the single network was extracted from the top 50 cited papers in a 1-year slice (Fig. S1). Different node types were selected according to the purpose of analysis, the size of node represents the number of publications or citation counts, each node was depicted with a series of citation tree-rings across a succession of time slice. The purple ring displays the structural properties of the node, and its thickness insinuates centrality degree, which is a measure associated with the transformative potential of scientific contributions. The red ring indicated a time slice in which citation bursts or an abrupt increase in references could be detected (Chen, Ibekwe-Sanjuan & Hou, 2010; Chen, 2006).

## Results

### Distribution by time

**Figure 1:**
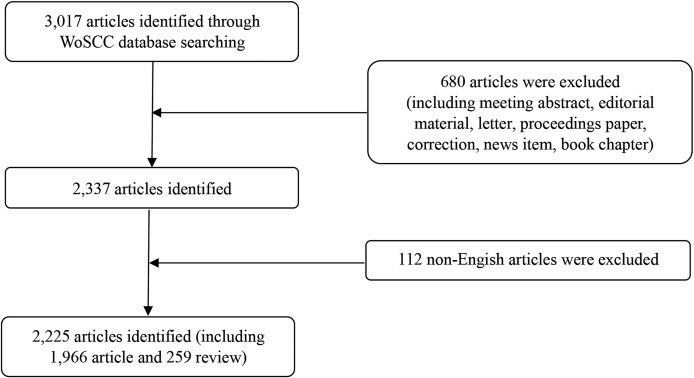
Flow chart of literature screening included in this study.

**Figure 2:**
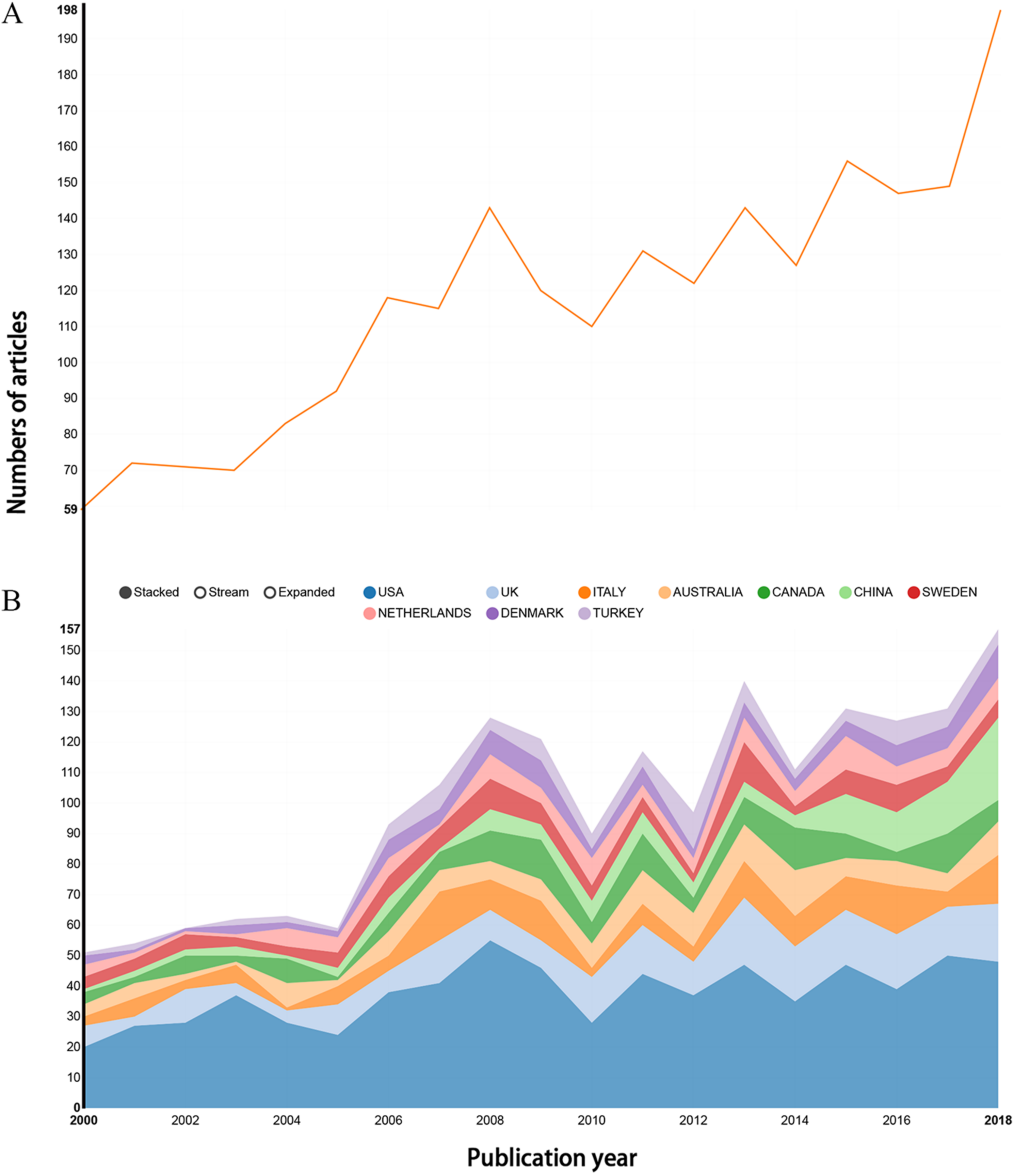
Publication outputs related to epilepsy during pregnancy research. (A) The number of annual publications and (B) growth trends of the top 10 countries/regions.

A total of 2,225 records were downloaded from the WoSCC database, including 1,966 articles and 259 reviews (Fig. 1; Fig. S2). Although we attempted to retrieve all of the literature in the field of epilepsy during pregnancy from 1986 to 2018, the results of distribution by time indicate that the literature in this field was first published in 2000 (Dataset S2). The trend of annual number of publications related to epilepsy during pregnancy from 2000 to 2018 appears in Fig. 2A, overall trends present fluctuating growth from 57 articles in 2000 to 198 in 2018.

### Distribution of countries/regions and institutions

**Figure 3:**
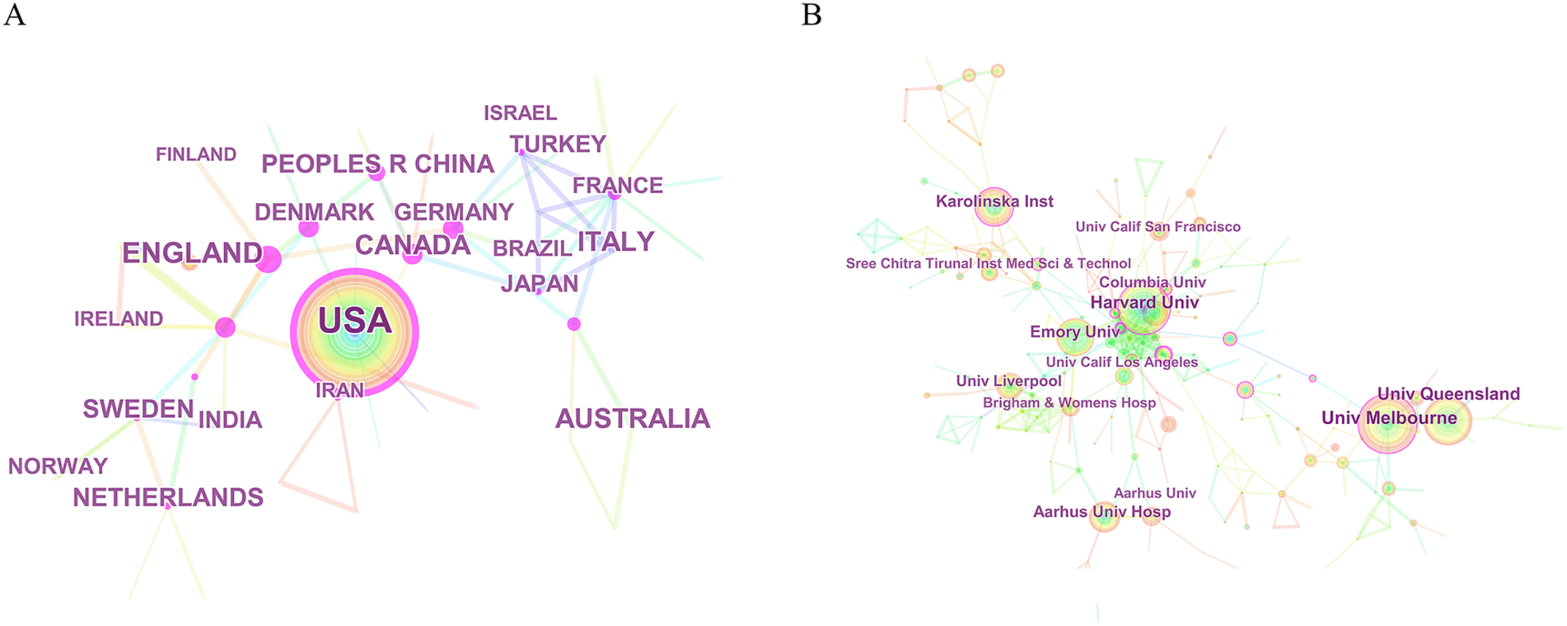
The distribution of countries/regions and institutions. (A) The network map of countries/regions that involved in epilepsy during pregnancy research and (B) the network map of institutions that involved in epilepsy during pregnancy research.

The 2,225 articles concerning epilepsy during pregnancy were contributed by active authors from 50 countries/regions. The top 10 countries/regions were occupied in the relevant research included the US, England, Italy, Australia, and Canada (Fig. 2B). The US contributed the most publications (717), followed by England (220), Italy (153), Australia (137), and Canada (135). Concerning the centrality index, although Denmark had a low scientific research output, it had a greater impact on other countries’ research (centrality = 0.54), followed by Canada (0.40) and People’s Republic of China (0.27) (Table 1). Broad and extensive cooperation had been carried out between countries/regions, which can be observed in the network map of countries/regions related to epilepsy during pregnancy research (Fig. 3A).

**Table 1:** The top 10 countries and institutions contributing to publications in epilepsy during pregnancy research.

Rank	Country/Region	Count^†^	Centrality	Institute	Count^†^	Centrality
1	US	717	0.21	University of Melbourne	68	0.15
2	England	220	0.00	Harvard University	60	0.19
3	Italy	153	0.01	University of Queensland	59	0.05
4	Australia	137	0.03	Karolinska Institution	47	0.11
5	Canada	135	0.40	Emory University	47	0.10
6	Sweden	111	0.14	Aarhus University Hospital	42	0.04
7	People’s Republic of China	96	0.27	University of Liverpool	37	0.03
8	Netherlands	96	0.15	Columbia University	34	0.06
9	Denmark	89	0.54	Sree Chitra Tirunal Institute for Medical Sciences and Technology	27	0.01
10	Turkey	86	0.00	University of California los Angeles	26	0.09

**Note:**

† The “count” refers to “the number of publications”.

The top 10 institutions engaged in relevant research sorted by number of publications included the University of Melbourne (68), Harvard University (60), the University of Queensland (59), Karolinska Institute (47), and Emory University (47) (Table 1). The network map of institutions involved in epilepsy during pregnancy research showed a low map density (Density = 0.0081) (Fig. 3B), suggesting that research groups were relatively dispersed, with scattered institutions and that mutual cooperation needed strengthening. The centrality index of most nodes was less than 0.15, indicating that most of the institutions’ influence was still at a low level and the degree of cooperation between institutions was inadequate.

### Distribution by journals

The 2,225 articles were published in 592 journals (Dataset S3), the top 15 related journals included *Epilepsia* (7.326%), *Epilepsy Behavior* (6.067%), *Seizure: European Journal of Epilepsy* (4.090%), *Neurology* (3.461%), *Epilepsy Research* (2.652%), and *Pediatric Neurology* (2.382%). Among these, *Neurology* had the largest IF of 8.055, followed by *Pediatrics* (5.515), *Epilepsia* (5.067), *Acta Neurologica Scandinavica* (3.126), and *Brain Research* (3.125). According to the JCR 2017 standards, *Neurology*, *Pediatrics, Epilepsia*, and *PlOS ONE* were classified as Q1 sorted by the IF of the JCR category to which it belongs. *Acta Neurologica Scandinavica*, *Brain Research*, *Seizure: European Journal of Epilepsy*, *Epilepsy Behavior*, and *Reproductive Toxicology* were classified as Q2. *Epilepsy Research*, *Pediatric Neurology*, and *European Journal of Paediatric Neurology* were classified as Q3 (Dataset S4). The literature published in the journal of *Neurology* that involved in epilepsy during pregnancy research had the highest *H*-index (77), followed by *Epilepsia* (47), *Epilepsy & Behavior* (24), *Seizure: European Journal of Epilepsy* (20), and *Epilepsy Research* (20) (Table 2; Dataset S5).

**Table 2:** The top 15 journals that published articles in epilepsy during pregnancy research (sorted by count).

Rank	Journal title	Count^†^ (N)	Percentage (N/2,225)	IF (2017)	Quartile in category (2017)	*H*-index^††^ (2017)
1	*Epilepsia*	163	7.326	5.067	Q1	47
2	*Epilepsy & Behavior*	135	6.067	2.600	Q2*	24
3	*Seizure: European Journal of Epilepsy*	91	4.090	2.839	Q2	20
4	*Neurology*	77	3.461	8.055	Q1	77
5	*Epilepsy Research*	59	2.652	2.491	Q3	20
6	*Pediatric Neurology*	53	2.382	2.398	Q3	18
7	*Journal of Child Neurology*	41	1.843	1.665	Q4	15
8	*PLOS ONE*	35	1.573	2.766	Q1**	12
9	*Brain & Development*	26	1.169	1.544	Q4	13
10	*Reproductive Toxicology*	26	1.169	2.580	Q2***	13
11	*Pediatrics*	24	1.079	5.515	Q1****	16
12	*Brain Research*	23	1.034	3.125	Q2*****	13
13	*Epileptic Disorders*	21	0.944	1.500	Q4	11
14	*European Journal of Paediatric Neurology*	18	0.809	2.362	Q3	9
15	*Acta Neurologica Scandinavica*	17	0.764	3.126	Q2	9

**Notes:**

† The “count” refers to “the number of publications”.

†† The *H*-index belongs to the set of documents published by the journal on the topic.

* In the column of quartile in category in the Table 1, the unspecified marked journals are of the same category of Clinical Neurology, that journals are labeled as “*,” “**,” “***,” “****,” and “*****” belong to the category of Behavioral Sciences, Multidisciplinary Sciences, Reproductive Biology, Pediatrics, and Neurosciences, respectively.

**Figure 4:**
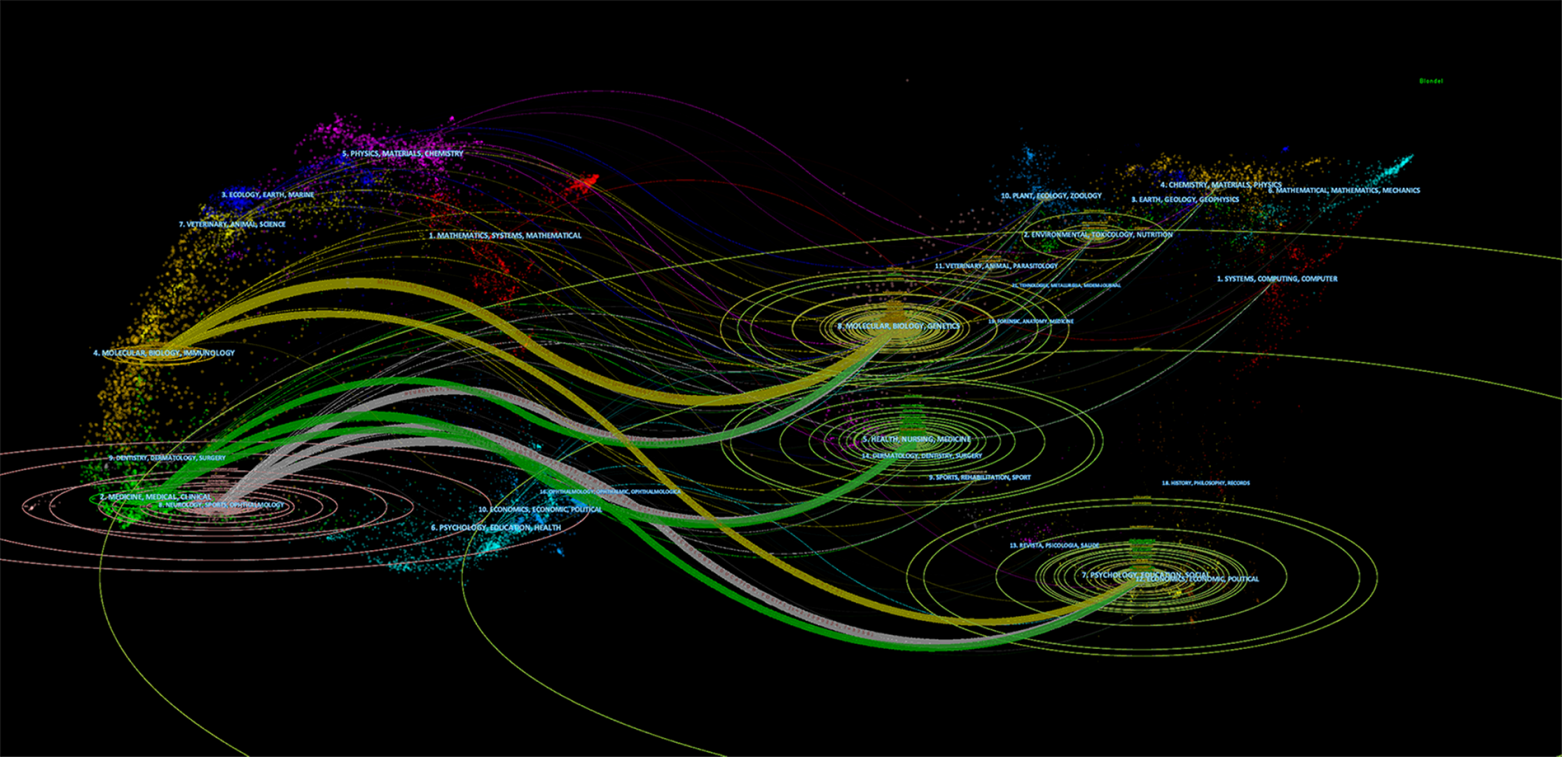
The dual-map overlay of journals related to epilepsy during pregnancy research. The graphics corresponding to the citing journals and the cited journals were located on the left and right of the network map, respectively. The blue labels represent the disciplines covered by the journal, the lines on the map start from the left and end on the right, representing the citation links. The ellipses of different size in the graph show the number of articles on their vertical axis and the number of authors on their horizontal axis.

Furthermore, we constructed a dual-map overlay of journals to clarify the cited relationship between journals (Fig. 4). It became clear that there were three main color citation paths in the double-map overlay of journals, the yellow path located at the top of pictures represents the papers published in molecular/biology/genetics journals that were partly self-cited and partly cited from journals in the psychology/education/social area, the intermediate green path represents papers published in medicine/medical/clinical journals, cited from journals in three fields: molecular/biology/genetics, health/nursing/medicine, and economics/political areas. The gray path at the bottom represents papers published in neurology/sports/ophthalmology journals, mainly cited from journals in molecular/biology/genetics, health/nursing/medicine, and economics/political areas.

### Distribution by author

**Figure 5:**
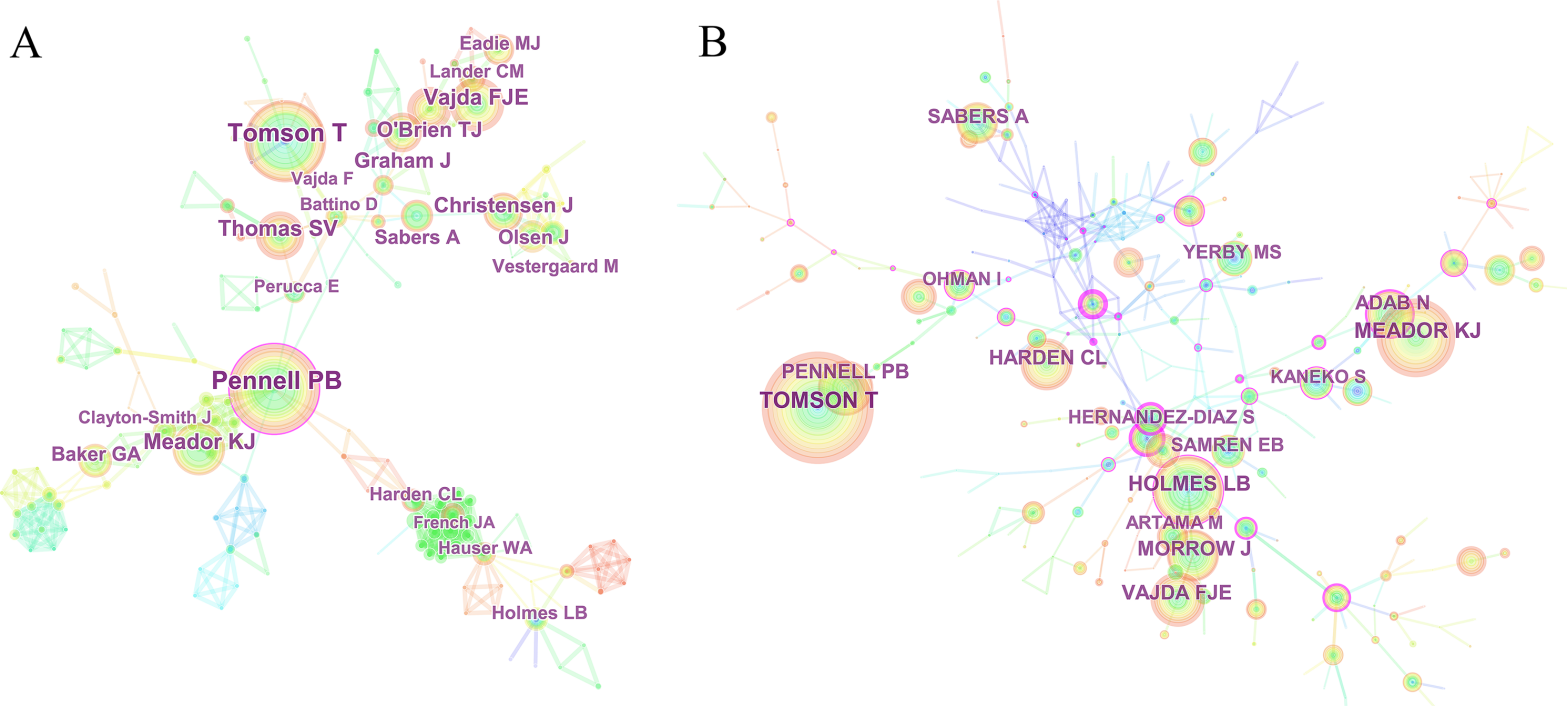
The distribution of authors contributed to epilepsy during pregnancy research. (A) The network map of productive authors and (B) The network map of co-cited authors.

The collaboration network between authors showed many sub-networks, indicating that small and medium-sized research groups were widely distributed in this area, and there was little communication and cooperation between authors (showed in Fig. 5A). Among all retrieved documents, the top 10 productive authors sorted by number of publications included Pennell PB, Tomson T, Vajda FJE, Meador KJ, Thomas SV, and O’Brien TJ (Table 3). Among them, Prof. Pennell, from the Brigham & Women’s Hospital of Harvard University in the US, ranked first with 48 articles, followed by Prof. Tomson from Sweden Karolinska University Hospital with 44 articles. These two scholars made great achievements in the research of epilepsy during pregnancy and had high authority. In addition, the centrality of these collaborations was less than 0.1 suggesting that collaboration among authors is insufficient.

**Table 3:** The top 10 authors and co-cited authors contributed to publications in epilepsy during pregnancy research.

Rank	Author	Count^†^	Centrality	Co-cited author	Citation counts	Centrality
1	Pennell PB	48	0.05	Tomson T	445	0.00
2	Tomson T	44	0.06	Meador KJ	305	0.00
3	Vajda FJE	29	0.00	Holmes LB	284	0.17
4	Meador KJ	28	0.03	Pennell PB	232	0.01
5	Thomas SV	28	0.01	Vajda FJE	219	0.01
6	O’Brien TJ	25	0.01	Harden CL	215	0.00
7	Christensen J	24	0.02	Morrow J	215	0.07
8	Graham J	23	0.01	Adab N	204	0.18
9	Boylan GB	20	0.01	Sabers A	183	0.01
10	Sabers A	19	0.02	Volpe JJ	164	0.01

**Note:**

† The “count” refers to “the number of publications”.

CiteSpace analyzed the information cited by the author and visualizes it in a co-cited authors network (Fig. 5B). Tomson T with 445 co-citations ranked first among the top 10 co-cited authors (Table 3), followed by Meador KJ (305), Holmes LB (284), and Pennell PB (232). These scholars conducted earlier research in the field of epilepsy during pregnancy, which laid a foundation for development of this domain. The centrality of most authors was less than 0.1, indicating that they did not form an influential core author group in the field of epilepsy during pregnancy research.

### Analysis of the intellectual landscape

**Figure 6:**
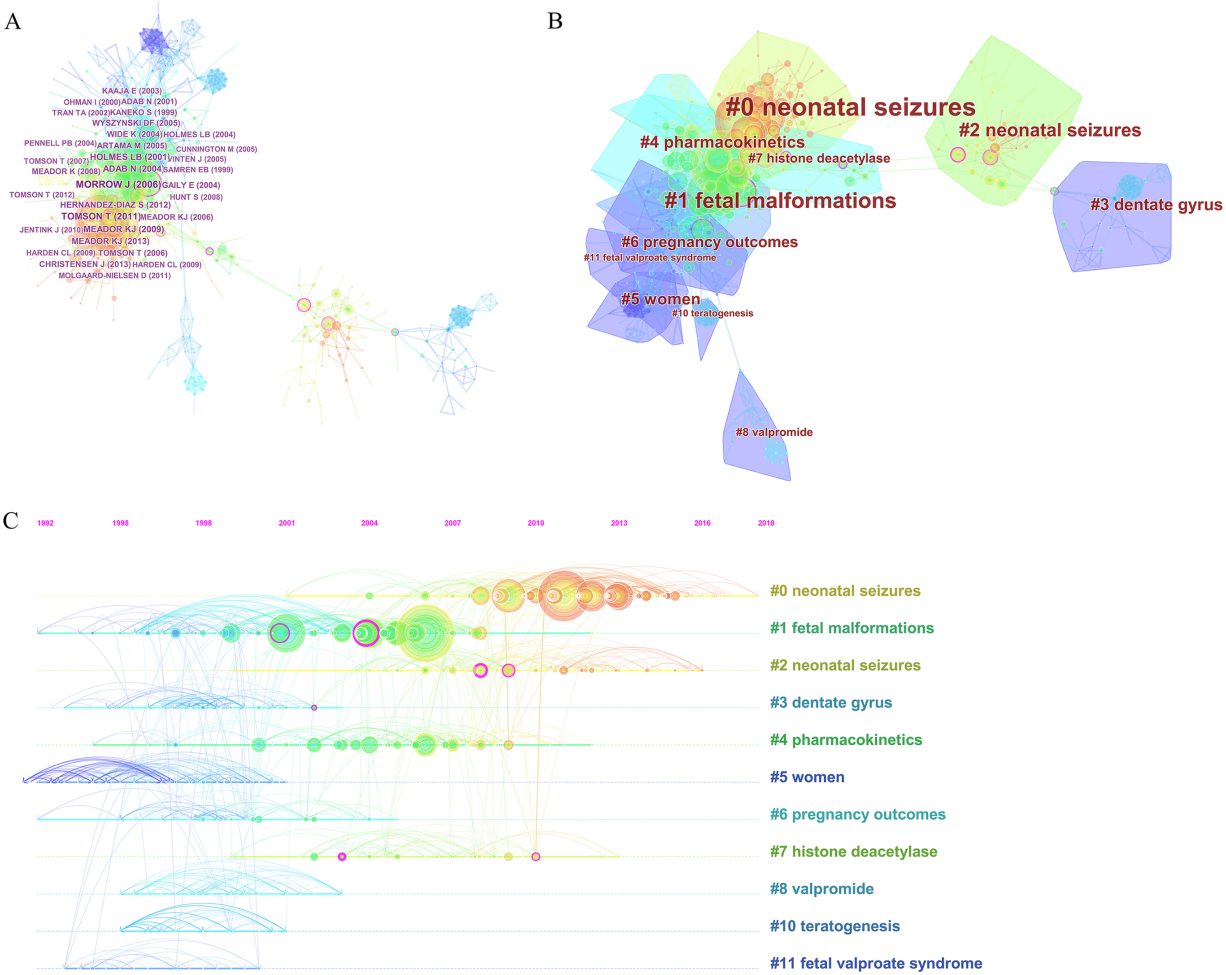
The analysis of references involved in epilepsy during pregnancy research. (A) The network map of co-citation reference, (B) the network map of co-citation clusters, and (C) the timeline view of co-citation clusters.

The network map of co-citation reference has revealed the intellectual landscape of this domain (Fig. 6A). It contained 771 references from the top 50 most-cited references per time slice for 2000–2018 (Dataset S6). Among the top 10 co-cited references sorted by the number of citations, the top ranked item was Morrow J (2006) with 162 citations in Cluster #1 (Table 4). In addition, we conducted the network map of co-citation clusters (Fig. 6B), which was one of important techniques in data mining and exploratory data analysis. The network had a high modularity of 0.8239 (showed in Fig. S3), indicating that the specialties in network map were clearly defined in terms of co-citation clusters. However, the mean silhouette value of 0.2169 was relatively low, for which the numerous small clusters possibly accounted, for the large major clusters we pay attention to in this paper is sufficiently high. Furthermore, the mean silhouette value of major clusters exceeded 0.75, indirectly indicating the reasonable and acceptable distributivity and homogeneity of clusters. Table 5 and Figure S4 demonstrates the specifics of the internal co-citation reference clusters. According to the log-likelihood ratio algorithm in CiteSpace software, Cluster #0 was labeled as “neonatal seizures” and ranked first, it contained 111 references with a mean silhouette value of 0.792, followed by the second largest cluster #1 was labeled as “fetal malformations.” Although the third largest cluster #2 was also labeled as “neonatal seizures,” cluster #2 had a different internal structure to cluster #0. A timeline visualization showed that the network was divided into distinct co-citation clusters (Fig. 6C). Tables 6 and 7 list key players of two major clusters #0 and #1 to indicate their critical research focuses.

**Table 4:** Top 10 co-cited references sorted by the number of citations.

Rank	Co-citation counts	Centrality	Cited reference	Author, year, journal, volume, and pages	Cluster #
1	162	0.02	Malformation risks of antiepileptic drugs in pregnancy: a prospective study from the UK Epilepsy and Pregnancy Register	Morrow J, 2006, J Neurol Neurosur Ps, V77, P193	1
2	137	0.01	Dose-dependent risk of malformations with antiepileptic drugs: an analysis of data from the EURAP epilepsy and pregnancy registry	Tomson T, 2011, Lancet Neurol, V10, P609	0
3	106	0.02	The teratogenicity of anticonvulsant drugs	Holmes LB, 2001, New Engl J Med, V344, P1132	1
4	97	0.03	Cognitive function at 3 years of age after fetal exposure to antiepileptic drugs	Meador KJ, 2009, New Engl J Med, V360, P1597	0
5	92	0.05	The longer-term outcome of children born to mothers with epilepsy	Adab N, 2004, J Neurol Neurosur Ps, V75, P1575	1
6	86	0.02	Comparative safety of antiepileptic drugs during pregnancy	Hernandez-diaz S, 2012, Neurology, V78, P1692	0
7	80	0.00	Fetal antiepileptic drug exposure and cognitive outcomes at age 6 years (NEAD study): a prospective observational study	Meador KJ, 2013, Lancet Neurol, V12, P244	0
8	76	0.02	Antiepileptic drug use of women with epilepsy and congenital malformations in offspring	Artama M, 2005, Neurology, V64, P1874	1
9	74	0.03	Major malformations in infants exposed to antiepileptic drugs in utero, with emphasis on carbamazepine and valproic acid: a nation-wide, population-based register study	Wide K, 2004, Acta Paediatr, V93, P174	1
10	73	0.02	Increased rate of major malformations in offspring exposed to valproate during pregnancy	Wyszynski DF, 2005, Neurology, V64, P961	1

**Table 5:** Top 11 largest clusters of co-cited references (size sorting).

Cluster ID	Size	Silhouette	Mean (year)*	Label (LLR)^†^^▴^	Label (TF*IDF)^††^	Label (MI)^†††^
#0	111	0.792	2011	Neonatal seizures	Antiepileptic drugs	Anorexia
#1	83	0.770	2001	Fetal malformations	Psychomotor development	Teratogenic
#2	60	0.974	2010	Neonatal seizures	Neonatal seizures	Epilepsy monitoring
#3	49	0.994	1998	Dentate gyrus	Preterm infants	Androgen
#4	48	0.873	2005	Pharmacokinetics	Malformations	Immunoassay
#5	46	0.936	1996	Women	Teratogenesis	Cohort studies
#6	42	0.778	1999	Pregnancy outcomes	Educational need	Substitution study
#7	29	0.948	2006	Histone deacetylase	Valproic acid	Glutamate
#8	23	0.997	1998	Valpromide	Teratogenicity	Epilepsy monitoring
#10	16	0.994	1997	Teratogenesis	Questionnaire	Epilepsy monitoring
#11	16	0.955	1996	Fetal valproate syndrome	Neuro development	Epilepsy

**Notes:**

* Mean (year) represents the average publication time of the literature contained in this cluster.

† LLR (Log-likelihood ratio) is one of the clustering label word extraction algorithm.

†† TF-IDF (Term frequency–inverse document frequency) is a commonly used weighting techniques for information retrieval and data mining, this algorithm can generate cluster labels based on the title of the citing document.

††† MI (Mutual information) is also one of the clustering label word extraction algorithm.

▴ Clusters are referred in terms of the labels selected by log-likelihood ratio test method (LLR) in this study.

**Table 6:** Cited and citing references of Cluster #0 neonatal seizures.

Cluster #0 neonatal seizures			
Cited references		Citing articles	
Cites	Author (year) journal, volume, page	Coverage (%)	Author (year) title
134	Tomson T (2011) Lancet Neurol, V10, P609	18	Eadie, MJ (2014) **Treating epilepsy** in pregnant women
97	Meador KJ (2009) New Engl J Med, V360, P1597	15	Tomson, T (2012) Teratogenic effects of antiepileptic drugs
83	Hernandez-diaz S (2012) Neurology, V78, P1692	10	Borthen, I (2012) Pregnancy complications in patients with epilepsy
78	Meador KJ (2013) Lancet Neurol, V12, P244	9	Hill, DS (2010) Teratogenic effects of antiepileptic drugs
62	Christensen J (2013) Jama-J AM Med Assoc, V309, P1696	9	Holmes, LB (2012) Newer anticonvulsants: lamotrigine, topiramate and gabapentin
60	Meador K (2008) Epilepsy Res, V81, P1	7	Bobo, WV (2015) Trends in the use of antiepileptic drugs among pregnant women in the us, 2001-2007: a medication exposure in pregnancy risk evaluation program study
52	Molgaard-nielsen D (2011) Jama-J AM Med Assoc, V305, P1996	7	Vajda, FJE (2014) The efficacy of the newer antiepileptic drugs in controlling seizures in pregnancy
51	Jentink J (2010) New Engl J Med, V362, P2158	7	Voinescu, PE (2017) Delivery of a personalized treatment approach to women with epilepsy

**Note:**

Cluster label terms are bold.

**Table 7:** Cited and citing references of cluster #1 fetal malformations.

Cluster #1 fetal malformations			
Cited references		Citing articles	
Cites	Author (year) journal, volume, page	Coverage (%)	Author (year) title
161	Morrow J (2006) J Neurol Neurosur Ps, V77, P193	17	Pennell, PB (2004) Pregnancy in women who have epilepsy
106	Holmes LB (2001) New Engl J Med, V344, P1132	13	Hill, DS (2010) Teratogenic effects of antiepileptic drugs
92	Adab N (2004) J Neurol Neurosur Ps, V75, P1575	13	Ozyurek, H (2010) Effect of prenatal levetiracetam exposure on motor and **cognitive functions** of rat off spring
76	Artama M (2005) Neurology, V64, P1874	12	Ikonomidou, C (2010) Antiepileptic drugs and brain development
74	Wide K (2004) Acta Paediatr, V93, P174	12	Kaplan, PW (2004) Reproductive health effects and teratogenicity of antiepileptic drugs

**Note:**

Cluster label terms are bold.

### Analysis of burst keywords

**Figure 7:**
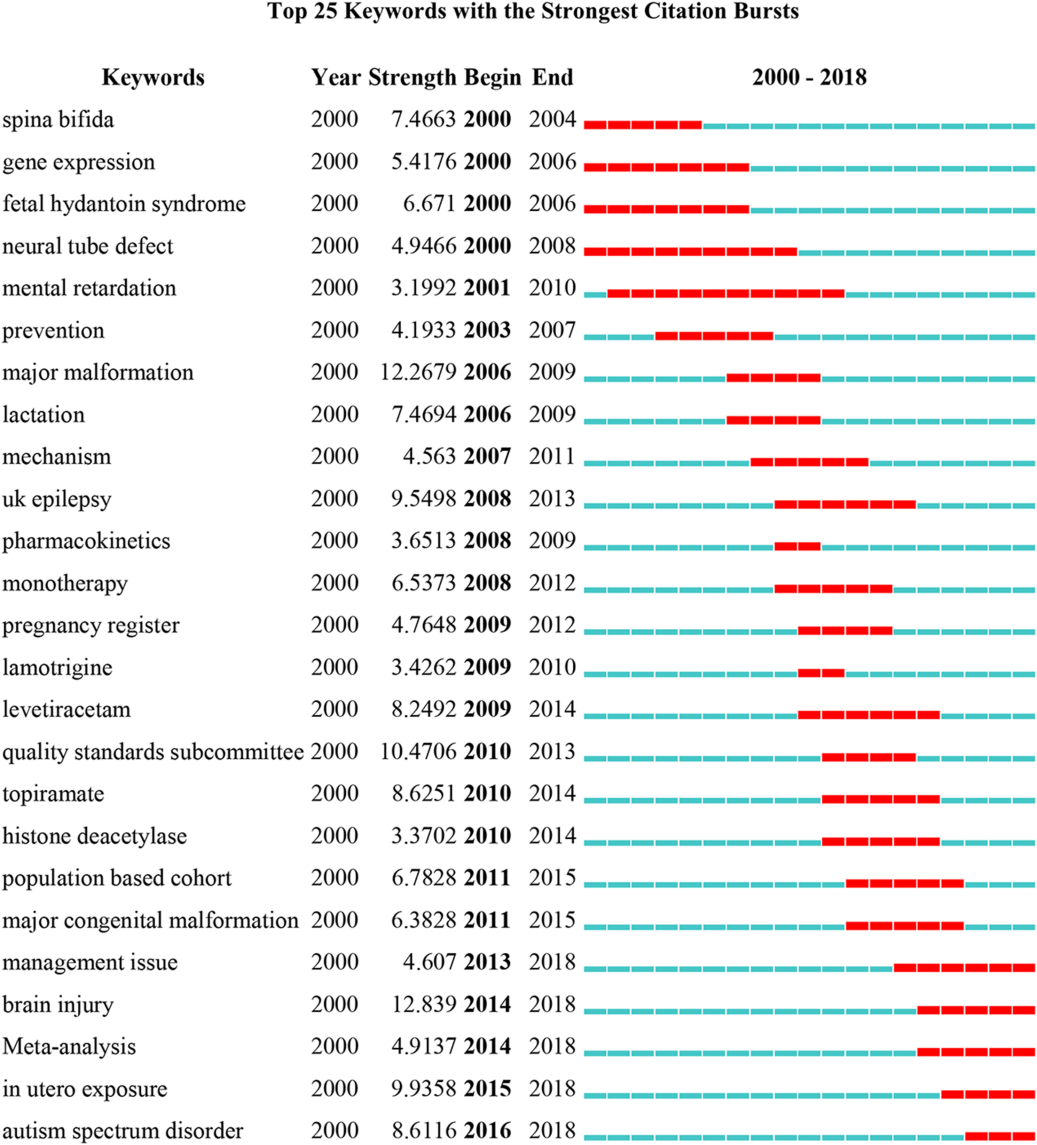
Top 25 keywords with the strongest citation bursts.

As a prominent representative of the research frontiers in a field of knowledge, burst keywords reflect the hotspots and frontiers in specific areas. As shown in Fig. 7, the time interval was plotted on the blue line and the period of burst keywords were plotted on the red line, which indicated the beginning and end of the time interval of each burst. “Management issue” (2013–2018), “brain injury” (2014–2018), “meta-analysis” (2014–2018), “in utero exposure” (2015–2018), and “autism spectrum disorder (ASD)” (2016–2018) were the keywords that had citation bursts after 2013.

## Discussion

We used CiteSpace, a science mapping software tool, for bibliometric analysis of relevant literature on epilepsy during pregnancy research, as well as a visual description of the research status and trends, which allowed for a systematic understanding of the past and future.

### General information

There were a total of 2,225 publications with a fluctuating growth trend over time. However, considering the cyclical nature of scientific research outputs, research in this area was generally increasing. The top 10 contributive countries/regions (two North American countries, five European countries, one Oceanian country, and two Asian countries) which were engaged in epilepsy during pregnancy research, contributed to 1,840 publications, accounting for 82.70% of the total number of publications. The US contributed 717 publications (about one-third of the total number of publications), reflecting its dominant position in epilepsy during pregnancy research. China and Turkey were the two-developing country in the list, which indicated they had made significant progress in epilepsy during pregnancy research over the past two decades. Furthermore, the most active collaborations were observed in Denmark, Canada, China, and the US.

Institutions with tremendous scientific research strength were observed to be mainly concentrated in higher education research institutions by examining both the publication counts and centrality, these institutions are important bases for medical scientific research and education. The top 10 institutions contributed to 447 articles, accounting for 20.09% of all publications. In this list, four of them (Harvard University, Emory University, Columbia University, and the University of California Los Angeles) were located in the US, indicating that institutions located in the US occupied the top rankings in terms of absolute contribution and relative influence, which is consistent with the analysis of contributions of countries in the field of epilepsy during pregnancy. Due to the close relationship between the level of health care and the speed of economic development, the institutional distribution table provides valuable information that helps researchers identify and choose appropriate collaborative institutions.

In terms of the top 15 academic journals, there were three journals: *Neurology* (8.055), *Pediatrics* (5.515), *Epilepsia* (5.067) had an IF score higher than 5, and these journals accounted for 11.87% (264/2,225) of total publications. The literature published in the journal of *Neurology* that involved in epilepsy during pregnancy research had the highest *H*-index (77), followed by *Epilepsia* (47), *Epilepsy & Behavior* (24), *Seizure: European Journal of Epilepsy* (20), and *Epilepsy Research* (20), indicating these journals were one of the most important information resources and the core journals in this area. According to the JCR 2017 standards, four journals were classified as Q1 contributed to 299 articles, accounting for 13.43% of all publications. And four journals were classified as Q2 contributed to 157 articles, accounting for 7.06% of all publications. Therefore, articles published in high-impact journals (classified as Q1, Q2) account for only one-fifth of the total numbers of publications, and the quality of related research in the field of epilepsy during pregnancy needs to be further improved.

The number of publications and count of citations could be adopted to evaluate the author’s absolute contribution and academic influence. Each of the top 10 authors identified in this analysis contributed to at least 19 publications. Therefore, they were referred to as “prolific authors.” Fortunately, five of these prolific authors (Tomson T, Meador KJ, Pennell PB, Vajda FJE, and Sabers A) were included in list of the top 10 co-cited authors, with regard to the annual co-citation counts, suggesting that these prolific authors consider both the number of articles and the quality of articles. Notably, Prof. Tomson had conducted a lot of earlier research, which laid a foundation for the development of this domain.

### Intellectual base

In bibliometrics, the contemporary developmental state of one discipline could be reflected by research hotspots, and the intellectual base of this field could be constituted by the references in these articles (Egghe & Rousseau, 2002). References with higher frequencies of citation overwhelmingly centered around the topic of the major congenital malformation (MCM) and cognitive function after fetal exposure to AEDs. A prospective study of Morrow from the UK Epilepsy and Pregnancy Register (Morrow et al., 2006) assessed the relative risk of MCM from in utero exposure to AEDs and showed that the MCM rate for polytherapy exposure was greater than for monotherapy. Further research by Tomson showed that the risk of MCMs could be affected by type of AEDs, dose and other variables, which ought to be considered under the situation of managing epilepsy in women of childbearing potential (Tomson et al., 2011). Holmes explored whether MCM was caused by maternal seizures itself or by AED exposure (Holmes et al., 2001). Particularly, a longer-term follow-up by Adab in children exposed to AEDs in utero (Shorvon, 2004) found that a lower verbal IQ and developmental delay may closely relate with recurrent tonic-clonic seizures during pregnancy. A large number of studies had shown that in utero exposure to valproic acid (VPA) was associated with increased risk of impaired cognitive function compared with other widely-used AEDs (Meador et al., 2009, 2013; Shorvon, 2004).

In the network map of co-citation references, Cluster #1 contained many nodes with purple rings of betweenness centrality, and Cluster #0 contained numerous nodes with red rings of citation bursts, indicating that this was the most recently formed cluster. Therefore, we particularly focused on cluster #0 to identify emerging trends in epilepsy during pregnancy. Eight most-cited references and eight citing articles in this cluster were selected, all among citations published in the past 10 years (showed in Table 6). An article by Eadie had the highest citation coverage of 18%, their primary concern was why maternal seizure control may deteriorate during pregnancy and how this may be avoided (Eadie, 2014). Other citing articles evaluated pregnancy and delivery complications in WWE (Borthen & Gilhus, 2012) or called for more high-quality studies of malformation risks of newer AEDs in pregnancy (Bobo et al., 2012; Holmes & Hernandez-Diaz, 2012; Vajda et al., 2014). Thus, future research in the field of epilepsy during pregnancy should focus on comprehensive management of pregnant and lactating women, and the evaluation of the efficacy and safety of newer AEDs through high-quality clinical research or systematic reviews, so as to provide evidence for rational clinical application.

### Research frontiers

#### Management issue

According to incomplete estimates, three to four newborns per 1,000 will be born to WWE (Kelly, 1984). Compared with general population, these women may encounter a 10-fold increase in risks of dying (Edey, Moran & Nashef, 2014), exceeding the twofold to threefold increase in standardized mortality rate throughout life for whole epileptic patients (Shorvon, 2004). Both maternal seizures and in utero exposure to AEDs posed a series of outstanding issues to WWE during various periods of pregnancy. Given all the challenges faced during pregnancy, as indicated by practice guidelines (Harden et al., 2009a, 2009b, 2009c), the pre-conception period was of the utmost importance for planning a secure, well-prepared and educated pregnancy. Pre-pregnancy counseling and planning by patients and physicians were keys to managing epilepsy and seizures during pregnancy.

#### Brain injury/neonatal seizures

Seizure occurs more frequently during the neonatal periods than at any other life period (Sands & McDonough, 2016). Hypoxic-ischemic encephalopathy caused by perinatal asphyxia was the most common etiology of neonatal seizures (Mitra et al., 2016). It remains controversial whether neonatal seizures themselves cause damage to the developing brain, or seizures may be a marker of more severe brain injury. There is still no consensus on what is the most appropriate treatment for neonatal seizures and how to assess the effects of treatment, especially for subclinical seizures (Kline-Fath et al., 2018). Currently, the first-generation AEDs are relatively ineffective in treating neonatal seizures. For electroencephalogram-confirmed or suspected seizures, phenobarbital is the first-line treatment in clinical practice (Clancy, 2006). Benzodiazepines (especially clonazepam, lorazepam, and midazolam) were also used for some cases of refractory epilepsy with phenobarbital failure (Glass, 2014). Although research data were scarce, the second-generation AEDs, which are widely accepted in adult and pediatric neurological practice, are being prescribed to treat neonatal seizures (Khan et al., 2013). In light of limited evidence, there is an urgent need for prospective, randomized, controlled trials and high-quality systematic, and meta-analyses to assess the efficacy and safety of these newer AEDs in neonatal seizures.

#### Systematic review and meta-analysis

According to Lund et al. (2016), it is unethical, unscientific, and wasteful to embark on new research without a systematic review of what is already known, especially when the subject involves humans or animals. Meta-analysis can be important in promoting the rapid development of science by quantifying current knowledge and identifying unknowns. Four of the six published systematic reviews with meta-analyses assessed pregnancy outcomes in WWE (Meador et al., 2008) and the safety of AEDs for neurological development in children exposed during pregnancy and breast feeding (Veroniki et al., 2017b). Particularly in recent years, attention has been on evaluating the risk of MCMs of in utero exposure to newer AEDs, such as topiramate (Alsaad, Chaudhry & Koren, 2015) and lamotrigine (Pariente et al., 2017). Two other studies used meta-analysis to evaluate the effect of epilepsy in pregnancy on fetal growth restriction (Chen et al., 2017b) and the efficacy and safety of eslicarbazepine acetate as add-on treatment for focal-onset seizures in pediatric patients (Lattanzi et al., 2018).

#### In utero exposure

These studies containing the keyword “in utero exposure” were primarily intended to evaluate the causal relationship exists between AEDs in utero exposure and adverse outcomes in offspring. Previous studies had focused on MCMs of AEDs in pregnancy and cognitive function after fetal exposure to AEDs. It is noteworthy that there were recent case reports of neonatal abstinence syndrome (NAS) for in utero exposure to carbamazepine (Passia et al., 2017), oxcarbazepine (Chen et al., 2017a; Rolnitsky, Merlob & Klinger, 2013), and gabapentin (Carrasco et al., 2015). NAS was most frequent after in utero exposure to opioids, while recent studies showed that exposure to AEDs during pregnancy may induce NAS. For infants within utero AED exposure, comprehensive assessments, and examinations were necessary for screening AED-induced NAS. In clinical practice, the physician faces the dilemma of balancing the risk of seizures to maternal and in utero exposure AEDs to developing fetuses. Researchers need to further clarify the mechanism of placental passage of AEDs at delivery, and to evaluate long-term outcomes for offspring with a history of AED in utero exposure.

#### Autism spectrum disorder

ASD is a developmental brain disorder characterized by limited and repetitive patterns of behavior, social and communication deficits, and is associated with difficulties with motor coordination (Main & Kulesza, 2017). Notwithstanding most ASD is idiopathic, it has been linked with hereditary factors and teratogens. The use of VPA during pregnancy significantly increases the risk of ASD in offspring (Nicolini & Fahnestock, 2018). In ASD treatment, research indicated that a ketogenic diet (Castro et al., 2017), astaxanthin (Al-Amin et al., 2015), zinc (Cezar et al., 2018), hydrogen-rich water (Guo et al., 2018), and agomelatine (Kumar, Sharma & Sharma, 2015) may improve behavioral disorder and oxidative stress in a prenatal VPA-induced mice model of autism. Molecular research should focus on solving this problems, both genetic/environmental interactions, and how they affect neurodevelopmental changes. Especially, since VPA is a histone deacetylase inhibitor, considering its role in epigenetic reprogramming is very important. The future directions should be to examine the etiology of autism and its biologic mechanism by employing genetic and environmental animal models.

### Strengths and limitations

To the best of our knowledge, this study is the first bibliometric analysis focus on epilepsy during pregnancy trends over the past two decades. There was no time limit for our literature retrieval, the data downloaded from WoSCC database covered the vast majority of articles in the field of epilepsy during pregnancy research, data analysis was relatively objective and comprehensive, which clearly showed the past and current status of epilepsy during pregnancy and predicted the future research frontier. However, this study consisted exclusively of original articles and reviews published in 2000–2018 and indexed by the web of science (WOS) database, since books, conference abstracts, and other journal publications were not included in the range of document screening, our data may not represent all of the literature. Furthermore, we only included articles written in English in our analysis, making the analysis incomplete to some extent. In terms of retrieval of databases, we only retrieved publications from the WoSCC database. Although other databases such as PubMed, Scopus, and Embase could provide a broader range of coverage of scientific literature, the references are not available. The WOS database is currently the main citation index database based on the mutual citation relationship between documents, which allows a scientific assessment of the following contents from the perspective of literature citation, including academic value of the literature and relevant researchers, institutions, journals, and national overall research output level. Thus, the WoSCC database may be the only appropriate choice. Finally, the results of this study were stable and highly reproducible, since this study covers the vast majority of papers from 2000 to 2018, and the latest published articles may not affect the final results.

## Conclusions

We constructed a series of science maps of journals, countries/regions, institutes, authors (co-cited authors), co-cited references, and citation burst keywords to identify the theme evolution and emerging trends in the intellectual landscape of this domain. Epilepsy during pregnancy is a subject matter with high development potential and bright future. A considerable number of papers were published in highly influential journals. The US had an important influence in this domain. The University of Melbourne, Harvard University, and the University of Queensland were identified as good research institutions for research collaboration. Prof. Pennell and Prof. Tomson were prominent leaders in this field. A major ongoing research trend was the MCM of AEDs in pregnancy and cognitive function after fetal exposure to AEDs. A more recent emerging trend focused on comprehensive management of pregnant and lactating women and the evaluation of the efficacy and safety of newer AEDs. Management issues, brain injury, meta-analysis, in utero exposure, and ASD may be the frontiers of epilepsy during pregnancy research in the next few years, and should be closely observed.

## Supplemental Information

10.7717/peerj.7115/supp-1 Supplemental Information 1 Search strategies.

10.7717/peerj.7115/supp-2 Supplemental Information 2 The number of publications by year, extracted from the Web of Science Core Collection database on March 7, 2019.

10.7717/peerj.7115/supp-3 Supplemental Information 3 The distribution of journals, extracted from the Web of Science Core Collection database on March 7, 2019.

10.7717/peerj.7115/supp-4 Supplemental Information 4 Impact factors, JCR categories and quartiles of the top 15 journals according to the JCR 2017 standards.

10.7717/peerj.7115/supp-5 Supplemental Information 5 The H-index of literature that involved in epilepsy during pregnancy research published in the top 15 journals, extracted from the Web of Science Core Collection database on March 7, 2019.

10.7717/peerj.7115/supp-6 Supplemental Information 6 The list of the top 50 most-cited references.

10.7717/peerj.7115/supp-7 Supplemental Information 7 Raw data that was imported into CiteSpace software.

10.7717/peerj.7115/supp-8 Supplemental Information 8 Raw data that was imported into the Online Analysis Platform of Literature Metrology.

10.7717/peerj.7115/supp-9 Supplemental Information 9 CiteSpace V 5.1.R8 SE, 62bit settings.

10.7717/peerj.7115/supp-10 Supplemental Information 10 The search history on epilepsy during pregnancy research that was extracted from the Web of Science Core Collection database on March 7, 2019 (Screenshot).

10.7717/peerj.7115/supp-11 Supplemental Information 11 The network map of co-citation clusters.

10.7717/peerj.7115/supp-12 Supplemental Information 12 The top 11 largest clusters of co-cited references that was generated by CiteSpace V (Screenshot).

10.7717/peerj.7115/supp-13 Supplemental Information 13 The network map of countries/regions distribution that was generated by CiteSpace V (Screenshot).

10.7717/peerj.7115/supp-14 Supplemental Information 14 The network map of institution distribution that was generated by CiteSpace V (Screenshot).

10.7717/peerj.7115/supp-15 Supplemental Information 15 The network map of productive authors that was generated by CiteSpace V (Screenshot).

10.7717/peerj.7115/supp-16 Supplemental Information 16 The network map of co-cited authors that was generated by CiteSpace V (Screenshot).

10.7717/peerj.7115/supp-17 Supplemental Information 17 The network map of co-citation reference that was generated by CiteSpace V (Screenshot).

10.7717/peerj.7115/supp-18 Supplemental Information 18 The network map of co-citation clusters that was generated by CiteSpace V (Screenshot).

10.7717/peerj.7115/supp-19 Supplemental Information 19 The timeline view of co-citation clusters that was generated by CiteSpace V (Screenshot).

## Additional Information and Declarations

### Data Availability

The following information was supplied regarding data availability: The raw data is available in the Supplemental Files.
