# Supplementary material for: Emerging trends and knowledge structure of epilepsy during pregnancy research for 2000–2018: a bibliometric analysis
Source: PeerJ. 2019 Jun 7;7:e7115. doi: 10.7717/peerj.7115 (PMC6557303; doi:10.7717/peerj.7115)
Supplement: Supplemental Information 1 [file peerj-07-7115-s001.doc]

***Search strategies***

#1：Title= (“convulsion*”) OR (“epilepsy*”) OR (“seizure*”)

#2：Title= (“antiepileptic*” ) OR ( “anticonvulsant*” ) OR ( “AED*” ) OR ( “phenytoin*” ) OR ( “phenobarbital*” ) OR ( “divalproex*” ) OR ( “valproic acid*” ) OR ( “carbamazepine*” ) OR ( “Oxcarbazepine*” ) OR ( “levetiracetam*” ) OR ( “gabapentin*” ) OR ( “lamotrigine*” ) OR ( “topiramate*”)

#3：TS= (“pregnancy*”) OR (“pregnancies*”) OR (“gestation*”) OR (“pregnant*”) OR (“maternal*”) OR (“fetus*”) OR (“foetus*”) OR (“newborn*”)

#4 = (#1 OR #2)

#5 = #4 AND #3
