# Supplementary material for: Emerging trends and knowledge structure of epilepsy during pregnancy research for 2000–2018: a bibliometric analysis
Source: PeerJ. 2019 Jun 7;7:e7115. doi: 10.7717/peerj.7115 (PMC6557303; doi:10.7717/peerj.7115)
Supplement: Supplemental Information 4 [file peerj-07-7115-s004.zip › 7/1. InCites Journal Citation Reports(NEUROLOGY).pdf]

2017 Journal Performance Data for: NEUROLOGY

ISSN: 0028-3878
eISSN: 1526-632X
LIPPINCOTT WILLIAMS & WILKINS
TWO COMMERCE SQ, 2001 MARKET ST, PHILADELPHIA, PA 19103
USA

TITLES
ISO: Neurology
JCR Abbrev: NEUROLOGY

LANGUAGES
English

CATEGORIES
CLINICAL NEUROLOGY - SCIE

PUBLICATION FREQUENCY
48 issues/year

Downloaded on 2019/4/19 上午11:28:47 from Journal Citation Reports (©2019 Clarivate Analytics)

Current Year

The data in the two graphs below and in the Journal Impact Factor calculation panels represent citation activity in 2017 to items published in the journal in the prior two years. They detail the components of the Journal Impact Factor. Use the "All Years" tab to access key metrics and additional data for the current year and all prior years for this journal.

2017 Journal Impact Factor & percentile rank in category for: NEUROLOGY

8.055
2017 Journal Impact Factor

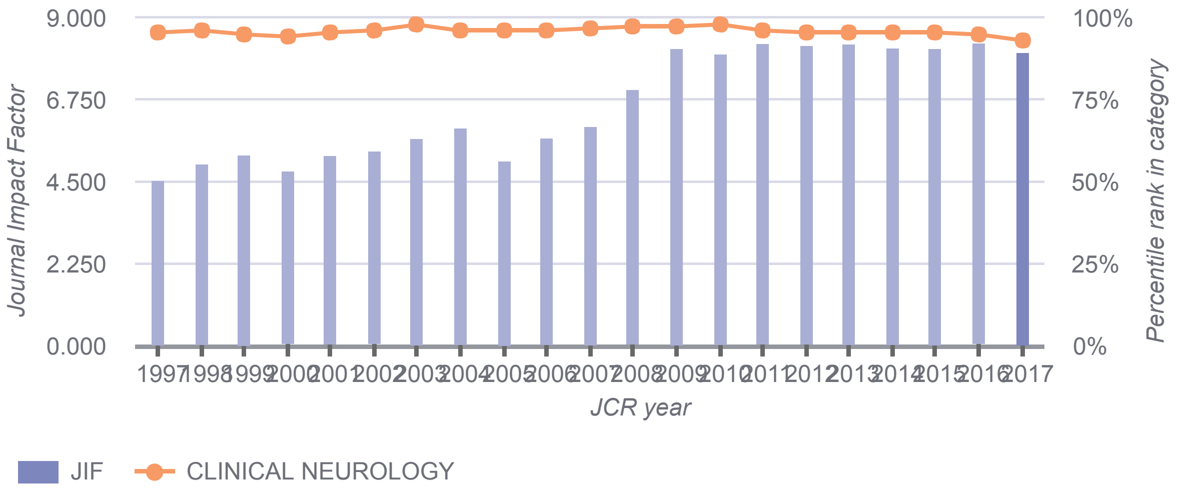

2017 JIF Citation Distribution for: NEUROLOGY

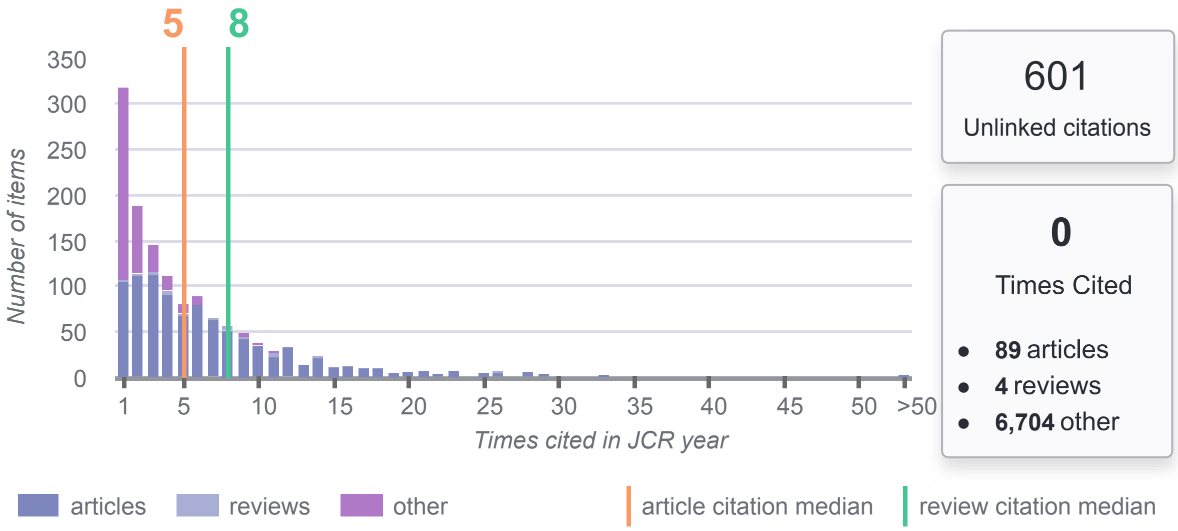

601
Unlinked citations

0
Times Cited

- **89** articles
- **4** reviews
- **6,704** other

Downloaded on 2019/4/19 上午11:28:47 from Journal Citation Reports (©2019 Clarivate Analytics)

Journal Impact Factor Calculation

$$\text{2017 Journal Impact Factor} = \frac{8{,}530}{1{,}072} = 7.957$$

How is Journal Impact Factor Calculated?

$$\text{JIF} = \frac{\text{Citations in 2017 to items published in } \mathbf{2015\ (4{,}555) + 2016\ (3{,}975)}}{\text{Number of citable items in } \mathbf{2015\ (502) + 2016\ (570)}} = \frac{8{,}530}{1{,}072}$$

Downloaded on 2019/4/19 上午11:28:47 from Journal Citation Reports (©2019 Clarivate Analytics)

Journal Impact Factor contributing items

Citable items in 2016 and 2015 (1,072)

TITLE	CITATIONS COUNTED TOWARDS JIF
International consensus diagnostic criteria for neuromyelitis optica spectrum disorders<br>By: Wingerchuk, Dean M.; Jacob, Anu; Jarius, Sven; Lana-Peixoto, Marco; Levy, Michael; et al.<br>**Volume: 85 Page: 177-189 Accession number: WOS:000357804900012**<br>**Document Type: Review**	238
Results from a phase 1 study of nusinersen (ISIS-SMNRx) in children with spinal muscular atrophy<br>By: Chiriboga, Claudia A.; Swoboda, Kathryn J.; Darras, Basil T.; Iannaccone, Susan T.; Montes, Jacqueline; et al.<br>**Volume: 86 Page: 890-897 Accession number: WOS:000371833600004**<br>**Document Type: Article**	70
A randomized, double-blind, placebo-controlled trial of resveratrol for Alzheimer disease<br>By: Turner, R. Scott; Aisen, Paul S.; Thomas, Ronald G.; Craft, Suzanne; van Dyck, Christopher H.; et al.<br>**Volume: 85 Page: 1383-1391 Accession number: WOS:000363288600006**<br>**Document Type: Article**	52
Long-term efficacy and safety of thalamic stimulation for drug-resistant partial epilepsy<br>By: Salanova, Vicenta; Sandok, Evan; Handforth, Adrian; Stern, John M.; Chung, Steve; et al.<br>**Volume: 84 Page: 1017-1025 Accession number: WOS:000351458100016**<br>**Document Type: Article**	45
MRI characteristics of neuromyelitis optica spectrum disorder An international update<br>By: Kim, Ho Jin; Wuerfel, Jens; Banwell, Brenda L.; Villoslada, Pablo; Saiz, Albert; et al.<br>**Volume: 84 Page: 1165-1173 Accession number: WOS:000351458600020**<br>**Document Type: Review**	44
Parkinson risk in idiopathic REM sleep behavior disorder Preparing for neuroprotective trials<br>By: Postuma, Ronald B.; Gagnon, Jean-Francois; Bertrand, Josie-Anne; Marchand, Daphne Genier; Montplaisir, Jacques Y.<br>**Volume: 84 Page: 1104-1113 Accession number: WOS:000351458600012**<br>**Document Type: Article**	41
Accuracy of clinical diagnosis of Parkinson disease A systematic review and meta-analysis<br>By: Rizzo, Giovanni; Copetti, Massimiliano; Arcuti, Simona; Martino, Davide; Fontana, Andrea; et al.<br>**Volume: 86 Page: 566-576 Accession number: WOS:000369541200012**<br>**Document Type: Review**	39

Downloaded on 2019/4/19 上午11:28:47 from Journal Citation Reports (©2019 Clarivate Analytics)

Citations in 2017 (8,530)

TITLE	CITATIONS COUNTED TOWARDS JIF
NEUROLOGY	481
JOURNAL OF ALZHEIMERS DISEASE	136
JOURNAL OF THE NEUROLOGICAL SCIENCES	129
PLOS ONE	119
JOURNAL OF NEUROLOGY	115
MOVEMENT DISORDERS	109
FRONTIERS IN NEUROLOGY	104
STROKE	102
SCIENTIFIC REPORTS	95
MULTIPLE SCLEROSIS JOURNAL	92

Downloaded on 2019/4/19 上午11:28:47 from Journal Citation Reports (©2019 Clarivate Analytics)

Key Indicators 2017

IMPACT METRICS		INFLUENCE METRICS		SOURCE METRICS	
**Total Cites**	88,499	**Eigenfactor Score**	0.11500	**Citable Items**	529
**Journal Impact Factor**	8.055	**Article Influence Score**	3.175	**% Articles in Citable Items**	95.46
**5 Year Impact Factor**	8.716	**Normalized Eigenfactor**	13.48500	**Average JIF Percentile**	93.655
**Immediacy Index**	1.994			**Cited Half-Life**	11.1
**Impact Factor Without Journal Self Cites**	7.600			**Citing Half-Life**	6.5

Source data

Journal source data 2017

	Articles	Reviews	Combined(C)	Other(O)	Percentage(C/(C+O))
**Number in JCR Year 2017 (A)**	505	24	529	680	43%
**Number of References (B)**	15,223	1,123	16,346	3,661	81%
**Ratio (B/A)**	30.1	46.8	30.9	5.4	

Downloaded on 2019/4/19 上午11:28:47 from Journal Citation Reports (©2019 Clarivate Analytics)

Box plot

Category Box Plot 2017

Category Box Plot

The category box plot depicts the distribution of Impact Factors for all journals in the category. The horizontal line that forms the top of the box is the 75th percentile (Q1). The horizontal line that forms the bottom is the 25th percentile (Q3). The horizontal line that intersects the box is the median Impact Factor for the category. Horizontal lines above and below the box, called whiskers, represent maximum and minimum values.
The top whisker is the smaller of the following two values:
the maximum Impact Factor (IF)
Q1 IF + 3.5(Q1 IF - Q3 IF)
The bottom whisker is the larger of the following two values:
the minimum Impact Factor (IF)
Q1 IF - 3.5(Q1 IF - Q3 IF)
Box Plots are provided for the current JCR year for each of the categories in which the journal is indexed.

NEUROLOGY, IF: 8.055

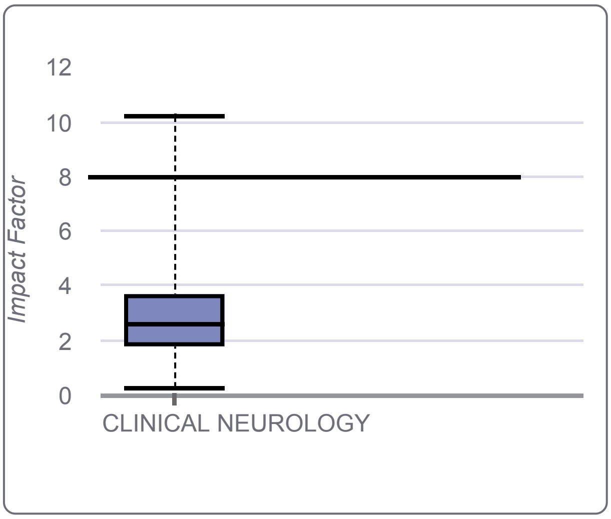

Downloaded on 2019/4/19 上午11:28:47 from Journal Citation Reports (©2019 Clarivate Analytics)

Rank

Rank 2017

JCR Impact Factor

JCR Year	CLINICAL NEUROLOGY		
	Rank	Quartile	JIF Percentile
2017	13/197	Q1	93.655
2016	9/194	Q1	95.619
2015	8/193	Q1	96.114
2014	8/192	Q1	96.094
2013	8/194	Q1	96.134
2012	8/193	Q1	96.114
2011	7/192	Q1	96.615
2010	4/185	Q1	98.108
2009	4/167	Q1	97.904
2008	4/156	Q1	97.756
2007	5/146	Q1	96.918
2006	6/147	Q1	96.259
2005	6/148	Q1	96.284
2004	5/140	Q1	96.786
2003	3/135	Q1	98.148
2002	5/138	Q1	96.739
2001	6/136	Q1	95.956
2000	8/137	Q1	94.526
1999	7/132	Q1	95.076
1998	5/125	Q1	96.400

Downloaded on 2019/4/19 上午11:28:47 from Journal Citation Reports (©2019 Clarivate Analytics)

Downloaded on 2019/4/19 上午11:28:47 from Journal Citation Reports (©2019 Clarivate Analytics)

ESI Total Citations 2017

Rank

JCR Year	NEUROSCIENCE & BEHAVIOR
2017	4/346-Q1
2016	4/345-Q1
2015	4/344-Q1
2014	4/337-Q1
2013	2/339-Q1

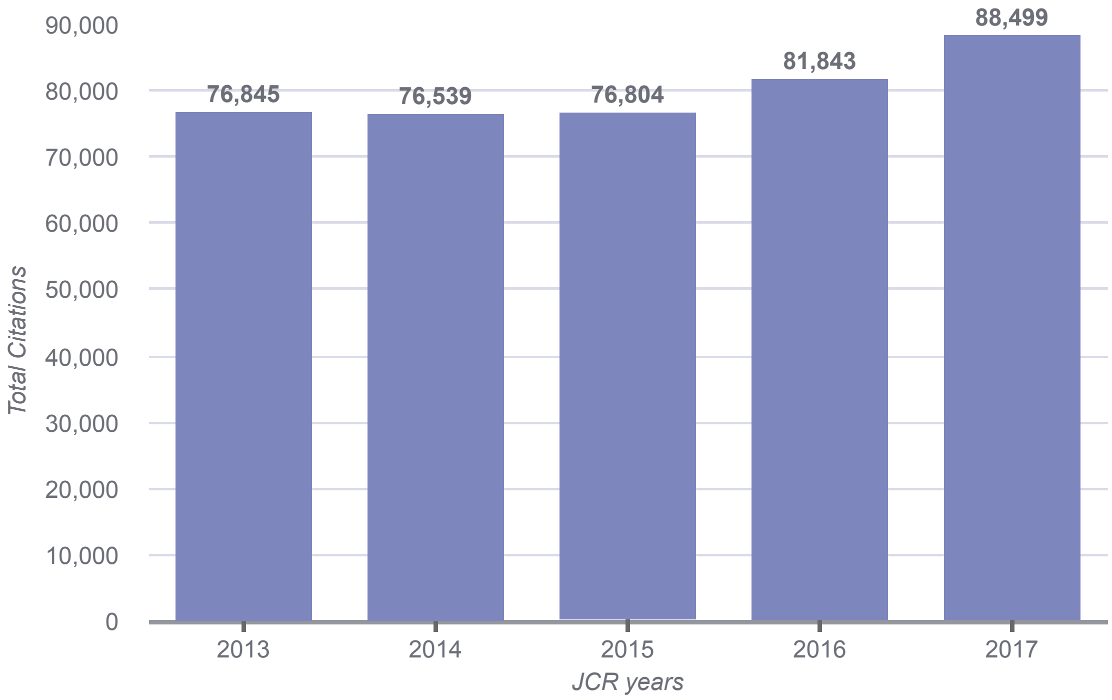

Downloaded on 2019/4/19 上午11:28:47 from Journal Citation Reports (©2019 Clarivate Analytics)

Cited Journal Data

Cited Half-Life Data

Customize columns

Cited Year	2017	2016	2015	2014	2013	2012	2011	2010	2009	2008	20[illegible]
#Cites from 2017	1,055	3,975	4,555	4,824	5,207	4,448	4,071	4,020	3,805	3,814	[illegible]
Cumulative %	1.19%	5.68%	10.83%	16.28%	22.17%	27.19%	31.79%	36.33%	40.63%	44.94%	10[illegible]

Cited Journal Graph 2017

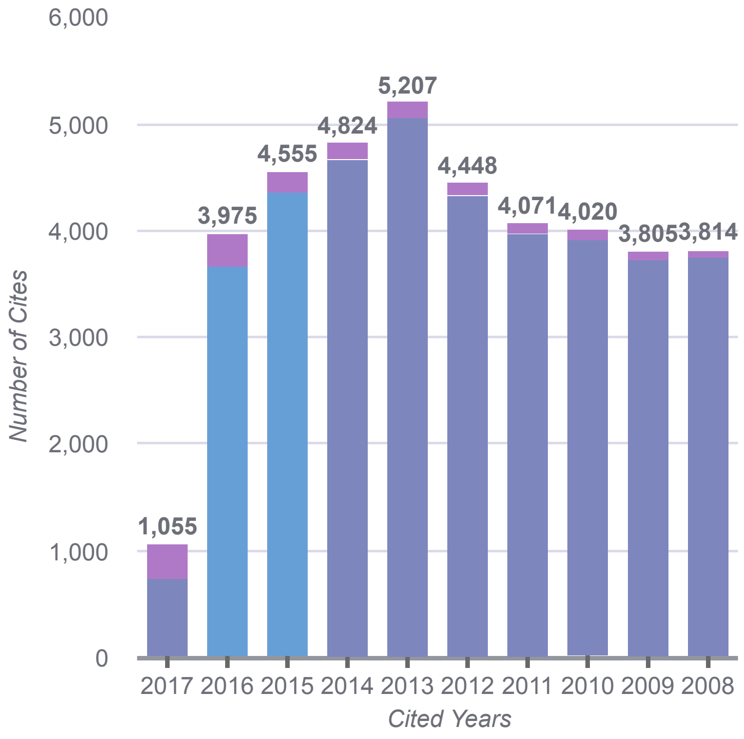

CITED JOURNAL GRAPH

The Cited Journal Graph shows the distribution (by cited year) of citations published in journals during the JCR year to items published in the Journal during the last 10 years.

The white/grey division indicates the cited half-life (if < 10.0). Half of the citations are to items that were published more recently than the cited half-life.

The two light-blue columns indicate citations used to calculate the Impact Factor (always the 2nd and 3rd columns).

Downloaded on 2019/4/19 上午11:28:47 from Journal Citation Reports (©2019 Clarivate Analytics)

Cited Journal Data

Customize columns

	Impact	Citing Journal	All Yrs	2017	2016	2015	2014	2013	2012	2011	2010	2009	2
		ALL Journals	88,499	1,055	3,975	4,555	4,824	5,207	4,448	4,071	4,020	3,805	3
		ALL OTHERS (1297)	1,297	10	31	48	60	81	58	52	55	48	
1	8.055	NEUROLOGY	2,276	317	298	183	159	148	120	100	95	74	
2	3.476	J ALZHEIMERS DIS	1,699	8	54	82	70	106	75	100	67	70	
3	2.766	PLOS ONE	1,474	9	53	66	85	83	77	59	77	82	
4	4.122	SCI REP-UK	1,243	12	36	59	77	80	64	60	73	57	
5	2.448	J NEUROL SCI	1,141	11	66	63	72	66	48	49	56	51	
6	3.508	FRONT NEUROL	1,129	19	54	50	62	57	48	59	43	47	
7	3.783	J NEUROL	830	11	57	58	53	63	46	33	42	43	
8	8.324	MOVEMENT DISORD	797	7	50	59	56	52	35	41	34	41	
9	5.280	MULT SCLER J	709	8	21	71	49	55	32	54	54	37	
10	2.371	INT REV NEUROBIOL	655	5	25	31	44	40	26	16	37	33	
11	4.454	NEUROBIOL AGING	655	6	27	35	28	47	38	44	27	32	
12	6.239	STROKE	638	14	61	41	43	54	59	36	34	36	
13	2.600	EPILEPSY BEHAV	604	5	29	30	25	26	44	28	33	24	

Rows 1 - 15 of 3,301 *(use csv export to download the full table)*

Downloaded on 2019/4/19 上午11:28:47 from Journal Citation Reports (©2019 Clarivate Analytics)

Citing Journal Data

Citing Half-Life Data

Customize columns

Citing Year	2017	2016	2015	2014	2013	2012	2011	2010	2009	2008	2
#Cites from 2017	671	1,939	1,955	1,769	1,565	1,368	1,234	1,142	901	786	
Cumulative %	3.35%	13.05%	22.82%	31.66%	39.48%	46.32%	52.49%	58.19%	62.70%	66.63%	1

Citing Journal Graph 2017

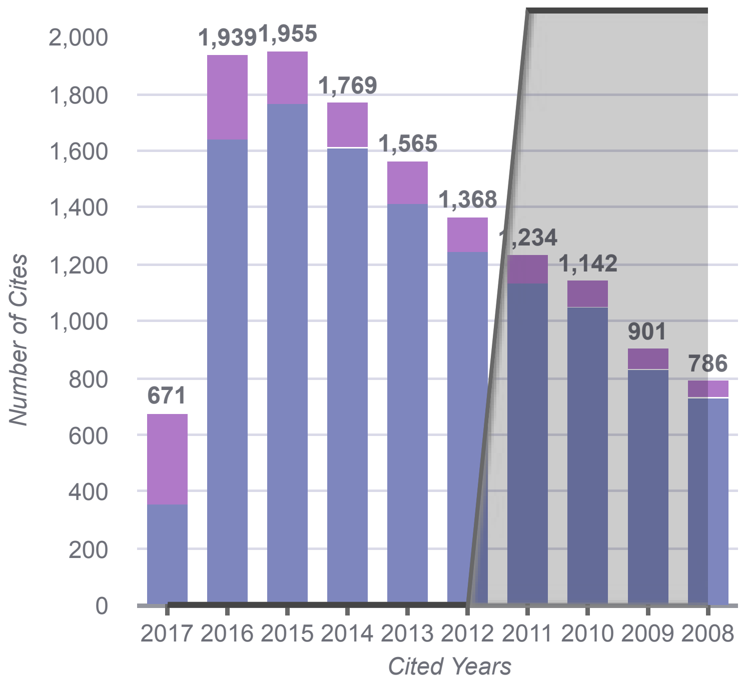

CITING JOURNAL GRAPH

The Citing Journal Graph shows the distribution (by cited year) of citations published in the Journal during the JCR year to items published in journals during the last 10 years.

The white/grey division indicates the citing half-life (if < 10.0). Half of the citations are to items that were published more recently than the citing half-life.

Downloaded on 2019/4/19 上午11:28:47 from Journal Citation Reports (©2019 Clarivate Analytics)

Citing Journal Data

Customize columns

	Impact	Cited Journal	All Yrs	2017	2016	2015	2014	2013	2012	2011	2010	2009	20[illegible]
		ALL Journals	20,007	671	1,939	1,955	1,769	1,565	1,368	1,234	1,142	901	7[illegible]
		ALL OTHERS (1660)	1,660	26	152	164	141	105	76	81	67	47	[illegible]
1	8.055	NEUROLOGY	2,276	317	298	183	159	148	120	100	95	74	[illegible]
2	6.239	STROKE	786	10	53	67	80	63	41	55	57	58	[illegible]
3	10.250	ANN NEUROL	520	8	41	44	41	29	26	66	31	25	[illegible]
4	27.144	LANCET NEUROL	509	9	67	42	63	73	44	36	56	38	[illegible]
5	10.848	BRAIN	501	4	39	53	26	35	38	43	32	26	[illegible]
6	7.144	J NEUROL NEUROSUR PS	388	10	27	23	38	30	32	19	21	14	[illegible]
7	79.260	NEW ENGL J MED	380	0	43	43	10	36	22	23	16	11	[illegible]
8	5.067	EPILEPSIA	315	7	12	19	24	31	24	27	33	10	[illegible]
9	8.324	MOVEMENT DISORD	294	7	21	40	17	25	20	21	27	7	[illegible]
10	53.254	LANCET	289	4	29	21	27	10	22	8	21	13	
11	47.661	JAMA-J AM MED ASSOC	250	5	19	34	24	11	8	26	6	5	
12	2.766	PLOS ONE	235	2	23	45	44	46	33	15	13	5	
13		ARCH NEUROL-CHICAGO	204	0	0	0	0	0	25	14	13	13	

Rows 1 - 15 of 1,142 *(use csv export to download the full table)*

Downloaded on 2019/4/19 上午11:28:47 from Journal Citation Reports (©2019 Clarivate Analytics)

Metric trend

Metric Trend

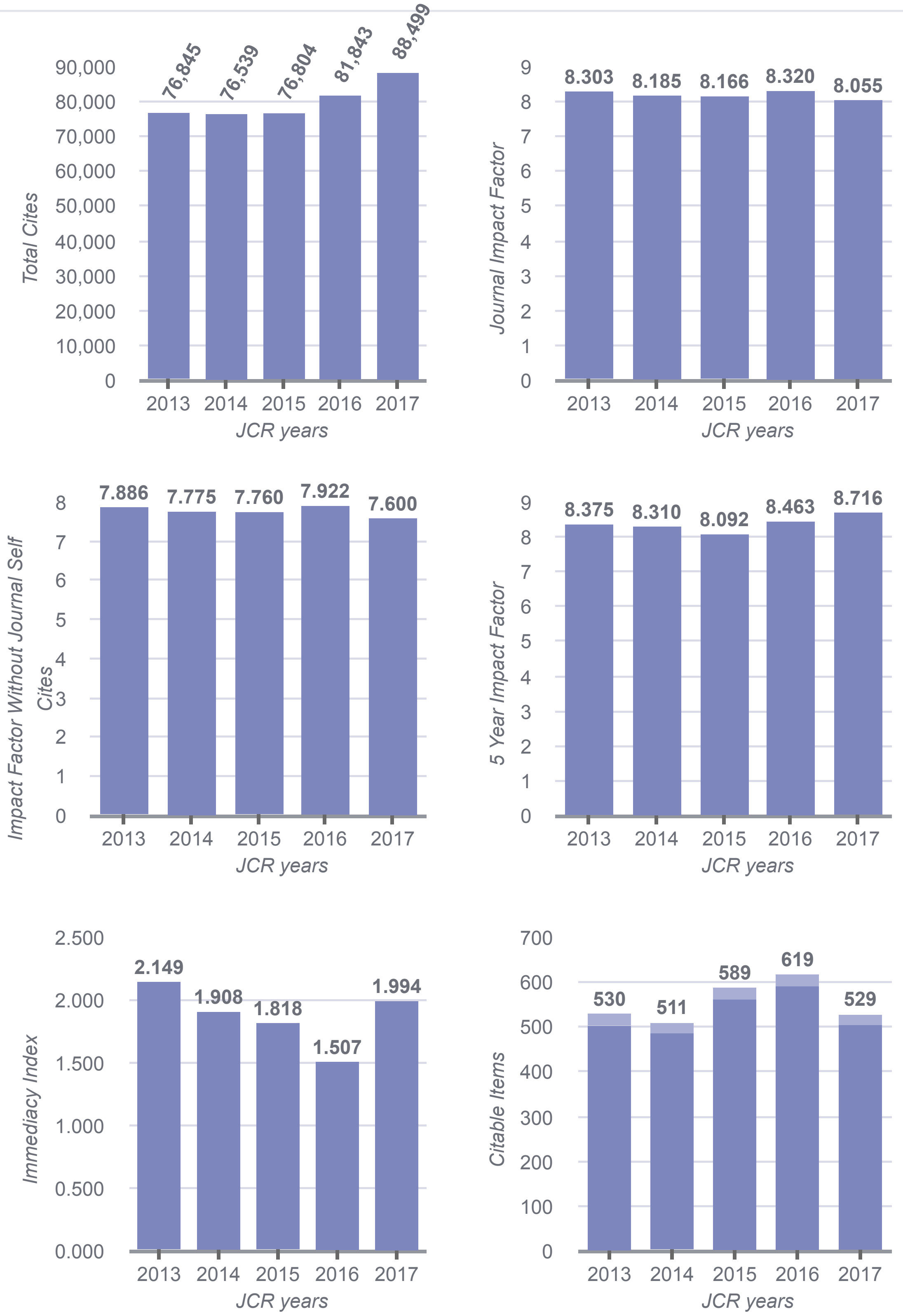

Downloaded on 2019/4/19 上午11:28:47 from Journal Citation Reports (©2019 Clarivate Analytics)

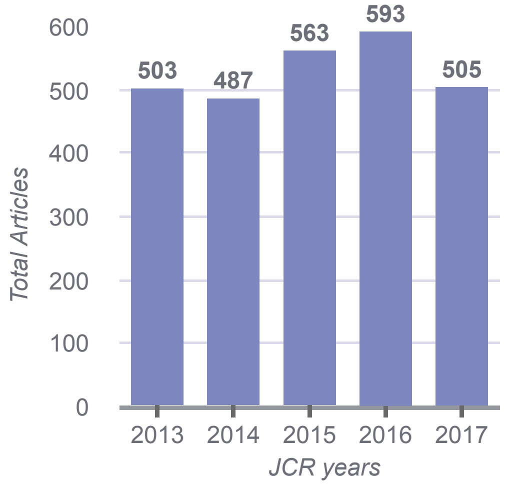

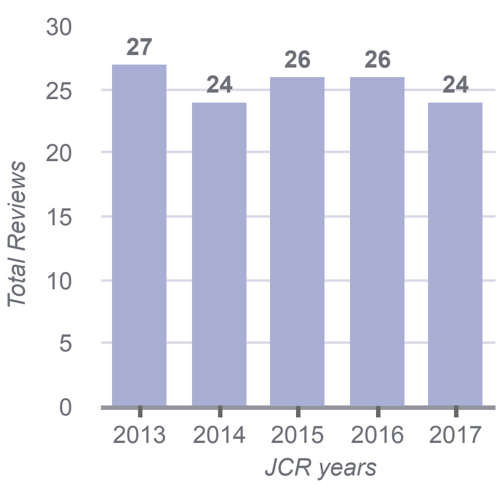

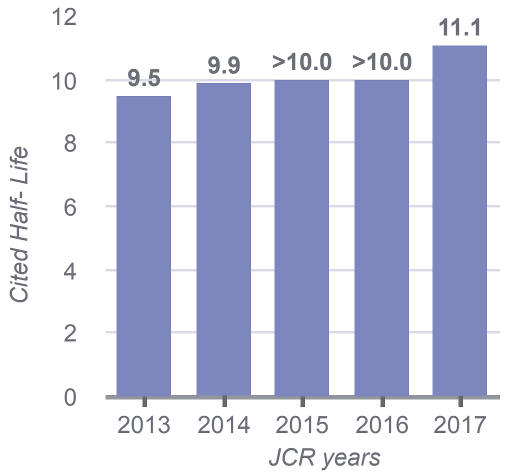

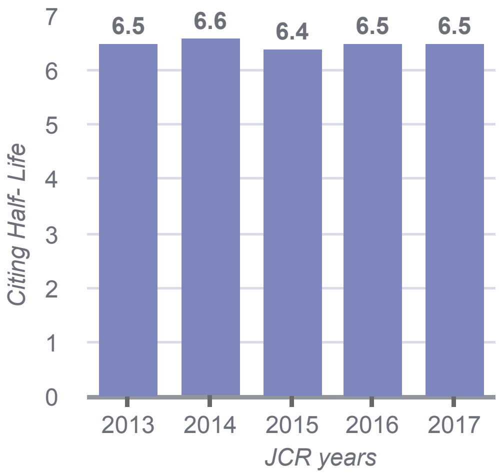

Downloaded on 2019/4/19 上午11:28:47 from Journal Citation Reports (©2019 Clarivate Analytics)

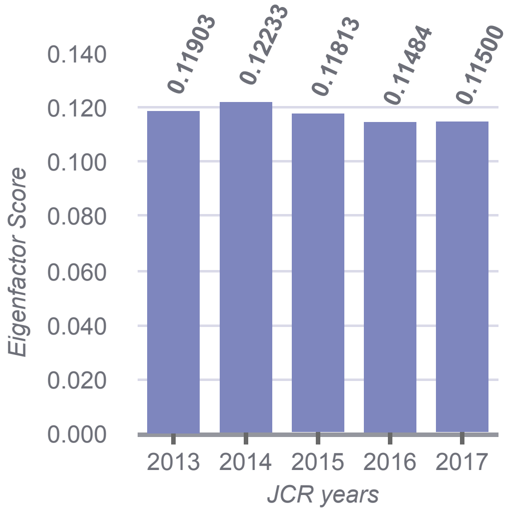

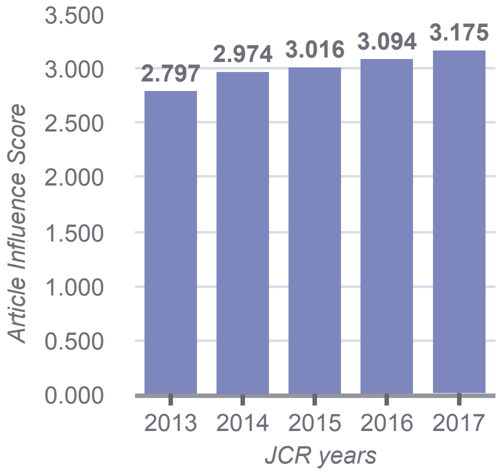

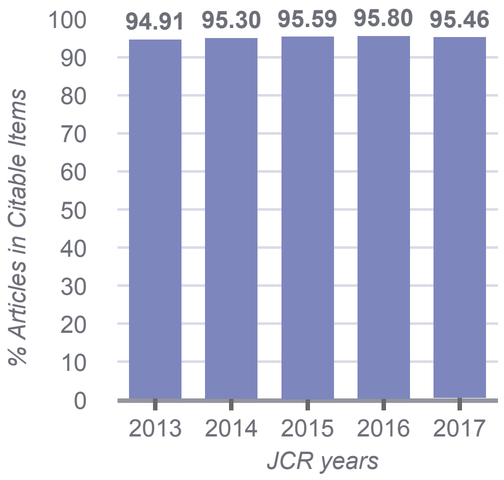

Downloaded on 2019/4/19 上午11:28:47 from Journal Citation Reports (©2019 Clarivate Analytics)

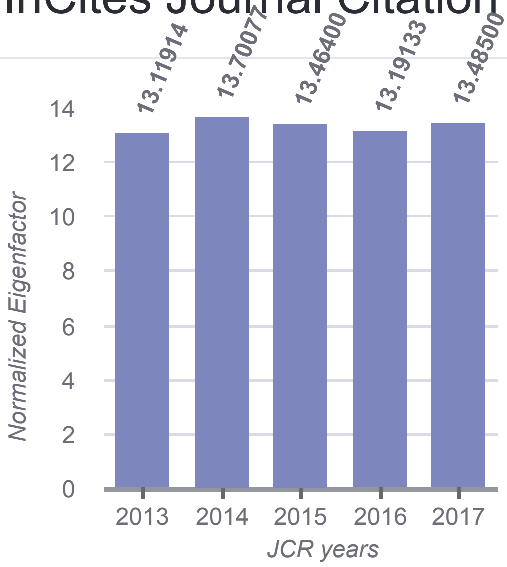

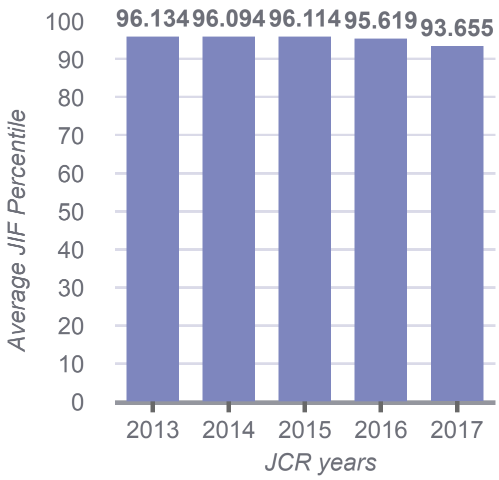

Downloaded on 2019/4/19 上午11:28:47 from Journal Citation Reports (©2019 Clarivate Analytics)

These data summarize the characteristics of the journal's published content for the most recent three years, that is, 2017 and the two prior years, combined. This information is based on all listed authors and addresses. It is meant to be descriptive rather than comparative.

Contributions by country/region

country	count
1. USA	5,809
2. Canada	727
3. England	702
4. GERMANY (FED REP GER)	619
5. Italy	533
6. France	338
7. Netherlands	330
8. Switzerland	265
9. Australia	260
10. Spain	232

Contributions by organizations

organization	count
1. HARVARD UNIVERSITY	661
2. UNIVERSITY OF CALIFORNIA SYSTEM	521
3. MAYO CLINIC	485
4. VA BOSTON HEALTHCARE SYSTEM	459
5. MASSACHUSETTS GENERAL HOSPITAL	362
6. UNIVERSITY OF LONDON	349
7. UNIVERSITY OF TEXAS SYSTEM	236
- COLUMBIA UNIVERSITY	236
9. UNIVERSITY OF PENNSYLVANIA	230
10. JOHNS HOPKINS UNIVERSITY	227

Downloaded on 2019/4/19 上午11:28:47 from Journal Citation Reports (©2019 Clarivate Analytics)
