# Supplementary material for: Emerging trends and knowledge structure of epilepsy during pregnancy research for 2000–2018: a bibliometric analysis
Source: PeerJ. 2019 Jun 7;7:e7115. doi: 10.7717/peerj.7115 (PMC6557303; doi:10.7717/peerj.7115)
Supplement: Supplemental Information 4 [file peerj-07-7115-s004.zip › 7/10. InCites Journal Citation Reports(REPRODUCTIVE TOXICOLOGY).pdf]

2017 Journal Performance Data for: EPILEPSY RESEARCH

ISSN: 0920-1211
eISSN: 1872-6844
ELSEVIER SCIENCE BV
PO BOX 211, 1000 AE AMSTERDAM, NETHERLANDS
NETHERLANDS

TITLES
ISO: Epilepsy Res.
JCR Abbrev: EPILEPSY RES

LANGUAGES
English

CATEGORIES
CLINICAL NEUROLOGY - SCIE

PUBLICATION FREQUENCY
10 issues/year

Downloaded on 2019/4/19 上午11:44:02 from Journal Citation Reports (©2019 Clarivate Analytics)

Current Year

The data in the two graphs below and in the Journal Impact Factor calculation panels represent citation activity in 2017 to items published in the journal in the prior two years. They detail the components of the Journal Impact Factor. Use the "All Years" tab to access key metrics and additional data for the current year and all prior years for this journal.

2017 Journal Impact Factor & percentile rank in category for: EPILEPSY RESEARCH

2.491

2017 Journal Impact Factor

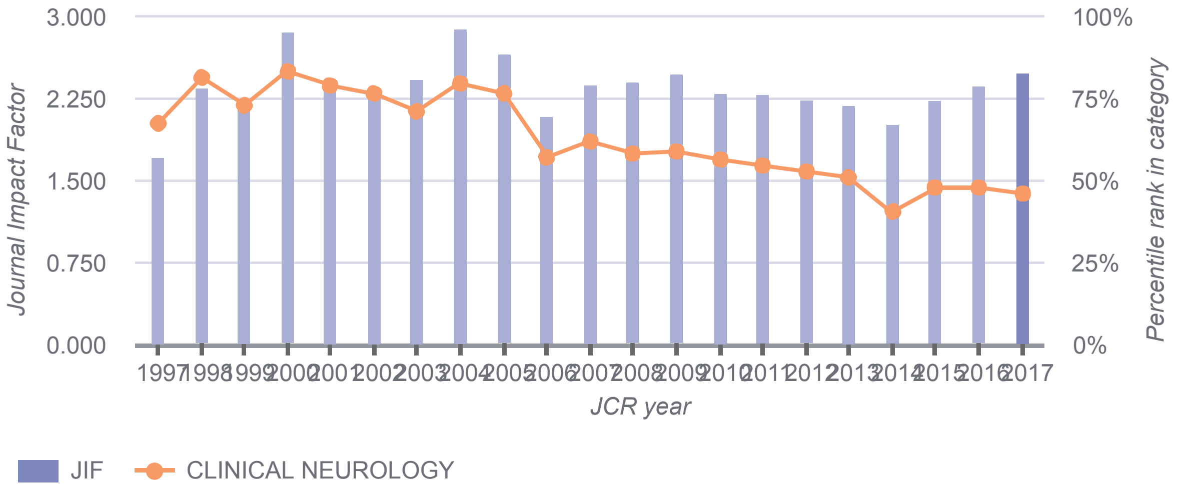

2017 JIF Citation Distribution for: EPILEPSY RESEARCH

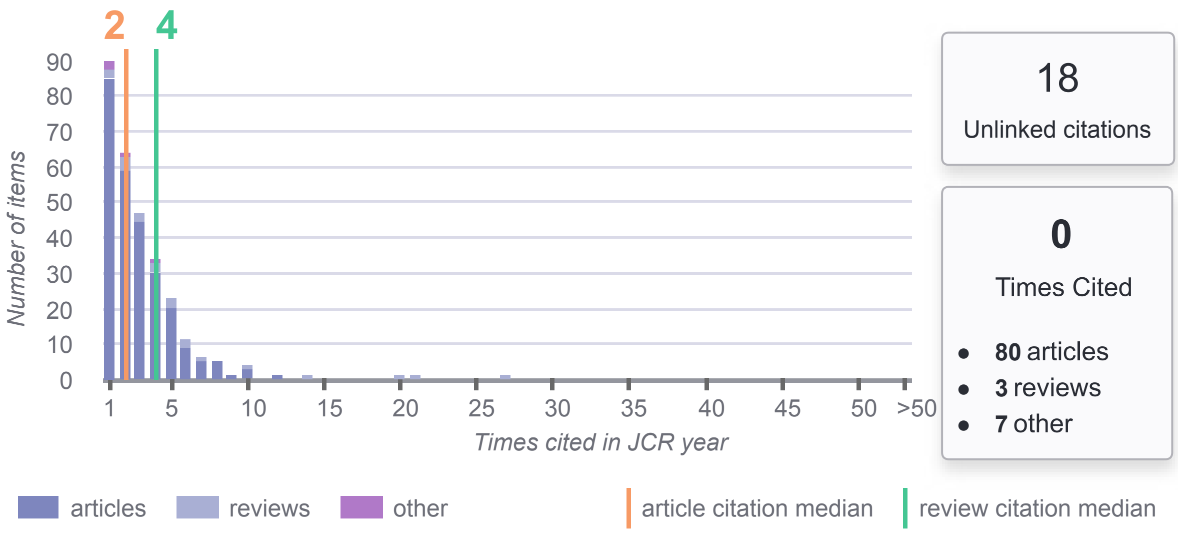

18

Unlinked citations

0

Times Cited

- **80** articles
- **3** reviews
- **7** other

Downloaded on 2019/4/19 上午11:44:02 from Journal Citation Reports (©2019 Clarivate Analytics)

Journal Impact Factor Calculation

$$\text{2017 Journal Impact Factor} = \frac{919}{369} = 2.491$$

How is Journal Impact Factor Calculated?

$$\text{JIF} = \frac{\text{Citations in 2017 to items published in } \mathbf{2015\ (534) + 2016\ (385)}}{\text{Number of citable items in } \mathbf{2015\ (185) + 2016\ (184)}} = \frac{919}{369}$$

Downloaded on 2019/4/19 上午11:44:02 from Journal Citation Reports (©2019 Clarivate Analytics)

Journal Impact Factor contributing items

Citable items in 2016 and 2015 (369)

TITLE	CITATIONS COUNTED TOWARDS JIF
Progress report on new antiepileptic drugs: A summary of the Twelfth Eilat Conference (EILAT XII)<br>By: Bialer, Meir; Johannessen, Svein I.; Levy, Rene H.; Perucca, Emilio; Tomson, Torbjorn; et al.<br>**Volume: 111 Page: 85-141 Accession number: WOS:000352679400011**<br>**Document Type: Review**	27
Current understanding of the mechanism of action of the antiepileptic drug lacosamide<br>By: Rogawski, Michael A.; Tofighy, Azita; White, H. Steve; Matagne, Alain; Wolff, Christian<br>**Volume: 110 Page: 189-205 Accession number: WOS:000349592800025**<br>**Document Type: Review**	21
Fit for purpose application of currently existing animal models in the discovery of novel epilepsy therapies<br>By: Loescher, Wolfgang<br>**Volume: 126 Page: 157-184 Accession number: WOS:000383825200022**<br>**Document Type: Review**	20
Juvenile myoclonic epilepsy: A system disorder of the brain<br>By: Wolf, Peter; Targas Yacubian, Elza Marcia; Avanzini, Giuliano; Sander, Thomas; Schmitz, Bettina; et al.<br>**Volume: 114 Page: 2-12 Accession number: WOS:000357438400002**<br>**Document Type: Review**	14
Safety, efficacy and outcome-related factors of perampanel over 12 months in a real-world setting: The FYDATA study<br>By: Villanueva, V.; Gonzalez-Giraldez, B.; Mauri, J. A.; Camacho, J. L.; Suller, A.; et al.<br>**Volume: 126 Page: 201-210 Accession number: WOS:000383825200025**<br>**Document Type: Article**	12
The pathophysiology of cardiac dysfunction in epilepsy<br>By: Ravindran, Krishnan; Powell, Kim L.; Todaro, Marian; O'Brien, Terence J.<br>**Volume: 127 Page: 19-29 Accession number: WOS:000401090200004**<br>**Document Type: Review**	10
Mortality in Dravet syndrome<br>By: Cooper, Monica S.; Lerman-Sagie, Tally; Mclellan, Ailsa; Pelekanos, James; Ramesh, Venkateswaran; et al.<br>**Volume: 128 Page: 43-47 Accession number: WOS:000390742500007**<br>**Document Type: Article**	10

Downloaded on 2019/4/19 上午11:44:02 from Journal Citation Reports (©2019 Clarivate Analytics)

Citations in 2017 (919)

TITLE	CITATIONS COUNTED TOWARDS JIF
EPILEPSY & BEHAVIOR	71
EPILEPSIA	55
SEIZURE-EUROPEAN JOURNAL OF EPILEPSY	53
EPILEPSY RESEARCH	52
CURRENT PHARMACEUTICAL DESIGN	28
FRONTIERS IN NEUROLOGY	22
NEUROCHEMICAL RESEARCH	15
SCIENTIFIC REPORTS	14
EXPERT OPINION ON PHARMACOTHERAPY	13
PEDIATRIC NEUROLOGY	9

Downloaded on 2019/4/19 上午11:44:02 from Journal Citation Reports (©2019 Clarivate Analytics)

Key Indicators 2017

IMPACT METRICS		INFLUENCE METRICS		SOURCE METRICS	
**Total Cites**	6,821	**Eigenfactor Score**	0.01000	**Citable Items**	182
**Journal Impact Factor**	2.491	**Article Influence Score**	0.709	**% Articles in Citable Items**	93.41
**5 Year Impact Factor**	2.399	**Normalized Eigenfactor**	1.14700	**Average JIF Percentile**	45.939
**Immediacy Index**	0.385			**Cited Half-Life**	8.1
**Impact Factor Without Journal Self Cites**	2.349			**Citing Half-Life**	8.7

Source data

Journal source data 2017

	Articles	Reviews	Combined(C)	Other(O)	Percentage(C/(C+O))
**Number in JCR Year 2017 (A)**	170	12	182	14	92%
**Number of References (B)**	5,908	796	6,704	55	99%
**Ratio (B/A)**	34.8	66.3	36.8	3.9	

Downloaded on 2019/4/19 上午11:44:02 from Journal Citation Reports (©2019 Clarivate Analytics)

Box plot

Category Box Plot 2017

Category Box Plot

The category box plot depicts the distribution of Impact Factors for all journals in the category. The horizontal line that forms the top of the box is the 75th percentile (Q1). The horizontal line that forms the bottom is the 25th percentile (Q3). The horizontal line that intersects the box is the median Impact Factor for the category. Horizontal lines above and below the box, called whiskers, represent maximum and minimum values.
The top whisker is the smaller of the following two values:
the maximum Impact Factor (IF)
Q1 IF + 3.5(Q1 IF - Q3 IF)
The bottom whisker is the larger of the following two values:
the minimum Impact Factor (IF)
Q1 IF - 3.5(Q1 IF - Q3 IF)
Box Plots are provided for the current JCR year for each of the categories in which the journal is indexed.

EPILEPSY RES, IF: 2.491

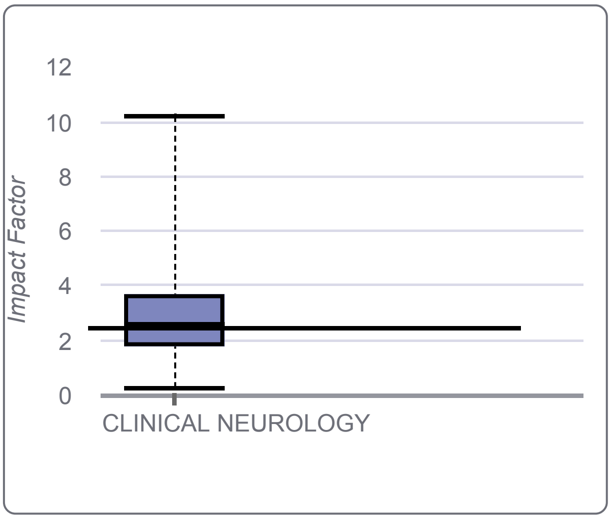

Downloaded on 2019/4/19 上午11:44:02 from Journal Citation Reports (©2019 Clarivate Analytics)

Rank

Rank 2017

JCR Impact Factor

JCR Year	CLINICAL NEUROLOGY		
	Rank	Quartile	JIF Percentile
2017	107/197	Q3	45.939
2016	102/194	Q3	47.680
2015	101/193	Q3	47.927
2014	114/192	Q3	40.885
2013	96/194	Q2	50.773
2012	91/193	Q2	53.109
2011	87/192	Q2	54.948
2010	81/185	Q2	56.486
2009	69/167	Q2	58.982
2008	65/156	Q2	58.654
2007	56/146	Q2	61.986
2006	63/147	Q2	57.483
2005	35/148	Q1	76.689
2004	29/140	Q1	79.643
2003	39/135	Q2	71.481
2002	32/138	Q1	77.174
2001	29/136	Q1	79.044
2000	23/137	Q1	83.577
1999	36/132	Q2	73.106
1998	23/125	Q1	82.000

Downloaded on 2019/4/19 上午11:44:02 from Journal Citation Reports (©2019 Clarivate Analytics)

Downloaded on 2019/4/19 上午11:44:02 from Journal Citation Reports (©2019 Clarivate Analytics)

ESI Total Citations 2017

Rank

JCR Year	NEUROSCIENCE & BEHAVIOR
2017	103/346-Q2
2016	104/345-Q2
2015	100/344-Q2
2014	100/337-Q2
2013	99/339-Q2

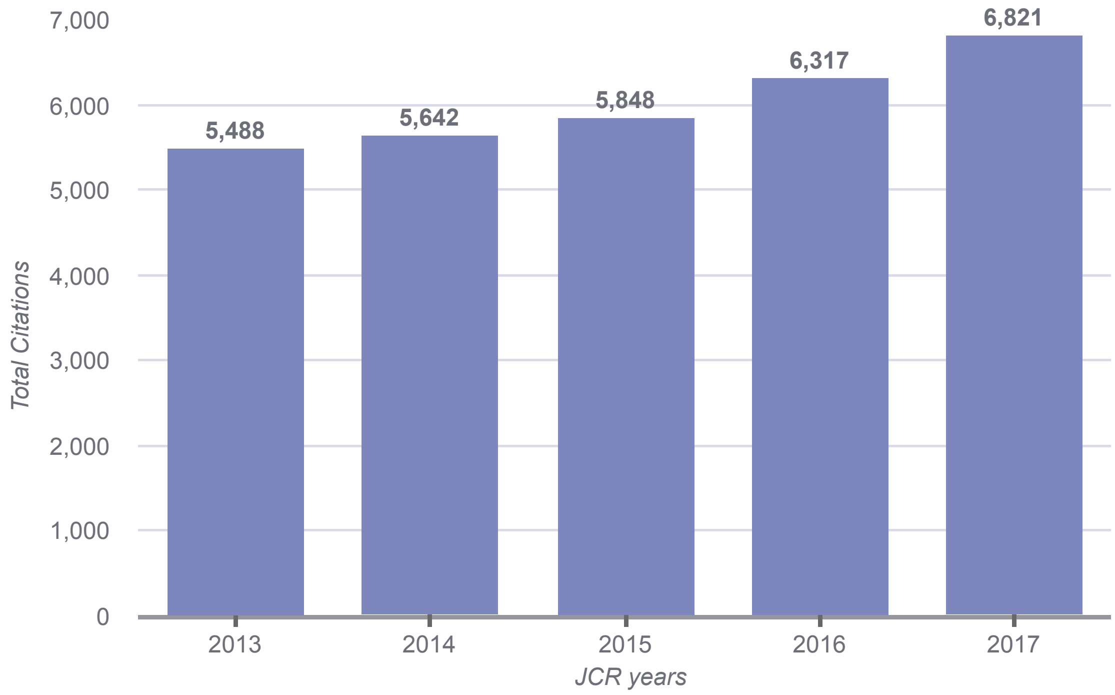

Downloaded on 2019/4/19 上午11:44:02 from Journal Citation Reports (©2019 Clarivate Analytics)

Cited Journal Data

Cited Half-Life Data

Customize columns

Cited Year	2017	2016	2015	2014	2013	2012	2011	2010	2009	2008	20
#Cites from 2017	70	385	534	558	560	376	419	457	370	304	
Cumulative %	1.03%	6.67%	14.50%	22.68%	30.89%	36.40%	42.55%	49.24%	54.67%	59.13%	10

Cited Journal Graph 2017

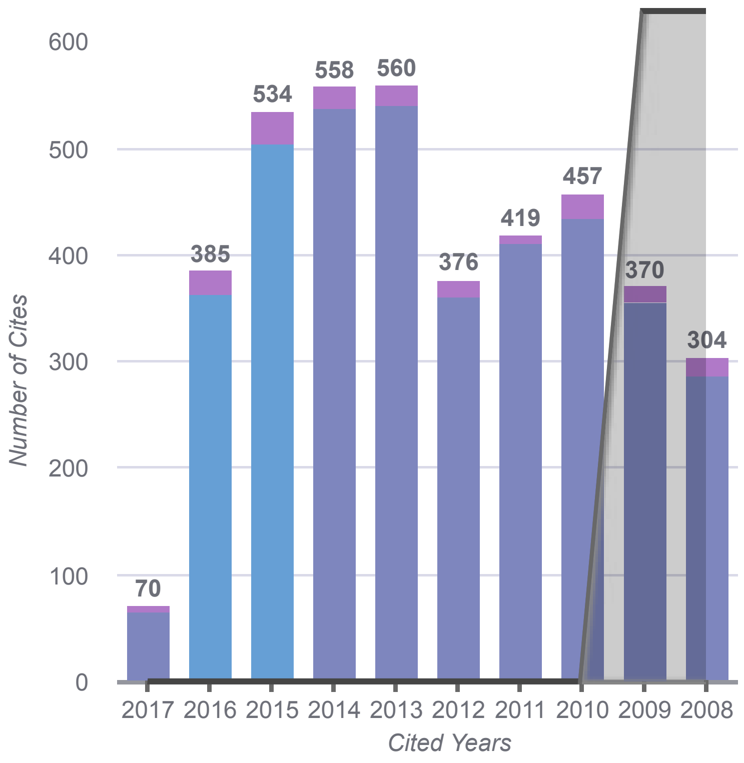

CITED JOURNAL GRAPH

The Cited Journal Graph shows the distribution (by cited year) of citations published in journals during the JCR year to items published in the Journal during the last 10 years.

The white/grey division indicates the cited half-life (if < 10.0). Half of the citations are to items that were published more recently than the cited half-life.

The two light-blue columns indicate citations used to calculate the Impact Factor (always the 2nd and 3rd columns).

Downloaded on 2019/4/19 上午11:44:02 from Journal Citation Reports (©2019 Clarivate Analytics)

Cited Journal Data

Customize columns

	Impact	Citing Journal	All Yrs	2017	2016	2015	2014	2013	2012	2011	2010	2009	2008	R
		ALL Journals	6,821	70	385	534	558	560	376	419	457	370	304	2,
		ALL OTHERS (557)	557	5	22	44	40	46	31	39	30	35	26	
1	2.600	EPILEPSY BEHAV	513	1	33	38	45	45	22	30	21	27	22	
2	5.067	EPILEPSIA	331	2	24	31	22	29	25	23	17	26	10	
3	2.839	SEIZURE-EUR J EPILEP	319	1	25	28	34	23	21	20	29	9	18	
4	2.491	EPILEPSY RES	289	6	22	30	20	19	16	8	22	15	17	
5	2.757	CURR PHARM DESIGN	154	3	9	19	17	14	4	7	9	8	4	
6	2.772	NEUROCHEM RES	122	0	9	6	10	14	4	7	10	7	3	
7	4.122	SCI REP-UK	101	2	7	7	4	7	11	4	10	0	3	
8	3.508	FRONT NEUROL	91	2	10	12	8	7	10	4	7	4	3	
9	2.766	PLOS ONE	78	0	5	4	2	8	3	3	5	3	6	
10	3.126	ACTA NEUROL SCAND	67	0	2	6	15	4	3	6	6	5	1	
11	10.848	BRAIN	59	0	3	5	3	7	5	3	8	3	12	
12	3.869	NEUROIMAGE-CLIN	57	0	3	5	12	8	1	2	4	2	6	
13	3.614	CLIN NEUROPHYSIOL	56	1	6	3	7	3	4	4	7	5	0	

***Rows 1 - 15 of 596** (use csv export to download the full table)*

Downloaded on 2019/4/19 上午11:44:02 from Journal Citation Reports (©2019 Clarivate Analytics)

Citing Journal Data

Citing Half-Life Data

Customize columns

Citing Year	2017	2016	2015	2014	2013	2012	2011	2010	2009	2008	20
#Cites from 2017	108	387	480	481	515	401	387	382	316	308	
Cumulative %	1.60%	7.32%	14.43%	21.54%	29.16%	35.09%	40.82%	46.47%	51.15%	55.70%	10

Citing Journal Graph 2017

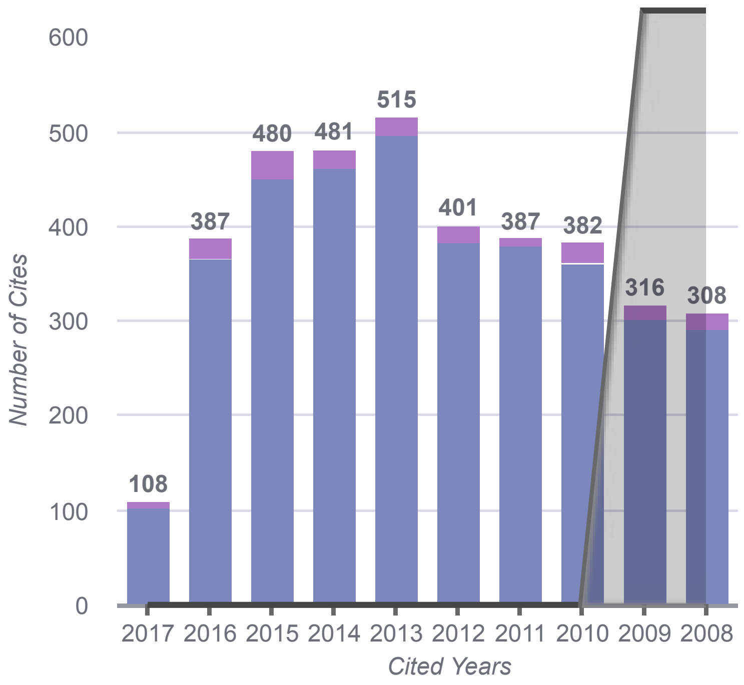

CITING JOURNAL GRAPH

The Citing Journal Graph shows the distribution (by cited year) of citations published in the Journal during the JCR year to items published in journals during the last 10 years.

The white/grey division indicates the citing half-life (if < 10.0). Half of the citations are to items that were published more recently than the citing half-life.

Downloaded on 2019/4/19 上午11:44:02 from Journal Citation Reports (©2019 Clarivate Analytics)

Citing Journal Data

Customize columns

	Impact	Cited Journal	All Yrs	2017	2016	2015	2014	2013	2012	2011	2010	2009	2008	R
		ALL Journals	6,759	108	387	480	481	515	401	387	382	316	308	2,
		ALL OTHERS (812)	812	21	55	80	76	61	48	59	37	29	26	
1	5.067	EPILEPSIA	984	13	44	44	63	77	74	69	75	52	45	
2	8.055	NEUROLOGY	301	4	8	15	10	13	18	13	15	11	22	
3	2.491	EPILEPSY RES	289	6	22	30	20	19	16	8	22	15	17	
4	2.600	EPILEPSY BEHAV	267	7	32	36	20	35	12	22	27	21	9	
5	2.839	SEIZURE-EUR J EPILEP	167	7	8	25	17	8	12	6	7	8	11	
6	10.848	BRAIN	146	1	7	5	5	2	5	6	6	1	13	
7	5.971	J NEUROSCI	128	0	4	5	5	5	3	11	8	10	8	
8	5.426	NEUROIMAGE	115	1	1	5	6	7	11	10	10	9	3	
9	10.250	ANN NEUROL	111	0	5	10	6	5	3	6	6	5	5	
10	7.144	J NEUROL NEUROSUR PS	74	0	3	3	5	1	2	1	0	1	0	
11	3.125	BRAIN RES	72	1	3	2	0	2	3	4	3	5	1	
12	27.144	LANCET NEUROL	63	1	7	2	6	7	0	7	0	3	15	
13	9.504	P NATL ACAD SCI USA	58	0	2	1	1	4	3	0	2	2	5	
14	2.766	PLOS ONE	58	1	4	3	11	23	4	10	1	1	0	

Rows 1 - 16 of 496 *(use csv export to download the full table)*

Downloaded on 2019/4/19 上午11:44:02 from Journal Citation Reports (©2019 Clarivate Analytics)

Metric trend

Metric Trend

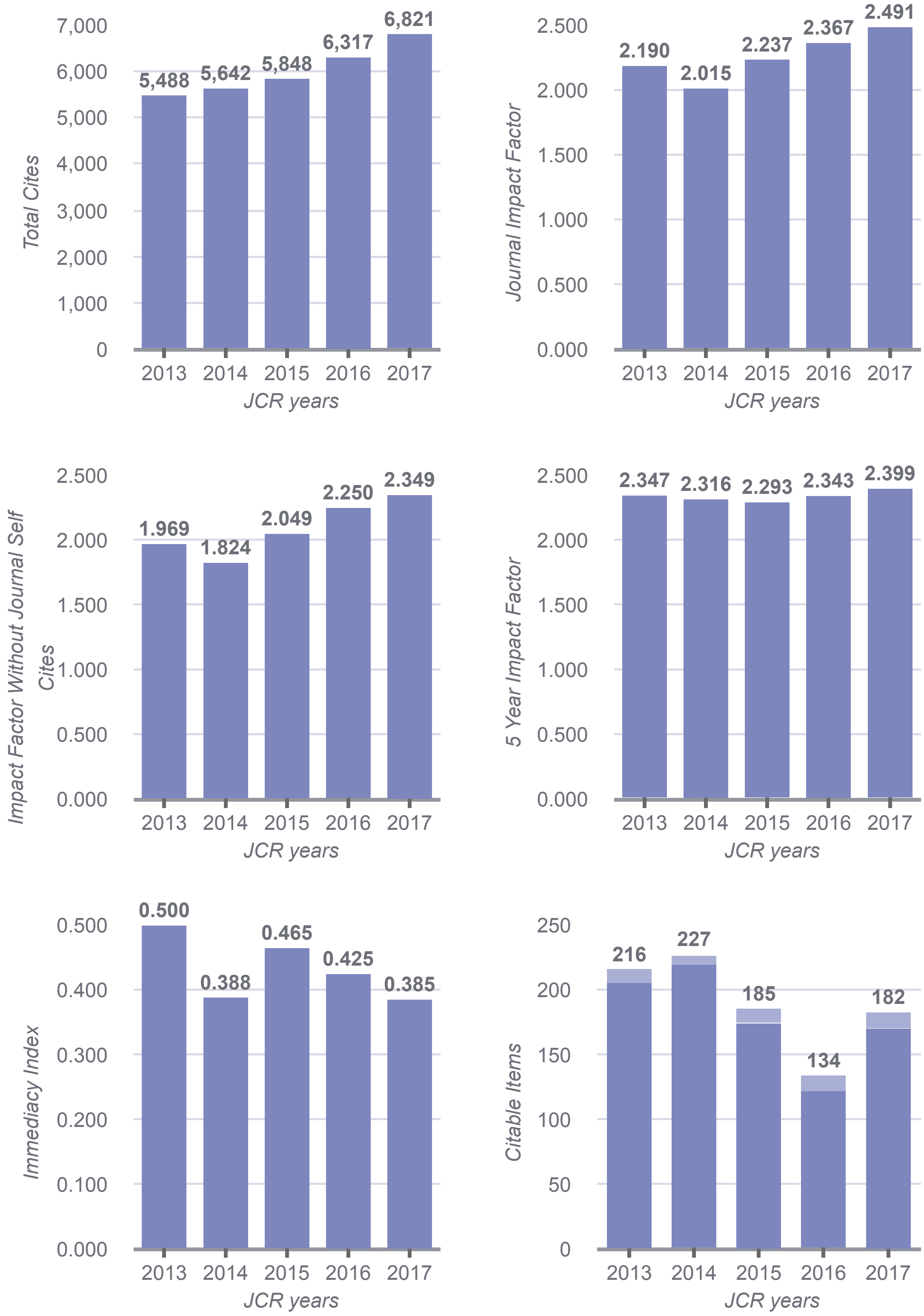

Downloaded on 2019/4/19 上午11:44:02 from Journal Citation Reports (©2019 Clarivate Analytics)

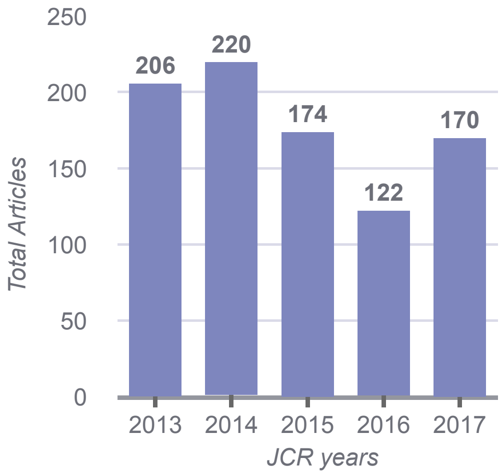

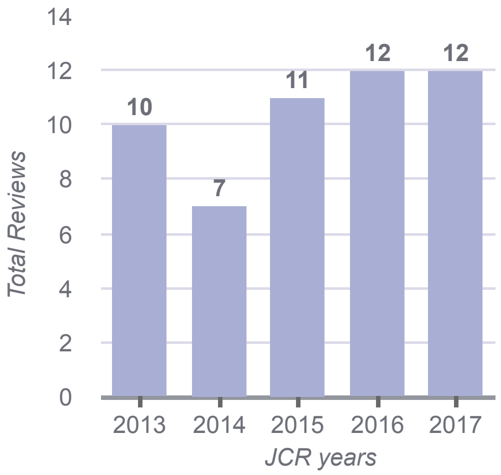

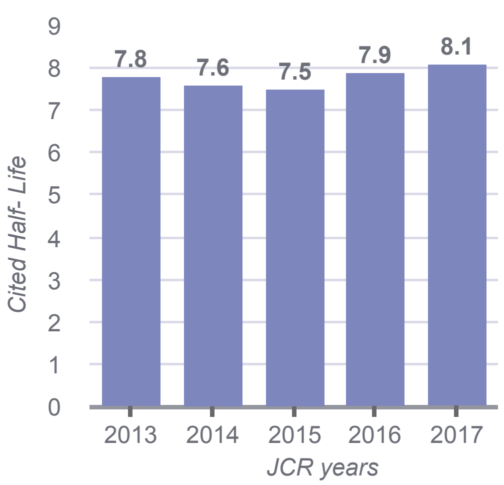

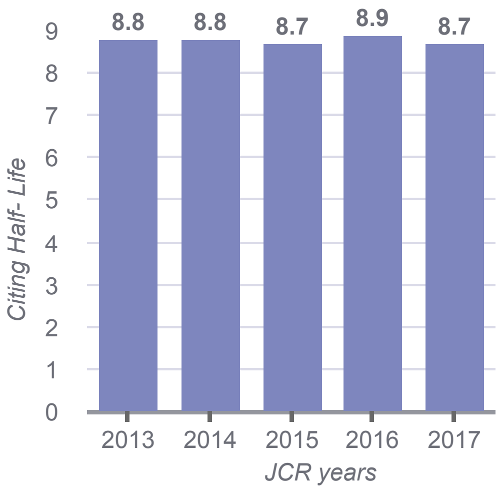

Downloaded on 2019/4/19 上午11:44:02 from Journal Citation Reports (©2019 Clarivate Analytics)

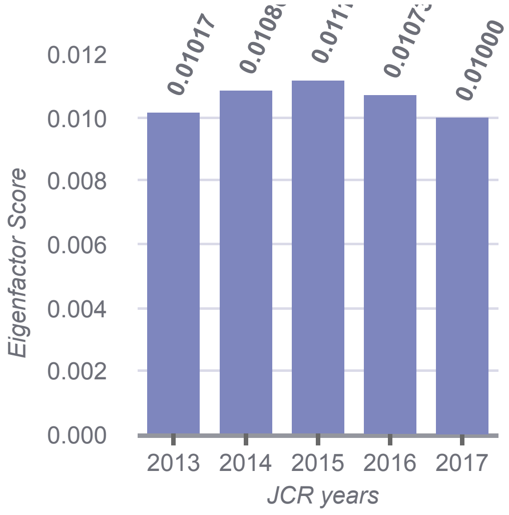

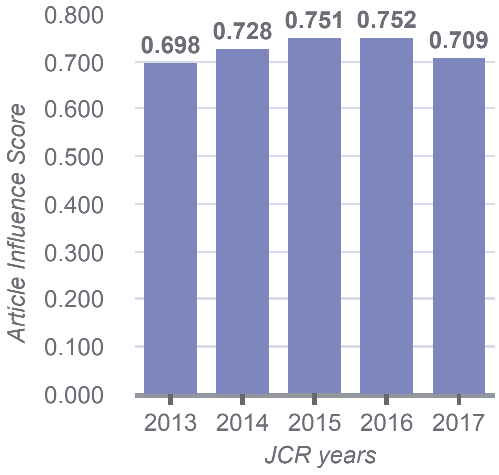

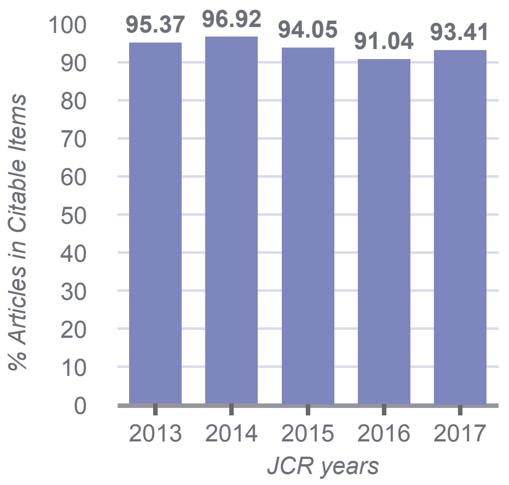

Downloaded on 2019/4/19 上午11:44:02 from Journal Citation Reports (©2019 Clarivate Analytics)

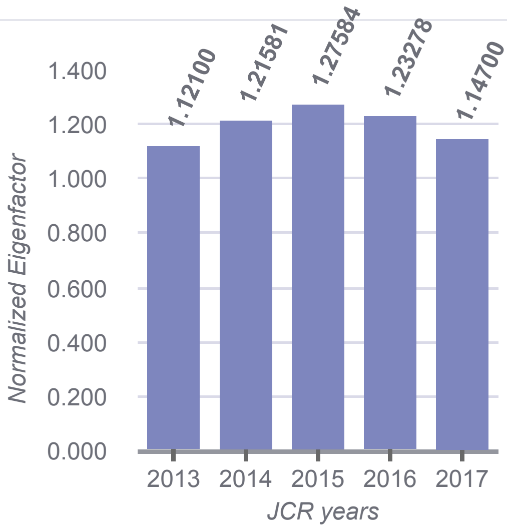

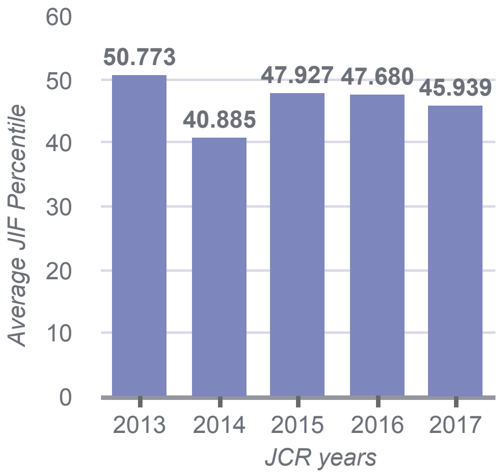

Downloaded on 2019/4/19 上午11:44:02 from Journal Citation Reports (©2019 Clarivate Analytics)

These data summarize the characteristics of the journal's published content for the most recent three years, that is, 2017 and the two prior years, combined. This information is based on all listed authors and addresses. It is meant to be descriptive rather than comparative.

Contributions by country/region

country	count
1. USA	180
2. CHINA MAINLAND	80
3. Italy	40
4. GERMANY (FED REP GER)	37
5. Japan	36
6. Canada	35
7. England	27
8. India	25
- Netherlands	25
10. Brazil	24

Contributions by organizations

organization	count
1. UNIVERSITY OF CALIFORNIA SYSTEM	24
2. UNIVERSITY OF MELBOURNE	20
3. HARVARD UNIVERSITY	19
4. VA BOSTON HEALTHCARE SYSTEM	16
- CAPITAL MEDICAL UNIVERSITY	16
6. UNIVERSITY OF LONDON	15
- FLOREY INSTITUTE OF NEUROSCIENCE & MENTAL HEALTH	15
8. SICHUAN UNIVERSITY	13
9. UCB PHARMA SA	12
- UNIVERSITY OF PENNSYLVANIA	12

Downloaded on 2019/4/19 上午11:44:02 from Journal Citation Reports (©2019 Clarivate Analytics)
