# Supplementary material for: Emerging trends and knowledge structure of epilepsy during pregnancy research for 2000–2018: a bibliometric analysis
Source: PeerJ. 2019 Jun 7;7:e7115. doi: 10.7717/peerj.7115 (PMC6557303; doi:10.7717/peerj.7115)
Supplement: Supplemental Information 4 [file peerj-07-7115-s004.zip › 7/11. InCites Journal Citation Reports(PEDIATRIC NEUROLOGY).pdf]

2017 Journal Performance Data for: PEDIATRIC NEUROLOGY

ISSN: 0887-8994
eISSN: 1873-5150
ELSEVIER SCIENCE INC
360 PARK AVE SOUTH, NEW YORK, NY 10010-1710
USA

TITLES
ISO: Pediatr. Neurol.
JCR Abbrev: PEDIATR NEUROL

LANGUAGES
English

CATEGORIES
CLINICAL NEUROLOGY - SCIE

PEDIATRICS - SCIE

PUBLICATION FREQUENCY
12 issues/year

Downloaded on 2019/4/19 上午11:45:37 from Journal Citation Reports (©2019 Clarivate Analytics)

Current Year

The data in the two graphs below and in the Journal Impact Factor calculation panels represent citation activity in 2017 to items published in the journal in the prior two years. They detail the components of the Journal Impact Factor. Use the "All Years" tab to access key metrics and additional data for the current year and all prior years for this journal.

2017 Journal Impact Factor & percentile rank in category for: PEDIATRIC NEUROLOGY

2.398

2017 Journal Impact Factor

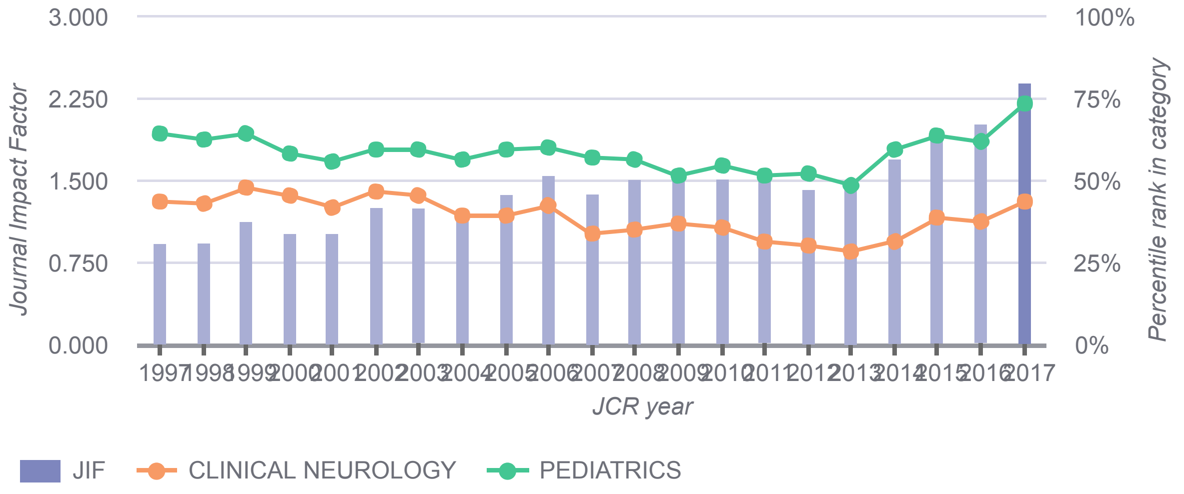

2017 JIF Citation Distribution for: PEDIATRIC NEUROLOGY

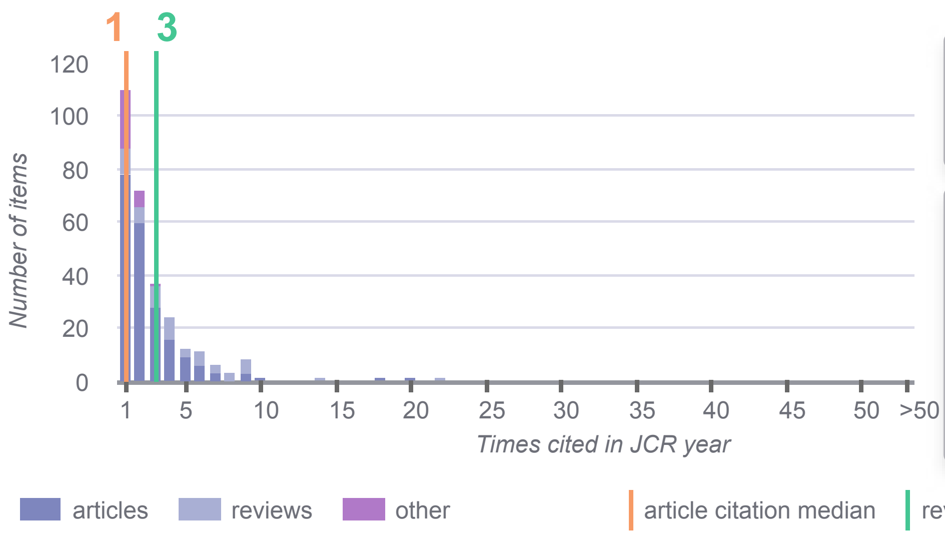

28

Unlinked citations

0

Times Cited

- **82** articles
- **8** reviews
- **91** other

Downloaded on 2019/4/19 上午11:45:37 from Journal Citation Reports (©2019 Clarivate Analytics)

Journal Impact Factor Calculation

2017 Journal Impact Factor $= \frac{837}{349} = 2.398$

How is Journal Impact Factor Calculated?

$$\text{JIF} = \frac{\text{Citations in 2017 to items published in } \mathbf{2015\ (500) + 2016\ (337)}}{\text{Number of citable items in } \mathbf{2015\ (177) + 2016\ (172)}} = \frac{837}{349}$$

Downloaded on 2019/4/19 上午11:45:37 from Journal Citation Reports (©2019 Clarivate Analytics)

Journal Impact Factor contributing items

Citable items in 2016 and 2015 (349)

TITLE	CITATIONS COUNTED TOWARDS JIF
Neurodevelopmental Outcome After Extreme Prematurity: A Review of the Literature<br>By: Jarjour, Imad T.<br>**Volume: 52 Page: 143-152 Accession number: WOS:000349580600002**<br>**Document Type: Review**	22
Tuberous Sclerosis Associated Neuropsychiatric Disorders (TAND) and the TAND Checklist<br>By: de Vries, Petrus J.; Jansen, Anna; Whittemore, Vicky H.; Leclezio, Loren; Byars, Anna W.; et al.<br>**Volume: 52 Page: 25-35 Accession number: WOS:000348411800004**<br>**Document Type: Article**	20
The Expanding Spectrum of Neurological Phenotypes in Children With ATP1A3 Mutations, Alternating Hemiplegia of Childhood, Rapid-onset Dystonia-Parkinsonism, CAPOS and Beyond<br>By: Sweney, Matthew T.; Newcomb, Tara M.; Swoboda, Kathryn J.<br>**Volume: 52 Page: 56-64 Accession number: WOS:000348411800008**<br>**Document Type: Article**	18
Mechanistic Target of Rapamycin (mTOR) in Tuberous Sclerosis Complex-Associated Epilepsy<br>By: Curatolo, Paolo<br>**Volume: 52 Page: 281-289 Accession number: WOS:000351022000004**<br>**Document Type: Review**	14
Pilot Study of Intensive Chemotherapy With Peripheral Hematopoietic Cell Support for Children Less Than 3 Years of Age With Malignant Brain Tumors, the CCG-99703 Phase I/II Study. A Report From the Children's Oncology Group<br>By: Cohen, Bruce H.; Packer, Roger J.; Pollack, Ian F.; Gajjar, Amar; Finlay, Jonathan L.; et al.<br>**Volume: 53 Page: 31-46 Accession number: WOS:000368465500005**<br>**Document Type: Article**	10
Recognition and Management of Acute Flaccid Myelitis in Children<br>By: Nelson, Gary R.; Bonkowsky, Joshua L.; Doll, Elizabeth; Green, Michael; Hedlund, Gary L.; et al.<br>**Volume: 55 Page: 17-21 Accession number: WOS:000370585200005**<br>**Document Type: Article**	9
Clinical Course of Six Children With GNAO1 Mutations Causing a Severe and Distinctive Movement Disorder<br>By: Ananth, Arnitha L.; Wu, Yvonne W.; Bernstein, Jonathan A.; Robichaux-Viehoever, Amy; Kim, Young-Min; et al.<br>**Volume: 59 Page: 81-84 Accession number: WOS:000379452200016**<br>**Document Type: Article**	9

Downloaded on 2019/4/19 上午11:45:37 from Journal Citation Reports (©2019 Clarivate Analytics)

Citations in 2017 (837)

TITLE	CITATIONS COUNTED TOWARDS JIF
PEDIATRIC NEUROLOGY	60
SEMINARS IN PEDIATRIC NEUROLOGY	18
NEUROLOGY	14
SCIENTIFIC REPORTS	13
JOURNAL OF CHILD NEUROLOGY	11
JOURNAL OF PEDIATRICS	11
PEDIATRICS	11
DEVELOPMENTAL MEDICINE AND CHILD NEUROLOGY	10
EPILEPSIA	10
FRONTIERS IN NEUROLOGY	10

Downloaded on 2019/4/19 上午11:45:37 from Journal Citation Reports (©2019 Clarivate Analytics)

Key Indicators 2017

IMPACT METRICS		INFLUENCE METRICS		SOURCE METRICS	
Total Cites	5,337	Eigenfactor Score	0.01000	Citable Items	165
Journal Impact Factor	2.398	Article Influence Score	0.730	% Articles in Citable Items	82.42
5 Year Impact Factor	2.295	Normalized Eigenfactor	1.11400	Average JIF Percentile	58.596
Immediacy Index	0.521			Cited Half-Life	7.9
Impact Factor Without Journal Self Cites	2.226			Citing Half-Life	8.4

Source data

Journal source data 2017

	Articles	Reviews	Combined(C)	Other(O)	Percentage(C/(C+O))
Number in JCR Year 2017 (A)	136	29	165	58	73%
Number of References (B)	3,465	1,849	5,314	271	95%
Ratio (B/A)	25.5	63.8	32.2	4.7	

Downloaded on 2019/4/19 上午11:45:37 from Journal Citation Reports (©2019 Clarivate Analytics)

Box plot

Category Box Plot 2017

Category Box Plot

The category box plot depicts the distribution of Impact Factors for all journals in the category. The horizontal line that forms the top of the box is the 75th percentile (Q1). The horizontal line that forms the bottom is the 25th percentile (Q3). The horizontal line that intersects the box is the median Impact Factor for the category. Horizontal lines above and below the box, called whiskers, represent maximum and minimum values.
The top whisker is the smaller of the following two values:
the maximum Impact Factor (IF)
Q1 IF + 3.5(Q1 IF - Q3 IF)
The bottom whisker is the larger of the following two values:
the minimum Impact Factor (IF)
Q1 IF - 3.5(Q1 IF - Q3 IF)
Box Plots are provided for the current JCR year for each of the categories in which the journal is indexed.

PEDIATR NEUROL, IF: 2.398

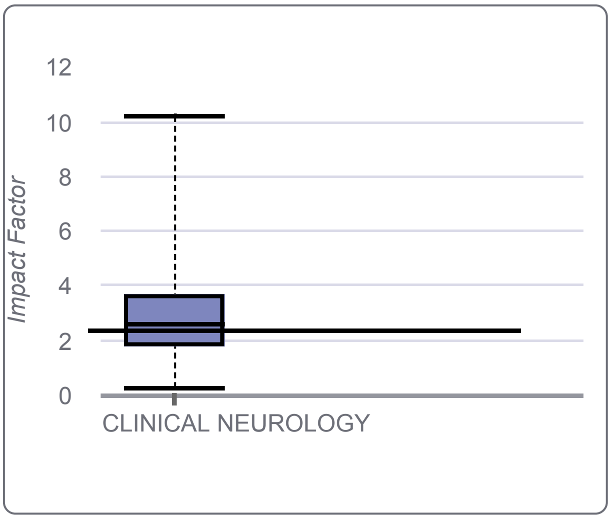

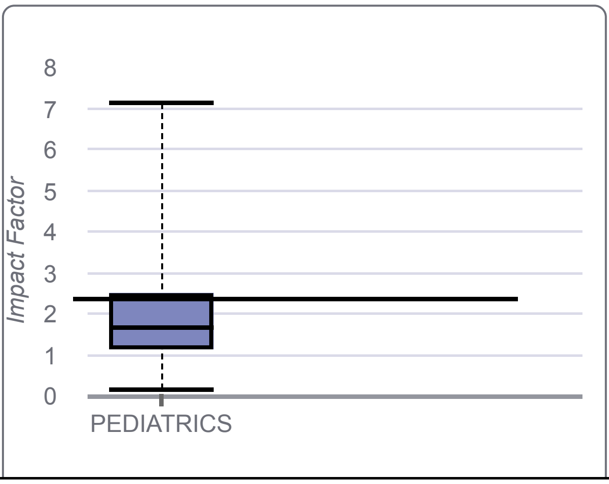

Downloaded on 2019/4/19 上午11:45:37 from Journal Citation Reports (©2019 Clarivate Analytics)

Rank

Rank 2017

JCR Impact Factor

JCR Year	CLINICAL NEUROLOGY			PEDIATRICS		
	Rank	Quartile	JIF Percentile	Rank	Quartile	JIF Percentile
2017	112/197	Q3	43.401	33/124	Q2	73.790
2016	122/194	Q3	37.371	46/121	Q2	62.397
2015	119/193	Q3	38.601	44/120	Q2	63.750
2014	132/192	Q3	31.510	49/120	Q2	59.583
2013	140/194	Q3	28.093	61/118	Q3	48.729
2012	135/193	Q3	30.311	59/122	Q2	52.049
2011	132/192	Q3	31.510	56/115	Q2	51.739
2010	119/185	Q3	35.946	50/109	Q2	54.587
2009	106/167	Q3	36.826	46/94	Q2	51.596
2008	102/156	Q3	34.936	38/86	Q2	56.395
2007	97/146	Q3	33.904	34/78	Q2	57.051
2006	85/147	Q3	42.517	30/74	Q2	60.135
2005	90/148	Q3	39.527	30/73	Q2	59.589
2004	85/140	Q3	39.643	31/70	Q2	56.429
2003	74/135	Q3	45.556	28/68	Q2	59.559
2002	74/138	Q3	46.739	28/68	Q2	59.559
2001	80/136	Q3	41.544	31/69	Q2	55.797
2000	75/137	Q3	45.620	30/71	Q2	58.451
1999	69/132	Q3	48.106	26/72	Q2	64.583
1998	72/125	Q3	42.800	27/71	Q2	62.676

Downloaded on 2019/4/19 上午11:45:37 from Journal Citation Reports (©2019 Clarivate Analytics)

Downloaded on 2019/4/19 上午11:45:37 from Journal Citation Reports (©2019 Clarivate Analytics)

ESI Total Citations 2017

Rank

JCR Year	NEUROSCIENCE & BEHAVIOR
2017	129/346-Q2
2016	130/345-Q2
2015	131/344-Q2
2014	138/337-Q2
2013	129/339-Q2

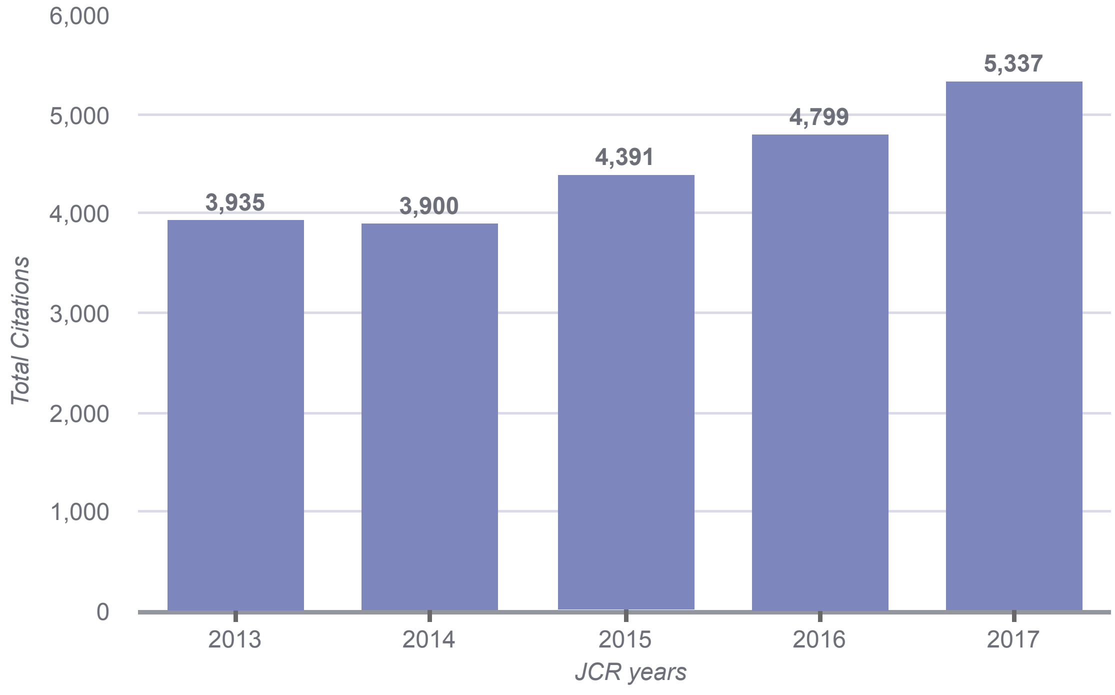

Downloaded on 2019/4/19 上午11:45:37 from Journal Citation Reports (©2019 Clarivate Analytics)

Cited Journal Data

Cited Half-Life Data

Customize columns

Cited Year	2017	2016	2015	2014	2013	2012	2011	2010	2009	2008	20
#Cites from 2017	86	337	500	510	505	326	212	223	242	232	
Cumulative %	1.61%	7.93%	17.29%	26.85%	36.31%	42.42%	46.39%	50.57%	55.11%	59.45%	10

Cited Journal Graph 2017

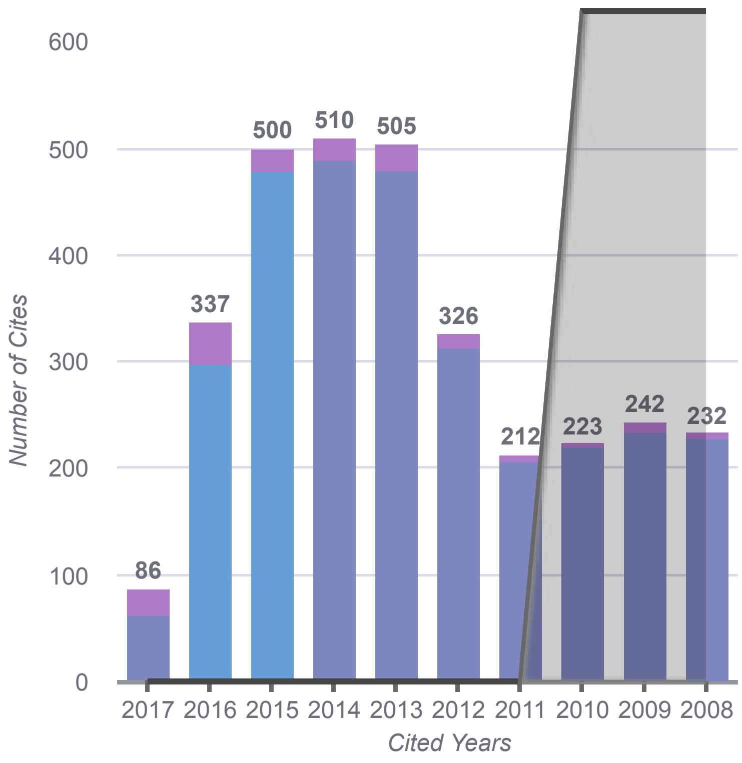

CITED JOURNAL GRAPH

The Cited Journal Graph shows the distribution (by cited year) of citations published in journals during the JCR year to items published in the Journal during the last 10 years.

The white/grey division indicates the cited half-life (if < 10.0). Half of the citations are to items that were published more recently than the cited half-life.

The two light-blue columns indicate citations used to calculate the Impact Factor (always the 2nd and 3rd columns).

Downloaded on 2019/4/19 上午11:45:37 from Journal Citation Reports (©2019 Clarivate Analytics)

Cited Journal Data

Customize columns

	Impact	Citing Journal	All Yrs	2017	2016	2015	2014	2013	2012	2011	2010	2009	2008	R
		ALL Journals	5,337	86	337	500	510	505	326	212	223	242	232	2,
		ALL OTHERS (706)	706	9	38	63	71	48	37	32	31	30	31	
1	2.398	PEDIATR NEUROL	210	25	39	21	20	24	13	6	3	9	5	
2	1.878	SEMIN PEDIATR NEUROL	90	4	7	11	8	6	8	6	5	0	1	
3	1.665	J CHILD NEUROL	86	1	3	8	9	7	5	5	3	5	5	
4	4.122	SCI REP-UK	73	2	4	9	10	3	4	4	2	6	4	
5	2.839	SEIZURE-EUR J EPILEP	70	1	5	3	5	7	3	5	5	1	2	
6	5.067	EPILEPSIA	60	2	3	7	8	3	9	1	0	0	0	
7	2.766	PLOS ONE	60	2	2	6	4	5	5	3	1	2	3	
8	2.600	EPILEPSY BEHAV	55	3	4	5	2	5	4	4	4	1	4	
9	3.289	DEV MED CHILD NEUROL	54	0	4	6	4	3	4	1	2	4	4	
10	2.362	EUR J PAEDIATR NEURO	52	0	1	1	4	3	5	6	4	6	3	
11	1.235	CHILD NERV SYST	49	0	2	5	5	4	1	3	2	2	5	
12	1.544	BRAIN DEV-JPN	48	0	5	3	4	4	4	0	3	5	0	

Rows 1 - 14 of 679 *(use csv export to download the full table)*

Downloaded on 2019/4/19 上午11:45:37 from Journal Citation Reports (©2019 Clarivate Analytics)

Citing Journal Data

Citing Half-Life Data

Customize columns

Citing Year	2017	2016	2015	2014	2013	2012	2011	2010	2009	2008	20
#Cites from 2017	89	361	401	403	408	357	337	300	277	258	
Cumulative %	1.59%	8.06%	15.24%	22.45%	29.76%	36.15%	42.18%	47.56%	52.52%	57.14%	10

Citing Journal Graph 2017

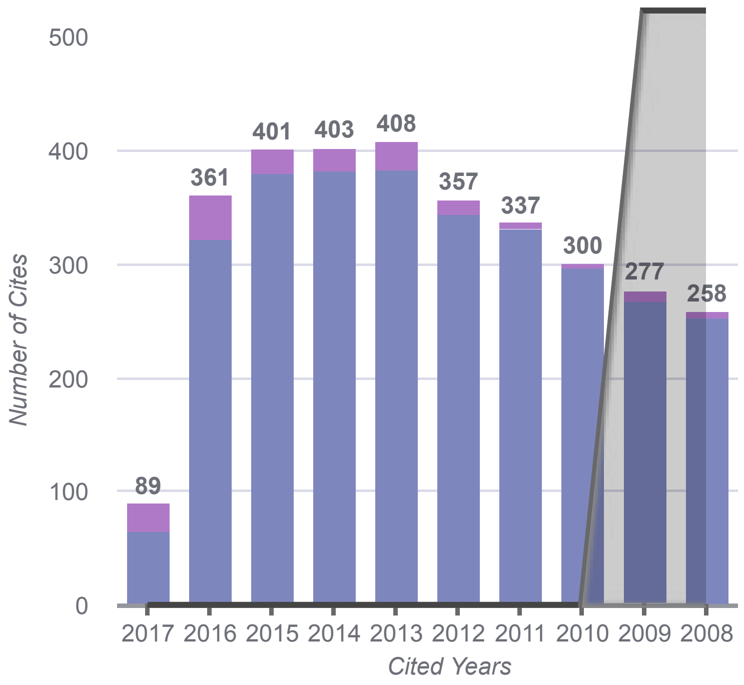

CITING JOURNAL GRAPH

The Citing Journal Graph shows the distribution (by cited year) of citations published in the Journal during the JCR year to items published in journals during the last 10 years.

The white/grey division indicates the citing half-life (if < 10.0). Half of the citations are to items that were published more recently than the citing half-life.

Downloaded on 2019/4/19 上午11:45:37 from Journal Citation Reports (©2019 Clarivate Analytics)

Citing Journal Data

Customize columns

	Impact	Cited Journal	All Yrs	2017	2016	2015	2014	2013	2012	2011	2010	2009	2008	R
		ALL Journals	5,585	89	361	401	403	408	357	337	300	277	258	2,
		ALL OTHERS (822)	822	11	54	72	62	55	42	52	40	32	30	
1	8.055	NEUROLOGY	311	2	18	17	23	22	20	18	10	10	22	
2	2.398	PEDIATR NEUROL	210	25	39	21	20	24	13	6	3	9	5	
3	5.067	EPILEPSIA	194	2	9	12	14	17	13	22	14	8	14	
4	5.515	PEDIATRICS	160	0	8	10	6	6	8	15	9	12	13	
5	6.239	STROKE	160	0	15	7	9	3	14	12	11	19	8	
6	1.665	J CHILD NEUROL	143	4	8	10	12	10	12	9	4	9	10	
7	3.289	DEV MED CHILD NEUROL	104	2	4	10	7	11	6	5	7	1	5	
8	10.250	ANN NEUROL	98	1	5	1	4	4	6	8	11	7	4	
9	3.667	J PEDIATR-US	83	3	4	4	4	6	4	4	5	5	4	
10	79.260	NEW ENGL J MED	74	0	0	8	3	5	6	3	5	2	0	
11	3.653	AM J NEURORADIOL	73	0	5	5	4	4	1	5	1	6	8	
12	2.600	EPILEPSY BEHAV	68	2	5	6	8	9	3	9	8	2	4	
13	1.544	BRAIN DEV-JPN	65	0	4	3	1	6	8	4	3	4	2	

Rows 1 - 15 of 497 *(use csv export to download the full table)*

Downloaded on 2019/4/19 上午11:45:37 from Journal Citation Reports (©2019 Clarivate Analytics)

Metric trend

Metric Trend

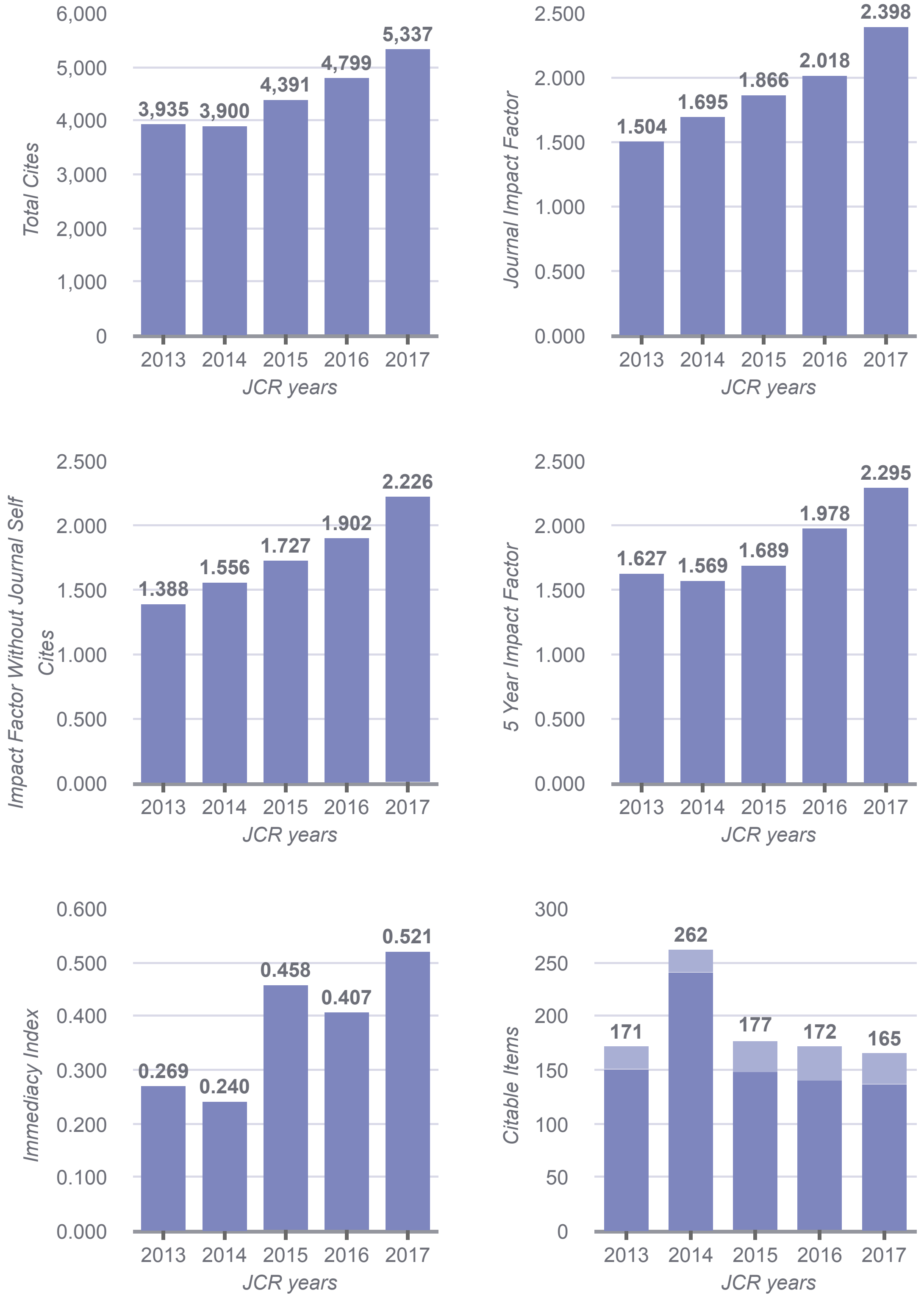

Downloaded on 2019/4/19 上午11:45:37 from Journal Citation Reports (©2019 Clarivate Analytics)

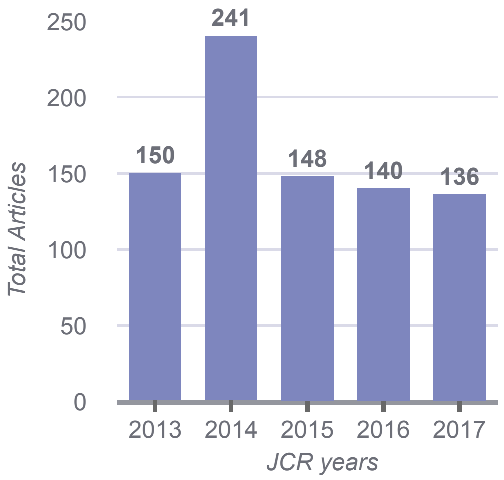

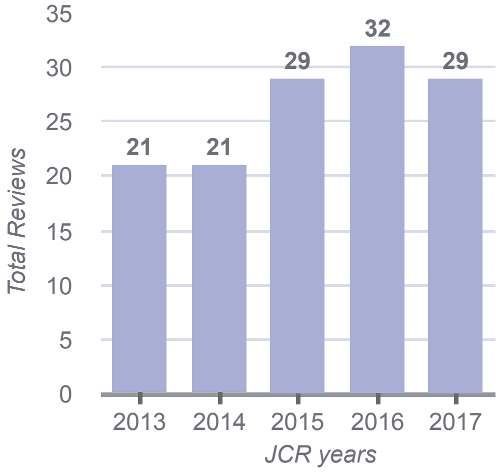

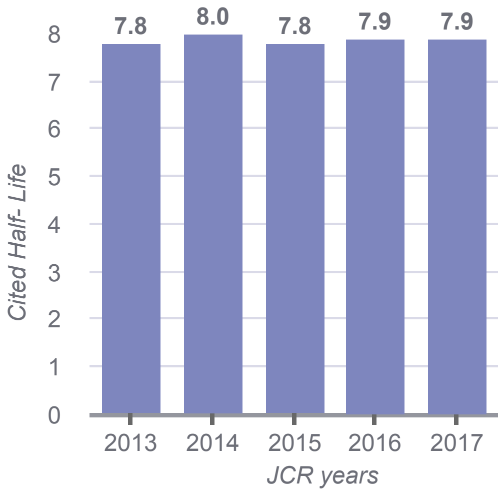

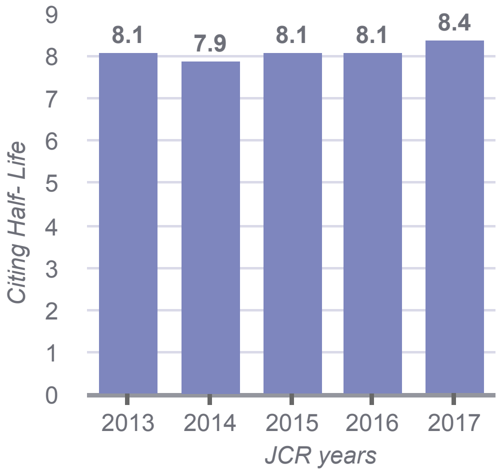

Downloaded on 2019/4/19 上午11:45:37 from Journal Citation Reports (©2019 Clarivate Analytics)

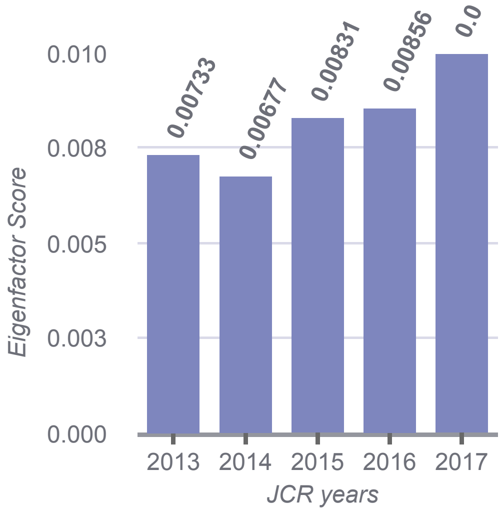

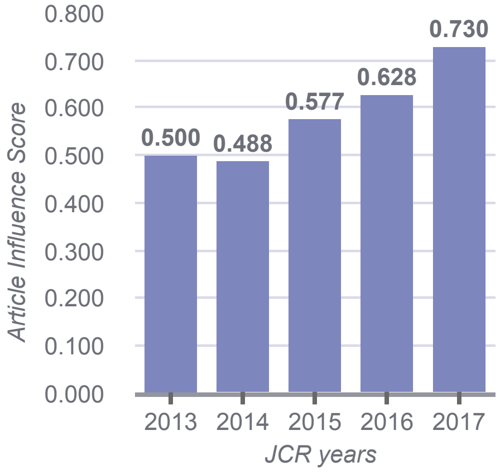

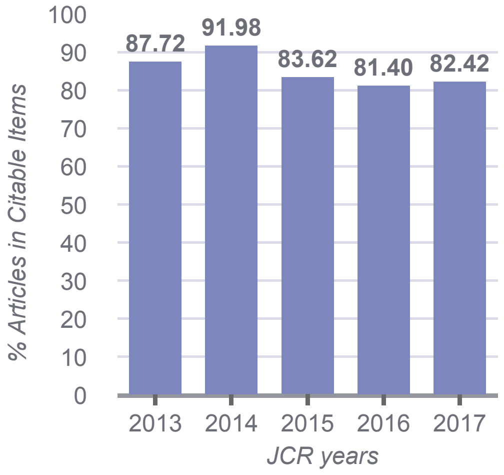

Downloaded on 2019/4/19 上午11:45:37 from Journal Citation Reports (©2019 Clarivate Analytics)

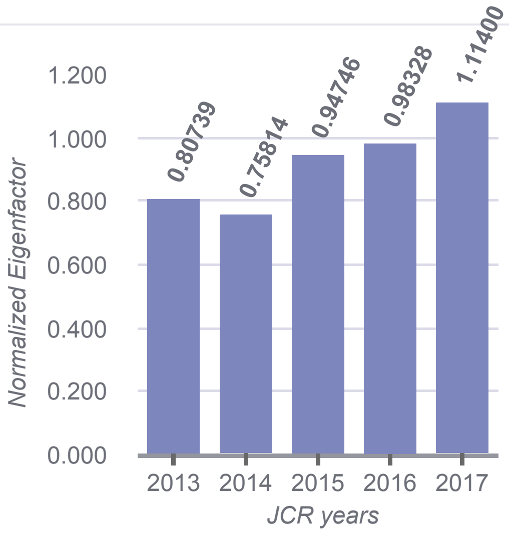

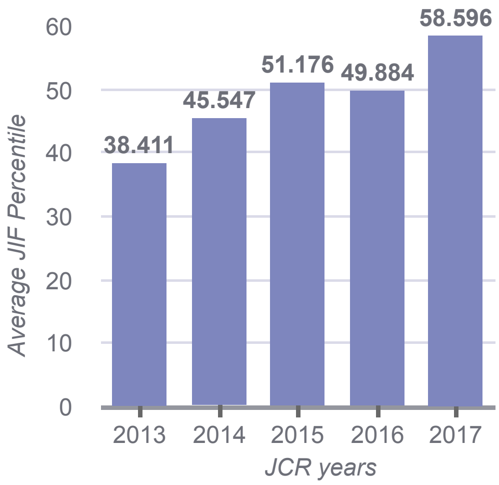

Downloaded on 2019/4/19 上午11:45:37 from Journal Citation Reports (©2019 Clarivate Analytics)

These data summarize the characteristics of the journal's published content for the most recent three years, that is, 2017 and the two prior years, combined. This information is based on all listed authors and addresses. It is meant to be descriptive rather than comparative.

Contributions by country/region

country	count
1. USA	421
2. Canada	63
3. India	32
- Japan	32
5. Italy	22
6. CHINA MAINLAND	20
7. Turkey	16
8. South Korea	15
9. Australia	14
10. England	13
- GERMANY (FED REP GER)	13

Contributions by organizations

organization	count
1. OHIO STATE UNIVERSITY	52
2. HARVARD UNIVERSITY	51
3. VA BOSTON HEALTHCARE SYSTEM	50
4. BOSTON CHILDREN'S HOSPITAL	47
5. UNIVERSITY OF CALIFORNIA SYSTEM	37
6. UNIVERSITY OF TORONTO	28
7. HOSPITAL FOR SICK CHILDREN (SICKKIDS)	27
8. JOHNS HOPKINS UNIVERSITY	26
9. UNIVERSITY OF PENNSYLVANIA	22
10. BAYLOR COLLEGE OF MEDICINE	21

Downloaded on 2019/4/19 上午11:45:37 from Journal Citation Reports (©2019 Clarivate Analytics)
