# Supplementary material for: Emerging trends and knowledge structure of epilepsy during pregnancy research for 2000–2018: a bibliometric analysis
Source: PeerJ. 2019 Jun 7;7:e7115. doi: 10.7717/peerj.7115 (PMC6557303; doi:10.7717/peerj.7115)
Supplement: Supplemental Information 4 [file peerj-07-7115-s004.zip › 7/12. InCites Journal Citation Reports(EUROPEAN JOURNAL OF PAEDIATRIC NEUROLOGY).pdf]

2017 Journal Performance Data for: EUROPEAN JOURNAL OF PAEDIATRIC NEUROLOGY

ISSN: 1090-3798
eISSN: 1532-2130
ELSEVIER SCI LTD
THE BOULEVARD, LANGFORD LANE, KIDLINGTON, OXFORD OX5 1GB, OXON, ENGLAND
ENGLAND

TITLES
ISO: Eur. J. Paediatr. Neurol.
JCR Abbrev: EUR J PAEDIATR NEURO

LANGUAGES
English

CATEGORIES
CLINICAL NEUROLOGY - SCIE

PEDIATRICS - SCIE

PUBLICATION FREQUENCY
6 issues/year

Downloaded on 2019/4/19 上午11:47:17 from Journal Citation Reports (©2019 Clarivate Analytics)

Current Year

The data in the two graphs below and in the Journal Impact Factor calculation panels represent citation activity in 2017 to items published in the journal in the prior two years. They detail the components of the Journal Impact Factor. Use the "All Years" tab to access key metrics and additional data for the current year and all prior years for this journal.

2017 Journal Impact Factor & percentile rank in category for: EUROPEAN JOURNAL OF PAEDIATRIC NEUROLOGY

2.362
2017 Journal Impact Factor

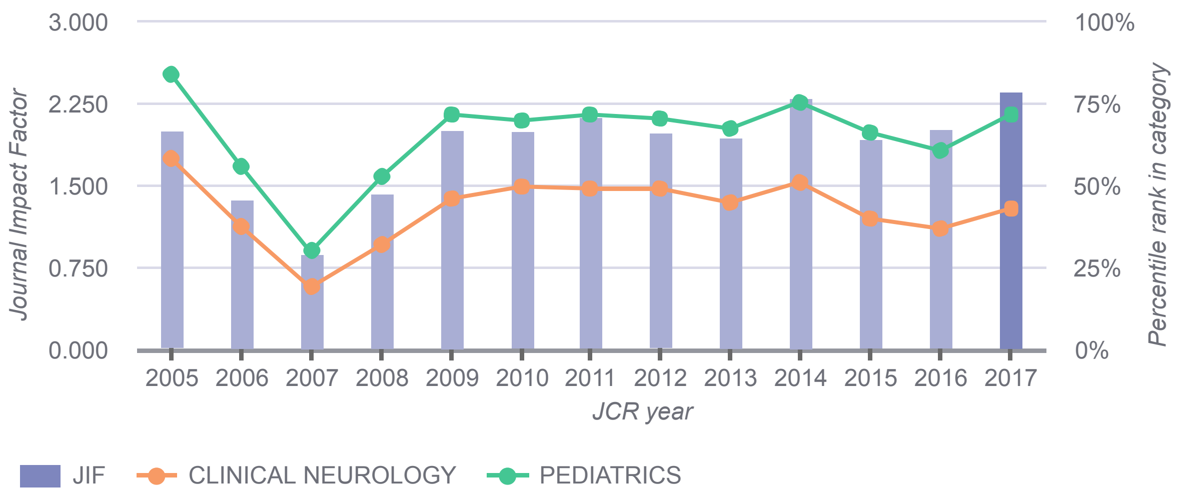

2017 JIF Citation Distribution for: EUROPEAN JOURNAL OF PAEDIATRIC NEUROLOGY

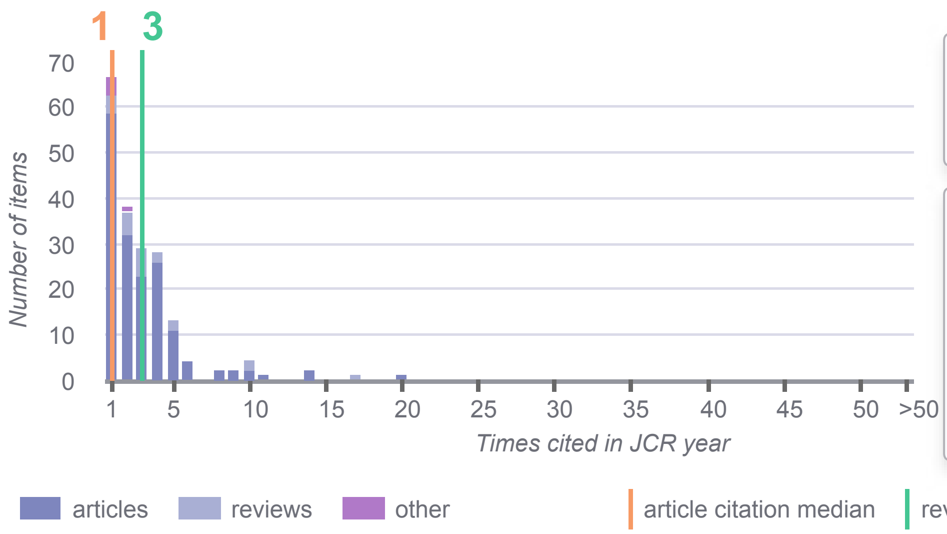

26
Unlinked citations

0
Times Cited

- **67** articles
- **3** reviews
- **21** other

Downloaded on 2019/4/19 上午11:47:17 from Journal Citation Reports (©2019 Clarivate Analytics)

Journal Impact Factor Calculation

$$\text{2017 Journal Impact Factor} = \frac{607}{257} = 2.362$$

How is Journal Impact Factor Calculated?

$$\text{JIF} = \frac{\text{Citations in 2017 to items published in } \mathbf{2015\ (290) + 2016\ (317)}}{\text{Number of citable items in } \mathbf{2015\ (109) + 2016\ (148)}} = \frac{607}{257}$$

Downloaded on 2019/4/19 上午11:47:17 from Journal Citation Reports (©2019 Clarivate Analytics)

Journal Impact Factor contributing items

Citable items in 2016 and 2015 (257)

TITLE	CITATIONS COUNTED TOWARDS JIF
Refractory absence seizures: An Italian multicenter retrospective study<br>By: Franzoni, Emilio; Accorsi, Patrizia; Giordano, Lucio; Coppola, Giangennaro; Cerminara, Caterina; et al.<br>**Volume: 19 Page: 660-664 Accession number: WOS:000363346400007**<br>**Document Type: Article**	20
Current role of melatonin in pediatric neurology: Clinical recommendations<br>By: Bruni, Oliviero; Van der Heijden, Kristiaan; Curatolo, Paolo; Alonso-Alconada, Daniel; Besag, Frank; et al.<br>**Volume: 19 Page: 122-133 Accession number: WOS:000350515100003**<br>**Document Type: Review**	17
Medication use in childhood dystonia<br>By: Lumsden, Daniel E.; Kaminska, Margaret; Tomlin, Stephen; Lin, Jean-Pierre<br>**Volume: 20 Page: 625-629 Accession number: WOS:000379106700020**<br>**Document Type: Article**	14
Interventional studies in childhood dystonia do not address the concerns of children and their carers<br>By: Lumsden, Daniel E.; Gimeno, Hortensia; Tustin, Kylee; Kaminska, Margaret; Lin, Jean-Pierre<br>**Volume: 19 Page: 327-336 Accession number: WOS:000353612000011**<br>**Document Type: Article**	14
Gabapentin can significantly improve dystonia severity and quality of life in children<br>By: Liow, Natasha Yuan-Kim; Gimeno, Hortensia; Lumsden, Daniel Edward; Marianczak, Jennifer; Kaminska, Margaret; et al.<br>**Volume: 20 Page: 100-107 Accession number: WOS:000368569100014**<br>**Document Type: Article**	11
Progression to musculoskeletal deformity in childhood dystonia<br>By: Lumsden, Daniel E.; Gimeno, Hortensia; Elze, Markus; Tustin, Kylee; Kaminska, Margaret; et al.<br>**Volume: 20 Page: 339-345 Accession number: WOS:000374799200001**<br>**Document Type: Article**	10
Ketogenic diet guidelines for infants with refractory epilepsy<br>By: van der Louw, Elles; Dressler, Anastasia; Klepper, Joerg; Auvin, Stephane; Cross, J. Helen; et al.<br>**Volume: 20 Page: 798-809 Accession number: WOS:000386322300002**<br>**Document Type: Review**	10

Downloaded on 2019/4/19 上午11:47:17 from Journal Citation Reports (©2019 Clarivate Analytics)

Citations in 2017 (607)

TITLE	CITATIONS COUNTED TOWARDS JIF
EUROPEAN JOURNAL OF PAEDIATRIC NEUROLOGY	50
ACTA MEDICA MEDITERRANEA	18
JOURNAL OF CHILD NEUROLOGY	16
EPILEPSY & BEHAVIOR	15
DEVELOPMENTAL MEDICINE AND CHILD NEUROLOGY	13
SEIZURE-EUROPEAN JOURNAL OF EPILEPSY	13
EPILEPSIA	12
PEDIATRIC NEUROLOGY	11
CURRENT OPINION IN NEUROLOGY	8
SEMINARS IN PEDIATRIC NEUROLOGY	8

Downloaded on 2019/4/19 上午11:47:17 from Journal Citation Reports (©2019 Clarivate Analytics)

Key Indicators 2017

IMPACT METRICS		INFLUENCE METRICS		SOURCE METRICS	
Total Cites	2,560	Eigenfactor Score	0.00600	Citable Items	108
Journal Impact Factor	2.362	Article Influence Score	0.738	% Articles in Citable Items	89.81
5 Year Impact Factor	2.326	Normalized Eigenfactor	0.69500	Average JIF Percentile	57.535
Immediacy Index	1.593			Cited Half-Life	5.0
Impact Factor Without Journal Self Cites	2.167			Citing Half-Life	7.9

Source data

Journal source data 2017

	Articles	Reviews	Combined(C)	Other(O)	Percentage(C/(C+O))
**Number in JCR Year 2017 (A)**	97	11	108	43	71%
**Number of References (B)**	3,199	749	3,948	195	95%
**Ratio (B/A)**	33.0	68.1	36.6	4.5	

Downloaded on 2019/4/19 上午11:47:17 from Journal Citation Reports (©2019 Clarivate Analytics)

Box plot

Category Box Plot 2017

Category Box Plot

The category box plot depicts the distribution of Impact Factors for all journals in the category. The horizontal line that forms the top of the box is the 75th percentile (Q1). The horizontal line that forms the bottom is the 25th percentile (Q3). The horizontal line that intersects the box is the median Impact Factor for the category. Horizontal lines above and below the box, called whiskers, represent maximum and minimum values.
The top whisker is the smaller of the following two values:
the maximum Impact Factor (IF)
Q1 IF + 3.5(Q1 IF - Q3 IF)
The bottom whisker is the larger of the following two values:
the minimum Impact Factor (IF)
Q1 IF - 3.5(Q1 IF - Q3 IF)
Box Plots are provided for the current JCR year for each of the categories in which the journal is indexed.

EUR J PAEDIATR NEURO, IF: 2.362

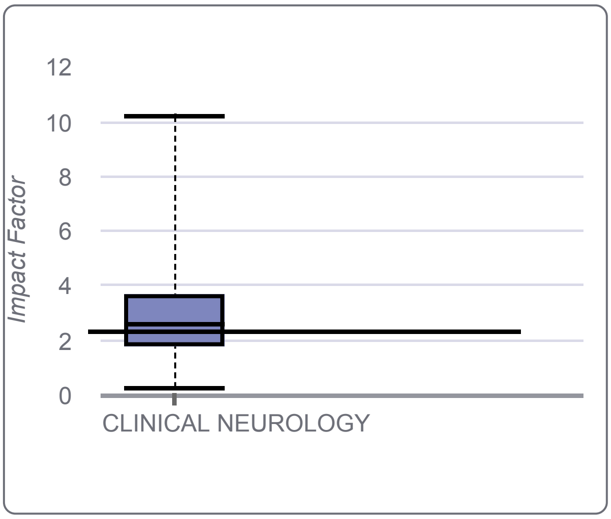

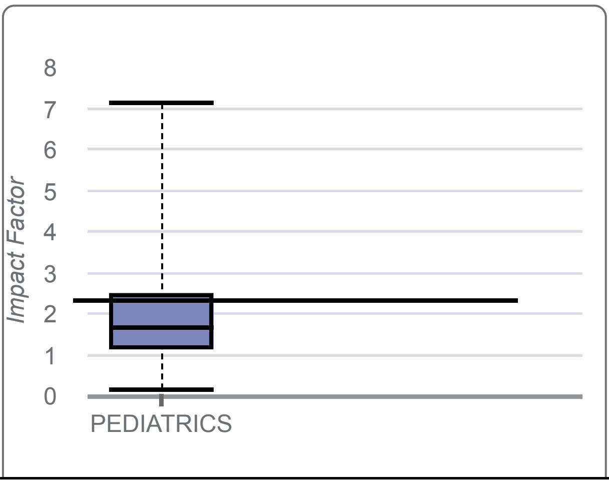

Downloaded on 2019/4/19 上午11:47:17 from Journal Citation Reports (©2019 Clarivate Analytics)

Rank

Rank 2017

JCR Impact Factor

JCR Year	CLINICAL NEUROLOGY			PEDIATRICS		
	Rank	Quartile	JIF Percentile	Rank	Quartile	JIF Percentile
2017	113/197	Q3	42.893	35/124	Q2	72.177
2016	123/194	Q3	36.856	48/121	Q2	60.744
2015	116/193	Q3	40.155	41/120	Q2	66.250
2014	95/192	Q2	50.781	30/120	Q1	75.417
2013	108/194	Q3	44.588	39/118	Q2	67.373
2012	99/193	Q3	48.964	36/122	Q2	70.902
2011	98/192	Q3	49.219	33/115	Q2	71.739
2010	93/185	Q3	50.000	33/109	Q2	70.183
2009	90/167	Q3	46.407	27/94	Q2	71.809
2008	107/156	Q3	31.731	41/86	Q2	52.907
2007	119/146	Q4	18.836	55/78	Q3	30.128
2006	92/147	Q3	37.755	33/74	Q2	56.081
2005	62/148	Q2	58.446	12/73	Q1	84.247

Downloaded on 2019/4/19 上午11:47:17 from Journal Citation Reports (©2019 Clarivate Analytics)

ESI Total Citations 2017

Rank

JCR Year	NEUROSCIENCE & BEHAVIOR
2017	213/346-Q3
2016	220/345-Q3
2015	222/344-Q3
2014	224/337-Q3
2013	225/339-Q3

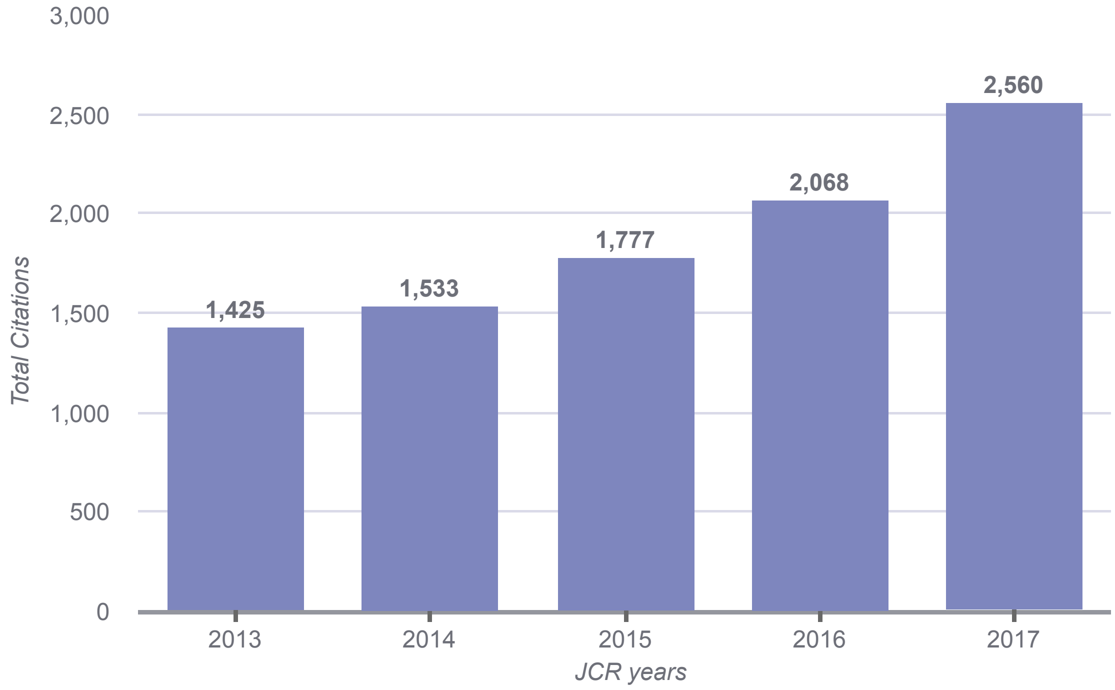

Downloaded on 2019/4/19 上午11:47:17 from Journal Citation Reports (©2019 Clarivate Analytics)

Cited Journal Data

Cited Half-Life Data

Customize columns

Cited Year	2017	2016	2015	2014	2013	2012	2011	2010	2009	2008	2
#Cites from 2017	172	317	290	249	234	271	158	185	143	125	
Cumulative %	6.72%	19.10%	30.43%	40.16%	49.30%	59.88%	66.05%	73.28%	78.87%	83.75%	1

Cited Journal Graph 2017

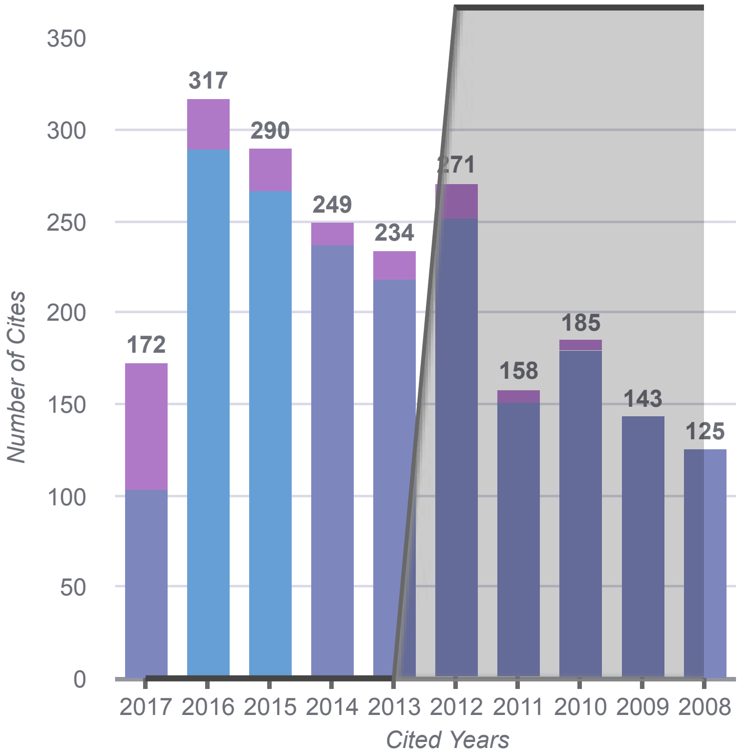

CITED JOURNAL GRAPH

The Cited Journal Graph shows the distribution (by cited year) of citations published in journals during the JCR year to items published in the Journal during the last 10 years.

The white/grey division indicates the cited half-life (if < 10.0). Half of the citations are to items that were published more recently than the cited half-life.

The two light-blue columns indicate citations used to calculate the Impact Factor (always the 2nd and 3rd columns).

Downloaded on 2019/4/19 上午11:47:17 from Journal Citation Reports (©2019 Clarivate Analytics)

Cited Journal Data

Customize columns

	Impact	Citing Journal	All Yrs	2017	2016	2015	2014	2013	2012	2011	2010	2009	2008	R
		ALL Journals	2,560	172	317	290	249	234	271	158	185	143	125	[illegible]
		ALL OTHERS (472)	472	10	41	54	46	46	50	31	35	36	24	
1	2.362	EUR J PAEDIATR NEURO	193	69	27	23	12	15	19	7	6	0	0	
2	2.398	PEDIATR NEUROL	61	0	7	4	10	12	5	6	3	1	4	
3	3.289	DEV MED CHILD NEUROL	57	5	4	9	6	4	4	5	5	2	9	
4	0.751	ACTA MEDICA MEDITERR	46	27	0	18	0	0	0	0	0	0	0	
5	2.839	SEIZURE-EUR J EPILEP	44	1	7	6	6	4	7	1	5	1	1	
6	2.600	EPILEPSY BEHAV	43	0	9	6	4	4	9	2	2	2	1	
7	1.665	J CHILD NEUROL	42	1	6	10	3	1	5	3	4	4	0	
8	5.067	EPILEPSIA	32	1	6	6	3	4	4	3	0	0	0	
9	2.766	PLOS ONE	30	2	4	1	4	2	6	3	1	0	1	
10	1.235	CHILD NERV SYST	23	2	0	4	2	5	3	1	3	0	0	
11	1.878	SEMIN PEDIATR NEUROL	23	2	6	2	1	0	1	1	2	1	0	

Rows 1 - 13 of 348 *(use csv export to download the full table)*

Downloaded on 2019/4/19 上午11:47:17 from Journal Citation Reports (©2019 Clarivate Analytics)

Citing Journal Data

Citing Half-Life Data

Customize columns

Citing Year	2017	2016	2015	2014	2013	2012	2011	2010	2009	2008	20[illegible]
#Cites from 2017	93	252	300	290	363	260	252	252	189	159	
Cumulative %	2.24%	8.33%	15.57%	22.57%	31.33%	37.61%	43.69%	49.77%	54.33%	58.17%	10[illegible]

Citing Journal Graph 2017

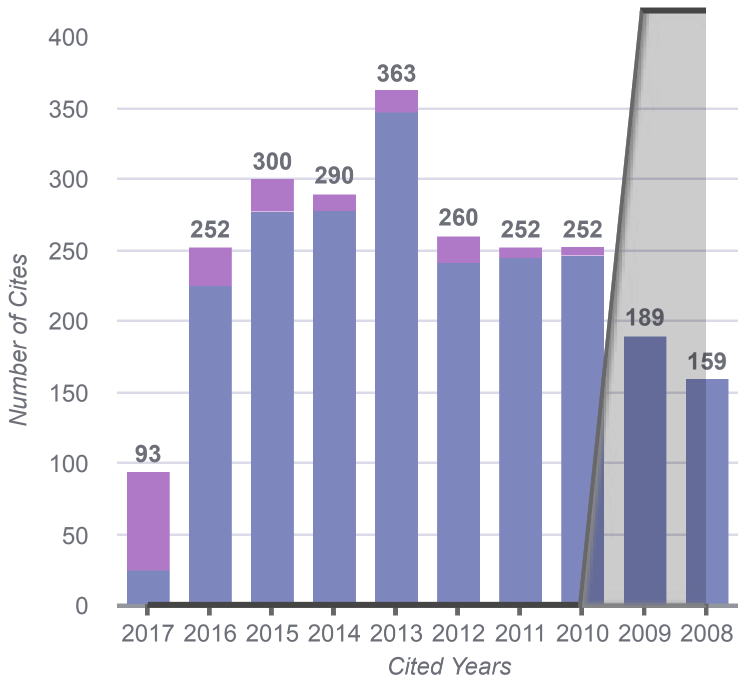

CITING JOURNAL GRAPH

The Citing Journal Graph shows the distribution (by cited year) of citations published in the Journal during the JCR year to items published in journals during the last 10 years.

The white/grey division indicates the citing half-life (if < 10.0). Half of the citations are to items that were published more recently than the citing half-life.

Downloaded on 2019/4/19 上午11:47:17 from Journal Citation Reports (©2019 Clarivate Analytics)

Citing Journal Data

Customize columns

	Impact	Cited Journal	All Yrs	2017	2016	2015	2014	2013	2012	2011	2010	2009	2008	R
		ALL Journals	4,143	93	252	300	290	363	260	252	252	189	159	1,
		ALL OTHERS (693)	693	7	40	36	45	57	38	44	25	31	28	
1	3.289	DEV MED CHILD NEUROL	193	0	20	5	16	29	9	16	18	4	8	
2	2.362	EUR J PAEDIATR NEURO	193	69	27	23	12	15	19	7	6	0	0	
3	8.055	NEUROLOGY	144	1	5	11	4	7	8	10	8	7	4	
4	8.324	MOVEMENT DISORD	92	0	2	2	4	39	3	8	12	3	1	
5	5.067	EPILEPSIA	91	0	3	10	7	5	5	3	11	5	3	
6	1.665	J CHILD NEUROL	86	1	6	8	8	7	2	7	6	2	3	
7	10.848	BRAIN	79	1	2	5	10	1	2	7	9	4	6	
8	7.144	J NEUROL NEUROSUR PS	70	1	8	3	18	5	0	2	5	3	0	
9	27.144	LANCET NEUROL	69	1	11	2	4	3	3	8	2	11	9	
10	5.515	PEDIATRICS	61	0	0	3	2	5	4	5	3	4	5	
11	10.250	ANN NEUROL	58	0	4	0	4	1	5	3	6	1	2	
12	2.398	PEDIATR NEUROL	52	0	1	1	4	3	5	6	4	6	3	

Rows 1 - 14 of 405 *(use csv export to download the full table)*

Downloaded on 2019/4/19 上午11:47:17 from Journal Citation Reports (©2019 Clarivate Analytics)

Metric trend

Metric Trend

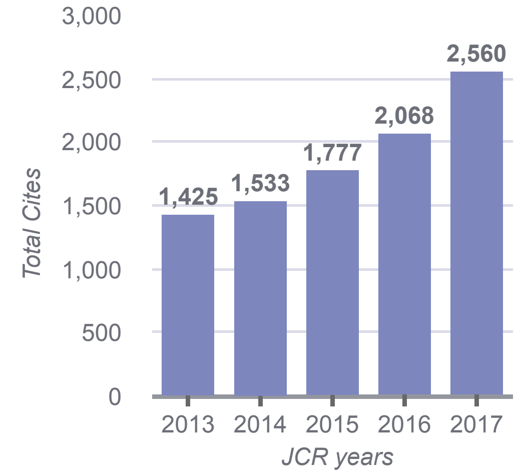

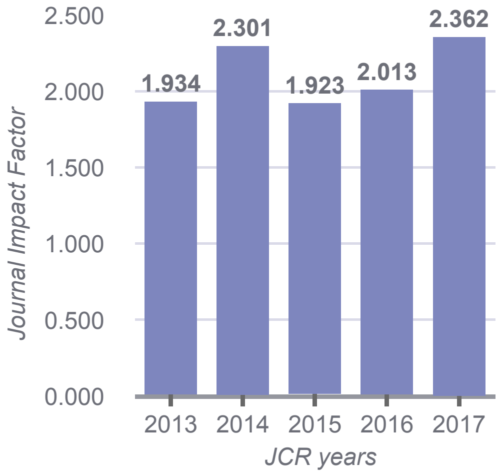

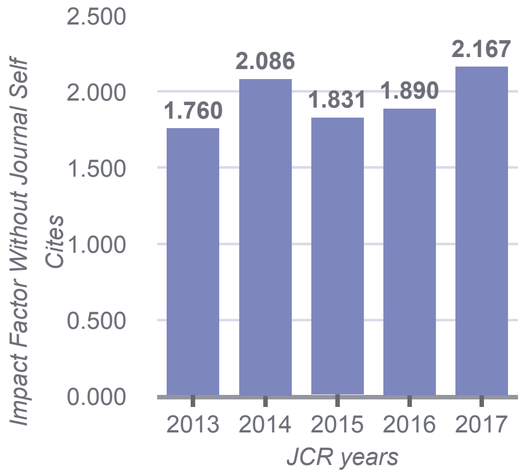

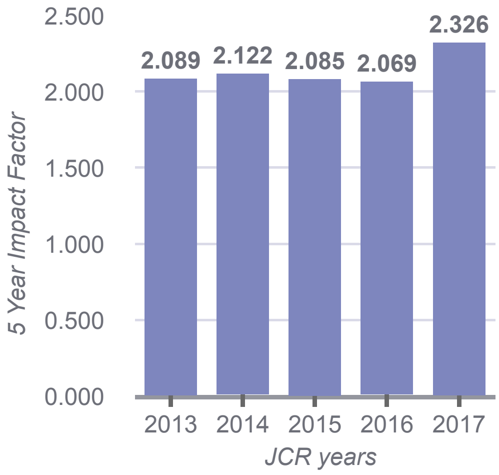

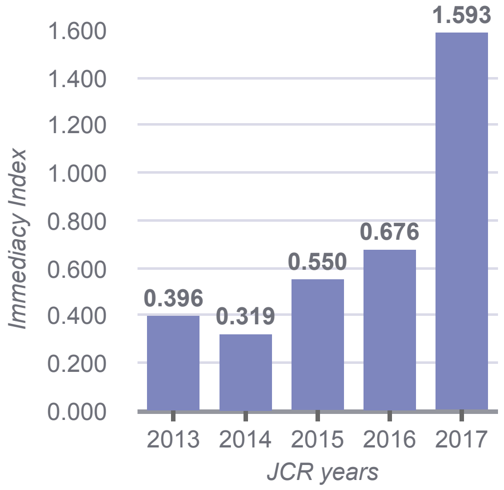

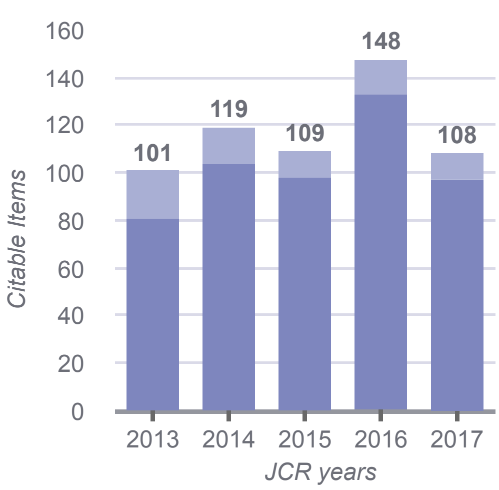

Downloaded on 2019/4/19 上午11:47:17 from Journal Citation Reports (©2019 Clarivate Analytics)

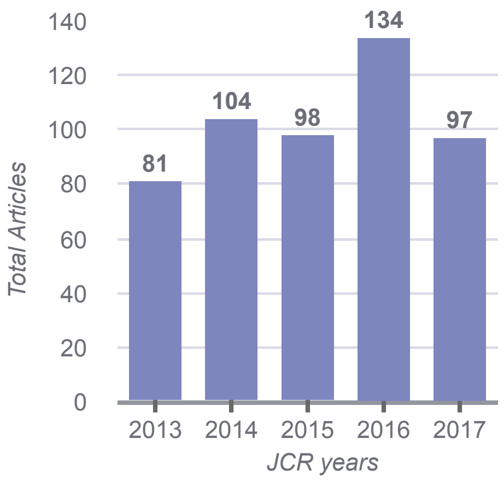

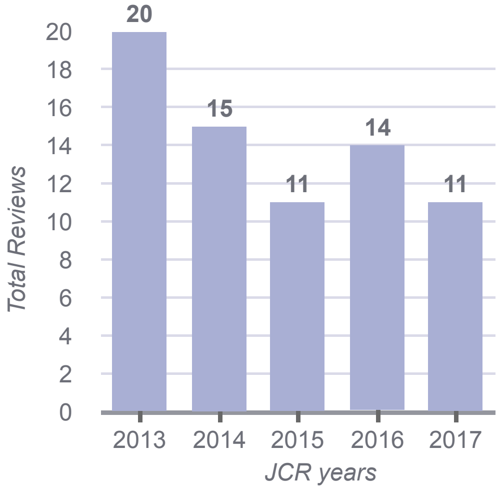

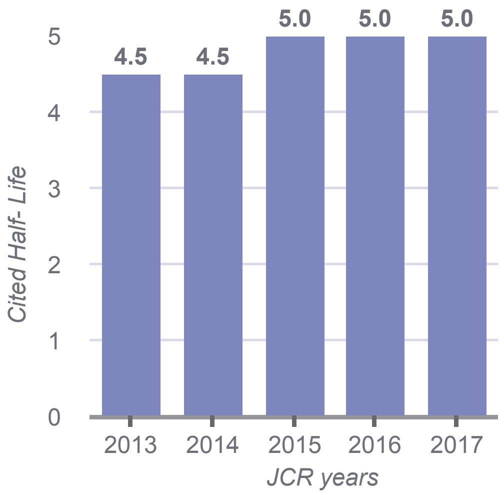

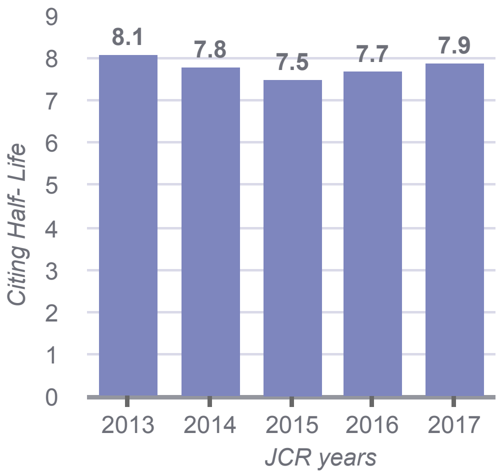

Downloaded on 2019/4/19 上午11:47:17 from Journal Citation Reports (©2019 Clarivate Analytics)

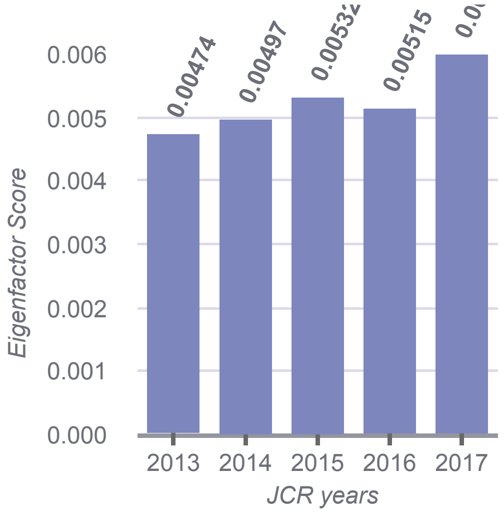

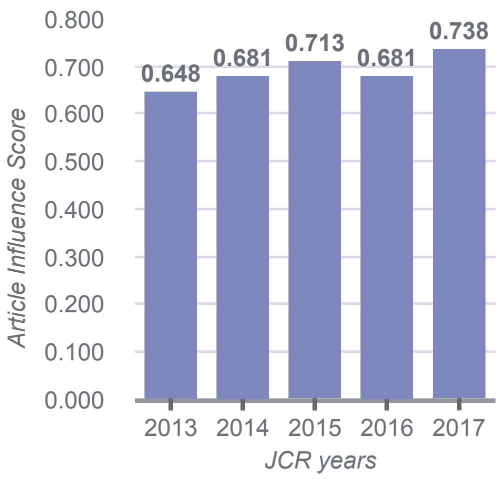

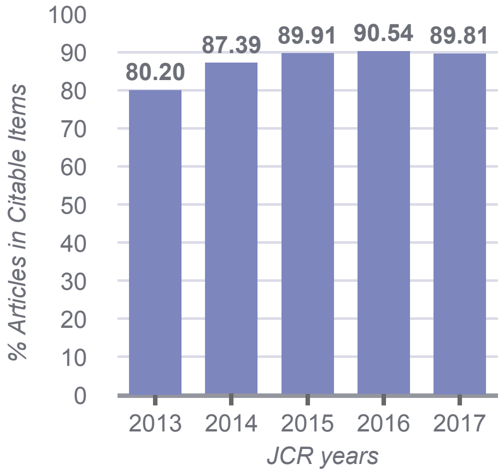

Downloaded on 2019/4/19 上午11:47:17 from Journal Citation Reports (©2019 Clarivate Analytics)

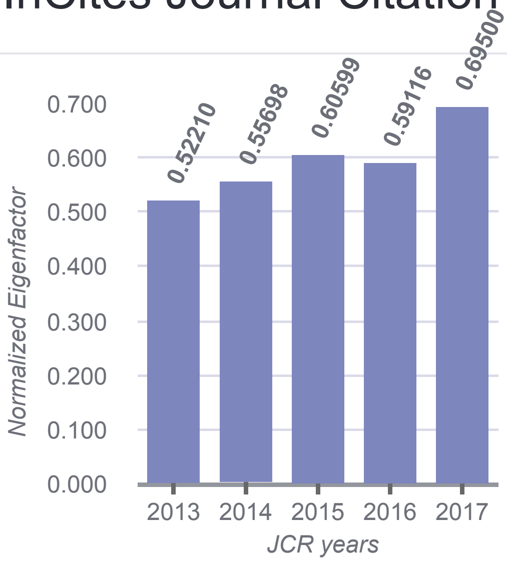

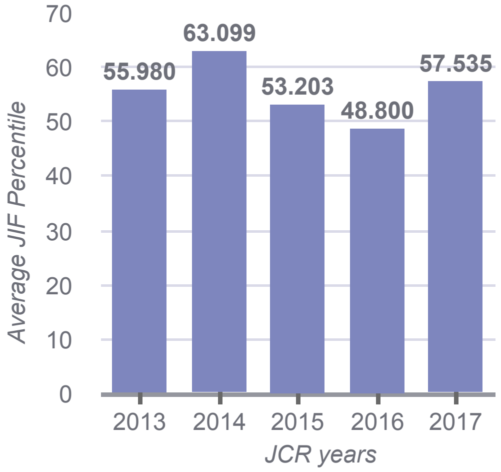

Downloaded on 2019/4/19 上午11:47:17 from Journal Citation Reports (©2019 Clarivate Analytics)

These data summarize the characteristics of the journal's published content for the most recent three years, that is, 2017 and the two prior years, combined. This information is based on all listed authors and addresses. It is meant to be descriptive rather than comparative.

Contributions by country/region

country	count
1. England	77
2. Italy	66
3. GERMANY (FED REP GER)	51
4. Netherlands	50
5. France	43
6. USA	35
7. Belgium	26
8. Spain	23
9. Switzerland	17
10. Austria	16
- Canada	16

Contributions by organizations

organization	count
1. UNIVERSITY OF LONDON	45
2. GUY'S & ST THOMAS' NHS FOUNDATION TRUST	25
3. ASSISTANCE PUBLIQUE HOPITAUX PARIS (APHP)	19
4. INSTITUT NATIONAL DE LA SANTE ET DE LA RECHERCHE MEDICALE (INSERM)	18
5. SAPIENZA UNIVERSITY ROME	17
6. MAASTRICHT UNIVERSITY	15
7. TEL AVIV UNIVERSITY	14
- IRCCS BAMBINO GESU	14
9. UNIVERSITE SORBONNE PARIS CITE-USPC (COMUE)	13
10. KU LEUVEN	12
- UNIVERSITY OF MUNICH	12

Downloaded on 2019/4/19 上午11:47:17 from Journal Citation Reports (©2019 Clarivate Analytics)
