# Supplementary material for: Emerging trends and knowledge structure of epilepsy during pregnancy research for 2000–2018: a bibliometric analysis
Source: PeerJ. 2019 Jun 7;7:e7115. doi: 10.7717/peerj.7115 (PMC6557303; doi:10.7717/peerj.7115)
Supplement: Supplemental Information 4 [file peerj-07-7115-s004.zip › 7/13. InCites Journal Citation Reports(JOURNAL OF CHILD NEUROLOGY).pdf]

2017 Journal Performance Data for: JOURNAL OF CHILD NEUROLOGY

ISSN: 0883-0738
eISSN: 1708-8283
SAGE PUBLICATIONS INC
2455 TELLER RD, THOUSAND OAKS, CA 91320
USA

TITLES
ISO: J. Child Neurol.
JCR Abbrev: J CHILD NEUROL

LANGUAGES
English

CATEGORIES
CLINICAL NEUROLOGY - SCIE

PEDIATRICS - SCIE

PUBLICATION FREQUENCY
14 issues/year

Downloaded on 2019/4/19 上午11:48:15 from Journal Citation Reports (©2019 Clarivate Analytics)

Current Year

The data in the two graphs below and in the Journal Impact Factor calculation panels represent citation activity in 2017 to items published in the journal in the prior two years. They detail the components of the Journal Impact Factor. Use the "All Years" tab to access key metrics and additional data for the current year and all prior years for this journal.

2017 Journal Impact Factor & percentile rank in category for: JOURNAL OF CHILD NEUROLOGY

1.665
2017 Journal Impact Factor

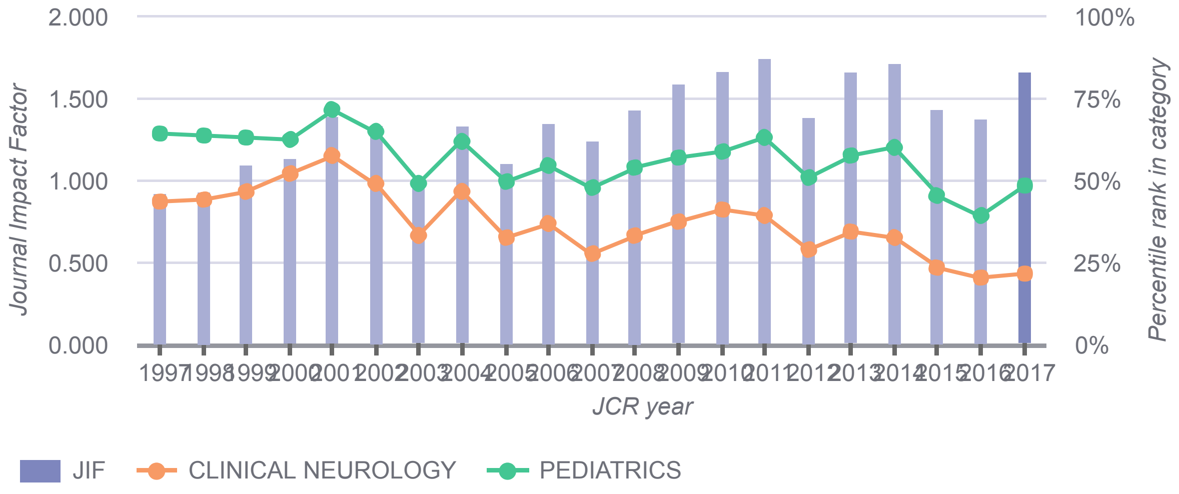

2017 JIF Citation Distribution for: JOURNAL OF CHILD NEUROLOGY

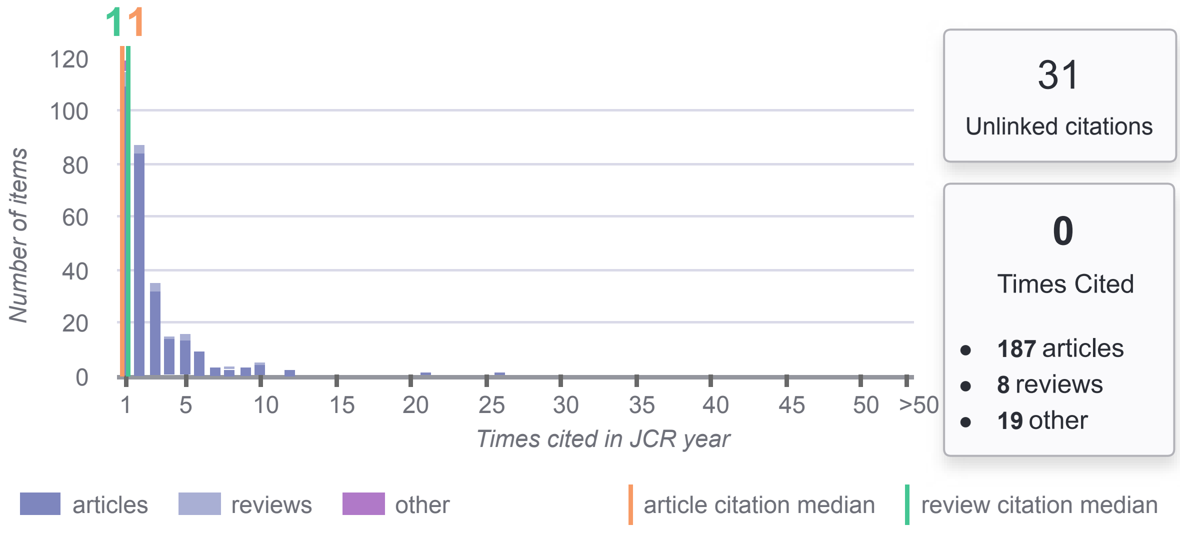

Downloaded on 2019/4/19 上午11:48:15 from Journal Citation Reports (©2019 Clarivate Analytics)

Journal Impact Factor Calculation

$$\text{2017 Journal Impact Factor} = \frac{816}{490} = 1.665$$

How is Journal Impact Factor Calculated?

$$\text{JIF} = \frac{\text{Citations in 2017 to items published in } \mathbf{2015\ (424) + 2016\ (392)}}{\text{Number of citable items in } \mathbf{2015\ (291) + 2016\ (199)}} = \frac{816}{490}$$

Downloaded on 2019/4/19 上午11:48:15 from Journal Citation Reports (©2019 Clarivate Analytics)

Journal Impact Factor contributing items

Citable items in 2016 and 2015 (490)

TITLE	CITATIONS COUNTED TOWARDS JIF
Intrathecal Injections in Children With Spinal Muscular Atrophy: Nusinersen Clinical Trial Experience<br>By: Hache, Manon; Swoboda, Kathryn J.; Sethna, Navil; Farrow-Gillespie, Alan; Khandji, Alexander; et al.<br>**Volume: 31 Page: 899-906 Accession number: WOS:000376264800014**<br>**Document Type: Article**	26
The Epidemiology of Traumatic Brain Injury in Children and Youths: A Review of Research Since 1990<br>By: Thurman, David J.<br>**Volume: 31 Page: 20-27 Accession number: WOS:000366650400002**<br>**Document Type: Article**	21
Evaluation of Basal Ganglia and Thalamic Inflammation in Children With Pediatric Autoimmune Neuropsychiatric Disorders Associated With Streptococcal Infection and Tourette Syndrome: A Positron Emission Tomographic (PET) Study Using C-11-[R]-PK11195<br>By: Kumar, Ajay; Williams, Mitchel T.; Chugani, Harry T.<br>**Volume: 30 Page: 749-756 Accession number: WOS:000353584100010**<br>**Document Type: Article**	12
Autism Spectrum Disorder and Epilepsy: Two Sides of the Same Coin?<br>By: Jeste, Shafali Spurling; Tuchman, Roberto<br>**Volume: 30 Page: 1963-1971 Accession number: WOS:000364914000010**<br>**Document Type: Article**	12
Early Infant Development and Intervention for Autism Spectrum Disorder<br>By: Sacrey, Lori-Ann R.; Bennett, Jeffrey A.; Zwaigenbaum, Lonnie<br>**Volume: 30 Page: 1921-1929 Accession number: WOS:000364914000006**<br>**Document Type: Article**	10
Late Effects of Treatment of Pediatric Central Nervous System Tumors<br>By: Roddy, Erika; Mueller, Sabine<br>**Volume: 31 Page: 237-254 Accession number: WOS:000368934800018**<br>**Document Type: Review**	10
Medical-School Partnership in Guiding Return to School Following Mild Traumatic Brain Injury in Youth<br>By: Gioia, Gerard A.<br>**Volume: 31 Page: 93-108 Accession number: WOS:000366650400010**<br>**Document Type: Article**	10

Downloaded on 2019/4/19 上午11:48:15 from Journal Citation Reports (©2019 Clarivate Analytics)

Citations in 2017 (816)

TITLE	CITATIONS COUNTED TOWARDS JIF
JOURNAL OF CHILD NEUROLOGY	31
PEDIATRIC NEUROLOGY	18
DEVELOPMENTAL MEDICINE AND CHILD NEUROLOGY	16
EUROPEAN JOURNAL OF PAEDIATRIC NEUROLOGY	14
CURRENT PHARMACEUTICAL DESIGN	13
PLOS ONE	11
SCIENTIFIC REPORTS	11
SEMINARS IN PEDIATRIC NEUROLOGY	10
CHILDS NERVOUS SYSTEM	9
FRONTIERS IN NEUROLOGY	8

Downloaded on 2019/4/19 上午11:48:15 from Journal Citation Reports (©2019 Clarivate Analytics)

Clarivate Analytics

Key Indicators 2017

IMPACT METRICS		INFLUENCE METRICS		SOURCE METRICS	
Total Cites	6,006	Eigenfactor Score	0.00900	Citable Items	137
Journal Impact Factor	1.665	Article Influence Score	0.525	% Articles in Citable Items	87.59
5 Year Impact Factor	1.600	Normalized Eigenfactor	1.05300	Average JIF Percentile	35.182
Immediacy Index	0.526			Cited Half-Life	8.8
Impact Factor Without Journal Self Cites	1.602			Citing Half-Life	8.5

Source data

Journal source data 2017

	Articles	Reviews	Combined(C)	Other(O)	Percentage(C/(C+O))
Number in JCR Year 2017 (A)	120	17	137	17	88%
Number of References (B)	3,390	1,131	4,521	82	98%
Ratio (B/A)	28.3	66.5	33.0	4.8	

Downloaded on 2019/4/19 上午11:48:15 from Journal Citation Reports (©2019 Clarivate Analytics)

Box plot

Category Box Plot 2017

Category Box Plot

The category box plot depicts the distribution of Impact Factors for all journals in the category. The horizontal line that forms the top of the box is the 75th percentile (Q1). The horizontal line that forms the bottom is the 25th percentile (Q3). The horizontal line that intersects the box is the median Impact Factor for the category. Horizontal lines above and below the box, called whiskers, represent maximum and minimum values.
The top whisker is the smaller of the following two values:
the maximum Impact Factor (IF)
Q1 IF + 3.5(Q1 IF - Q3 IF)
The bottom whisker is the larger of the following two values:
the minimum Impact Factor (IF)
Q1 IF - 3.5(Q1 IF - Q3 IF)
Box Plots are provided for the current JCR year for each of the categories in which the journal is indexed.

J CHILD NEUROL, IF: 1.665

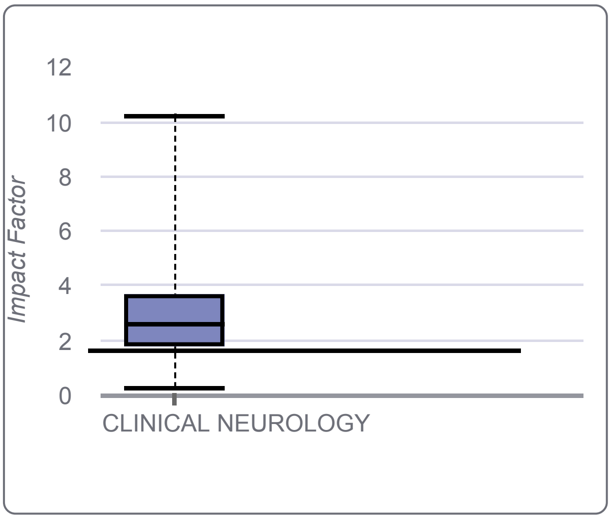

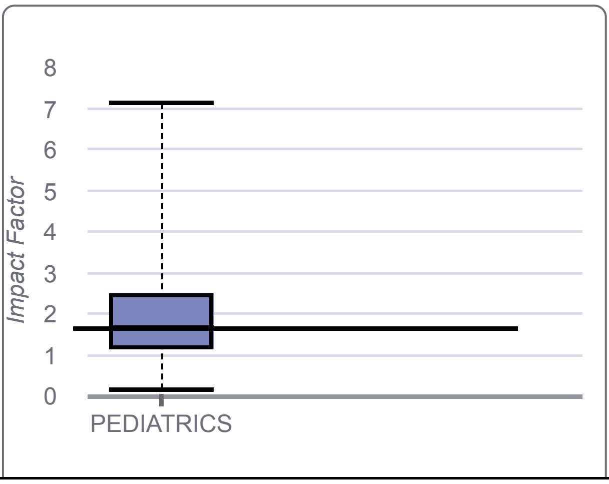

Downloaded on 2019/4/19 上午11:48:15 from Journal Citation Reports (©2019 Clarivate Analytics)

Rank

Rank 2017

JCR Impact Factor

JCR Year	CLINICAL NEUROLOGY			PEDIATRICS		
	Rank	Quartile	JIF Percentile	Rank	Quartile	JIF Percentile
2017	155/197	Q4	21.574	64/124	Q3	48.790
2016	155/194	Q4	20.361	74/121	Q3	39.256
2015	148/193	Q4	23.575	66/120	Q3	45.417
2014	130/192	Q3	32.552	48/120	Q2	60.417
2013	128/194	Q3	34.278	50/118	Q2	58.051
2012	138/193	Q3	28.756	60/122	Q2	51.230
2011	117/192	Q3	39.323	43/115	Q2	63.043
2010	109/185	Q3	41.351	45/109	Q2	59.174
2009	105/167	Q3	37.425	41/94	Q2	56.915
2008	105/156	Q3	33.013	40/86	Q2	54.070
2007	106/146	Q3	27.740	41/78	Q3	48.077
2006	93/147	Q3	37.075	34/74	Q2	54.730
2005	100/148	Q3	32.770	37/73	Q3	50.000
2004	75/140	Q3	46.786	27/70	Q2	62.143
2003	91/135	Q3	32.963	35/68	Q3	49.265
2002	71/138	Q3	48.913	24/68	Q2	65.441
2001	58/136	Q2	57.721	20/69	Q2	71.739
2000	66/137	Q2	52.190	27/71	Q2	62.676
1999	71/132	Q3	46.591	27/72	Q2	63.194
1998	70/125	Q3	44.400	26/71	Q2	64.085

Downloaded on 2019/4/19 上午11:48:15 from Journal Citation Reports (©2019 Clarivate Analytics)

Downloaded on 2019/4/19 上午11:48:15 from Journal Citation Reports (©2019 Clarivate Analytics)

ESI Total Citations 2017

Rank

JCR Year	NEUROSCIENCE & BEHAVIOR
2017	121/346-Q2
2016	116/345-Q2
2015	115/344-Q2
2014	111/337-Q2
2013	105/339-Q2

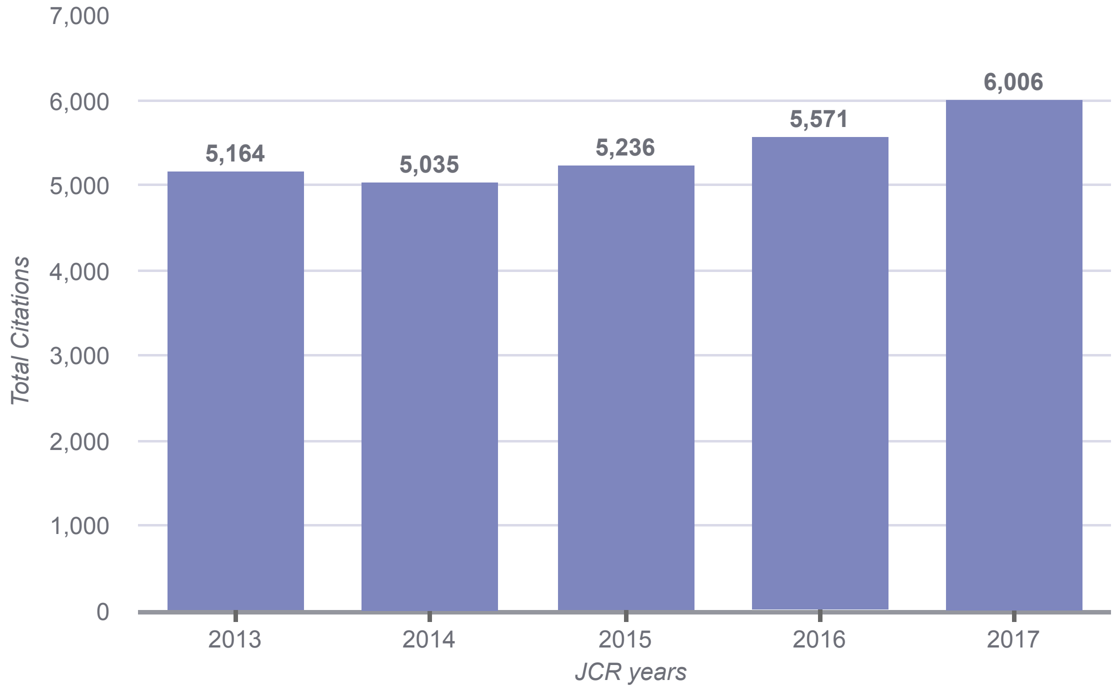

Downloaded on 2019/4/19 上午11:48:15 from Journal Citation Reports (©2019 Clarivate Analytics)

Cited Journal Data

Cited Half-Life Data

Customize columns

Cited Year	2017	2016	2015	2014	2013	2012	2011	2010	2009	2008	2(
#Cites from 2017	72	392	424	498	364	317	348	285	398	330	
Cumulative %	1.20%	7.73%	14.79%	23.08%	29.14%	34.42%	40.21%	44.96%	51.58%	57.08%	1(

Cited Journal Graph 2017

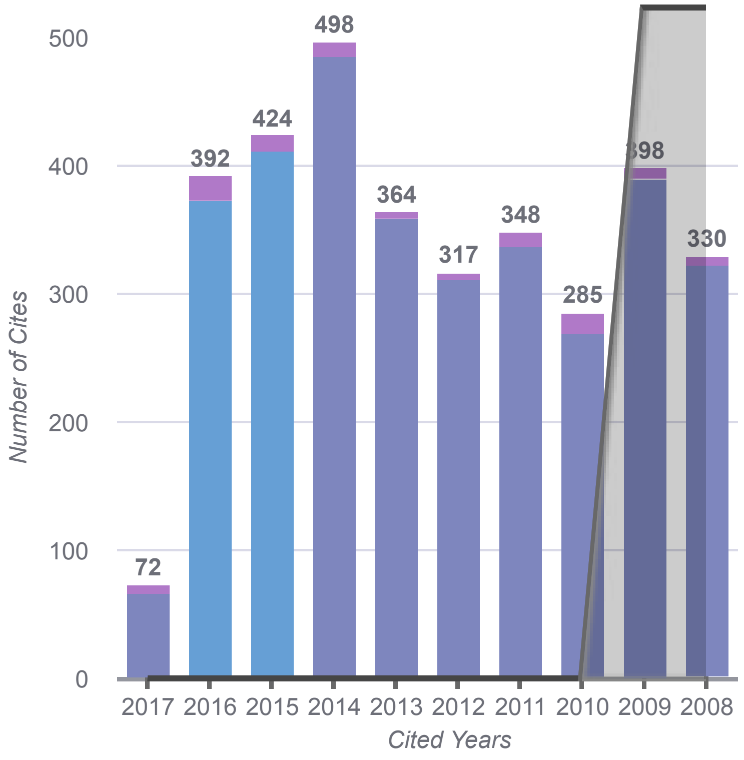

CITED JOURNAL GRAPH

The Cited Journal Graph shows the distribution (by cited year) of citations published in journals during the JCR year to items published in the Journal during the last 10 years.

The white/grey division indicates the cited half-life (if < 10.0). Half of the citations are to items that were published more recently than the cited half-life.

The two light-blue columns indicate citations used to calculate the Impact Factor (always the 2nd and 3rd columns).

Downloaded on 2019/4/19 上午11:48:15 from Journal Citation Reports (©2019 Clarivate Analytics)

Cited Journal Data

Customize columns

	Impact	Citing Journal	All Yrs	2017	2016	2015	2014	2013	2012	2011	2010	2009	2008	R
		ALL Journals	6,006	72	392	424	498	364	317	348	285	398	330	2,
		ALL OTHERS (804)	804	12	42	58	65	43	42	41	39	61	56	
1	2.398	PEDIATR NEUROL	143	4	8	10	12	10	12	9	4	9	10	
2	1.665	J CHILD NEUROL	140	6	19	12	11	5	4	11	15	8	6	
3	3.289	DEV MED CHILD NEUROL	101	0	7	9	16	8	6	10	3	2	7	
4	2.766	PLOS ONE	88	0	7	4	5	8	5	11	2	5	7	
5	2.362	EUR J PAEDIATR NEURO	86	1	6	8	8	7	2	7	6	2	3	
6	1.878	SEMIN PEDIATR NEUROL	80	2	5	5	7	5	6	5	2	6	5	
7	4.122	SCI REP-UK	77	0	7	4	6	3	4	4	3	6	5	
8	2.600	EPILEPSY BEHAV	60	0	5	3	2	3	6	5	1	3	4	
9	2.839	SEIZURE-EUR J EPILEP	56	1	0	5	3	4	4	0	1	2	1	
10	5.067	EPILEPSIA	49	0	4	2	3	7	3	3	4	1	1	
11		SPIN MUSC ATR DISXB	46	0	0	0	4	3	4	4	0	2	1	

Rows 1 - 13 of 740 *(use csv export to download the full table)*

Downloaded on 2019/4/19 上午11:48:15 from Journal Citation Reports (©2019 Clarivate Analytics)

Citing Journal Data

Citing Half-Life Data

Customize columns

Citing Year	2017	2016	2015	2014	2013	2012	2011	2010	2009	2008	20
#Cites from 2017	40	228	310	340	329	310	320	270	275	179	
Cumulative %	0.87%	5.82%	12.56%	19.94%	27.09%	33.83%	40.78%	46.64%	52.62%	56.51%	10

Citing Journal Graph 2017

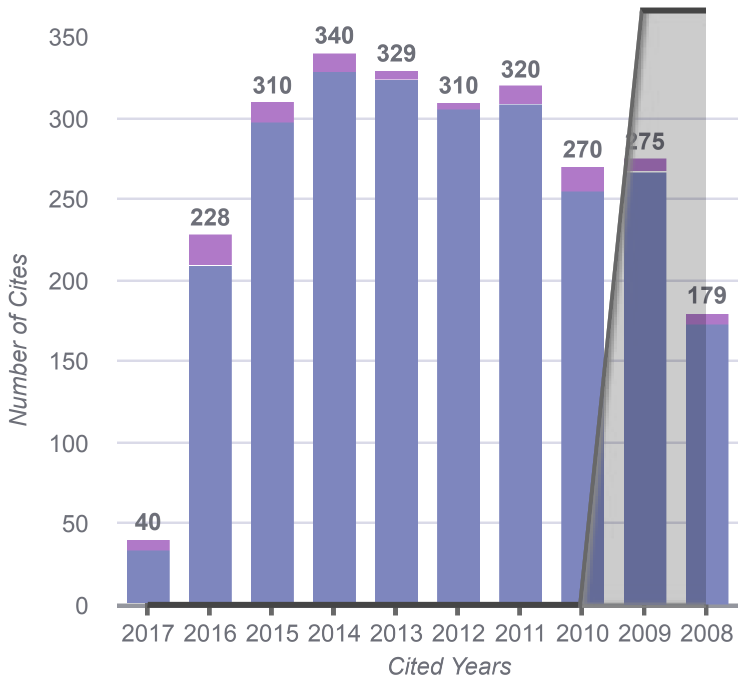

CITING JOURNAL GRAPH

The Citing Journal Graph shows the distribution (by cited year) of citations published in the Journal during the JCR year to items published in journals during the last 10 years.

The white/grey division indicates the citing half-life (if < 10.0). Half of the citations are to items that were published more recently than the citing half-life.

Downloaded on 2019/4/19 上午11:48:15 from Journal Citation Reports (©2019 Clarivate Analytics)

Citing Journal Data

Customize columns

	Impact	Cited Journal	All Yrs	2017	2016	2015	2014	2013	2012	2011	2010	2009	2008	R
		ALL Journals	4,603	40	228	310	340	329	310	320	270	275	179	2,
		ALL OTHERS (804)	804	12	35	64	56	63	56	54	31	43	28	
1	8.055	NEUROLOGY	187	0	8	13	12	17	12	15	13	7	5	
2	5.067	EPILEPSIA	150	1	6	11	12	13	8	14	9	7	15	
3	1.665	J CHILD NEUROL	140	6	19	12	11	5	4	11	15	8	6	
4	5.515	PEDIATRICS	113	0	6	4	7	6	5	8	8	2	6	
5	3.289	DEV MED CHILD NEUROL	111	2	6	3	6	7	8	5	9	11	6	
6	2.398	PEDIATR NEUROL	86	1	3	8	9	7	5	5	3	5	5	
7	10.250	ANN NEUROL	69	0	3	1	7	0	7	6	4	6	3	
8	10.848	BRAIN	52	0	1	3	5	4	2	2	4	3	5	
9	3.667	J PEDIATR-US	52	0	2	4	0	5	2	5	6	10	2	
10	27.144	LANCET NEUROL	49	0	4	3	5	7	1	2	10	4	5	
11	2.600	EPILEPSY BEHAV	48	1	5	6	5	3	2	4	5	3	4	
12	1.544	BRAIN DEV-JPN	46	0	2	4	1	1	5	3	4	3	0	
13	2.264	AM J MED GENET A	43	0	1	2	7	2	2	4	5	1	3	

Rows 1 - 15 of 494 *(use csv export to download the full table)*

Downloaded on 2019/4/19 上午11:48:15 from Journal Citation Reports (©2019 Clarivate Analytics)

Metric trend

Metric Trend

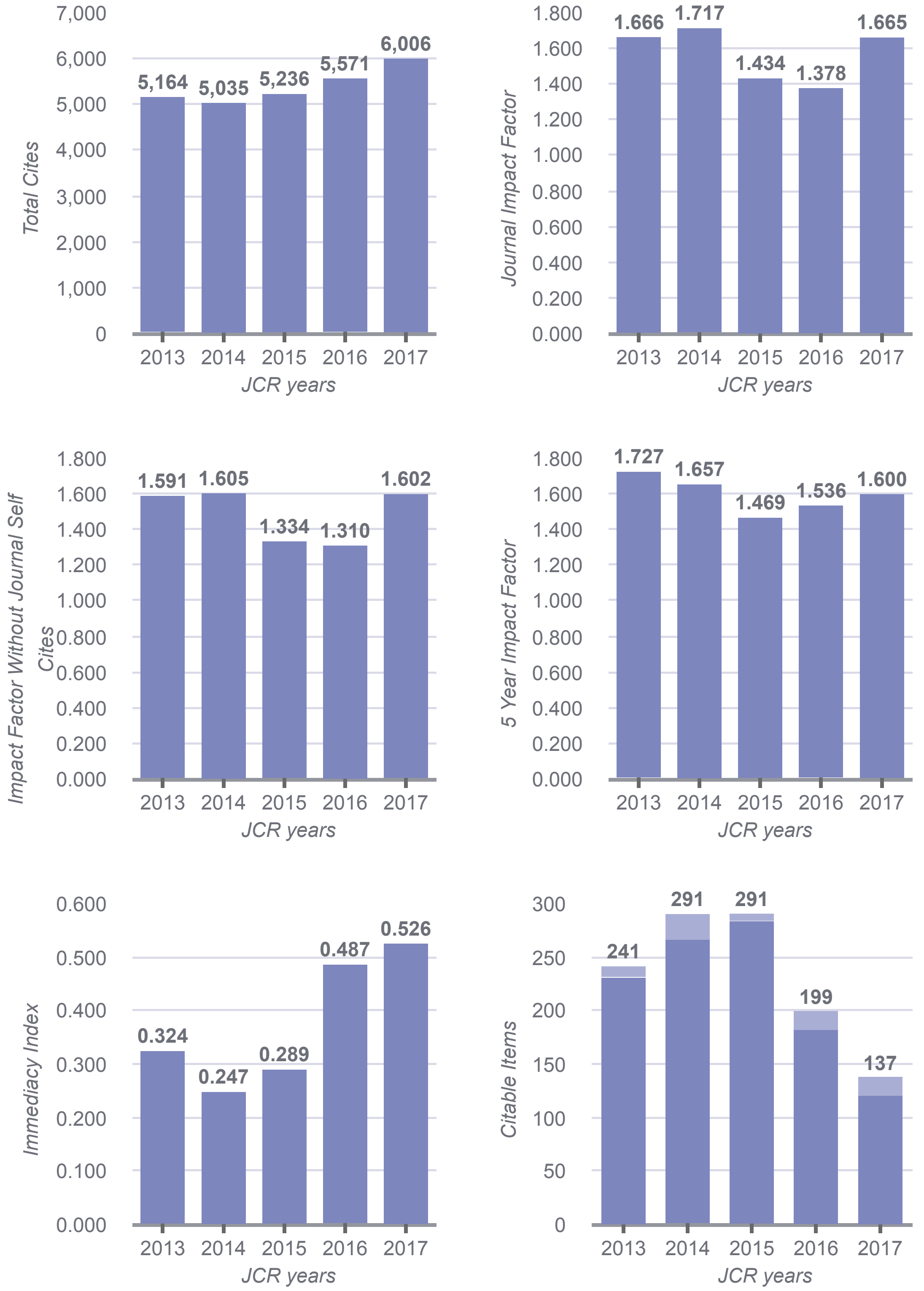

Downloaded on 2019/4/19 上午11:48:15 from Journal Citation Reports (©2019 Clarivate Analytics)

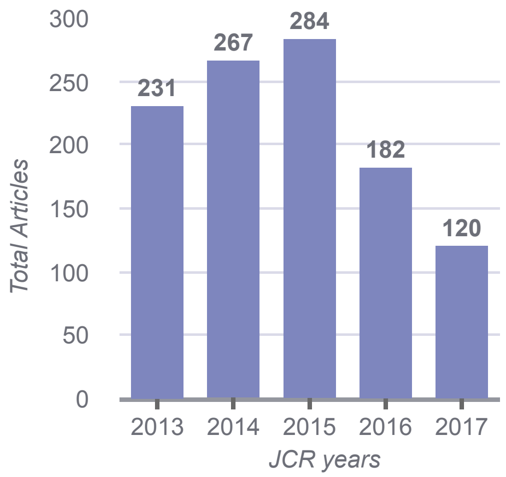

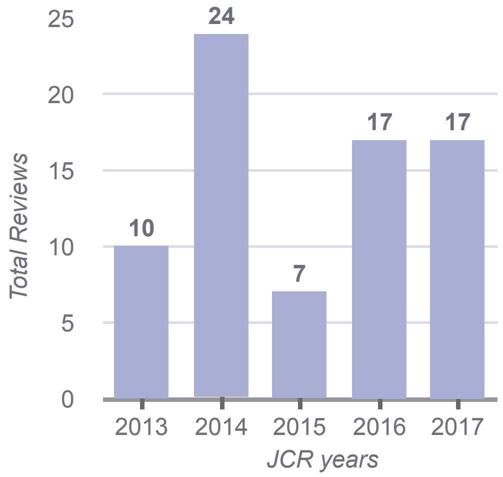

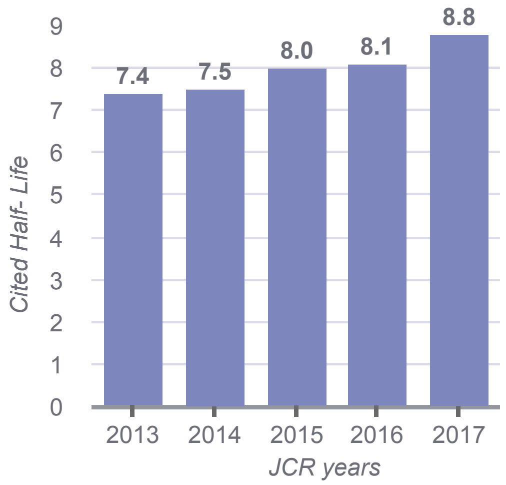

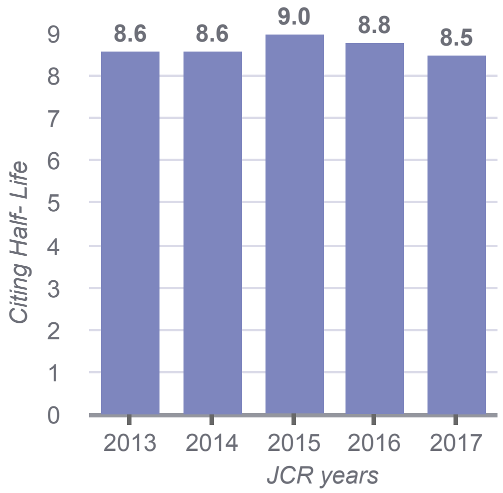

Downloaded on 2019/4/19 上午11:48:15 from Journal Citation Reports (©2019 Clarivate Analytics)

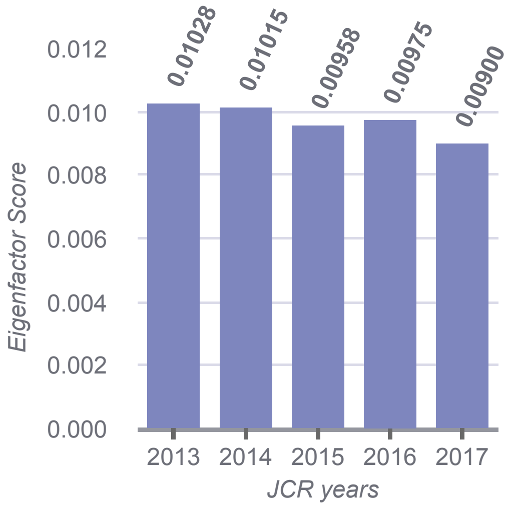

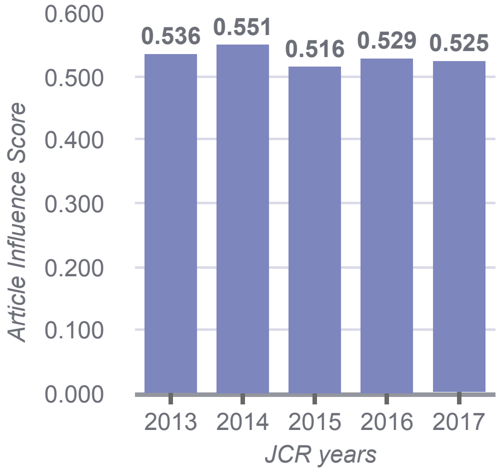

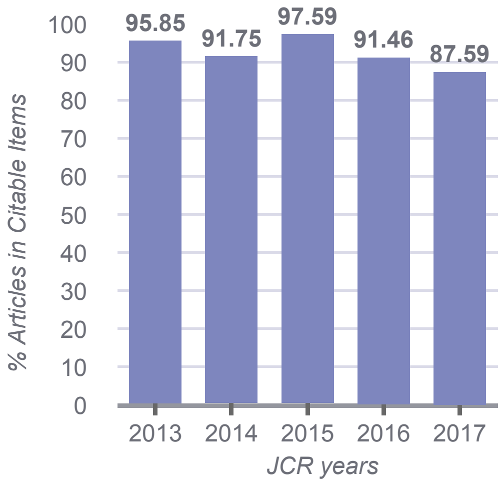

Downloaded on 2019/4/19 上午11:48:15 from Journal Citation Reports (©2019 Clarivate Analytics)

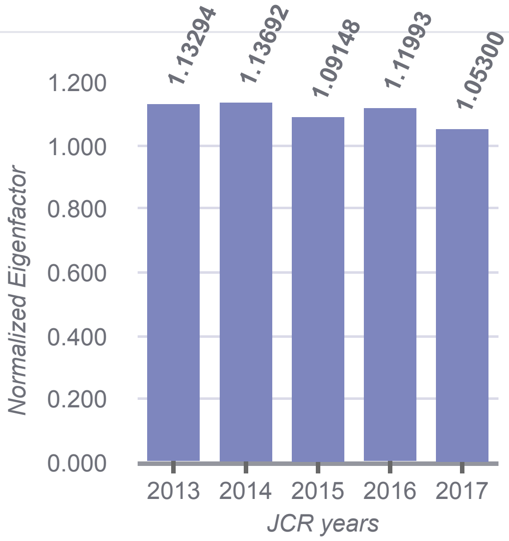

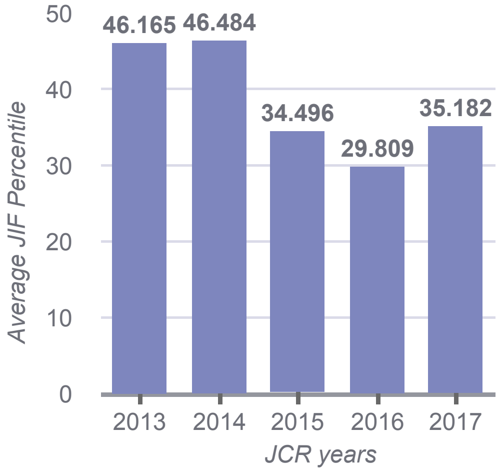

Downloaded on 2019/4/19 上午11:48:15 from Journal Citation Reports (©2019 Clarivate Analytics)

These data summarize the characteristics of the journal's published content for the most recent three years, that is, 2017 and the two prior years, combined. This information is based on all listed authors and addresses. It is meant to be descriptive rather than comparative.

Contributions by country/region

country	count
1. USA	293
2. Turkey	62
3. Canada	48
4. CHINA MAINLAND	42
5. India	38
6. Italy	31
7. Israel	26
8. England	25
9. Netherlands	20
10. GERMANY (FED REP GER)	15
- South Korea	15

Contributions by organizations

organization	count
1. HARVARD UNIVERSITY	36
2. UNIVERSITY OF CALIFORNIA SYSTEM	32
3. VA BOSTON HEALTHCARE SYSTEM	29
4. BOSTON CHILDREN'S HOSPITAL	26
5. OHIO STATE UNIVERSITY	23
6. MAYO CLINIC	21
7. TEL AVIV UNIVERSITY	20
8. UNIVERSITY OF TORONTO	19
9. JOHNS HOPKINS UNIVERSITY	13
- HOSPITAL FOR SICK CHILDREN (SICKKIDS)	13

Downloaded on 2019/4/19 上午11:48:15 from Journal Citation Reports (©2019 Clarivate Analytics)
