# Supplementary material for: Emerging trends and knowledge structure of epilepsy during pregnancy research for 2000–2018: a bibliometric analysis
Source: PeerJ. 2019 Jun 7;7:e7115. doi: 10.7717/peerj.7115 (PMC6557303; doi:10.7717/peerj.7115)
Supplement: Supplemental Information 4 [file peerj-07-7115-s004.zip › 7/14. InCites Journal Citation Reports(BRAIN & DEVELOPMENT).pdf]

2017 Journal Performance Data for: BRAIN & DEVELOPMENT

ISSN: 0387-7604
eISSN: 1872-7131
ELSEVIER SCIENCE BV
PO BOX 211, 1000 AE AMSTERDAM, NETHERLANDS
NETHERLANDS

TITLES
ISO: Brain Dev.
JCR Abbrev: BRAIN DEV-JPN

LANGUAGES
English

CATEGORIES
CLINICAL NEUROLOGY - SCIE

PUBLICATION FREQUENCY
10 issues/year

Downloaded on 2019/4/19 上午11:49:14 from Journal Citation Reports (©2019 Clarivate Analytics)

Current Year

The data in the two graphs below and in the Journal Impact Factor calculation panels represent citation activity in 2017 to items published in the journal in the prior two years. They detail the components of the Journal Impact Factor. Use the "All Years" tab to access key metrics and additional data for the current year and all prior years for this journal.

2017 Journal Impact Factor & percentile rank in category for: BRAIN & DEVELOPMENT

1.544

2017 Journal Impact Factor

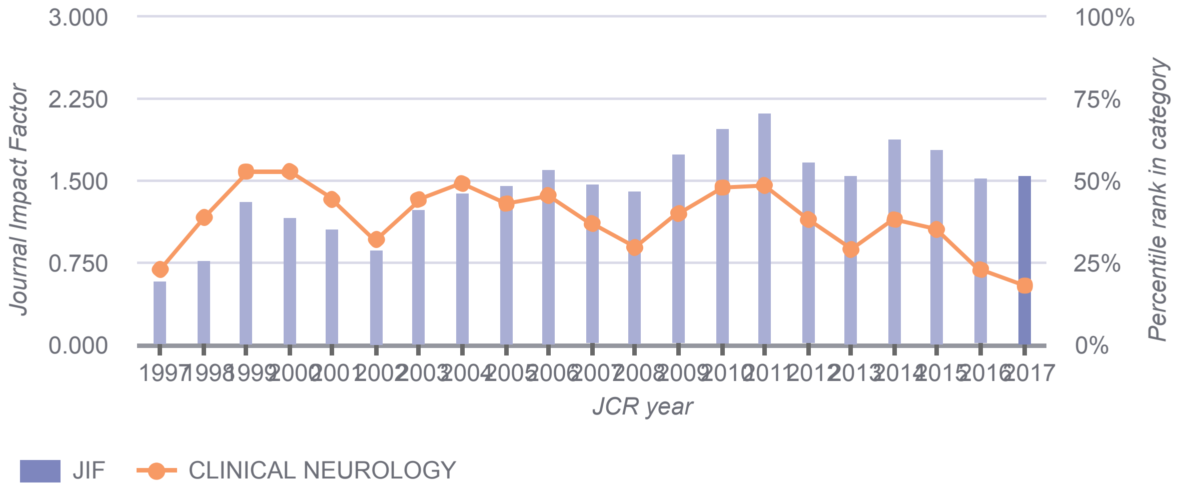

2017 JIF Citation Distribution for: BRAIN & DEVELOPMENT

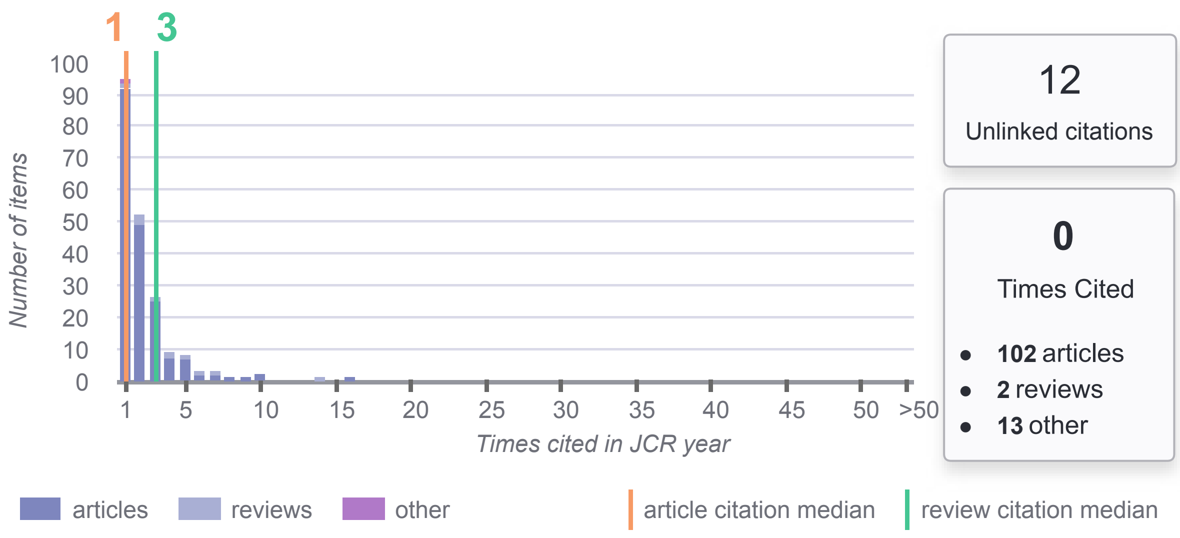

Downloaded on 2019/4/19 上午11:49:14 from Journal Citation Reports (©2019 Clarivate Analytics)

Journal Impact Factor Calculation

$$2017 \text{ Journal Impact Factor} = \frac{471}{305} = 1.544$$

How is Journal Impact Factor Calculated?

$$\text{JIF} = \frac{\text{Citations in 2017 to items published in } \mathbf{2015\ (208) + 2016\ (263)}}{\text{Number of citable items in } \mathbf{2015\ (154) + 2016\ (151)}} = \frac{471}{305}$$

Downloaded on 2019/4/19 上午11:49:14 from Journal Citation Reports (©2019 Clarivate Analytics)

Journal Impact Factor contributing items

Citable items in 2016 and 2015 (305)

TITLE	CITATIONS COUNTED TOWARDS JIF
Progressive increase of T1 signal intensity in the dentate nucleus and globus pallidus on unenhanced T1-weighted MR images in the pediatric brain exposed to multiple doses of gadolinium contrast<br>By: Roberts, Donna R.; Holden, Kenton R.<br>**Volume: 38 Page: 331-336 Accession number: WOS:000371193300010**<br>**Document Type: Article**	16
Mutations in alpha- and beta-tubulin encoding genes: Implications in brain malformations<br>By: Romaniello, Romina; Arrigoni, Filippo; Bassi, Maria Teresa; Borgatti, Renato<br>**Volume: 37 Page: 273-280 Accession number: WOS:000350094300001**<br>**Document Type: Review**	14
Efficacy of antiepileptic drugs for the treatment of Dravet syndrome with different genotypes<br>By: Shi, Xiu-Yu; Tomonoh, Yuko; Wang, Wen-Ze; Ishii, Atsushi; Higurashi, Norimichi; et al.<br>**Volume: 38 Page: 40-46 Accession number: WOS:000367859900007**<br>**Document Type: Article**	10
High prevalence of genetic alterations in early-onset epileptic encephalopathies associated with infantile movement disorders<br>By: Kobayashi, Yu; Saitsu, Hirotomo; Matsumoto, Naomichi; Tohyama, Jun; Kato, Mitsuhiro; et al.<br>**Volume: 38 Page: 285-292 Accession number: WOS:000371193300004**<br>**Document Type: Article**	10
Retrospective diagnosis of congenital cytomegalovirus infection in children with autism spectrum disorder but no other major neurologic deficit<br>By: Sakamoto, Ayako; Moriuchi, Hiroyuki; Matsuzaki, Junko; Motoyama, Kazunori; Moriuchi, Masako<br>**Volume: 37 Page: 200-205 Accession number: WOS:000349429700003**<br>**Document Type: Article**	9
Circadian-relevant genes are highly polymorphic in autism spectrum disorder patients<br>By: Yang, Zhiliang; Matsumoto, Ayumi; Nakayama, Kazuhiro; Jimbo, Eriko F.; Kojima, Karin; et al.<br>**Volume: 38 Page: 91-99 Accession number: WOS:000367859900014**<br>**Document Type: Article**	8
Dramatic effect of levetiracetam in early-onset epileptic encephalopathy due to STXBP1 mutation<br>By: Dilena, Robertino; Striano, Pasquale; Traverso, Monica; Viri, Maurizio; Cristofori, Gloria; et al.<br>**Volume: 38 Page: 128-131 Accession number: WOS:000367859900021**<br>**Document Type: Article**	7

Downloaded on 2019/4/19 上午11:49:14 from Journal Citation Reports (©2019 Clarivate Analytics)

Citations in 2017 (471)

TITLE	CITATIONS COUNTED TOWARDS JIF
BRAIN & DEVELOPMENT	29
CURRENT PHARMACEUTICAL DESIGN	10
SCIENTIFIC REPORTS	8
PEDIATRIC NEUROLOGY	7
AMERICAN JOURNAL OF MEDICAL GENETICS PART A	6
HUMAN MOLECULAR GENETICS	6
HUMAN MUTATION	6
JOURNAL OF CHILD NEUROLOGY	6
NEUROPEDIATRICS	6
SEIZURE-EUROPEAN JOURNAL OF EPILEPSY	6

Downloaded on 2019/4/19 上午11:49:14 from Journal Citation Reports (©2019 Clarivate Analytics)

Key Indicators 2017

IMPACT METRICS		INFLUENCE METRICS		SOURCE METRICS	
Total Cites	3,855	Eigenfactor Score	0.00500	Citable Items	132
Journal Impact Factor	1.544	Article Influence Score	0.502	% Articles in Citable Items	86.36
5 Year Impact Factor	1.631	Normalized Eigenfactor	0.58200	Average JIF Percentile	18.020
Immediacy Index	0.341			Cited Half-Life	9.5
Impact Factor Without Journal Self Cites	1.449			Citing Half-Life	8.9

Source data

Journal source data 2017

	Articles	Reviews	Combined(C)	Other(O)	Percentage(C/(C+O))
Number in JCR Year 2017 (A)	114	18	132	16	89%
Number of References (B)	2,170	1,027	3,197	60	98%
Ratio (B/A)	19.0	57.1	24.2	3.8	

Downloaded on 2019/4/19 上午11:49:14 from Journal Citation Reports (©2019 Clarivate Analytics)

Box plot

Category Box Plot 2017

Category Box Plot

The category box plot depicts the distribution of Impact Factors for all journals in the category. The horizontal line that forms the top of the box is the 75th percentile (Q1). The horizontal line that forms the bottom is the 25th percentile (Q3). The horizontal line that intersects the box is the median Impact Factor for the category. Horizontal lines above and below the box, called whiskers, represent maximum and minimum values.
The top whisker is the smaller of the following two values:
the maximum Impact Factor (IF)
Q1 IF + 3.5(Q1 IF - Q3 IF)
The bottom whisker is the larger of the following two values:
the minimum Impact Factor (IF)
Q1 IF - 3.5(Q1 IF - Q3 IF)
Box Plots are provided for the current JCR year for each of the categories in which the journal is indexed.

BRAIN DEV-JPN, IF: 1.544

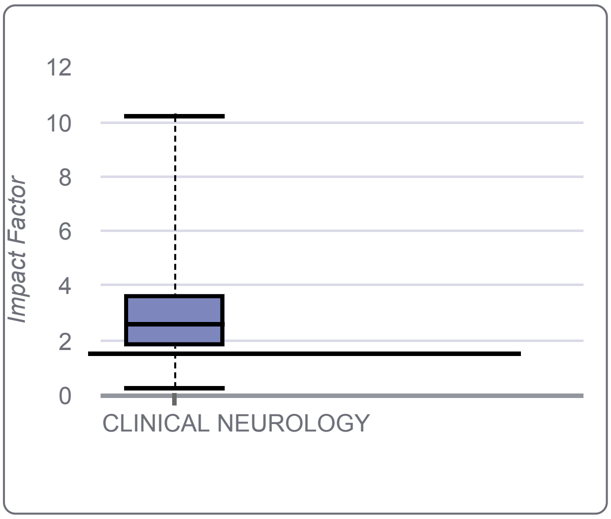

Downloaded on 2019/4/19 上午11:49:14 from Journal Citation Reports (©2019 Clarivate Analytics)

Rank

Rank 2017

JCR Impact Factor

JCR Year	CLINICAL NEUROLOGY		
	Rank	Quartile	JIF Percentile
2017	162/197	Q4	18.020
2016	150/194	Q4	22.938
2015	126/193	Q3	34.974
2014	119/192	Q3	38.281
2013	138/194	Q3	29.124
2012	120/193	Q3	38.083
2011	99/192	Q3	48.698
2010	97/185	Q3	47.838
2009	101/167	Q3	39.820
2008	110/156	Q3	29.808
2007	93/146	Q3	36.644
2006	81/147	Q3	45.238
2005	85/148	Q3	42.905
2004	72/140	Q3	48.929
2003	76/135	Q3	44.074
2002	94/138	Q3	32.246
2001	76/136	Q3	44.485
2000	65/137	Q2	52.920
1999	63/132	Q2	52.652
1998	77/125	Q3	38.800

Downloaded on 2019/4/19 上午11:49:14 from Journal Citation Reports (©2019 Clarivate Analytics)

Downloaded on 2019/4/19 上午11:49:14 from Journal Citation Reports (©2019 Clarivate Analytics)

ESI Total Citations 2017

Rank

JCR Year	NEUROSCIENCE & BEHAVIOR
2017	169/346-Q2
2016	166/345-Q2
2015	150/344-Q2
2014	150/337-Q2
2013	144/339-Q2

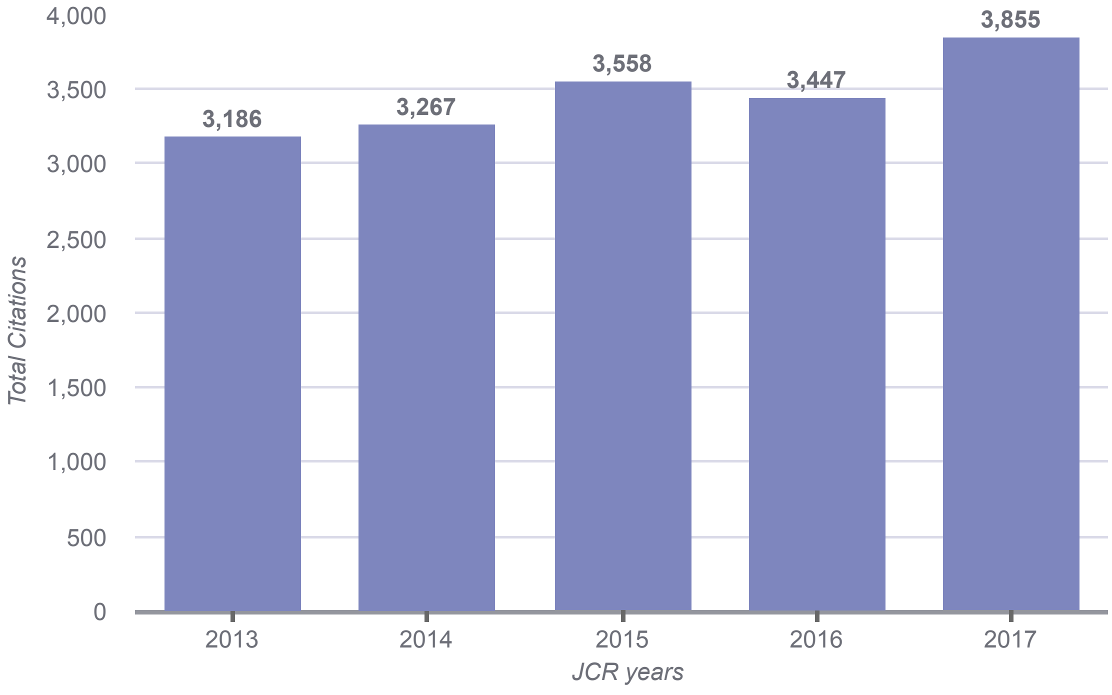

Downloaded on 2019/4/19 上午11:49:14 from Journal Citation Reports (©2019 Clarivate Analytics)

Cited Journal Data

Cited Half-Life Data

Customize columns

Cited Year	2017	2016	2015	2014	2013	2012	2011	2010	2009	2008	20
#Cites from 2017	45	263	208	247	250	208	157	208	269	139	
Cumulative %	1.17%	7.99%	13.39%	19.79%	26.28%	31.67%	35.75%	41.14%	48.12%	51.73%	10

Cited Journal Graph 2017

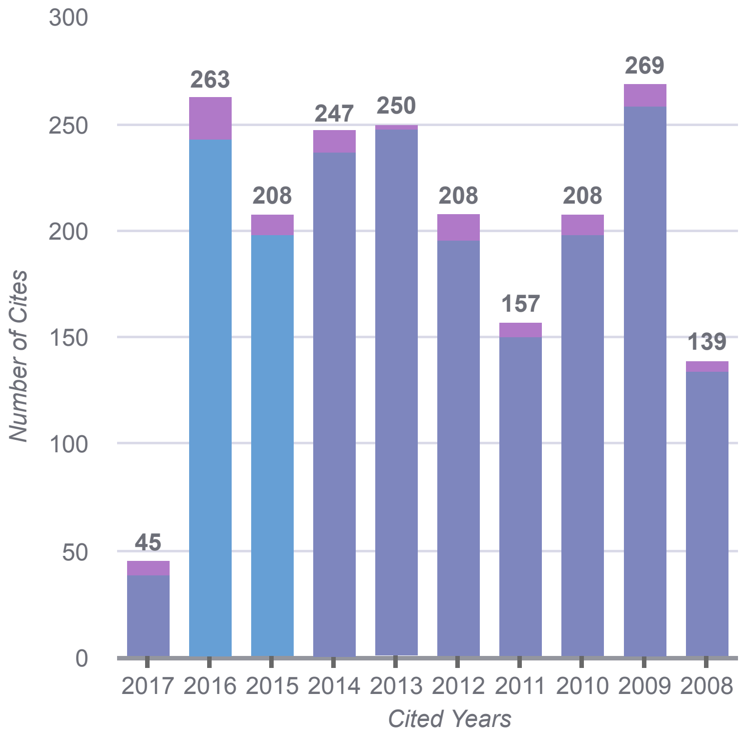

CITED JOURNAL GRAPH

The Cited Journal Graph shows the distribution (by cited year) of citations published in journals during the JCR year to items published in the Journal during the last 10 years.

The white/grey division indicates the cited half-life (if < 10.0). Half of the citations are to items that were published more recently than the cited half-life.

The two light-blue columns indicate citations used to calculate the Impact Factor (always the 2nd and 3rd columns).

Downloaded on 2019/4/19 上午11:49:14 from Journal Citation Reports (©2019 Clarivate Analytics)

Cited Journal Data

Customize columns

	Impact	Citing Journal	All Yrs	2017	2016	2015	2014	2013	2012	2011	2010	2009	2008	R
		ALL Journals	3,855	45	263	208	247	250	208	157	208	269	139	1,
		ALL OTHERS (600)	600	3	29	30	40	43	31	19	30	33	21	
1	1.544	BRAIN DEV-JPN	123	7	20	9	10	2	12	7	9	10	5	
2	2.398	PEDIATR NEUROL	65	0	4	3	1	6	8	4	3	4	2	
3	4.122	SCI REP-UK	55	1	6	2	2	1	0	3	1	5	2	
4	2.600	EPILEPSY BEHAV	51	0	2	1	2	3	0	0	8	5	2	
5	1.665	J CHILD NEUROL	46	0	2	4	1	1	5	3	4	3	0	
6	2.839	SEIZURE-EUR J EPILEP	46	0	3	3	2	4	2	2	3	3	1	
7	2.766	PLOS ONE	42	1	4	0	1	4	2	3	1	5	2	
8	2.757	CURR PHARM DESIGN	40	4	8	2	4	2	3	0	3	2	2	
9	5.067	EPILEPSIA	39	0	4	1	3	4	2	1	0	3	3	
10	1.878	SEMIN PEDIATR NEUROL	34	2	1	2	2	4	3	2	5	1	0	
11	3.508	FRONT NEUROL	30	1	1	2	0	2	0	4	2	2	2	
12	0.751	ACTA MEDICA MEDITERR	28	0	0	0	0	0	11	0	0	0	0	

Rows 1 - 14 of 530 *(use csv export to download the full table)*

Downloaded on 2019/4/19 上午11:49:14 from Journal Citation Reports (©2019 Clarivate Analytics)

Citing Journal Data

Citing Half-Life Data

Customize columns

Citing Year	2017	2016	2015	2014	2013	2012	2011	2010	2009	2008	20
#Cites from 2017	11	171	261	241	222	196	191	197	149	129	
Cumulative %	0.34%	5.59%	13.60%	21.00%	27.82%	33.83%	39.70%	45.75%	50.32%	54.28%	10

Citing Journal Graph 2017

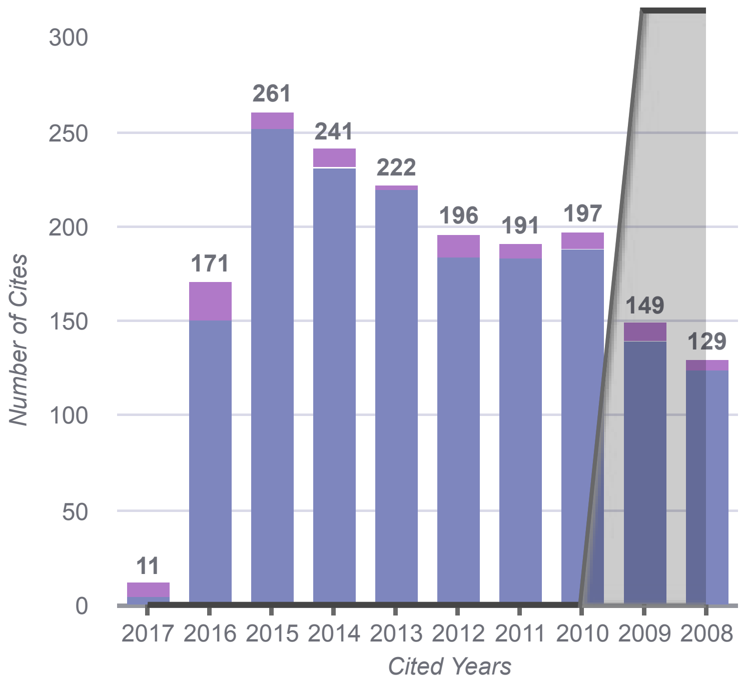

CITING JOURNAL GRAPH

The Citing Journal Graph shows the distribution (by cited year) of citations published in the Journal during the JCR year to items published in journals during the last 10 years.

The white/grey division indicates the citing half-life (if < 10.0). Half of the citations are to items that were published more recently than the citing half-life.

Downloaded on 2019/4/19 上午11:49:14 from Journal Citation Reports (©2019 Clarivate Analytics)

Citing Journal Data

Customize columns

	Impact	Cited Journal	All Yrs	2017	2016	2015	2014	2013	2012	2011	2010	2009	2008	R
		ALL Journals	3,257	11	171	261	241	222	196	191	197	149	129	1,
		ALL OTHERS (670)	670	0	42	58	64	48	35	25	20	30	18	
1	5.067	EPILEPSIA	129	0	5	9	8	6	7	8	16	9	5	
2	1.544	BRAIN DEV-JPN	123	7	20	9	10	2	12	7	9	10	5	
3	8.055	NEUROLOGY	102	1	4	6	6	3	8	8	7	5	2	
4	2.398	PEDIATR NEUROL	48	0	5	3	4	4	4	0	3	5	0	
5	5.515	PEDIATRICS	43	0	1	5	4	1	1	6	3	2	1	
6	10.250	ANN NEUROL	41	0	2	2	7	2	2	2	2	3	3	
7	10.848	BRAIN	40	0	0	3	2	1	4	1	3	1	2	
8	2.491	EPILEPSY RES	38	0	1	6	2	4	6	0	3	2	1	
9	3.289	DEV MED CHILD NEUROL	37	0	1	2	0	6	3	3	2	1	3	
10	1.665	J CHILD NEUROL	37	0	1	4	2	2	2	4	5	3	1	
11	2.264	AM J MED GENET A	35	1	3	4	2	5	2	3	6	1	0	
12	2.766	PLOS ONE	35	0	3	4	9	8	5	1	3	2	0	
13	8.855	AM J HUM GENET	29	0	0	1	0	2	2	1	0	3	0	
14	5.426	NEUROIMAGE	29	0	0	1	2	2	3	1	1	2	1	

Rows 1 - 16 of 372 *(use csv export to download the full table)*

Downloaded on 2019/4/19 上午11:49:14 from Journal Citation Reports (©2019 Clarivate Analytics)

Metric trend

Metric Trend

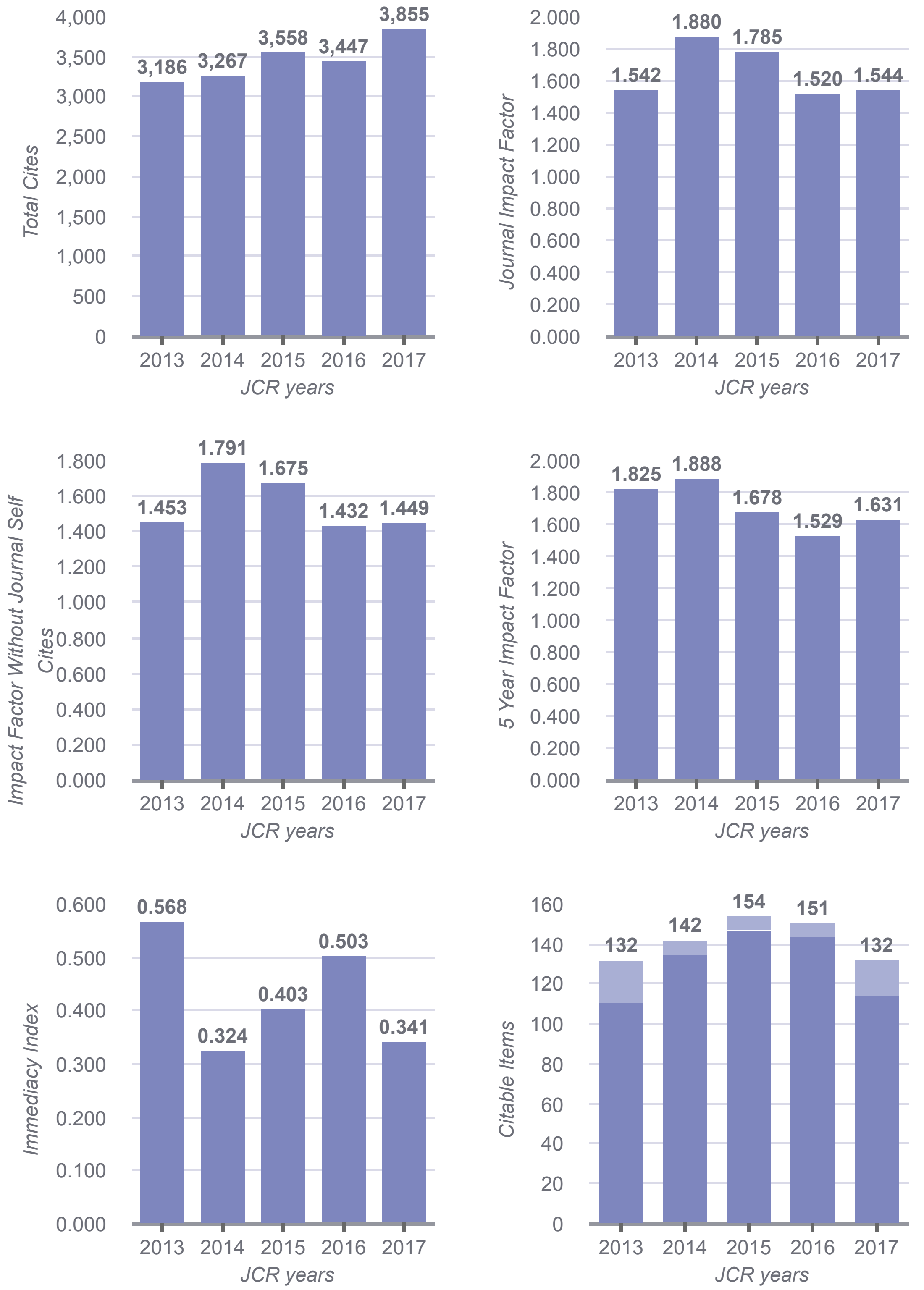

Downloaded on 2019/4/19 上午11:49:14 from Journal Citation Reports (©2019 Clarivate Analytics)

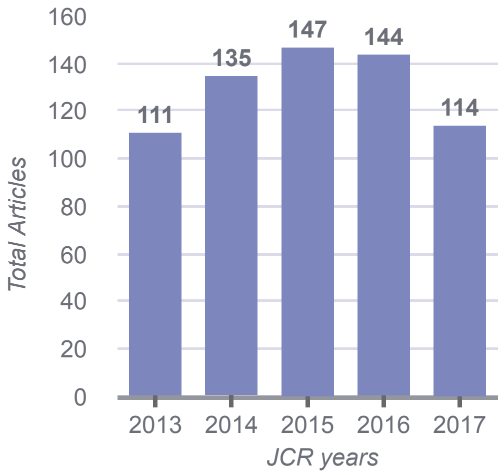

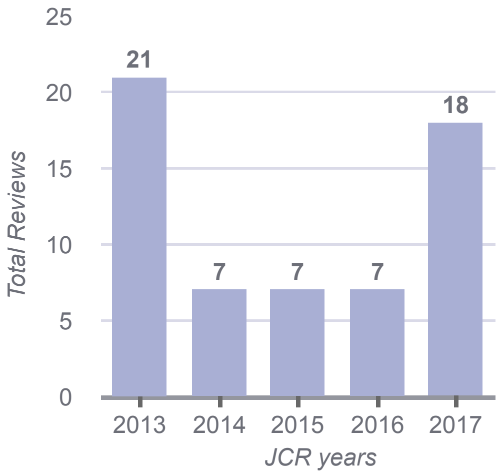

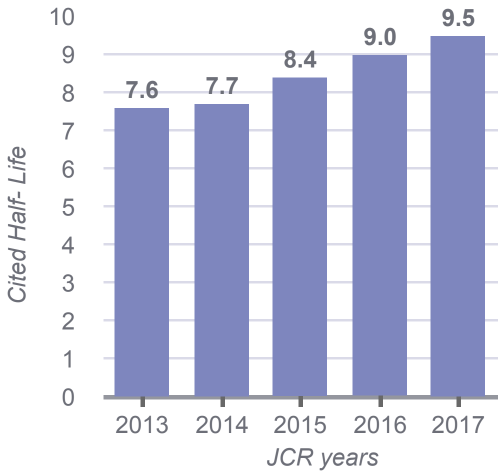

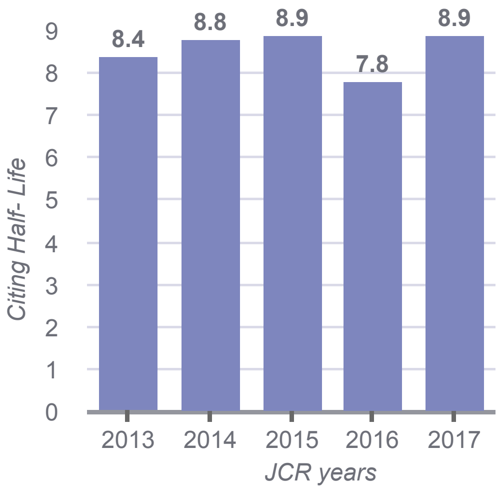

Downloaded on 2019/4/19 上午11:49:14 from Journal Citation Reports (©2019 Clarivate Analytics)

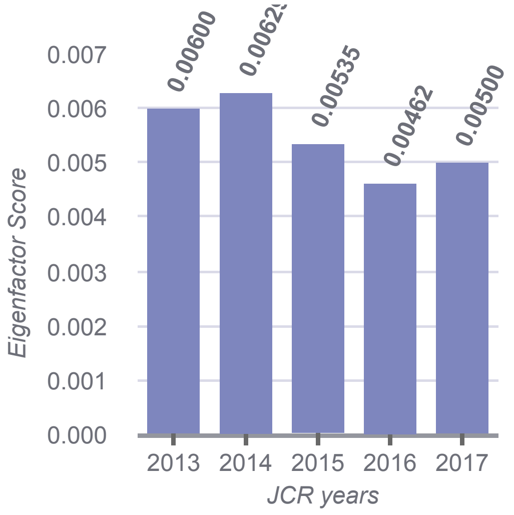

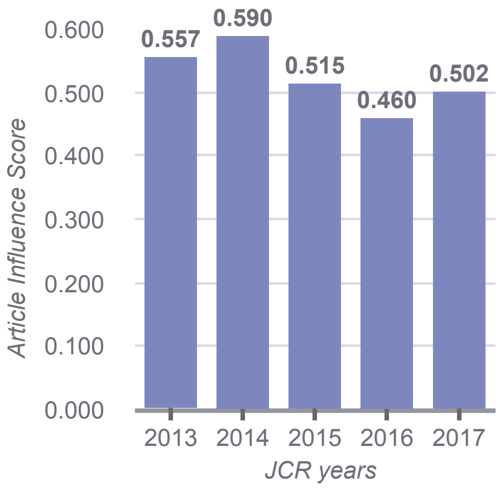

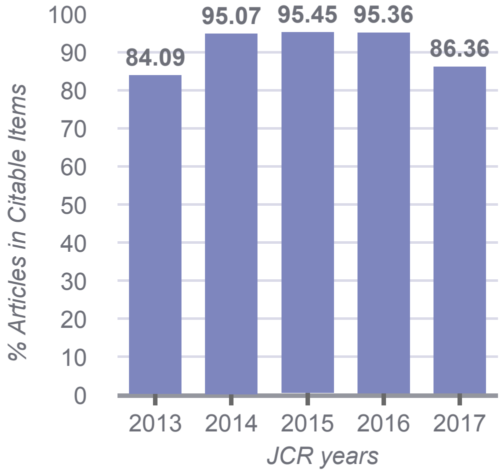

Downloaded on 2019/4/19 上午11:49:14 from Journal Citation Reports (©2019 Clarivate Analytics)

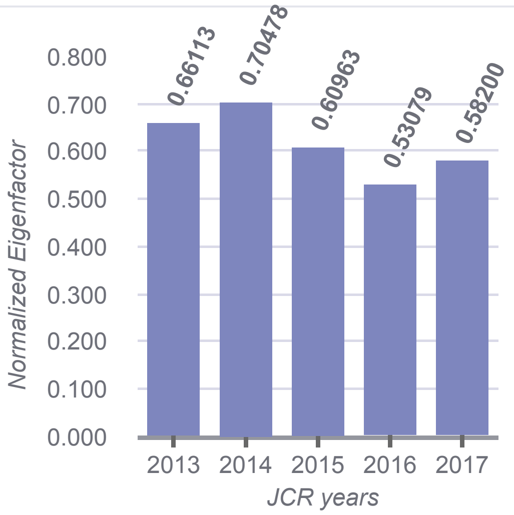

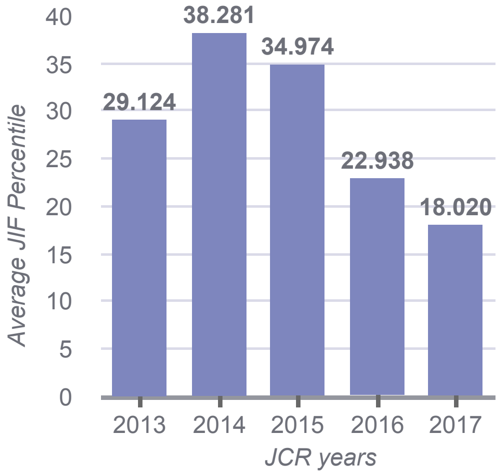

Downloaded on 2019/4/19 上午11:49:14 from Journal Citation Reports (©2019 Clarivate Analytics)

These data summarize the characteristics of the journal's published content for the most recent three years, that is, 2017 and the two prior years, combined. This information is based on all listed authors and addresses. It is meant to be descriptive rather than comparative.

Contributions by country/region

country	count
1. Japan	270
2. CHINA MAINLAND	38
3. Turkey	30
4. Italy	26
5. USA	25
6. South Korea	19
7. India	11
8. Canada	8
- France	8
10. GERMANY (FED REP GER)	7
- Tunisia	7
- Taiwan	7

Contributions by organizations

organization	count
1. TOKYO WOMEN'S MEDICAL UNIVERSITY	34
2. NATIONAL CENTER FOR NEUROLOGY & PSYCHIATRY - JAPAN	30
3. YOKOHAMA CITY UNIVERSITY	24
4. TOTTORI UNIVERSITY	22
5. KOBE UNIVERSITY	21
6. TOHOKU UNIVERSITY	20
7. OKAYAMA UNIVERSITY	18
8. JUNTENDO UNIVERSITY	16
9. JICHI MEDICAL UNIVERSITY	15
- AICHI MEDICAL UNIVERSITY	15

Downloaded on 2019/4/19 上午11:49:14 from Journal Citation Reports (©2019 Clarivate Analytics)
