# Supplementary material for: Emerging trends and knowledge structure of epilepsy during pregnancy research for 2000–2018: a bibliometric analysis
Source: PeerJ. 2019 Jun 7;7:e7115. doi: 10.7717/peerj.7115 (PMC6557303; doi:10.7717/peerj.7115)
Supplement: Supplemental Information 4 [file peerj-07-7115-s004.zip › 7/15. InCites Journal Citation Reports(EPILEPTIC DISORDERS).pdf]

2017 Journal Performance Data for: EPILEPTIC DISORDERS

ISSN: 1294-9361
eISSN: 1950-6945
JOHN LIBBEY EUROTEXT LTD
127 AVE DE LA REPUBLIQUE, 92120 MONTROUGE, FRANCE
FRANCE

TITLES
ISO: Epileptic Disord.
JCR Abbrev: EPILEPTIC DISORD

LANGUAGES
English

CATEGORIES
CLINICAL NEUROLOGY - SCIE

PUBLICATION FREQUENCY
4 issues/year

Downloaded on 2019/4/19 上午11:50:20 from Journal Citation Reports (©2019 Clarivate Analytics)

Current Year

The data in the two graphs below and in the Journal Impact Factor calculation panels represent citation activity in 2017 to items published in the journal in the prior two years. They detail the components of the Journal Impact Factor. Use the "All Years" tab to access key metrics and additional data for the current year and all prior years for this journal.

2017 Journal Impact Factor & percentile rank in category for: EPILEPTIC DISORDERS

1.500
2017 Journal Impact Factor

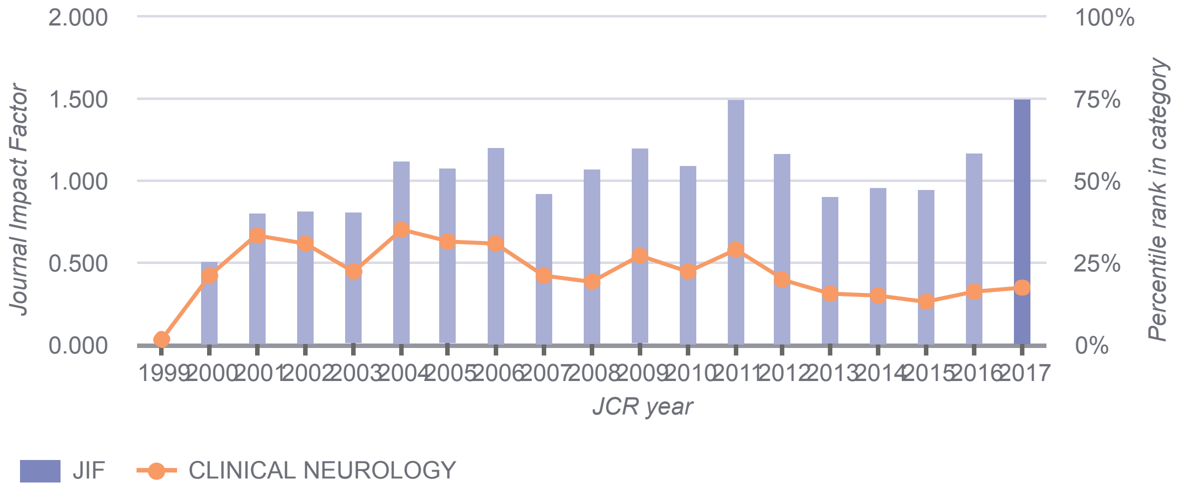

2017 JIF Citation Distribution for: EPILEPTIC DISORDERS

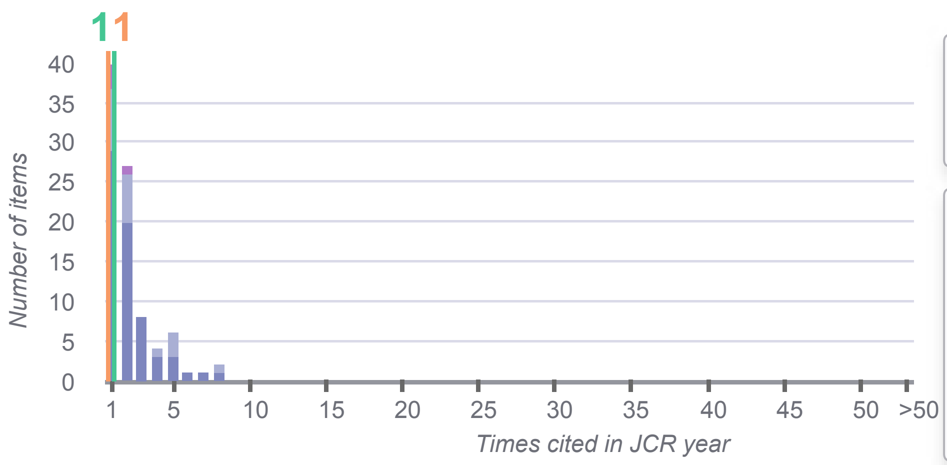

17
Unlinked citations

0
Times Cited

- **51** articles
- **4** reviews
- **8** other

Downloaded on 2019/4/19 上午11:50:20 from Journal Citation Reports (©2019 Clarivate Analytics)

Journal Impact Factor Calculation

$$2017\ \text{Journal Impact Factor} = \frac{210}{140} = 1.500$$

How is Journal Impact Factor Calculated?

$$\text{JIF} = \frac{\text{Citations in 2017 to items published in } \mathbf{2015\ (96) + 2016\ (114)}}{\text{Number of citable items in } \mathbf{2015\ (61) + 2016\ (79)}} = \frac{210}{140}$$

Downloaded on 2019/4/19 上午11:50:20 from Journal Citation Reports (©2019 Clarivate Analytics)

Journal Impact Factor contributing items

Citable items in 2016 and 2015 (140)

TITLE	CITATIONS COUNTED TOWARDS JIF
Incidence, prevalence and aetiology of seizures and epilepsy in children<br>By: Camfield, Peter; Camfield, Carol<br>**Volume: 17 Page: 117-123 Accession number: WOS:000356804800002**<br>**Document Type: Article**	8
From here to epilepsy: the risk of seizure in patients with Alzheimer's disease<br>By: Nicastro, Nicolas; Assal, Frederic; Seeck, Margitta<br>**Volume: 18 Page: 1-12 Accession number: WOS:000371816200001**<br>**Document Type: Review**	8
Vagus nerve stimulation in children with drug-resistant epilepsy: age at implantation and shorter duration of epilepsy as predictors of better efficacy?<br>By: Lagae, Lieven; Verstrepen, An; Nada, Ayman; van Loon, Johan; Theys, Tom; et al.<br>**Volume: 17 Page: 308-314 Accession number: WOS:000362348900011**<br>**Document Type: Article**	7
Efficacy and safety of lacosamide as an adjunctive therapy for refractory focal epilepsy in paediatric patients: a retrospective single-centre study<br>By: Toupin, Jean-Francois; Carmant, Lionel; Birca, Ala; Lortie, Anne; Major, Philippe; et al.<br>**Volume: 17 Page: 436-443 Accession number: WOS:000368935100009**<br>**Document Type: Article**	6
Epilepsy-associated tumours: what epileptologists should know about neuropathology, terminology, and classification systems<br>By: Holthausen, Hans; Bluemcke, Ingmar<br>**Volume: 18 Page: 240-251 Accession number: WOS:000384078800003**<br>**Document Type: Article**	5
Drug-resistant parietal epilepsy: polymorphic ictal semiology does not preclude good post-surgical outcome<br>By: Francione, Stefano; Cossu, Massimo; Lo Russo, Giorgio; Liava, Alexandra; Mai, Roberto; et al.<br>**Volume: 17 Page: 32-46 Accession number: WOS:000353029700004**<br>**Document Type: Article**	5
Lafora disease<br>By: Turnbull, Julie; Tiberia, Erica; Striano, Pasquale; Genton, Pierre; Carpenter, Stirling; et al.<br>**Volume: 18 Page: S38-S62 Accession number: WOS:000405632300005**<br>**Document Type: Review**	5

Downloaded on 2019/4/19 上午11:50:20 from Journal Citation Reports (©2019 Clarivate Analytics)

Citations in 2017 (210)

TITLE	CITATIONS COUNTED TOWARDS JIF
EPILEPSY & BEHAVIOR	16
EPILEPTIC DISORDERS	16
SEIZURE-EUROPEAN JOURNAL OF EPILEPSY	15
EPILEPSIA	11
SEMINARS IN PEDIATRIC NEUROLOGY	11
CURRENT PHARMACEUTICAL DESIGN	10
AMERICAN JOURNAL OF MEDICAL GENETICS PART A	5
CLINICAL NEUROPHYSIOLOGY	4
JOURNAL OF CLINICAL NEUROPHYSIOLOGY	4
REVISTA DE NEUROLOGIA	4

Downloaded on 2019/4/19 上午11:50:20 from Journal Citation Reports (©2019 Clarivate Analytics)

Key Indicators 2017

IMPACT METRICS		INFLUENCE METRICS		SOURCE METRICS	
Total Cites	1,207	Eigenfactor Score	0.00200	Citable Items	55
Journal Impact Factor	1.500	Article Influence Score	0.418	% Articles in Citable Items	94.55
5 Year Impact Factor	1.471	Normalized Eigenfactor	0.24100	Average JIF Percentile	17.513
Immediacy Index	0.091			Cited Half-Life	6.9
Impact Factor Without Journal Self Cites	1.385			Citing Half-Life	9.0

Source data

Journal source data 2017

	Articles	Reviews	Combined(C)	Other(O)	Percentage(C/(C+O))
**Number in JCR Year 2017 (A)**	52	3	55	4	93%
**Number of References (B)**	1,160	342	1,502	3	99%
**Ratio (B/A)**	22.3	114.0	27.3	0.8	

Downloaded on 2019/4/19 上午11:50:20 from Journal Citation Reports (©2019 Clarivate Analytics)

Box plot

Category Box Plot 2017

Category Box Plot

The category box plot depicts the distribution of Impact Factors for all journals in the category. The horizontal line that forms the top of the box is the 75th percentile (Q1). The horizontal line that forms the bottom is the 25th percentile (Q3). The horizontal line that intersects the box is the median Impact Factor for the category. Horizontal lines above and below the box, called whiskers, represent maximum and minimum values.
The top whisker is the smaller of the following two values:
the maximum Impact Factor (IF)
Q1 IF + 3.5(Q1 IF - Q3 IF)
The bottom whisker is the larger of the following two values:
the minimum Impact Factor (IF)
Q1 IF - 3.5(Q1 IF - Q3 IF)
Box Plots are provided for the current JCR year for each of the categories in which the journal is indexed.

EPILEPTIC DISORD, IF: 1.500

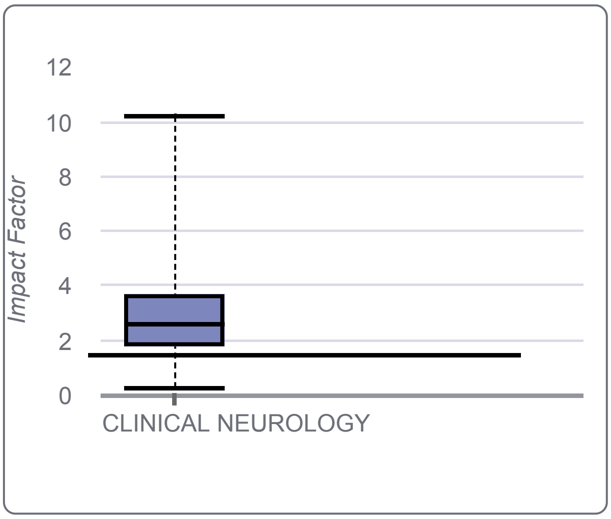

Downloaded on 2019/4/19 上午11:50:20 from Journal Citation Reports (©2019 Clarivate Analytics)

Rank

Rank 2017

JCR Impact Factor

JCR Year	CLINICAL NEUROLOGY		
	Rank	Quartile	JIF Percentile
2017	163/197	Q4	17.513
2016	163/194	Q4	16.237
2015	168/193	Q4	13.212
2014	164/192	Q4	14.844
2013	165/194	Q4	15.206
2012	155/193	Q4	19.948
2011	137/192	Q3	28.906
2010	144/185	Q4	22.432
2009	122/167	Q3	27.246
2008	127/156	Q4	18.910
2007	116/146	Q4	20.890
2006	102/147	Q3	30.952
2005	102/148	Q3	31.419
2004	91/140	Q3	35.357
2003	106/135	Q4	21.852
2002	96/138	Q3	30.797
2001	91/136	Q3	33.456
2000	109/137	Q4	20.803
1999	131/132	Q4	1.136

Downloaded on 2019/4/19 上午11:50:20 from Journal Citation Reports (©2019 Clarivate Analytics)

ESI Total Citations 2017

Rank

JCR Year	NEUROSCIENCE & BEHAVIOR
2017	277/346-Q4
2016	274/345-Q4
2015	279/344-Q4
2014	268/337-Q4
2013	262/339-Q4

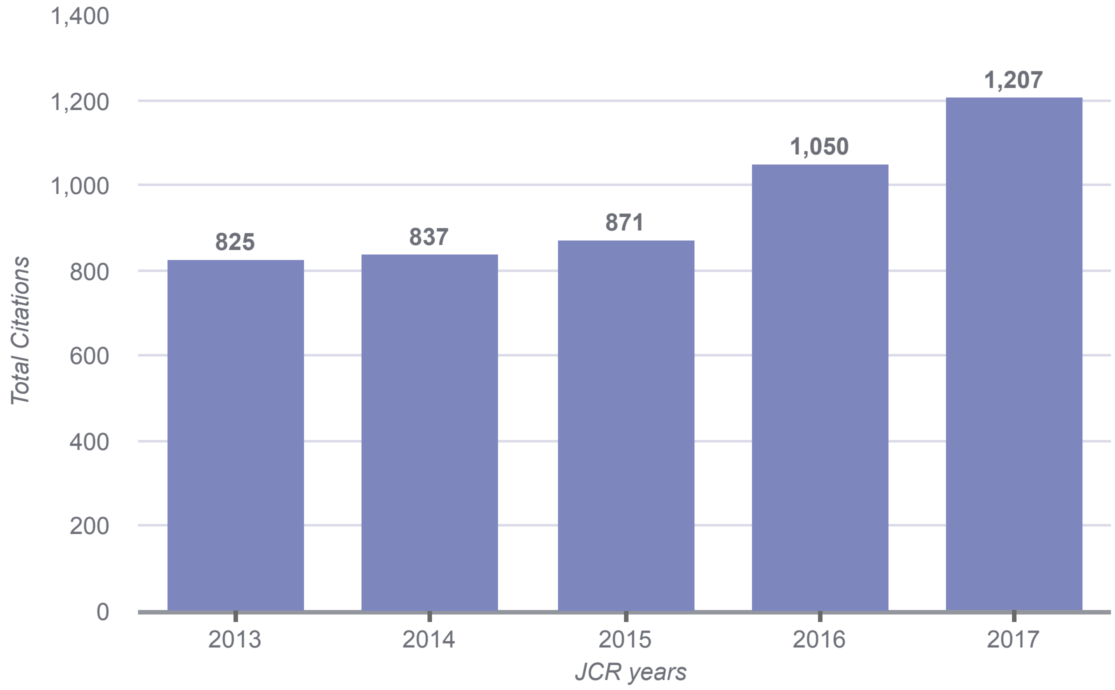

Downloaded on 2019/4/19 上午11:50:20 from Journal Citation Reports (©2019 Clarivate Analytics)

Cited Journal Data

Cited Half-Life Data

Customize columns

Cited Year	2017	2016	2015	2014	2013	2012	2011	2010	2009	2008	20
#Cites from 2017	5	114	96	128	86	104	82	66	66	29	
Cumulative %	0.41%	9.86%	17.81%	28.42%	35.54%	44.16%	50.95%	56.42%	61.89%	64.29%	10

Cited Journal Graph 2017

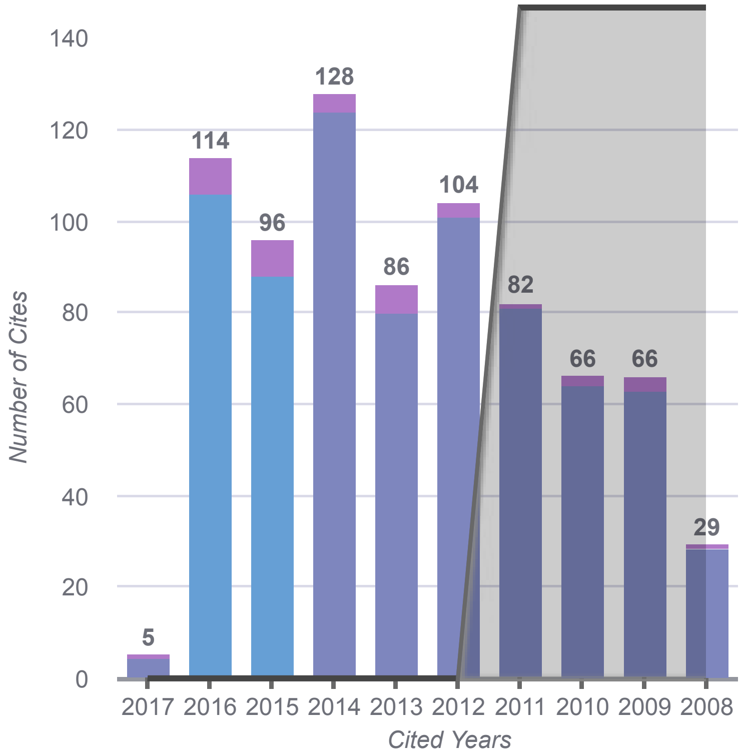

CITED JOURNAL GRAPH

The Cited Journal Graph shows the distribution (by cited year) of citations published in journals during the JCR year to items published in the Journal during the last 10 years.

The white/grey division indicates the cited half-life (if < 10.0). Half of the citations are to items that were published more recently than the cited half-life.

The two light-blue columns indicate citations used to calculate the Impact Factor (always the 2nd and 3rd columns).

Downloaded on 2019/4/19 上午11:50:20 from Journal Citation Reports (©2019 Clarivate Analytics)

Cited Journal Data

Customize columns

	Impact	Citing Journal	All Yrs	2017	2016	2015	2014	2013	2012	2011	2010	2009	2008	R
		ALL Journals	1,207	5	114	96	128	86	104	82	66	66	29	[illegible]
		ALL OTHERS (211)	211	0	18	21	27	14	14	11	16	12	7	
1	2.600	EPILEPSY BEHAV	96	0	10	6	7	7	10	9	2	9	2	
2	5.067	EPILEPSIA	83	0	3	8	7	7	2	0	3	7	1	
3	2.839	SEIZURE-EUR J EPILEP	73	0	9	6	9	5	10	7	1	1	1	
4	1.500	EPILEPTIC DISORD	56	1	8	8	4	6	3	1	2	3	1	
5	2.757	CURR PHARM DESIGN	35	0	6	4	8	4	3	2	1	1	1	
6	2.491	EPILEPSY RES	31	0	2	0	2	2	4	3	2	4	1	
7	3.508	FRONT NEUROL	21	0	0	2	0	2	1	5	1	3	1	
8	1.878	SEMIN PEDIATR NEUROL	17	0	8	3	0	0	0	4	0	0	0	
9	3.614	CLIN NEUROPHYSIOL	16	0	2	2	0	3	2	1	3	0	0	
10	1.982	J CLIN NEUROPHYSIOL	16	0	2	2	2	2	1	0	1	0	1	
11		Z EPILEPTOL	16	0	1	1	2	2	1	1	3	0	0	
12	1.924	WORLD NEUROSURG	15	0	0	1	2	1	2	1	2	2	1	

Rows 1 - 14 of 154 *(use csv export to download the full table)*

Downloaded on 2019/4/19 上午11:50:20 from Journal Citation Reports (©2019 Clarivate Analytics)

Citing Journal Data

Citing Half-Life Data

Customize columns

Citing Year	2017	2016	2015	2014	2013	2012	2011	2010	2009	2008	2[illegible]
#Cites from 2017	20	77	108	89	111	110	89	78	68	57	
Cumulative %	1.33%	6.45%	13.62%	19.53%	26.91%	34.22%	40.13%	45.32%	49.83%	53.62%	1[illegible]

Citing Journal Graph 2017

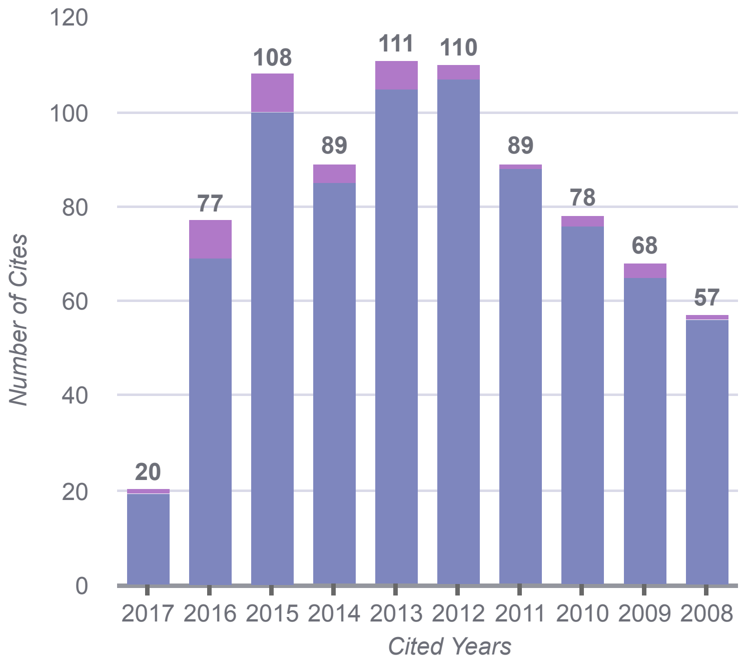

CITING JOURNAL GRAPH

The Citing Journal Graph shows the distribution (by cited year) of citations published in the Journal during the JCR year to items published in journals during the last 10 years.

The white/grey division indicates the citing half-life (if < 10.0). Half of the citations are to items that were published more recently than the citing half-life.

Downloaded on 2019/4/19 上午11:50:20 from Journal Citation Reports (©2019 Clarivate Analytics)

Citing Journal Data

Customize columns

	Impact	Cited Journal	All Yrs	2017	2016	2015	2014	2013	2012	2011	2010	2009	2008	R
		ALL Journals	1,505	20	77	108	89	111	110	89	78	68	57	[illegible]
		ALL OTHERS (255)	255	5	9	24	12	19	26	18	13	8	10	
1	5.067	EPILEPSIA	271	3	14	13	12	19	15	17	17	13	12	[illegible]
2	8.055	NEUROLOGY	116	1	7	6	5	7	6	4	5	2	4	
3	2.600	EPILEPSY BEHAV	61	0	3	9	5	7	7	7	7	3	6	
4	1.500	EPILEPTIC DISORD	56	1	8	8	4	6	3	1	2	3	1	
5	10.848	BRAIN	50	0	2	0	2	3	0	1	3	3	2	
6	2.839	SEIZURE-EUR J EPILEP	48	2	2	7	2	4	5	4	1	5	1	
7	10.250	ANN NEUROL	44	0	0	1	2	0	3	1	0	2	0	
8	2.491	EPILEPSY RES	42	0	0	1	4	4	1	6	4	2	3	
9	1.544	BRAIN DEV-JPN	24	0	0	0	1	0	1	1	0	1	0	
10	1.665	J CHILD NEUROL	19	1	1	1	2	0	1	1	1	1	2	
11	7.144	J NEUROL NEUROSUR PS	19	0	0	0	2	0	2	0	0	0	1	
12	27.144	LANCET NEUROL	19	0	1	1	1	5	2	3	0	1	3	
13	1.982	J CLIN NEUROPHYSIOL	18	0	1	0	0	1	3	0	0	2	0	

Rows 1 - 15 of 133 *(use csv export to download the full table)*

Downloaded on 2019/4/19 上午11:50:20 from Journal Citation Reports (©2019 Clarivate Analytics)

Metric trend

Metric Trend

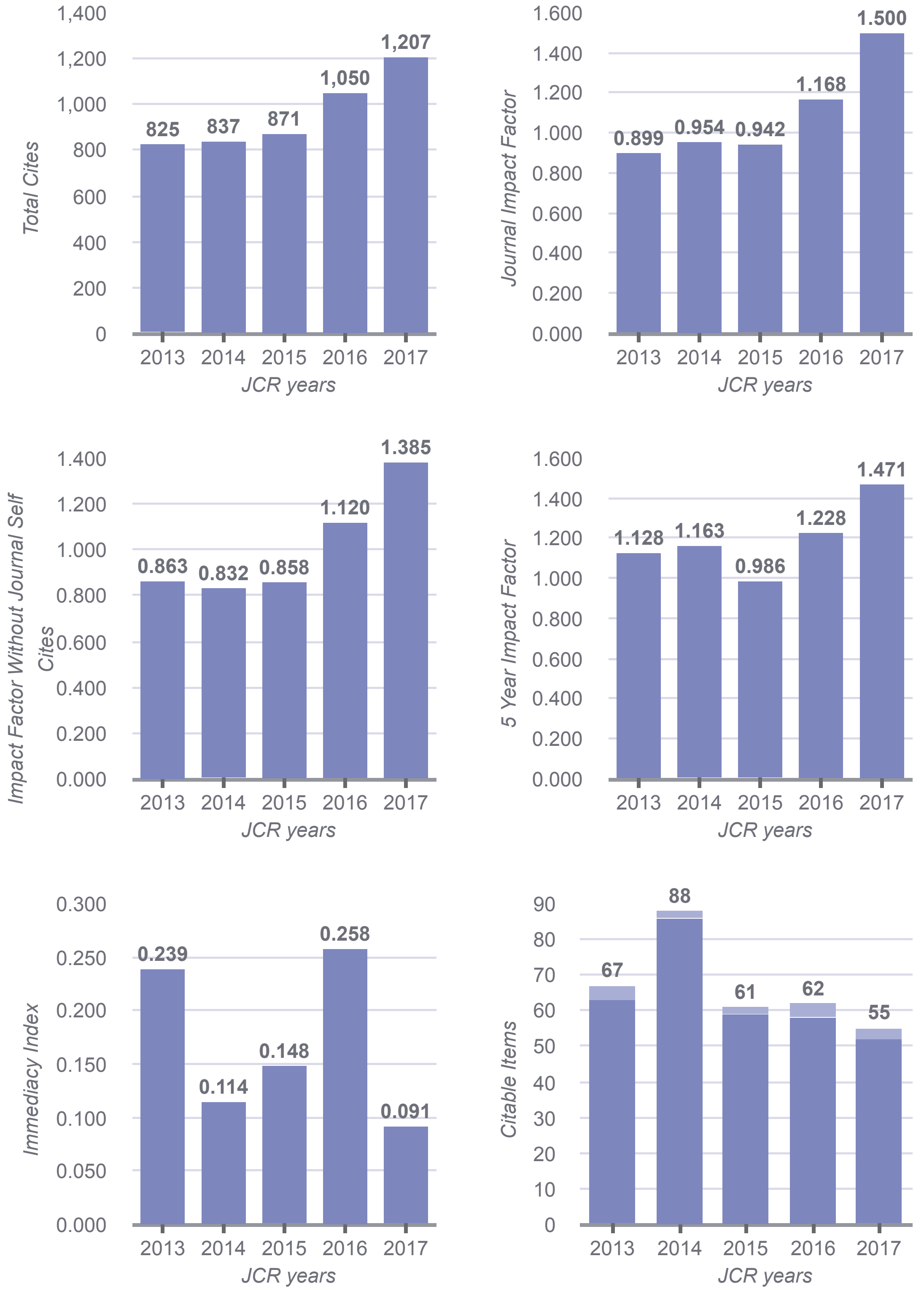

Downloaded on 2019/4/19 上午11:50:20 from Journal Citation Reports (©2019 Clarivate Analytics)

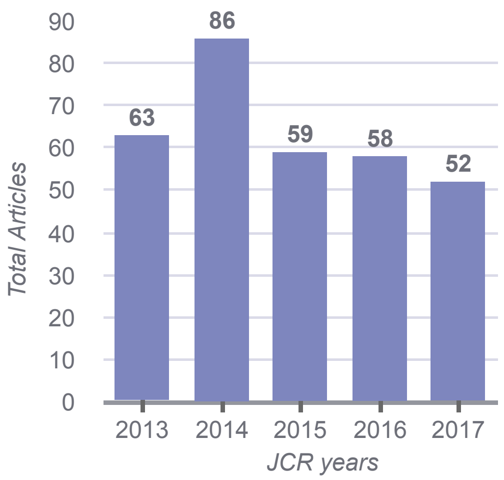

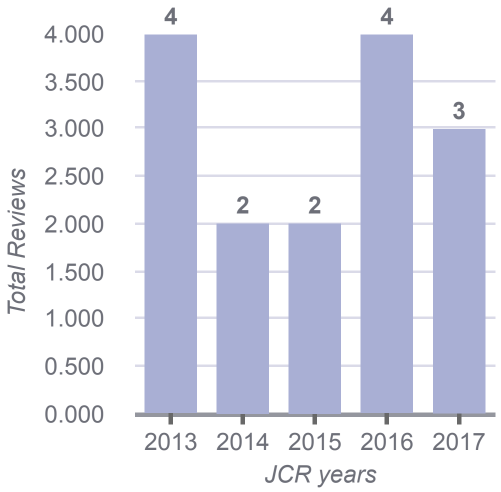

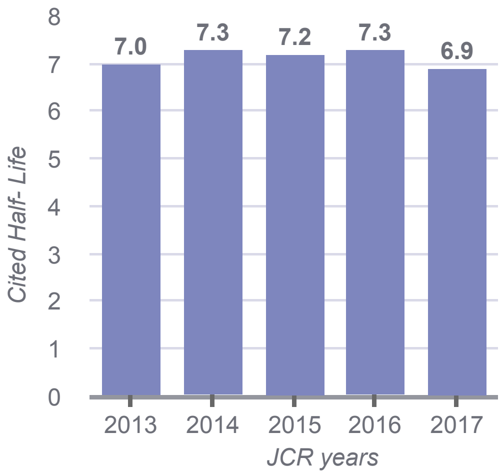

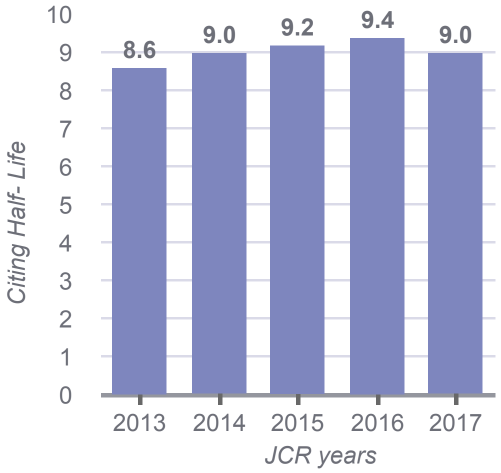

Downloaded on 2019/4/19 上午11:50:20 from Journal Citation Reports (©2019 Clarivate Analytics)

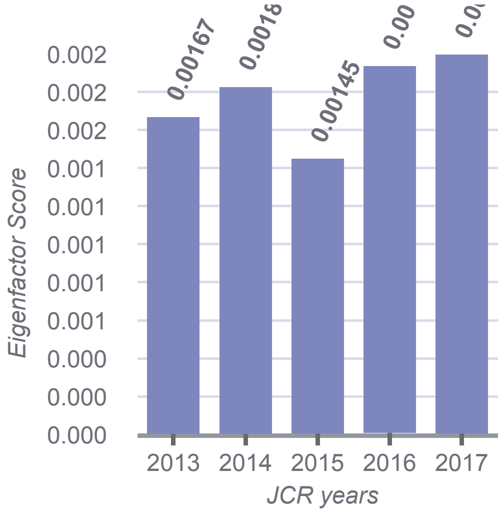

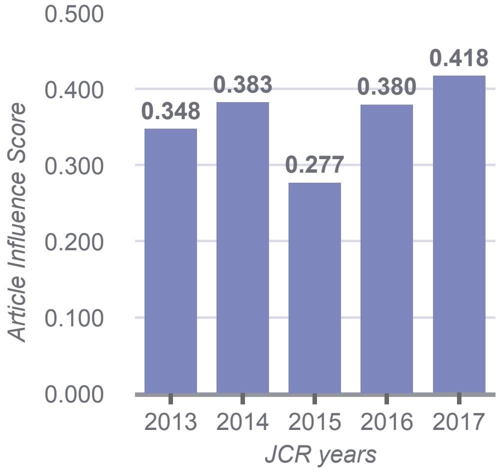

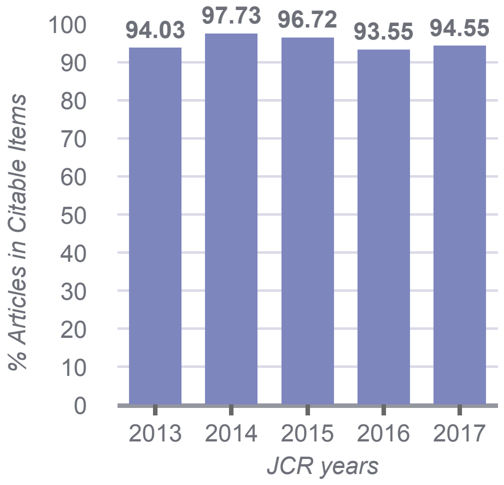

Downloaded on 2019/4/19 上午11:50:20 from Journal Citation Reports (©2019 Clarivate Analytics)

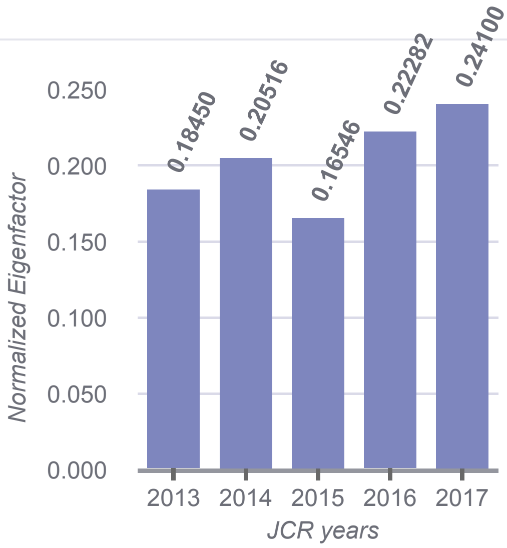

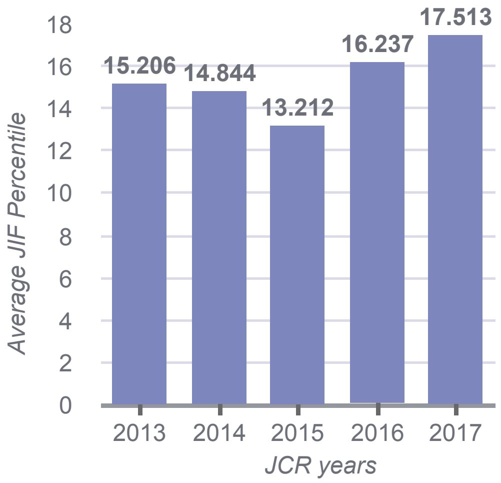

Downloaded on 2019/4/19 上午11:50:20 from Journal Citation Reports (©2019 Clarivate Analytics)

These data summarize the characteristics of the journal's published content for the most recent three years, that is, 2017 and the two prior years, combined. This information is based on all listed authors and addresses. It is meant to be descriptive rather than comparative.

Contributions by country/region

country	count
1. USA	58
2. Italy	38
3. England	28
4. France	27
5. Canada	24
6. Netherlands	19
7. Japan	17
8. GERMANY (FED REP GER)	15
9. Finland	13
10. Turkey	12

Contributions by organizations

organization	count
1. IRCCS ISTITUTO DELLE SCIENZE NEUROLOGICHE DI BOLOGNA (ISNB)	18
2. MAASTRICHT UNIVERSITY	12
3. EPILEPSY CTR KEMPENHAEGHE	10
- INDIANA UNIVERSITY SYSTEM	10
- UNIVERSITY OF CALIFORNIA SYSTEM	10
- JAMES WHITCOMB RILEY HOSP CHILDREN	10
- UNIVERSITY OF TURKU	10
- EAST LONDON NHS FDN TRUST	10
9. INSTITUT NATIONAL DE LA SANTE ET DE LA RECHERCHE MEDICALE (INSERM)	9
10. UNIVERSITY OF LONDON	8
- UNIVERSITY OF TORONTO	8

Downloaded on 2019/4/19 上午11:50:20 from Journal Citation Reports (©2019 Clarivate Analytics)
