# Supplementary material for: Emerging trends and knowledge structure of epilepsy during pregnancy research for 2000–2018: a bibliometric analysis
Source: PeerJ. 2019 Jun 7;7:e7115. doi: 10.7717/peerj.7115 (PMC6557303; doi:10.7717/peerj.7115)
Supplement: Supplemental Information 4 [file peerj-07-7115-s004.zip › 7/2. InCites Journal Citation Reports(PEDIATRICS).pdf]

2017 Journal Performance Data for: PEDIATRICS

ISSN: 0031-4005
eISSN: 1098-4275
AMER ACAD PEDIATRICS
141 NORTH-WEST POINT BLVD, ELK GROVE VILLAGE, IL 60007-1098
USA

TITLES
ISO: Pediatrics
JCR Abbrev: PEDIATRICS

LANGUAGES
English

CATEGORIES
PEDIATRICS - SCIE

PUBLICATION FREQUENCY
12 issues/year

Downloaded on 2019/4/19 上午11:32:06 from Journal Citation Reports (©2019 Clarivate Analytics)

Current Year

The data in the two graphs below and in the Journal Impact Factor calculation panels represent citation activity in 2017 to items published in the journal in the prior two years. They detail the components of the Journal Impact Factor. Use the "All Years" tab to access key metrics and additional data for the current year and all prior years for this journal.

2017 Journal Impact Factor & percentile rank in category for: PEDIATRICS

5.515
2017 Journal Impact Factor

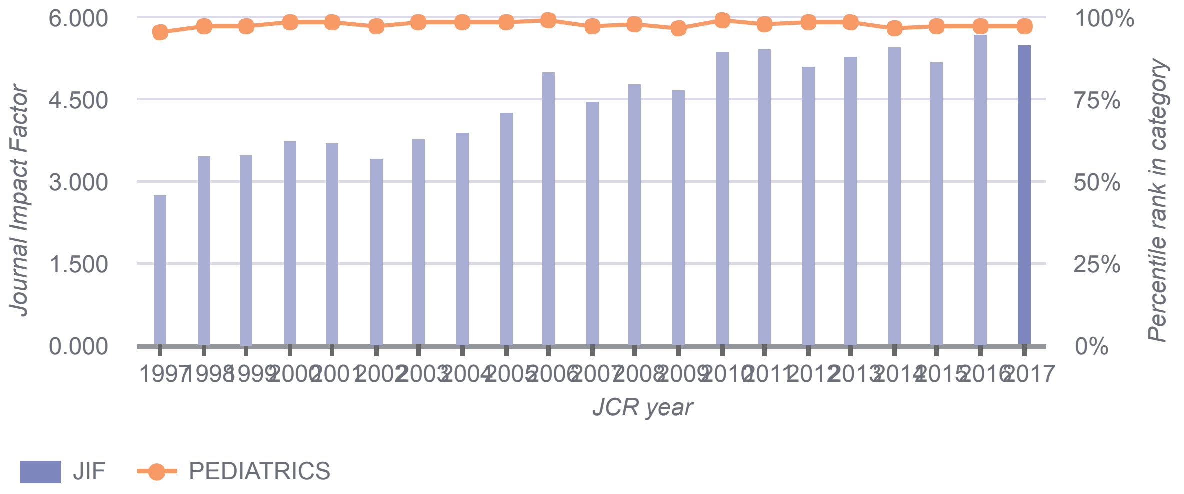

2017 JIF Citation Distribution for: PEDIATRICS

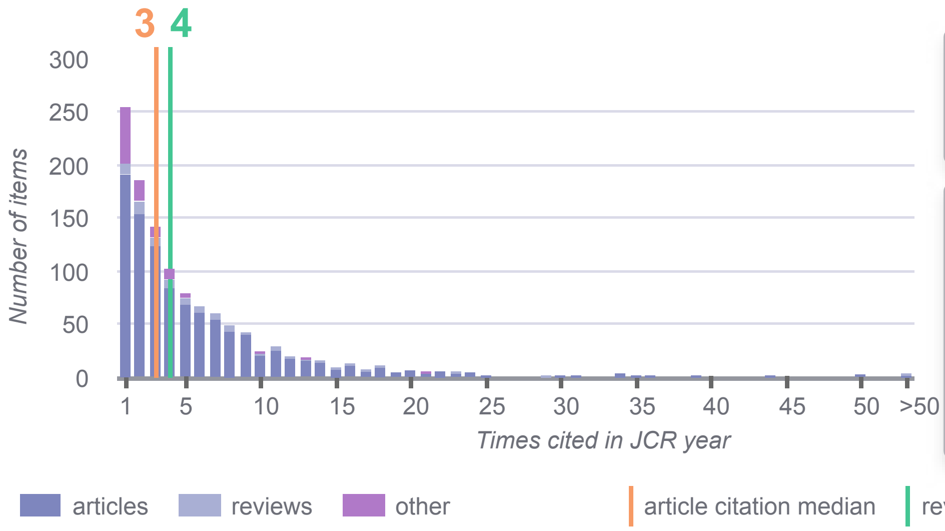

773
Unlinked citations

0
Times Cited

- **250** articles
- **11** reviews
- **225** other

Downloaded on 2019/4/19 上午11:32:06 from Journal Citation Reports (©2019 Clarivate Analytics)

Journal Impact Factor Calculation

$$2017\ \text{Journal Impact Factor} = \frac{7{,}329}{1{,}329} = 5.515$$

How is Journal Impact Factor Calculated?

$$\text{JIF} = \frac{\text{Citations in 2017 to items published in } \mathbf{2015\ (4{,}235) + 2016\ (3{,}094)}}{\text{Number of citable items in } \mathbf{2015\ (616) + 2016\ (713)}} = \frac{7{,}329}{1{,}329}$$

Downloaded on 2019/4/19 上午11:32:06 from Journal Citation Reports (©2019 Clarivate Analytics)

Journal Impact Factor contributing items

Citable items in 2016 and 2015 (1,329)

TITLE	CITATIONS COUNTED TOWARDS JIF
Vascular Anomalies Classification: Recommendations From the International Society for the Study of Vascular Anomalies<br>By: Wassef, Michel; Lopez-Gutierrez, Juan-Carlos; Lord, David J. E.; Mitchel, Sally; Powell, Julie; et al.<br>**Volume: 136 Page: E203-E214 Accession number: WOS:000357296000023**<br>**Document Type: Article**	82
Prevalence of Attention-Deficit/Hyperactivity Disorder: A Systematic Review and Meta-analysis<br>By: Thomas, Rae; Sanders, Sharon; Doust, Jenny; Beller, Elaine; Glasziou, Paul<br>**Volume: 135 Page: E994-E1001 Accession number: WOS:000353726700023**<br>**Document Type: Review**	80
Risk Factors for Exclusive E-Cigarette Use and Dual E-Cigarette Use and Tobacco Use in Adolescents<br>By: Wills, Thomas A.; Knight, Rebecca; Williams, Rebecca J.; Pagano, Ian; Sargent, James D.<br>**Volume: 135 Page: E43-E51 Accession number: WOS:000347172200007**<br>**Document Type: Article**	50
Benefits of Strict Rest After Acute Concussion: A Randomized Controlled Trial<br>By: Thomas, Danny George; Apps, Jennifer N.; Hoffmann, Raymond G.; McCrea, Michael; Hammeke, Thomas<br>**Volume: 135 Page: 213-223 Accession number: WOS:000348666300040**<br>**Document Type: Article**	50
Prevalence of HPV After Introduction of the Vaccination Program in the United States<br>By: Markowitz, Lauri E.; Liu, Gui; Hariri, Susan; Steinau, Martin; Dunne, Eileen F.; et al.<br>**Volume: 137 Accession number: WOS:000371395800016 Document Type: Article**	44
Cognition and Brain Structure Following Early Childhood Surgery With Anesthesia<br>By: Backeljauw, Barynia; Holland, Scott K.; Altaye, Mekibib; Loepke, Andreas W.<br>**Volume: 136 Page: E1-E12 Accession number: WOS:000357296000001**<br>**Document Type: Article**	39
Cesarean Section and Chronic Immune Disorders<br>By: Sevelsted, Astrid; Stokholm, Jakob; Bonnelykke, Klaus; Bisgaard, Hans<br>**Volume: 135 Page: E92-E98 Accession number: WOS:000347172200013**<br>**Document Type: Article**	36

Downloaded on 2019/4/19 上午11:32:06 from Journal Citation Reports (©2019 Clarivate Analytics)

Citations in 2017 (7,329)

TITLE	CITATIONS COUNTED TOWARDS JIF
PEDIATRICS	522
JOURNAL OF PEDIATRICS	171
PLOS ONE	98
JAMA PEDIATRICS	96
JOURNAL OF PERINATOLOGY	89
ACADEMIC PEDIATRICS	85
ACTA PAEDIATRICA	68
BMJ OPEN	59
CURRENT OPINION IN PEDIATRICS	55
SCIENTIFIC REPORTS	53

Downloaded on 2019/4/19 上午11:32:06 from Journal Citation Reports (©2019 Clarivate Analytics)

Key Indicators 2017

IMPACT METRICS		INFLUENCE METRICS		SOURCE METRICS	
**Total Cites**	77,301	**Eigenfactor Score**	0.11900	**Citable Items**	593
**Journal Impact Factor**	5.515	**Article Influence Score**	2.459	**% Articles in Citable Items**	93.25
**5 Year Impact Factor**	6.442	**Normalized Eigenfactor**	13.85200	**Average JIF Percentile**	97.984
**Immediacy Index**	1.022			**Cited Half-Life**	9.3
**Impact Factor Without Journal Self Cites**	5.121			**Citing Half-Life**	6.9

Source data

Journal source data 2017

	Articles	Reviews	Combined(C)	Other(O)	Percentage(C/(C+O))
**Number in JCR Year 2017 (A)**	553	40	593	173	77%
**Number of References (B)**	20,329	2,753	23,082	1,623	93%
**Ratio (B/A)**	36.8	68.8	38.9	9.4	

Downloaded on 2019/4/19 上午11:32:06 from Journal Citation Reports (©2019 Clarivate Analytics)

Box plot

Category Box Plot 2017

Category Box Plot

The category box plot depicts the distribution of Impact Factors for all journals in the category. The horizontal line that forms the top of the box is the 75th percentile (Q1). The horizontal line that forms the bottom is the 25th percentile (Q3). The horizontal line that intersects the box is the median Impact Factor for the category. Horizontal lines above and below the box, called whiskers, represent maximum and minimum values.
The top whisker is the smaller of the following two values:
the maximum Impact Factor (IF)
Q1 IF + 3.5(Q1 IF - Q3 IF)
The bottom whisker is the larger of the following two values:
the minimum Impact Factor (IF)
Q1 IF - 3.5(Q1 IF - Q3 IF)
Box Plots are provided for the current JCR year for each of the categories in which the journal is indexed.

PEDIATRICS, IF: 5.515

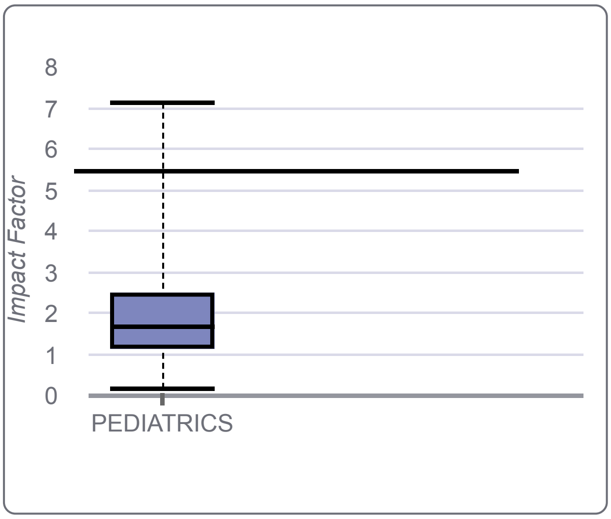

Downloaded on 2019/4/19 上午11:32:06 from Journal Citation Reports (©2019 Clarivate Analytics)

Rank

Rank 2017

JCR Impact Factor

JCR Year	PEDIATRICS		
	Rank	Quartile	JIF Percentile
2017	3/124	Q1	97.984
2016	3/121	Q1	97.934
2015	3/120	Q1	97.917
2014	4/120	Q1	97.083
2013	2/118	Q1	98.729
2012	2/122	Q1	98.770
2011	2/115	Q1	98.696
2010	1/109	Q1	99.541
2009	3/94	Q1	97.340
2008	2/86	Q1	98.256
2007	2/78	Q1	98.077
2006	1/74	Q1	99.324
2005	1/73	Q1	99.315
2004	1/70	Q1	99.286
2003	1/68	Q1	99.265
2002	2/68	Q1	97.794
2001	1/69	Q1	99.275
2000	1/71	Q1	99.296
1999	2/72	Q1	97.917
1998	2/71	Q1	97.887

Downloaded on 2019/4/19 上午11:32:06 from Journal Citation Reports (©2019 Clarivate Analytics)

Downloaded on 2019/4/19 上午11:32:06 from Journal Citation Reports (©2019 Clarivate Analytics)

ESI Total Citations 2017

Rank

JCR Year	CLINICAL MEDICINE
2017	13/2061-Q1
2016	13/2029-Q1
2015	14/2012-Q1
2014	15/1975-Q1
2013	16/1955-Q1

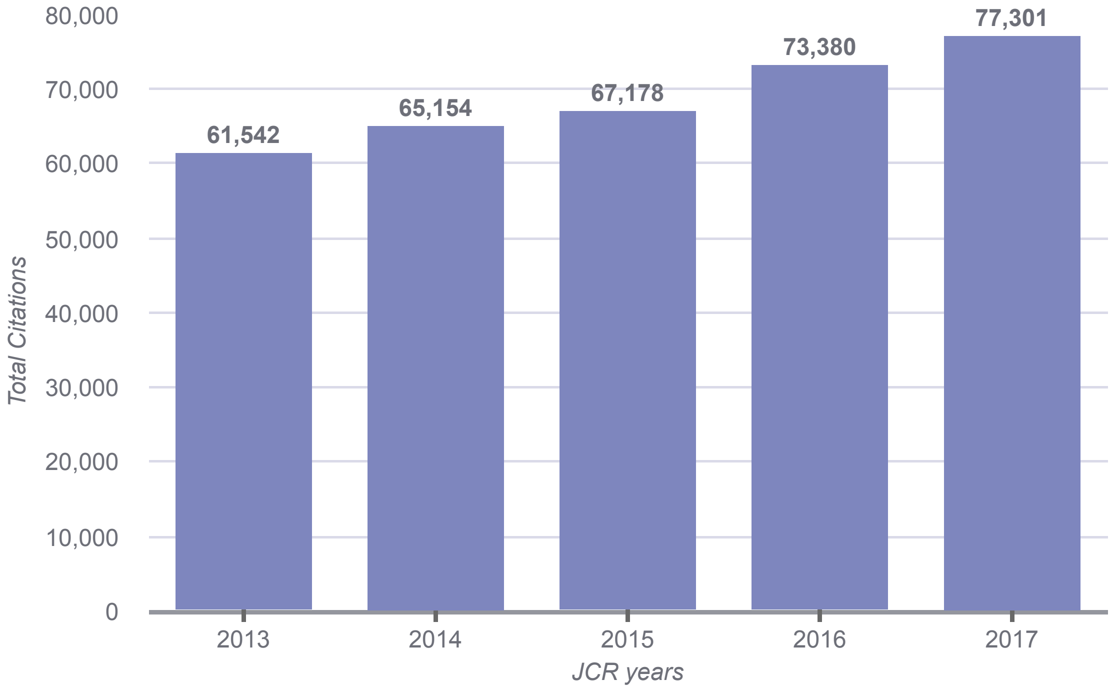

Downloaded on 2019/4/19 上午11:32:06 from Journal Citation Reports (©2019 Clarivate Analytics)

Clarivate Analytics

Cited Journal Data

Cited Half-Life Data

Customize columns

Cited Year	2017	2016	2015	2014	2013	2012	2011	2010	2009	2008	20
#Cites from 2017	606	3,094	4,235	5,095	4,859	5,272	5,003	4,542	4,679	4,143	
Cumulative %	0.78%	4.79%	10.27%	16.86%	23.14%	29.96%	36.43%	42.31%	48.36%	53.72%	10

Cited Journal Graph 2017

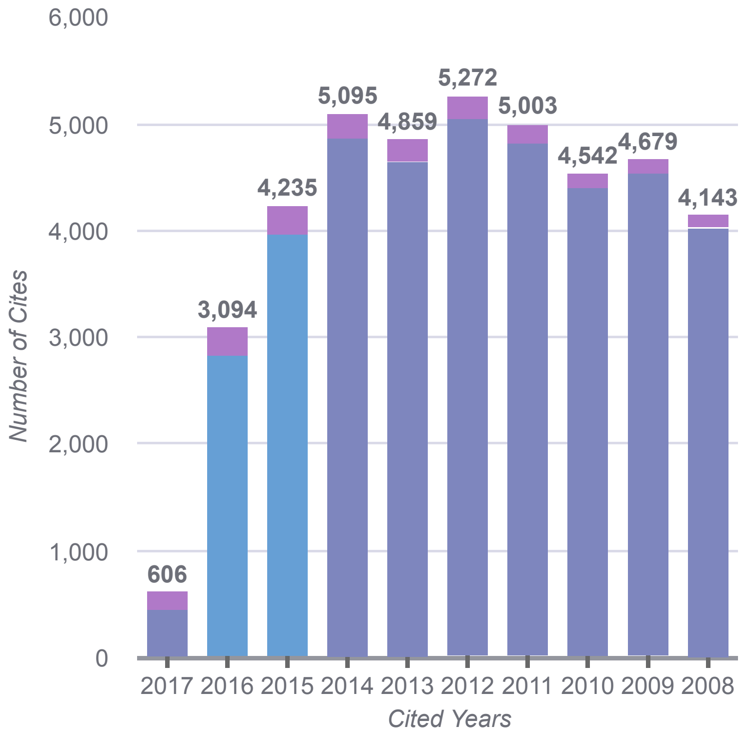

CITED JOURNAL GRAPH

The Cited Journal Graph shows the distribution (by cited year) of citations published in journals during the JCR year to items published in the Journal during the last 10 years.

The white/grey division indicates the cited half-life (if < 10.0). Half of the citations are to items that were published more recently than the cited half-life.

The two light-blue columns indicate citations used to calculate the Impact Factor (always the 2nd and 3rd columns).

Downloaded on 2019/4/19 上午11:32:06 from Journal Citation Reports (©2019 Clarivate Analytics)

Clarivate Analytics

Cited Journal Data

Customize columns

	Impact	Citing Journal	All Yrs	2017	2016	2015	2014	2013	2012	2011	2010	2009	20
		ALL Journals	77,301	606	3,094	4,235	5,095	4,859	5,272	5,003	4,542	4,679	4,
		ALL OTHERS (1657)	1,657	9	38	64	117	67	102	110	91	86	
1	5.515	PEDIATRICS	2,761	174	266	256	222	213	198	173	129	133	
2	3.667	J PEDIATR-US	1,452	13	66	105	94	78	86	111	88	83	
3	2.766	PLOS ONE	1,180	5	36	62	82	66	67	70	79	70	
4	2.183	J PERINATOL	753	8	34	55	38	48	61	53	43	45	
5	6.754	COCHRANE DB SYST REV	666	4	12	21	26	35	31	21	21	32	
6	2.806	ACAD PEDIATR	589	3	42	43	69	46	46	33	31	53	
7	2.580	ACTA PAEDIATR	556	2	34	34	41	44	46	34	36	31	
8	1.409	CLIN PEDIATR	545	7	25	26	34	46	33	40	30	32	
9	4.122	SCI REP-UK	533	3	26	27	29	30	42	30	30	34	
10	2.413	BMJ OPEN	521	4	24	35	40	34	37	30	38	28	
11	3.123	PEDIATR RES	474	1	15	23	25	24	27	28	25	33	
12	10.769	JAMA PEDIATR	464	12	65	31	53	28	43	40	26	23	
13	3.285	VACCINE	451	5	22	30	45	25	19	43	38	17	
14	1.623	AM J PERINAT	447	0	8	27	29	30	36	29	30	32	
15	2.042	BMC PEDIATR	376	0	4	13	15	23	34	27	26	21	

Rows 1 - 17 of 4,157 *(use csv export to download the full table)*

Downloaded on 2019/4/19 上午11:32:06 from Journal Citation Reports (©2019 Clarivate Analytics)

Citing Journal Data

Citing Half-Life Data

Customize columns

Citing Year	2017	2016	2015	2014	2013	2012	2011	2010	2009	2008	20
#Cites from 2017	560	1,866	2,246	2,153	2,017	1,627	1,504	1,331	1,210	1,023	
Cumulative %	2.27%	9.82%	18.91%	27.63%	35.79%	42.38%	48.46%	53.85%	58.75%	62.89%	10

Citing Journal Graph 2017

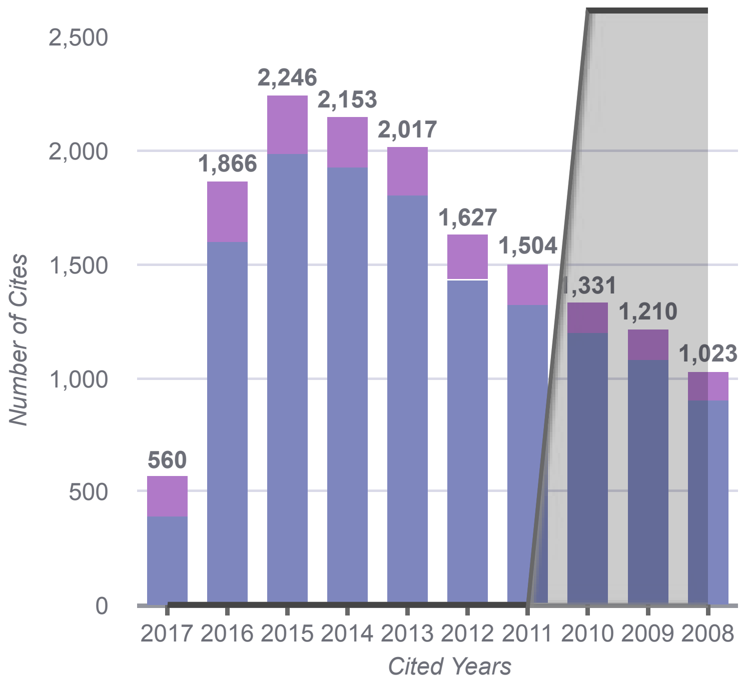

CITING JOURNAL GRAPH

The Citing Journal Graph shows the distribution (by cited year) of citations published in the Journal during the JCR year to items published in journals during the last 10 years.

The white/grey division indicates the citing half-life (if < 10.0). Half of the citations are to items that were published more recently than the citing half-life.

Downloaded on 2019/4/19 上午11:32:06 from Journal Citation Reports (©2019 Clarivate Analytics)

Citing Journal Data

Customize columns

	Impact	Cited Journal	All Yrs	2017	2016	2015	2014	2013	2012	2011	2010	2009	20[illegible]
		ALL Journals	24,705	560	1,866	2,246	2,153	2,017	1,627	1,504	1,331	1,210	1,[illegible]
		ALL OTHERS (3570)	3,570	65	242	303	260	234	205	176	156	129	
1	5.515	PEDIATRICS	2,761	174	266	256	222	213	198	173	129	133	
2	3.667	J PEDIATR-US	461	10	31	46	43	41	23	18	28	25	
3	47.661	JAMA-J AM MED ASSOC	457	2	48	25	36	31	22	17	28	12	
4	79.260	NEW ENGL J MED	417	12	35	45	24	21	18	25	25	16	
5	53.254	LANCET	281	6	22	13	19	30	26	19	7	16	
6	12.888	MMWR-MORBID MORTAL W	229	4	45	35	25	30	9	15	11	7	
7	10.769	JAMA PEDIATR	211	8	54	59	46	44	0	0	0	0	
8		ARCH PEDIAT ADOL MED	207	0	0	0	0	0	18	18	20	20	
9	23.562	BMJ-BRIT MED J	206	0	9	15	7	12	18	12	12	26	
10	2.766	PLOS ONE	196	1	17	39	46	45	23	14	5	3	
11	3.258	ARCH DIS CHILD	184	0	9	11	7	4	10	11	7	6	
12	2.806	ACAD PEDIATR	163	7	36	17	28	23	15	13	14	9	
13	6.754	COCHRANE DB SYST REV	162	0	15	19	21	26	21	16	8	7	

Rows 1 - 15 of 1,821 *(use csv export to download the full table)*

Downloaded on 2019/4/19 上午11:32:06 from Journal Citation Reports (©2019 Clarivate Analytics)

Metric trend

Metric Trend

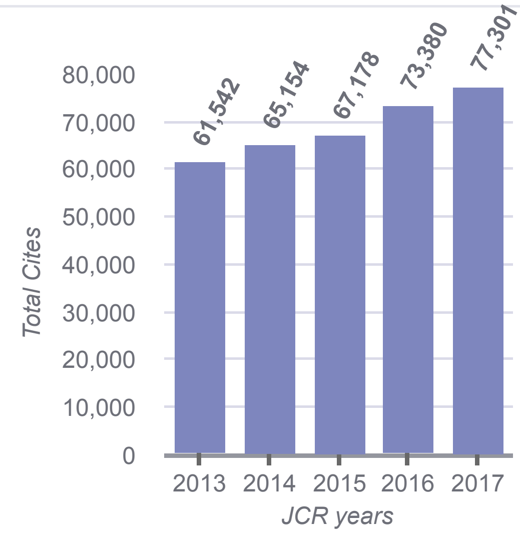

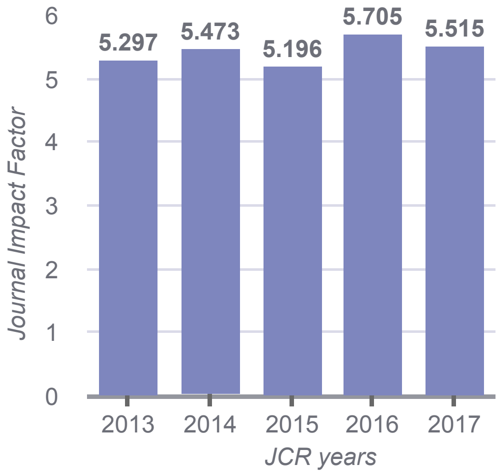

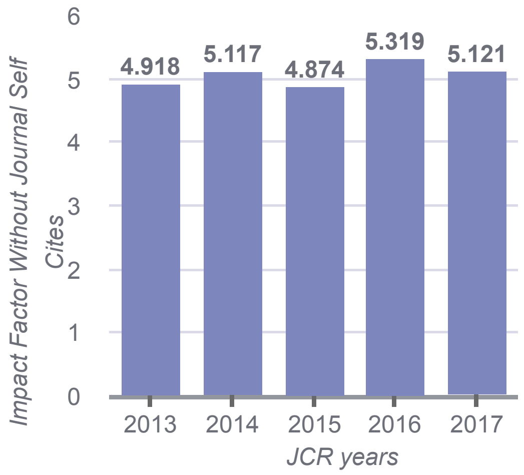

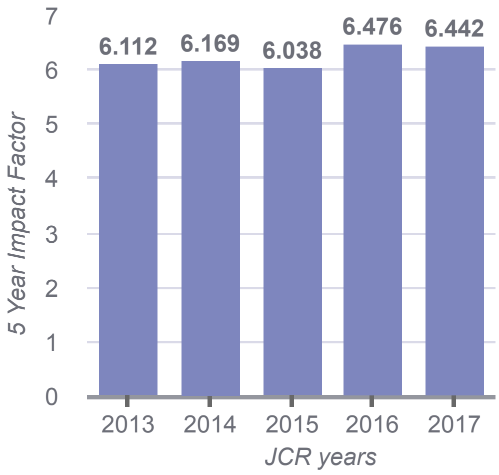

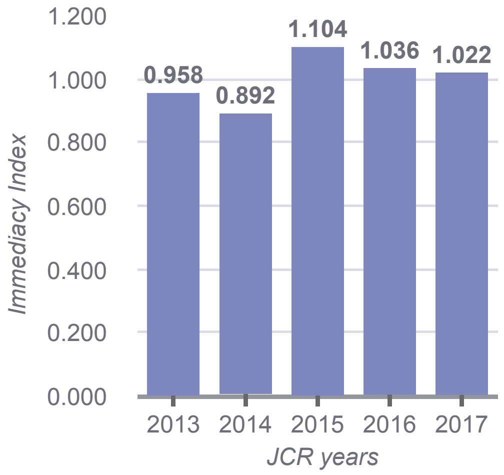

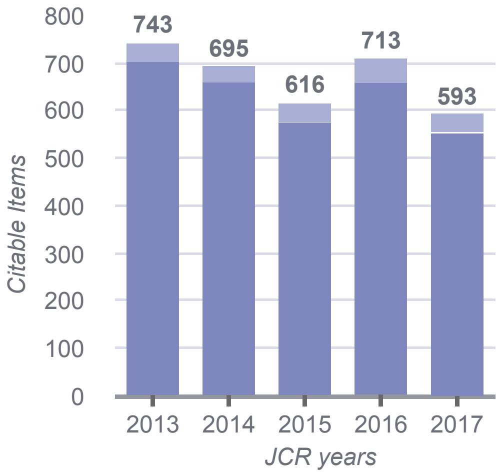

Downloaded on 2019/4/19 上午11:32:06 from Journal Citation Reports (©2019 Clarivate Analytics)

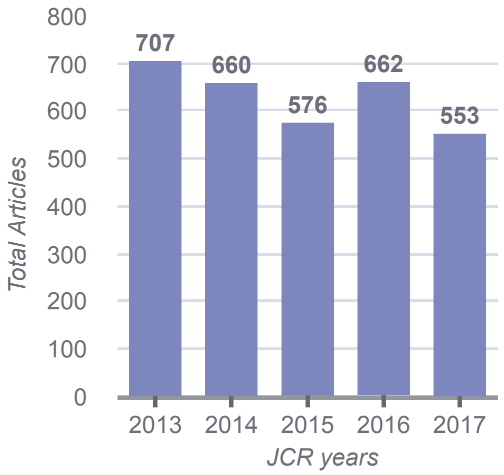

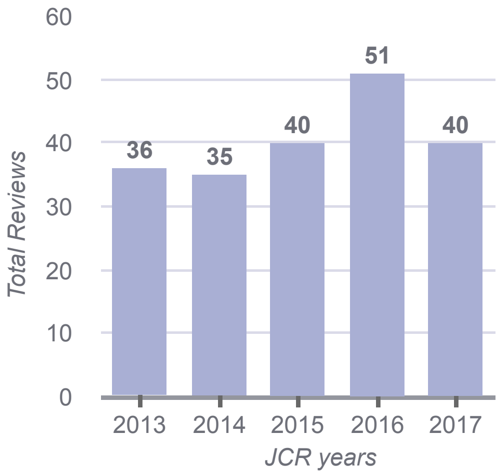

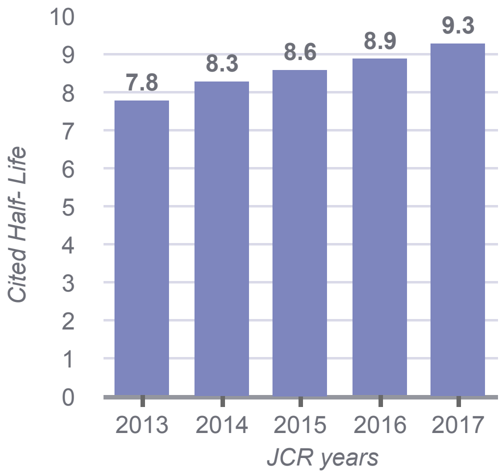

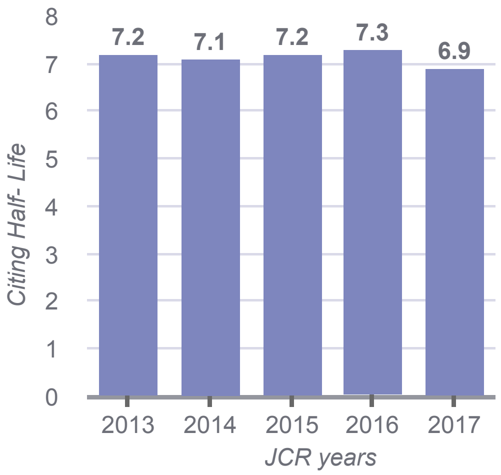

Downloaded on 2019/4/19 上午11:32:06 from Journal Citation Reports (©2019 Clarivate Analytics)

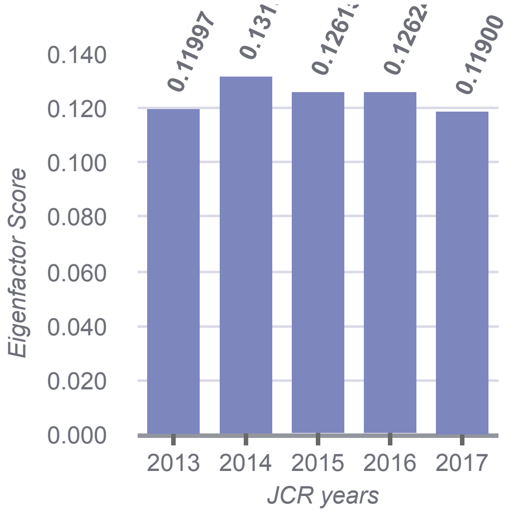

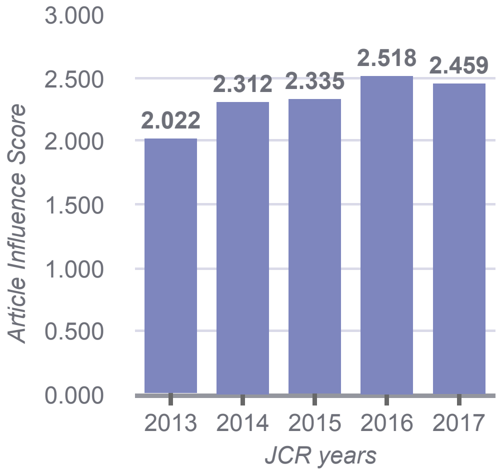

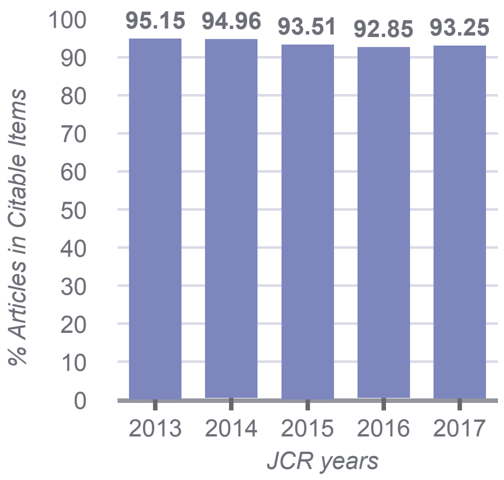

Downloaded on 2019/4/19 上午11:32:06 from Journal Citation Reports (©2019 Clarivate Analytics)

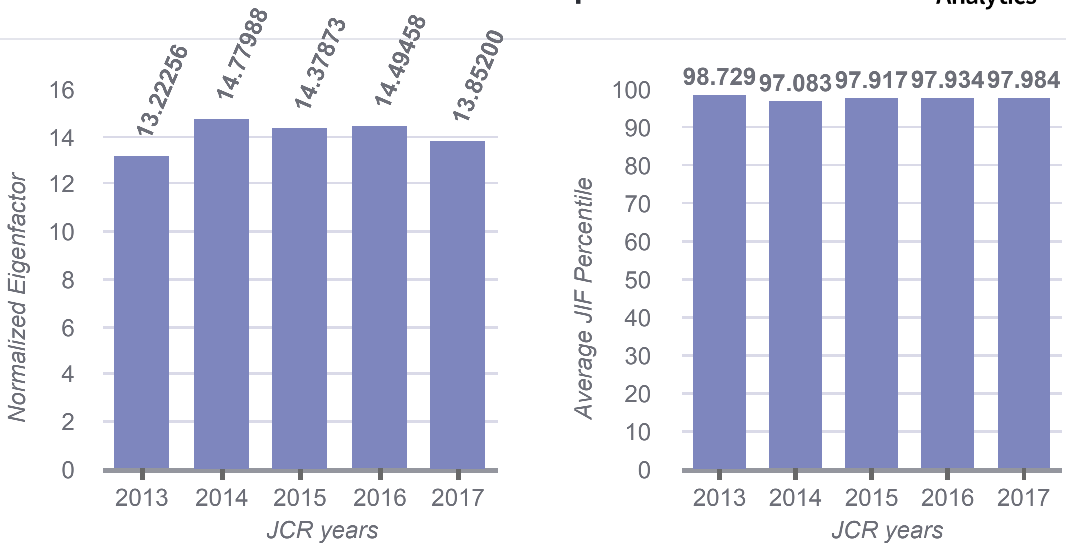

Downloaded on 2019/4/19 上午11:32:06 from Journal Citation Reports (©2019 Clarivate Analytics)

These data summarize the characteristics of the journal's published content for the most recent three years, that is, 2017 and the two prior years, combined. This information is based on all listed authors and addresses. It is meant to be descriptive rather than comparative.

Contributions by country/region

country	count
1. USA	1,684
2. Canada	184
3. England	132
4. Australia	123
5. Netherlands	57
6. France	48
7. Italy	47
8. GERMANY (FED REP GER)	45
9. Sweden	42
10. Norway	33
- Spain	33

Contributions by organizations

organization	count
1. HARVARD UNIVERSITY	287
2. VA BOSTON HEALTHCARE SYSTEM	220
3. UNIVERSITY OF CALIFORNIA SYSTEM	181
4. BOSTON CHILDREN'S HOSPITAL	169
5. UNIVERSITY OF PENNSYLVANIA	164
6. CHILDRENS HOSPITAL OF PHILADELPHIA	139
7. CHILDREN'S MERCY HOSPITAL	104
8. CINCINNATI CHILDREN'S HOSPITAL MEDICAL CENTER	103
9. CENTERS FOR DISEASE CONTROL & PREVENTION - USA	101
10. UNIVERSITY OF WASHINGTON	97

Downloaded on 2019/4/19 上午11:32:06 from Journal Citation Reports (©2019 Clarivate Analytics)
