# Supplementary material for: Emerging trends and knowledge structure of epilepsy during pregnancy research for 2000–2018: a bibliometric analysis
Source: PeerJ. 2019 Jun 7;7:e7115. doi: 10.7717/peerj.7115 (PMC6557303; doi:10.7717/peerj.7115)
Supplement: Supplemental Information 4 [file peerj-07-7115-s004.zip › 7/3. InCites Journal Citation Reports(EPILEPSIA).pdf]

2017 Journal Performance Data for: EPILEPSIA

ISSN: 0013-9580
eISSN: 1528-1167
WILEY
111 RIVER ST, HOBOKEN 07030-5774, NJ
USA

TITLES
ISO: Epilepsia
JCR Abbrev: EPILEPSIA

LANGUAGES
English

CATEGORIES
CLINICAL NEUROLOGY - SCIE

PUBLICATION FREQUENCY
12 issues/year

Downloaded on 2019/4/19 上午11:34:02 from Journal Citation Reports (©2019 Clarivate Analytics)

Current Year

The data in the two graphs below and in the Journal Impact Factor calculation panels represent citation activity in 2017 to items published in the journal in the prior two years. They detail the components of the Journal Impact Factor. Use the "All Years" tab to access key metrics and additional data for the current year and all prior years for this journal.

2017 Journal Impact Factor & percentile rank in category for: EPILEPSIA

5.067

2017 Journal Impact Factor

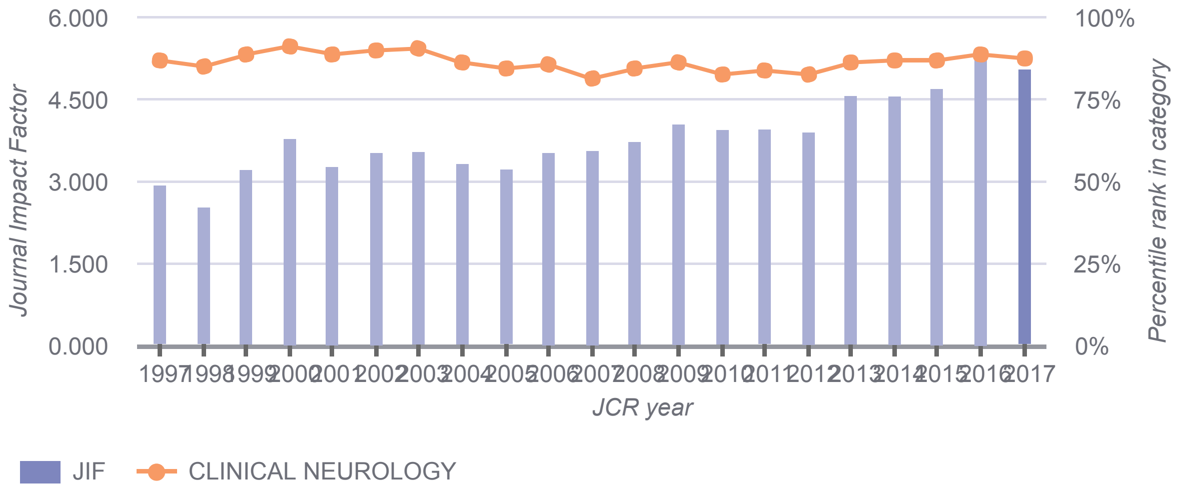

2017 JIF Citation Distribution for: EPILEPSIA

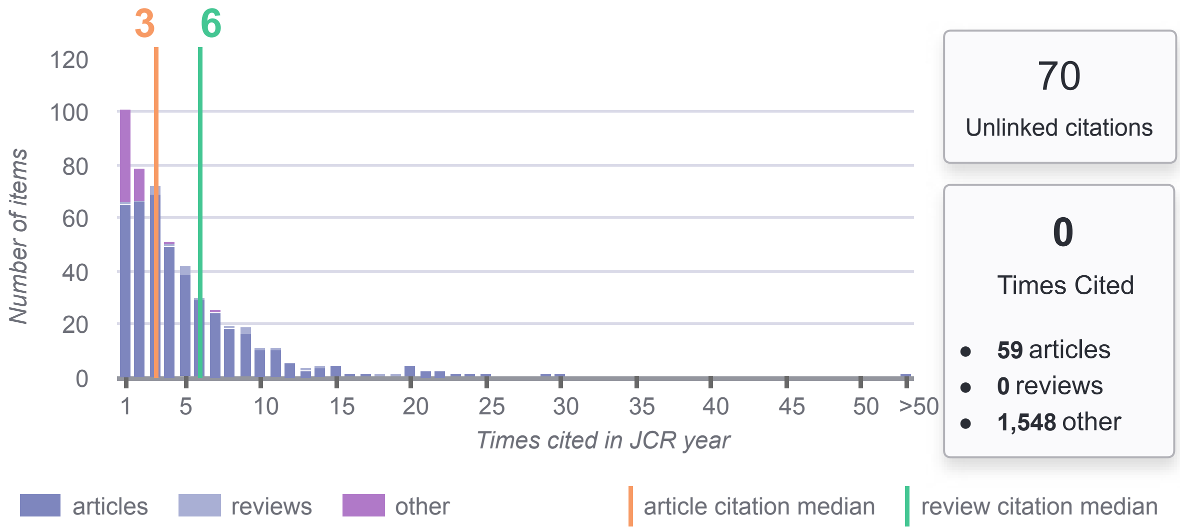

Downloaded on 2019/4/19 上午11:34:02 from Journal Citation Reports (©2019 Clarivate Analytics)

Journal Impact Factor Calculation

$$2017 \text{ Journal Impact Factor} = \frac{2{,}554}{504} = 5.067$$

How is Journal Impact Factor Calculated?

$$\text{JIF} = \frac{\text{Citations in 2017 to items published in } \mathbf{2015\ (1{,}490) + 2016\ (1{,}064)}}{\text{Number of citable items in } \mathbf{2015\ (247) + 2016\ (257)}} = \frac{2{,}554}{504}$$

Downloaded on 2019/4/19 上午11:34:02 from Journal Citation Reports (©2019 Clarivate Analytics)

Journal Impact Factor contributing items

Citable items in 2016 and 2015 (504)

TITLE	CITATIONS COUNTED TOWARDS JIF
A definition and classification of status epilepticus - Report of the ILAE Task Force on Classification of Status Epilepticus<br><br>By: Trinka, Eugen; Cock, Hannah; Hesdorffer, Dale; Rossetti, Andrea O.; Scheffer, Ingrid E.; et al.<br>**Volume: 56 Page: 1515-1523 Accession number: WOS:000362582400012**<br>**Document Type: Article**	104
A randomized, double-blind, placebo-controlled, multicenter, parallel-group study to evaluate the efficacy and safety of adjunctive brivaracetam in adult patients with uncontrolled partial-onset seizures<br><br>By: Klein, Pavel; Schiemann, Jimmy; Sperling, Michael R.; Whitesides, John; Liang, Wei; et al.<br>**Volume: 56 Page: 1890-1898 Accession number: WOS:000366524500007**<br>**Document Type: Article**	30
Laser interstitial thermal therapy for medically intractable mesial temporal lobe epilepsy<br><br>By: Kang, Joon Y.; Sperling, Michael R.; Wu, Chengyuan; Tracy, Joseph; Lorenzo, Matthew; et al.<br>**Volume: 57 Page: 325-334 Accession number: WOS:000370048100019**<br>**Document Type: Article**	29
Diagnostic yield of genetic testing in epileptic encephalopathy in childhood<br><br>By: Mercimek-Mahmutoglu, Saadet; Minassian, Berge A.; Moharir, Mahendranath; Siriwardena, Komudi; Weiss, Shelly K.; et al.<br>**Volume: 56 Page: 707-716 Accession number: WOS:000354641700012**<br>**Document Type: Article**	25
MR-guided laser interstitial thermal therapy for pediatric drug-resistant lesional epilepsy<br><br>By: Lewis, Evan Cole; Weil, Alexander G.; Duchowny, Michael; Bhatia, Sanjiv; Ragheb, John; et al.<br>**Volume: 56 Page: 1590-1598 Accession number: WOS:000362582400021**<br>**Document Type: Article**	24
Summary of recommendations for the management of infantile seizures: Task Force Report for the ILAE Commission of Pediatrics<br><br>By: Wilmshurst, Jo M.; Jovic, Nebojsa J.; Nordli, Doug; Hirtz, Deborah; Wong, Virginia; et al.<br>**Volume: 56 Page: 1185-1197 Accession number: WOS:000358958700004**<br>**Document Type: Article**	23
Valproate in the treatment of epilepsy in girls and women of childbearing potential<br><br>By: Tomson, Torbjorn; Marson, Anthony; Boon, Paul; Canevini, Maria Paola; Covanis, Athanasios; et al.<br>**Volume: 56 Page: 1006-1019 Accession number: WOS:000357728400007**<br>**Document Type: Article**	22

Downloaded on 2019/4/19 上午11:34:02 from Journal Citation Reports (©2019 Clarivate Analytics)

Citations in 2017 (2,554)

TITLE	CITATIONS COUNTED TOWARDS JIF
EPILEPSIA	251
EPILEPSY & BEHAVIOR	204
SEIZURE-EUROPEAN JOURNAL OF EPILEPSY	152
EPILEPSY RESEARCH	88
CURRENT PHARMACEUTICAL DESIGN	68
FRONTIERS IN NEUROLOGY	41
SEMINARS IN PEDIATRIC NEUROLOGY	41
CURRENT OPINION IN NEUROLOGY	36
NEUROLOGY	31
SCIENTIFIC REPORTS	30

Downloaded on 2019/4/19 上午11:34:02 from Journal Citation Reports (©2019 Clarivate Analytics)

Key Indicators 2017

IMPACT METRICS		INFLUENCE METRICS		SOURCE METRICS	
Total Cites	26,301	Eigenfactor Score	0.03200	Citable Items	282
Journal Impact Factor	5.067	Article Influence Score	1.533	% Articles in Citable Items	89.01
5 Year Impact Factor	5.176	Normalized Eigenfactor	3.79200	Average JIF Percentile	88.071
Immediacy Index	1.401			Cited Half-Life	8.7
Impact Factor Without Journal Self Cites	4.569			Citing Half-Life	7.8

Source data

Journal source data 2017

	Articles	Reviews	Combined(C)	Other(O)	Percentage(C/(C+O))
Number in JCR Year 2017 (A)	251	31	282	633	30%
Number of References (B)	8,631	2,589	11,220	152	98%
Ratio (B/A)	34.4	83.5	39.8	0.2	

Downloaded on 2019/4/19 上午11:34:02 from Journal Citation Reports (©2019 Clarivate Analytics)

Box plot

Category Box Plot 2017

Category Box Plot

The category box plot depicts the distribution of Impact Factors for all journals in the category. The horizontal line that forms the top of the box is the 75th percentile (Q1). The horizontal line that forms the bottom is the 25th percentile (Q3). The horizontal line that intersects the box is the median Impact Factor for the category. Horizontal lines above and below the box, called whiskers, represent maximum and minimum values.
The top whisker is the smaller of the following two values:
the maximum Impact Factor (IF)
Q1 IF + 3.5(Q1 IF - Q3 IF)
The bottom whisker is the larger of the following two values:
the minimum Impact Factor (IF)
Q1 IF - 3.5(Q1 IF - Q3 IF)
Box Plots are provided for the current JCR year for each of the categories in which the journal is indexed.

EPILEPSIA, IF: 5.067

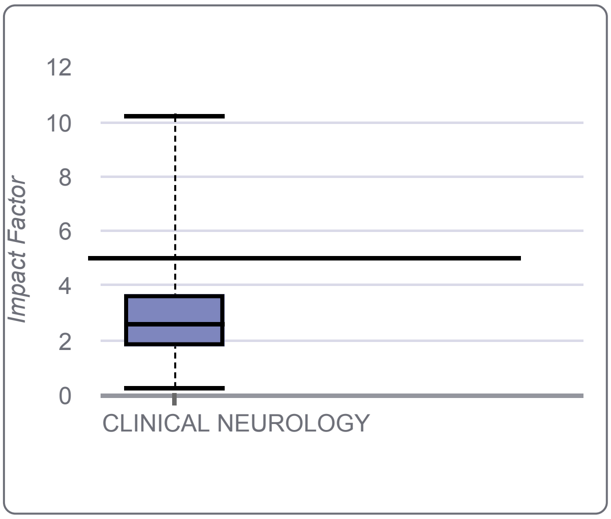

Downloaded on 2019/4/19 上午11:34:02 from Journal Citation Reports (©2019 Clarivate Analytics)

Rank

Rank 2017

JCR Impact Factor

JCR Year	CLINICAL NEUROLOGY		
	Rank	Quartile	JIF Percentile
2017	24/197	Q1	88.071
2016	22/194	Q1	88.918
2015	25/193	Q1	87.306
2014	25/192	Q1	87.240
2013	26/194	Q1	86.856
2012	33/193	Q1	83.161
2011	31/192	Q1	84.115
2010	32/185	Q1	82.973
2009	23/167	Q1	86.527
2008	24/156	Q1	84.936
2007	27/146	Q1	81.849
2006	21/147	Q1	86.054
2005	23/148	Q1	84.797
2004	19/140	Q1	86.786
2003	13/135	Q1	90.741
2002	14/138	Q1	90.217
2001	15/136	Q1	89.338
2000	12/137	Q1	91.606
1999	15/132	Q1	89.015
1998	19/125	Q1	85.200

Downloaded on 2019/4/19 上午11:34:02 from Journal Citation Reports (©2019 Clarivate Analytics)

Downloaded on 2019/4/19 上午11:34:02 from Journal Citation Reports (©2019 Clarivate Analytics)

ESI Total Citations 2017

Rank

JCR Year	NEUROSCIENCE & BEHAVIOR
2017	22/346-Q1
2016	21/345-Q1
2015	22/344-Q1
2014	22/337-Q1
2013	24/339-Q1

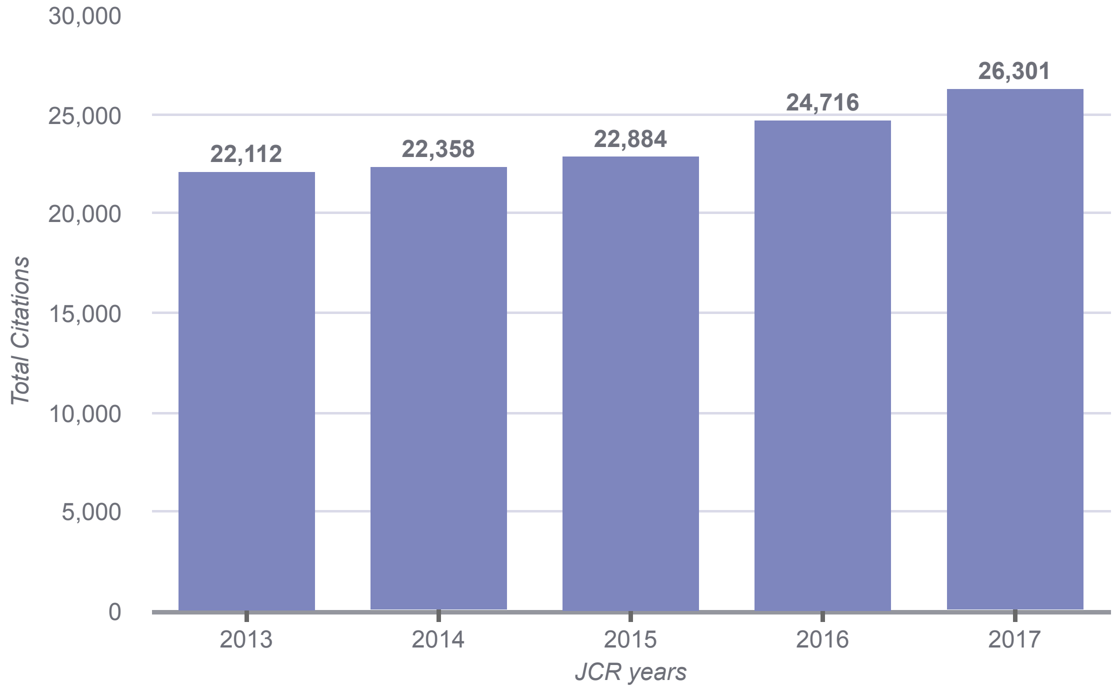

Downloaded on 2019/4/19 上午11:34:02 from Journal Citation Reports (©2019 Clarivate Analytics)

Cited Journal Data

Cited Half-Life Data

Customize columns

Cited Year	2017	2016	2015	2014	2013	2012	2011	2010	2009	2008	20
#Cites from 2017	395	1,064	1,490	1,629	2,045	1,733	1,865	2,036	1,366	1,199	
Cumulative %	1.50%	5.55%	11.21%	17.41%	25.18%	31.77%	38.86%	46.60%	51.80%	56.36%	10

Cited Journal Graph 2017

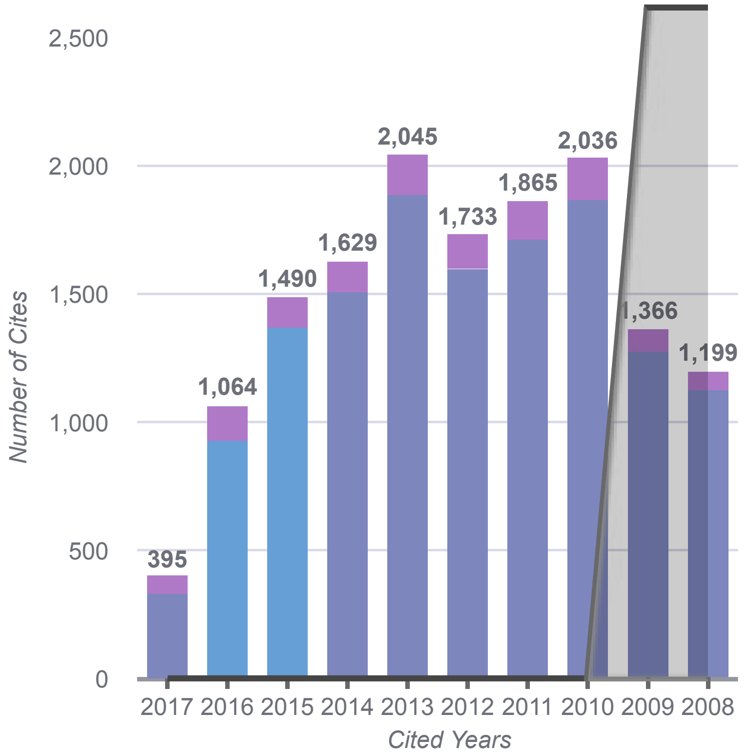

CITED JOURNAL GRAPH

The Cited Journal Graph shows the distribution (by cited year) of citations published in journals during the JCR year to items published in the Journal during the last 10 years.

The white/grey division indicates the cited half-life (if < 10.0). Half of the citations are to items that were published more recently than the cited half-life.

The two light-blue columns indicate citations used to calculate the Impact Factor (always the 2nd and 3rd columns).

Downloaded on 2019/4/19 上午11:34:02 from Journal Citation Reports (©2019 Clarivate Analytics)

Cited Journal Data

Customize columns

	Impact	Citing Journal	All Yrs	2017	2016	2015	2014	2013	2012	2011	2010	2009	20
		ALL Journals	26,301	395	1,064	1,490	1,629	2,045	1,733	1,865	2,036	1,366	1,
		ALL OTHERS (701)	701	6	19	32	39	41	66	40	46	32	
1	2.600	EPILEPSY BEHAV	2,093	24	80	124	118	154	115	148	162	103	
2	5.067	EPILEPSIA	1,899	69	136	115	121	156	135	146	161	84	
3	2.839	SEIZURE-EUR J EPILEP	1,508	25	60	92	94	132	90	100	107	82	
4	2.491	EPILEPSY RES	984	13	44	44	63	77	74	69	75	52	
5	2.757	CURR PHARM DESIGN	611	13	34	34	30	56	40	55	34	34	
6	3.508	FRONT NEUROL	402	18	23	18	22	37	28	33	41	16	
7	4.122	SCI REP-UK	351	2	13	17	26	31	31	27	32	22	
8	3.614	CLIN NEUROPHYSIOL	342	8	7	16	31	23	18	20	17	20	
9	8.055	NEUROLOGY	315	7	12	19	24	31	24	27	33	10	
10	6.754	COCHRANE DB SYST REV	290	0	3	9	12	13	12	6	17	11	
11	1.500	EPILEPTIC DISORD	271	3	14	13	12	19	15	17	17	13	
12	1.878	SEMIN PEDIATR NEUROL	260	10	18	23	10	15	8	32	12	15	
13	2.766	PLOS ONE	252	1	7	6	10	22	17	12	18	19	

Rows 1 - 15 of 1,138 *(use csv export to download the full table)*

Downloaded on 2019/4/19 上午11:34:02 from Journal Citation Reports (©2019 Clarivate Analytics)

Citing Journal Data

Citing Half-Life Data

Customize columns

Citing Year	2017	2016	2015	2014	2013	2012	2011	2010	2009	2008	20
#Cites from 2017	217	808	993	823	797	797	719	631	510	481	
Cumulative %	1.91%	9.01%	17.75%	24.98%	31.99%	39.00%	45.32%	50.87%	55.36%	59.58%	10

Citing Journal Graph 2017

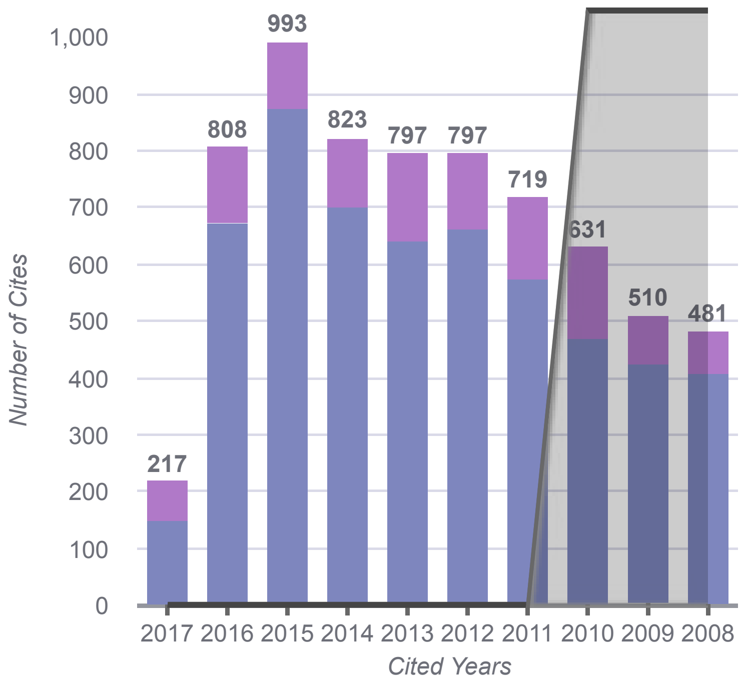

CITING JOURNAL GRAPH

The Citing Journal Graph shows the distribution (by cited year) of citations published in the Journal during the JCR year to items published in journals during the last 10 years.

The white/grey division indicates the citing half-life (if < 10.0). Half of the citations are to items that were published more recently than the citing half-life.

Downloaded on 2019/4/19 上午11:34:02 from Journal Citation Reports (©2019 Clarivate Analytics)

Clarivate Analytics

Citing Journal Data

Customize columns

	Impact	Cited Journal	All Yrs	2017	2016	2015	2014	2013	2012	2011	2010	2009	2008	R
		ALL Journals	11,372	217	808	993	823	797	797	719	631	510	481	4,
		ALL OTHERS (1018)	1,018	22	80	100	70	70	62	42	46	36	45	
1	5.067	EPILEPSIA	1,899	69	136	115	121	156	135	146	161	84	74	
2	8.055	NEUROLOGY	579	5	30	46	33	31	43	37	21	25	36	
3	2.600	EPILEPSY BEHAV	482	11	36	82	43	38	53	32	27	30	25	
4	2.491	EPILEPSY RES	331	2	24	31	22	29	25	23	17	26	10	
5	10.848	BRAIN	326	7	15	19	20	11	21	32	15	20	28	
6	10.250	ANN NEUROL	252	2	17	26	20	12	24	8	10	5	6	
7	2.839	SEIZURE-EUR J EPILEP	243	4	32	35	20	18	21	27	8	14	5	
8	5.971	J NEUROSCI	229	0	6	15	23	12	13	10	14	18	20	
9	27.144	LANCET NEUROL	132	3	20	4	14	19	5	11	6	4	19	
10	5.426	NEUROIMAGE	125	3	1	3	15	8	15	8	15	7	7	
11	3.614	CLIN NEUROPHYSIOL	123	2	29	8	6	8	11	7	6	1	2	
12	4.319	J NEUROSURG	121	2	11	9	6	7	10	3	9	11	2	
13	2.766	PLOS ONE	108	2	12	17	17	19	18	11	6	2	1	
14	7.144	J NEUROL NEUROSUR PS	107	3	8	4	12	3	7	1	3	1	0	

Rows 1 - 16 of 667 *(use csv export to download the full table)*

Downloaded on 2019/4/19 上午11:34:02 from Journal Citation Reports (©2019 Clarivate Analytics)

Clarivate Analytics

Metric trend

Metric Trend

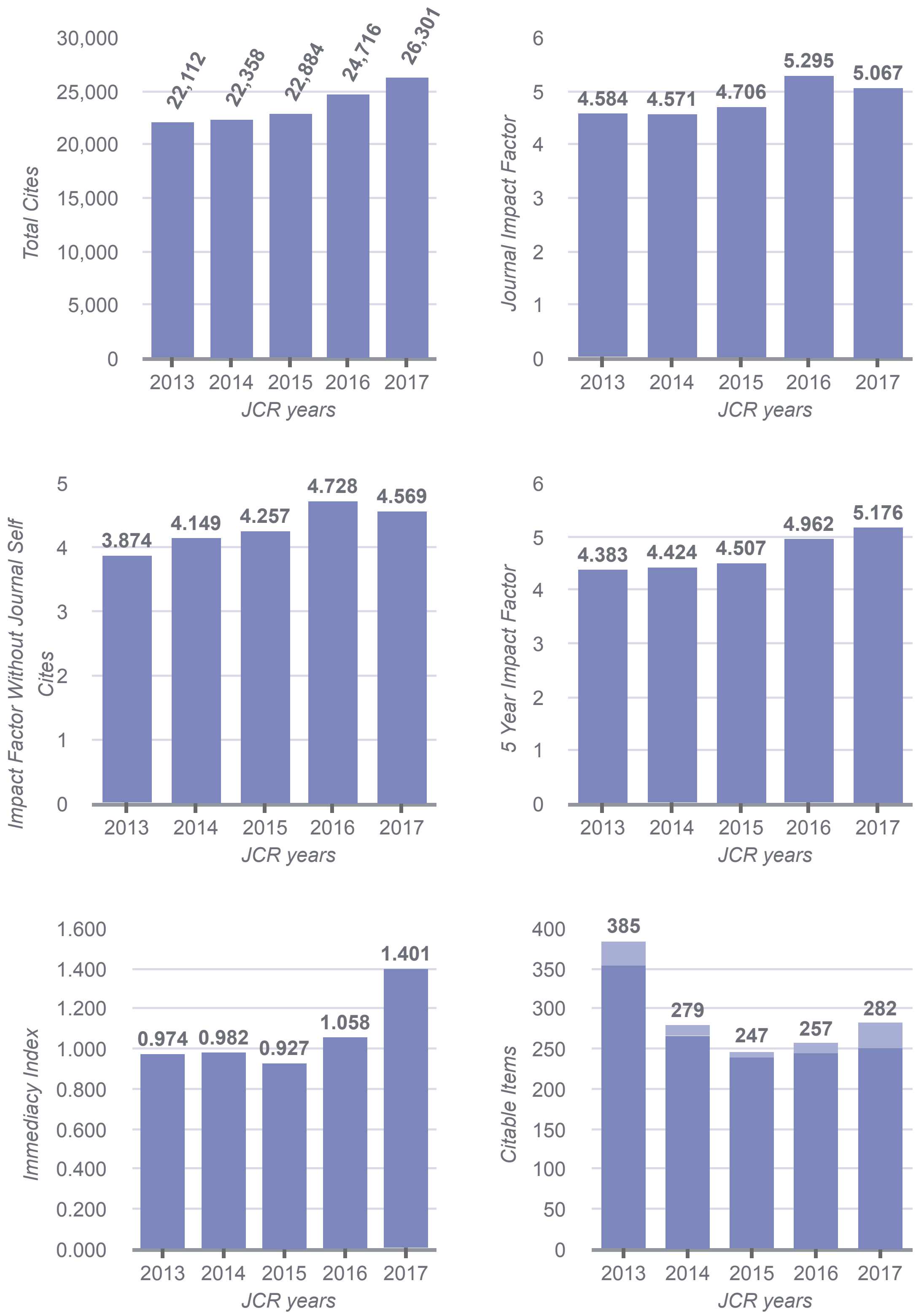

Downloaded on 2019/4/19 上午11:34:02 from Journal Citation Reports (©2019 Clarivate Analytics)

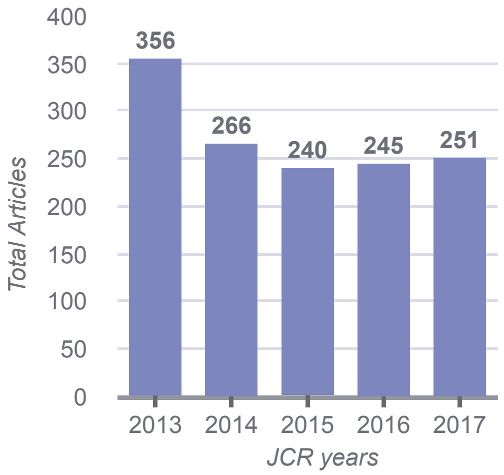

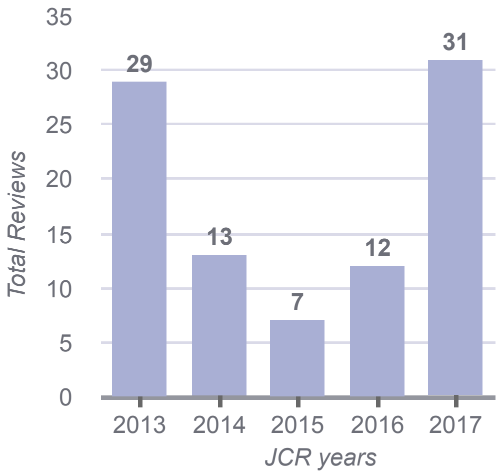

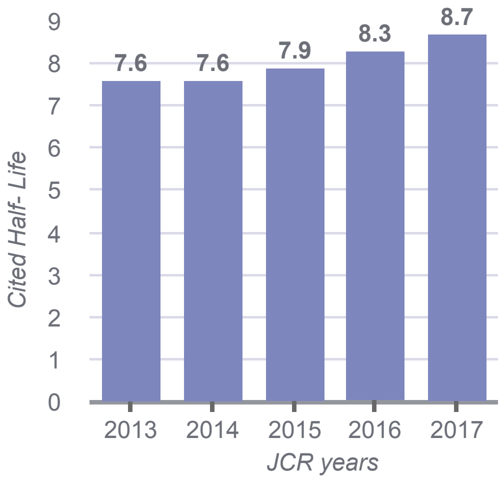

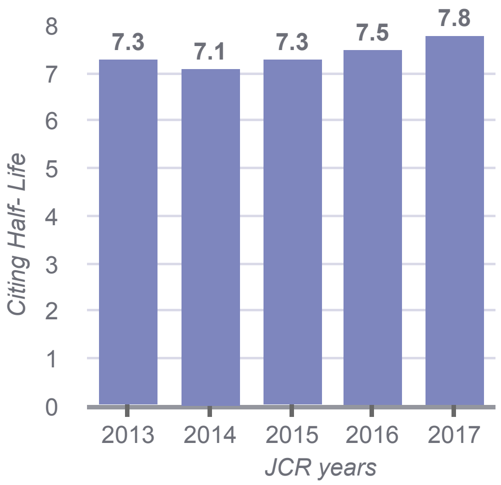

Downloaded on 2019/4/19 上午11:34:02 from Journal Citation Reports (©2019 Clarivate Analytics)

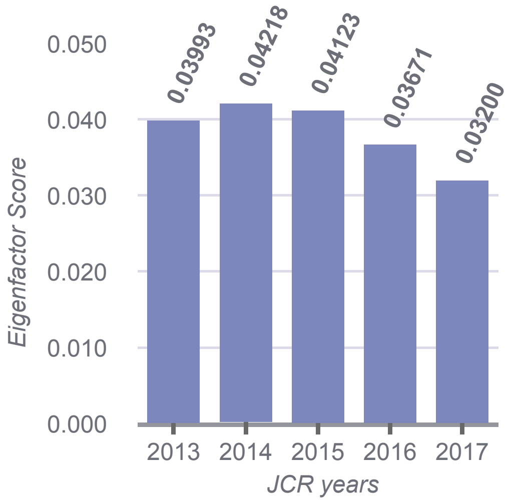

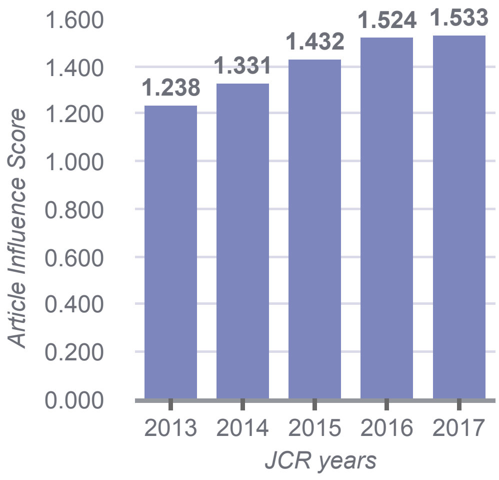

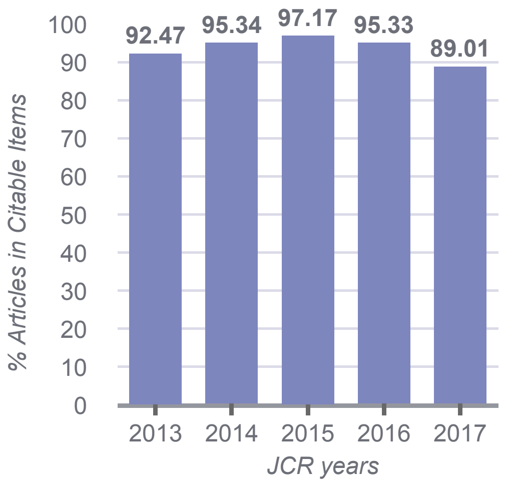

Downloaded on 2019/4/19 上午11:34:02 from Journal Citation Reports (©2019 Clarivate Analytics)

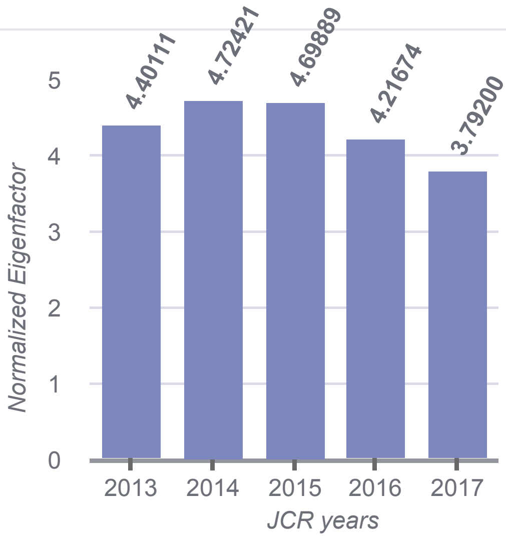

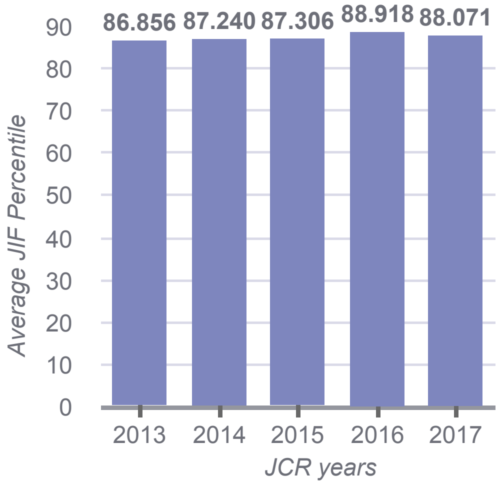

Downloaded on 2019/4/19 上午11:34:02 from Journal Citation Reports (©2019 Clarivate Analytics)

These data summarize the characteristics of the journal's published content for the most recent three years, that is, 2017 and the two prior years, combined. This information is based on all listed authors and addresses. It is meant to be descriptive rather than comparative.

Contributions by country/region

country	count
1. USA	695
2. England	334
3. Italy	329
4. GERMANY (FED REP GER)	287
5. Turkey	213
6. France	182
7. Netherlands	173
8. Canada	165
9. Australia	134
10. Belgium	117

Contributions by organizations

organization	count
1. UNIVERSITY OF LONDON	173
2. UNIVERSITY OF MELBOURNE	94
3. UNIVERSITY OF CALIFORNIA SYSTEM	86
4. INSTITUT NATIONAL DE LA SANTE ET DE LA RECHERCHE MEDICALE (INSERM)	81
5. UTRECHT UNIVERSITY	79
6. HARVARD UNIVERSITY	66
7. ASSISTANCE PUBLIQUE HOPITAUX PARIS (APHP)	59
8. FLOREY INSTITUTE OF NEUROSCIENCE & MENTAL HEALTH	58
9. ISTANBUL UNIVERSITY	55
10. DANISH EPILEPSY CTR	54

Downloaded on 2019/4/19 上午11:34:02 from Journal Citation Reports (©2019 Clarivate Analytics)
