# Supplementary material for: Emerging trends and knowledge structure of epilepsy during pregnancy research for 2000–2018: a bibliometric analysis
Source: PeerJ. 2019 Jun 7;7:e7115. doi: 10.7717/peerj.7115 (PMC6557303; doi:10.7717/peerj.7115)
Supplement: Supplemental Information 4 [file peerj-07-7115-s004.zip › 7/4. InCites Journal Citation Reports(ACTA NEUROLOGICA SCANDINAVICA).pdf]

2017 Journal Performance Data for: ACTA NEUROLOGICA SCANDINAVICA

ISSN: 0001-6314
eISSN: 1600-0404
WILEY
111 RIVER ST, HOBOKEN 07030-5774, NJ
DENMARK

TITLES
ISO: Acta Neurol. Scand.
JCR Abbrev: ACTA NEUROL SCAND

LANGUAGES
English

CATEGORIES
CLINICAL NEUROLOGY - SCIE

PUBLICATION FREQUENCY
12 issues/year

Downloaded on 2019/4/19 上午11:35:07 from Journal Citation Reports (©2019 Clarivate Analytics)

Current Year

The data in the two graphs below and in the Journal Impact Factor calculation panels represent citation activity in 2017 to items published in the journal in the prior two years. They detail the components of the Journal Impact Factor. Use the "All Years" tab to access key metrics and additional data for the current year and all prior years for this journal.

2017 Journal Impact Factor & percentile rank in category for: ACTA NEUROLOGICA SCANDINAVICA

3.126
2017 Journal Impact Factor

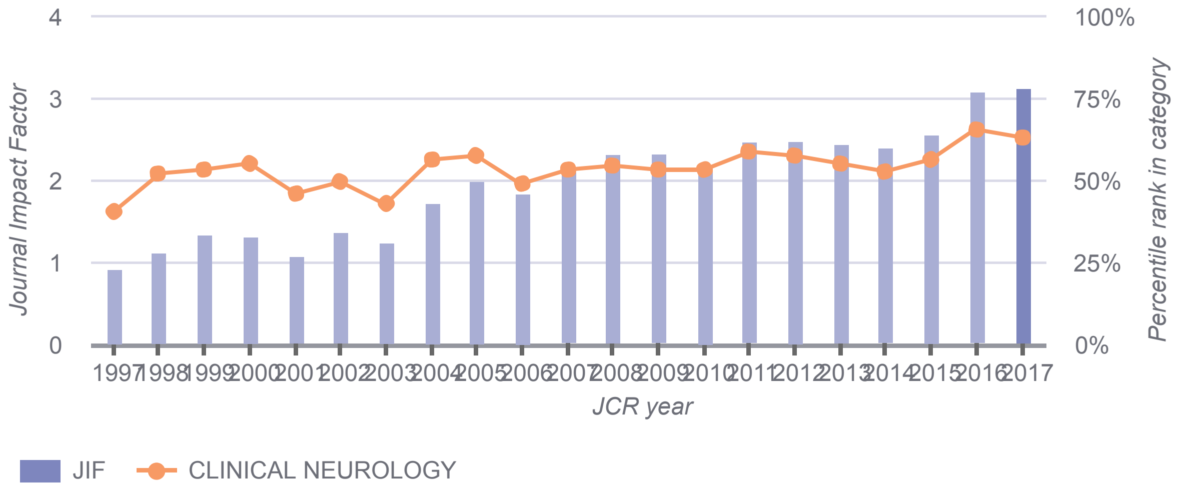

2017 JIF Citation Distribution for: ACTA NEUROLOGICA SCANDINAVICA

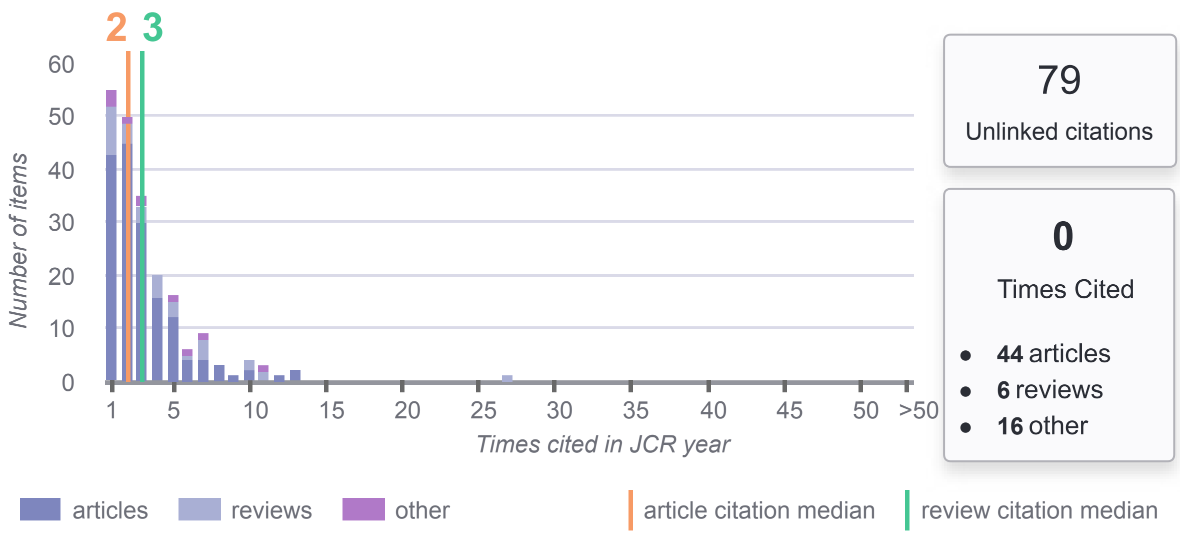

79
Unlinked citations

0
Times Cited

- **44** articles
- **6** reviews
- **16** other

Downloaded on 2019/4/19 上午11:35:07 from Journal Citation Reports (©2019 Clarivate Analytics)

Journal Impact Factor Calculation

$$\text{2017 Journal Impact Factor} = \frac{769}{246} = 3.126$$

How is Journal Impact Factor Calculated?

$$\text{JIF} = \frac{\text{Citations in 2017 to items published in } \mathbf{2015\ (383) + 2016\ (386)}}{\text{Number of citable items in } \mathbf{2015\ (122) + 2016\ (124)}} = \frac{769}{246}$$

Downloaded on 2019/4/19 上午11:35:07 from Journal Citation Reports (©2019 Clarivate Analytics)

Journal Impact Factor contributing items

Citable items in 2016 and 2015 (246)

TITLE	CITATIONS COUNTED TOWARDS JIF
The genetic background of Parkinson's disease: current progress and future prospects<br>By: Kalinderi, K.; Bostantjopoulou, S.; Fidani, L.<br>**Volume: 134 Page: 314-326 Accession number: WOS:000386752700001**<br>**Document Type: Review**	**27**
Greater occipital nerve blockade for the treatment of chronic migraine: a randomized, multicenter, double-blind, and placebo-controlled study<br>By: Inan, L. E.; Inan, N.; Karadas, O.; Gul, H. L.; Erdemoglu, A. K.; et al.<br>**Volume: 132 Page: 270-277 Accession number: WOS:000360801700007**<br>**Document Type: Article**	**13**
An open-label evaluator-blinded clinical study of minocycline neuroprotection in ischemic stroke: gender-dependent effect<br>By: Amiri-Nikpour, M. R.; Nazarbaghi, S.; Hamdi-Holasou, M.; Rezaei, Y.<br>**Volume: 131 Page: 45-50 Accession number: WOS:000347004800006**<br>**Document Type: Article**	**13**
Burden and cost of neurological diseases: a European North-South comparison<br>By: Raggi, A.; Leonardi, M.<br>**Volume: 132 Page: 16-22 Accession number: WOS:000357734600003**<br>**Document Type: Article**	**12**
Registers of multiple sclerosis in Denmark<br>By: Koch-Henriksen, N.; Magyari, M.; Laursen, B.<br>**Volume: 132 Page: 4-10 Accession number: WOS:000355852600002**<br>**Document Type: Review**	**11**
Multiple sclerosis and environmental factors: the role of vitamin D, parasites, and Epstein-Barr virus infection<br>By: Correale, J.; Gaitan, M. I.<br>**Volume: 132 Page: 46-55 Accession number: WOS:000355852600009**<br>**Document Type: Review**	**11**
Non-motor symptoms and quality of life in tremor dominant vs postural instability gait disorder Parkinson's disease patients<br>By: Wu, Y.; Guo, X. -Y.; Wei, Q. -Q.; Ou, R. -W.; Song, W.; et al.<br>**Volume: 133 Page: 330-337 Accession number: WOS:000373774300002**<br>**Document Type: Article**	**10**

Downloaded on 2019/4/19 上午11:35:07 from Journal Citation Reports (©2019 Clarivate Analytics)

Citations in 2017 (769)

TITLE	CITATIONS COUNTED TOWARDS JIF
ACTA NEUROLOGICA SCANDINAVICA	91
JOURNAL OF THE NEUROLOGICAL SCIENCES	15
SEIZURE-EUROPEAN JOURNAL OF EPILEPSY	14
PLOS ONE	13
BRAIN AND BEHAVIOR	12
FRONTIERS IN NEUROLOGY	12
EPILEPSY & BEHAVIOR	11
JOURNAL OF NEUROLOGY	10
MULTIPLE SCLEROSIS JOURNAL	9
NATURE REVIEWS NEUROLOGY	9

Downloaded on 2019/4/19 上午11:35:07 from Journal Citation Reports (©2019 Clarivate Analytics)

Key Indicators 2017

IMPACT METRICS		INFLUENCE METRICS		SOURCE METRICS	
Total Cites	6,943	Eigenfactor Score	0.00800	Citable Items	186
Journal Impact Factor	3.126	Article Influence Score	0.858	% Articles in Citable Items	81.72
5 Year Impact Factor	2.877	Normalized Eigenfactor	0.90700	Average JIF Percentile	63.198
Immediacy Index	1.016			Cited Half-Life	10.8
Impact Factor Without Journal Self Cites	2.756			Citing Half-Life	7.7

Source data

Journal source data 2017

	Articles	Reviews	Combined(C)	Other(O)	Percentage(C/(C+O))
Number in JCR Year 2017 (A)	152	34	186	24	88%
Number of References (B)	4,572	2,101	6,673	190	97%
Ratio (B/A)	30.1	61.8	35.9	7.9	

Downloaded on 2019/4/19 上午11:35:07 from Journal Citation Reports (©2019 Clarivate Analytics)

Box plot

Category Box Plot 2017

Category Box Plot

The category box plot depicts the distribution of Impact Factors for all journals in the category. The horizontal line that forms the top of the box is the 75th percentile (Q1). The horizontal line that forms the bottom is the 25th percentile (Q3). The horizontal line that intersects the box is the median Impact Factor for the category. Horizontal lines above and below the box, called whiskers, represent maximum and minimum values.
The top whisker is the smaller of the following two values:
the maximum Impact Factor (IF)
Q1 IF + 3.5(Q1 IF - Q3 IF)
The bottom whisker is the larger of the following two values:
the minimum Impact Factor (IF)
Q1 IF - 3.5(Q1 IF - Q3 IF)
Box Plots are provided for the current JCR year for each of the categories in which the journal is indexed.

ACTA NEUROL SCAND, IF: 3.126

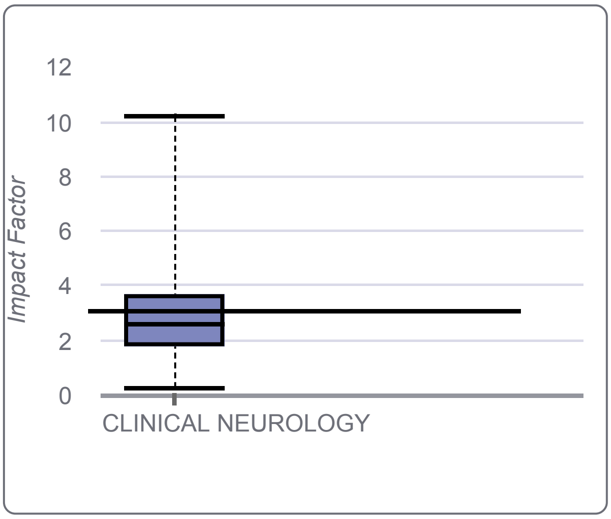

Downloaded on 2019/4/19 上午11:35:07 from Journal Citation Reports (©2019 Clarivate Analytics)

Rank

Rank 2017

JCR Impact Factor

JCR Year	CLINICAL NEUROLOGY		
	Rank	Quartile	JIF Percentile
2017	73/197	Q2	63.198
2016	67/194	Q2	65.722
2015	84/193	Q2	56.736
2014	91/192	Q2	52.865
2013	87/194	Q2	55.412
2012	82/193	Q2	57.772
2011	79/192	Q2	59.115
2010	86/185	Q2	53.784
2009	78/167	Q2	53.593
2008	71/156	Q2	54.808
2007	68/146	Q2	53.767
2006	75/147	Q3	49.320
2005	63/148	Q2	57.770
2004	61/140	Q2	56.786
2003	77/135	Q3	43.333
2002	70/138	Q3	49.638
2001	74/136	Q3	45.956
2000	62/137	Q2	55.109
1999	62/132	Q2	53.409
1998	60/125	Q2	52.400

Downloaded on 2019/4/19 上午11:35:07 from Journal Citation Reports (©2019 Clarivate Analytics)

Downloaded on 2019/4/19 上午11:35:07 from Journal Citation Reports (©2019 Clarivate Analytics)

ESI Total Citations 2017

Rank

JCR Year	NEUROSCIENCE & BEHAVIOR
2017	99/346-Q2
2016	109/345-Q2
2015	101/344-Q2
2014	99/337-Q2
2013	89/339-Q2

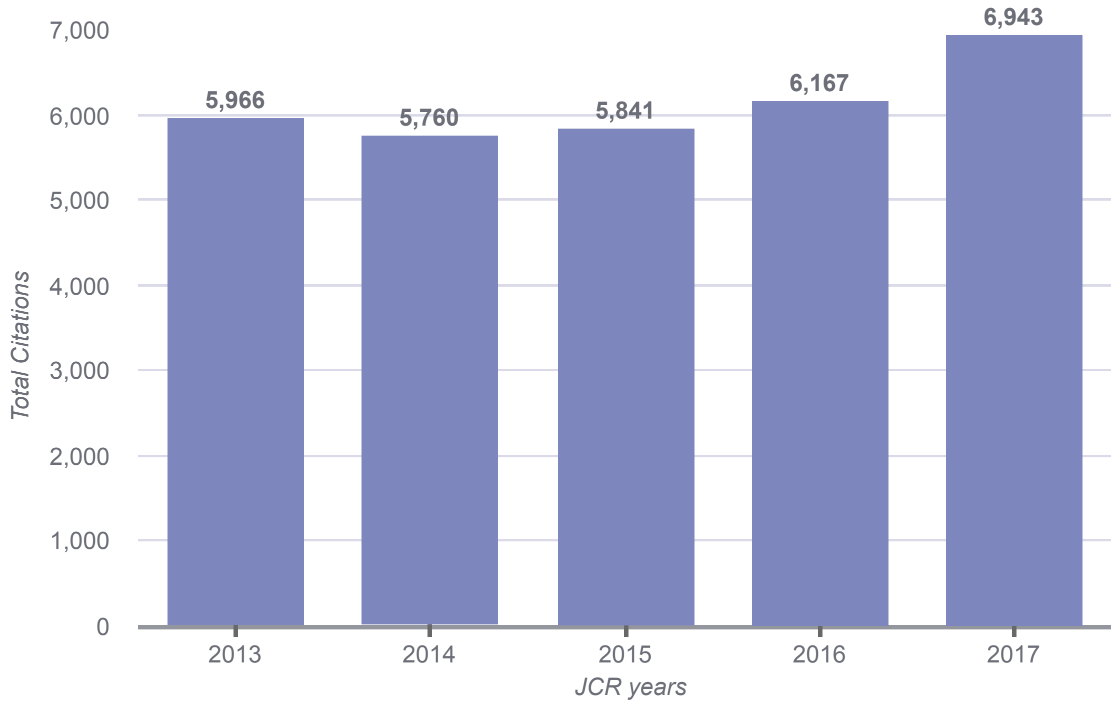

Downloaded on 2019/4/19 上午11:35:07 from Journal Citation Reports (©2019 Clarivate Analytics)

Cited Journal Data

Cited Half-Life Data

Customize columns

Cited Year	2017	2016	2015	2014	2013	2012	2011	2010	2009	2008	20
#Cites from 2017	189	386	383	380	346	395	280	317	299	256	
Cumulative %	2.72%	8.28%	13.80%	19.27%	24.25%	29.94%	33.98%	38.54%	42.85%	46.54%	10

Cited Journal Graph 2017

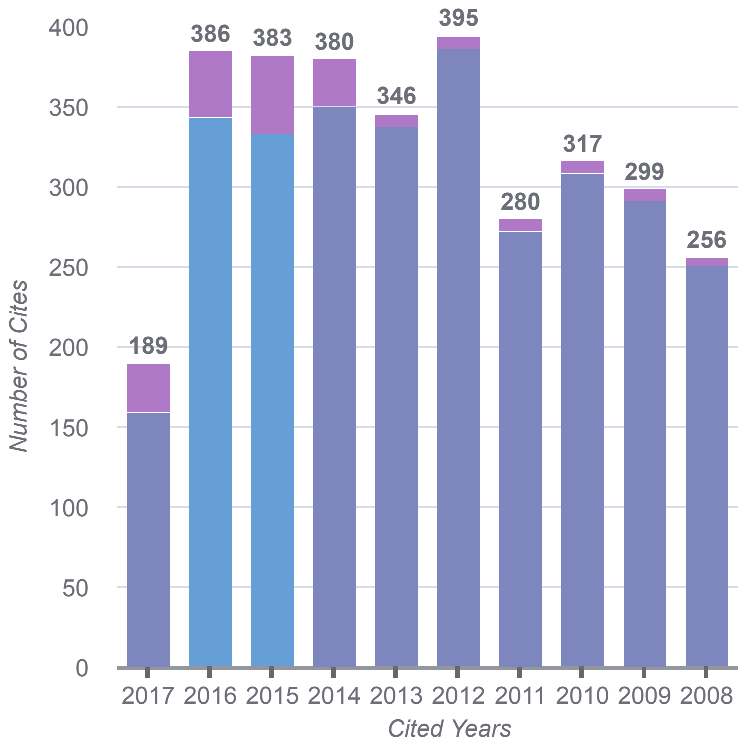

CITED JOURNAL GRAPH

The Cited Journal Graph shows the distribution (by cited year) of citations published in journals during the JCR year to items published in the Journal during the last 10 years.

The white/grey division indicates the cited half-life (if < 10.0). Half of the citations are to items that were published more recently than the cited half-life.

The two light-blue columns indicate citations used to calculate the Impact Factor (always the 2nd and 3rd columns).

Downloaded on 2019/4/19 上午11:35:07 from Journal Citation Reports (©2019 Clarivate Analytics)

Cited Journal Data

Customize columns

	Impact	Citing Journal	All Yrs	2017	2016	2015	2014	2013	2012	2011	2010	2009	2008	R
		ALL Journals	6,943	189	386	383	380	346	395	280	317	299	256	3,
		ALL OTHERS (853)	853	18	32	34	38	38	39	34	33	37	35	
1	3.126	ACTA NEUROL SCAND	241	30	42	49	29	8	8	8	8	7	5	
2	2.766	PLOS ONE	131	0	5	8	14	10	10	5	7	3	5	
3	2.448	J NEUROL SCI	104	0	9	6	9	7	3	4	9	2	4	
4	2.600	EPILEPSY BEHAV	91	3	10	1	6	3	3	6	2	12	1	
5	2.839	SEIZURE-EUR J EPILEP	88	1	11	3	7	7	7	4	3	3	2	
6	4.122	SCI REP-UK	85	3	2	1	5	1	6	6	2	2	4	
7	3.508	FRONT NEUROL	82	1	4	8	8	3	10	0	5	5	2	
8	3.783	J NEUROL	81	4	7	3	4	6	5	2	5	6	2	
9	2.371	INT REV NEUROBIOL	78	5	8	4	2	6	2	4	2	0	2	
10	5.280	MULT SCLER J	64	0	0	9	7	8	11	3	3	3	3	
11	2.219	BRAIN BEHAV	60	1	7	5	6	4	6	3	2	4	2	
12	5.067	EPILEPSIA	58	4	2	2	6	8	1	7	2	3	1	
13	8.055	NEUROLOGY	54	3	3	3	4	1	3	1	1	1	4	
14	3.582	FRONT AGING NEUROSCI	49	3	1	1	2	2	3	3	0	3	1	

Rows 1 - 16 of 844 *(use csv export to download the full table)*

Downloaded on 2019/4/19 上午11:35:07 from Journal Citation Reports (©2019 Clarivate Analytics)

Clarivate Analytics

Citing Journal Data

Citing Half-Life Data

Customize columns

Citing Year	2017	2016	2015	2014	2013	2012	2011	2010	2009	2008	20
#Cites from 2017	93	391	579	623	534	480	435	410	349	299	
Cumulative %	1.36%	7.05%	15.49%	24.57%	32.35%	39.34%	45.68%	51.65%	56.74%	61.10%	10

Citing Journal Graph 2017

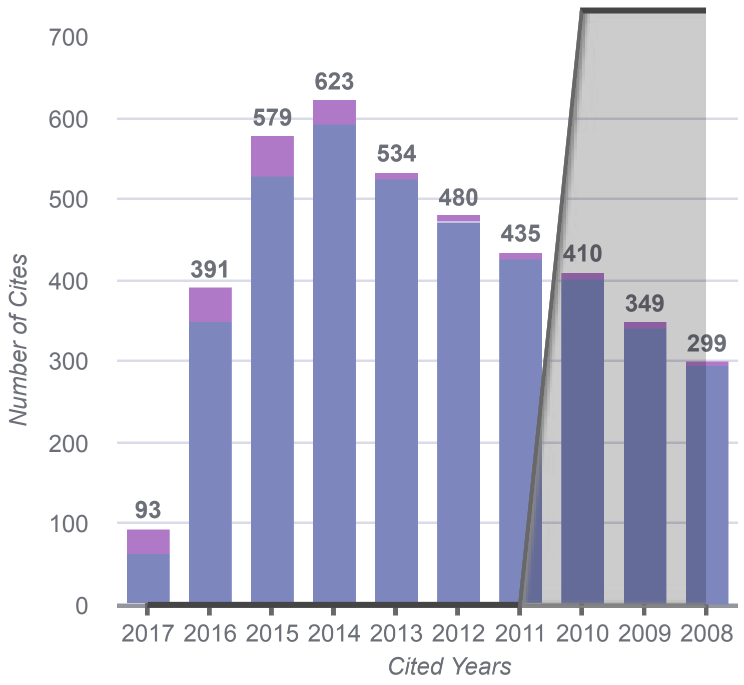

CITING JOURNAL GRAPH

The Citing Journal Graph shows the distribution (by cited year) of citations published in the Journal during the JCR year to items published in journals during the last 10 years.

The white/grey division indicates the citing half-life (if < 10.0). Half of the citations are to items that were published more recently than the citing half-life.

Downloaded on 2019/4/19 上午11:35:07 from Journal Citation Reports (©2019 Clarivate Analytics)

Citing Journal Data

Customize columns

	Impact	Cited Journal	All Yrs	2017	2016	2015	2014	2013	2012	2011	2010	2009	2008	R
		ALL Journals	6,863	93	391	579	623	534	480	435	410	349	299	2,
		ALL OTHERS (859)	859	8	55	82	70	62	64	41	44	27	36	
1	8.055	NEUROLOGY	424	3	16	23	33	24	30	20	13	22	14	
2	3.126	ACTA NEUROL SCAND	241	30	42	49	29	8	8	8	8	7	5	
3	6.239	STROKE	231	1	3	25	21	16	14	9	10	10	11	
4	5.067	EPILEPSIA	226	0	5	11	11	25	17	17	19	12	7	
5	7.144	J NEUROL NEUROSUR PS	173	2	11	12	9	11	6	5	10	8	9	
6	8.324	MOVEMENT DISORD	165	0	5	11	12	9	9	8	19	9	10	
7	5.280	MULT SCLER J	141	6	11	13	17	19	16	13	6	6	6	
8	3.783	J NEUROL	138	5	6	4	13	14	15	8	10	5	8	
9	27.144	LANCET NEUROL	134	1	7	13	17	10	7	11	10	17	10	
10	10.250	ANN NEUROL	118	1	3	5	8	4	4	12	13	4	3	
11	10.848	BRAIN	100	0	1	3	2	4	8	10	9	4	4	
12	79.260	NEW ENGL J MED	97	2	1	3	2	5	6	5	9	2	6	
13	2.600	EPILEPSY BEHAV	93	1	2	19	16	8	5	7	4	16	2	

Rows 1 - 15 of 561 *(use csv export to download the full table)*

Downloaded on 2019/4/19 上午11:35:07 from Journal Citation Reports (©2019 Clarivate Analytics)

Metric trend

Metric Trend

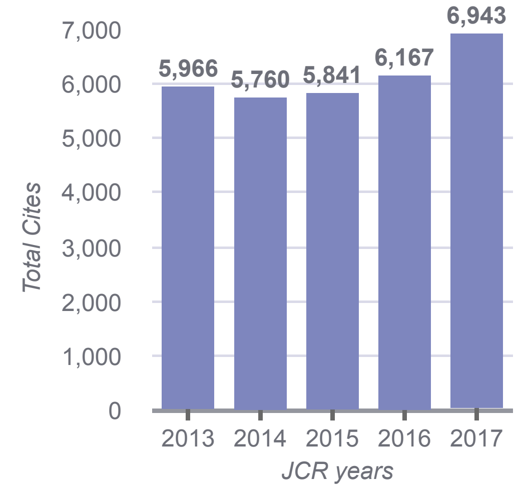

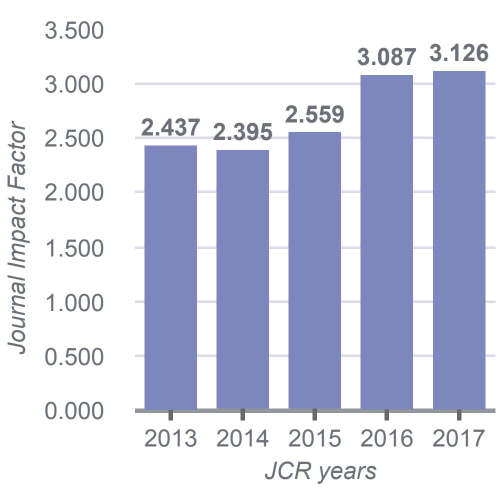

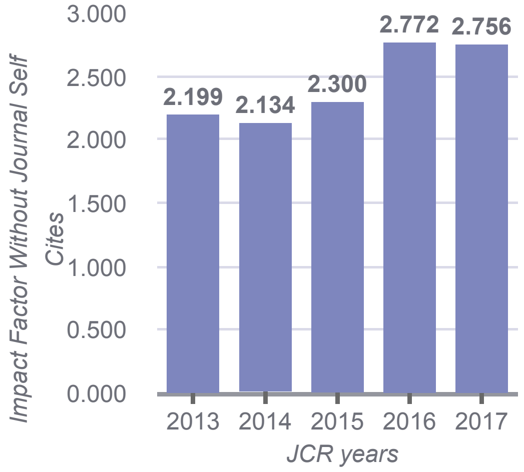

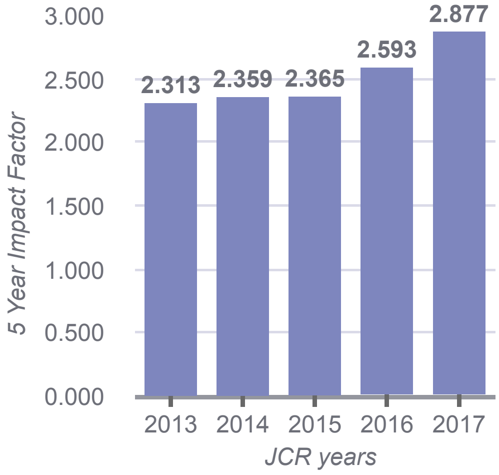

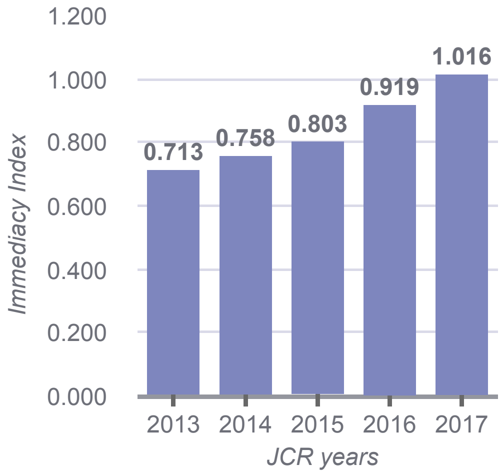

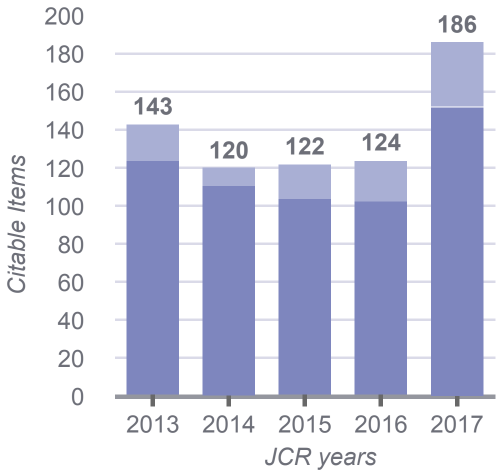

Downloaded on 2019/4/19 上午11:35:07 from Journal Citation Reports (©2019 Clarivate Analytics)

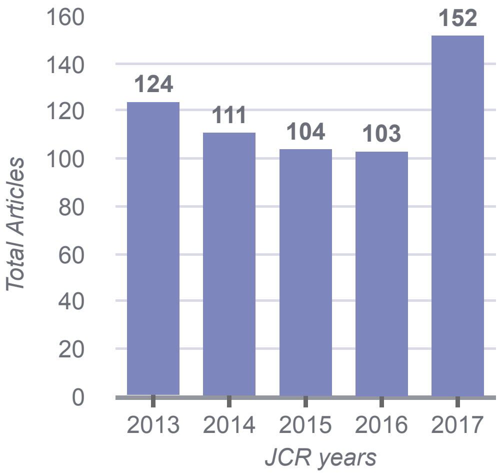

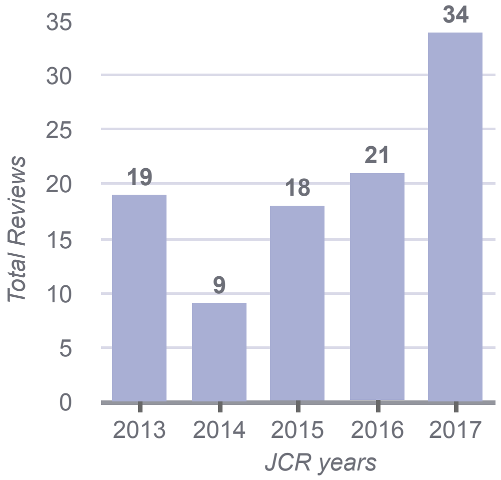

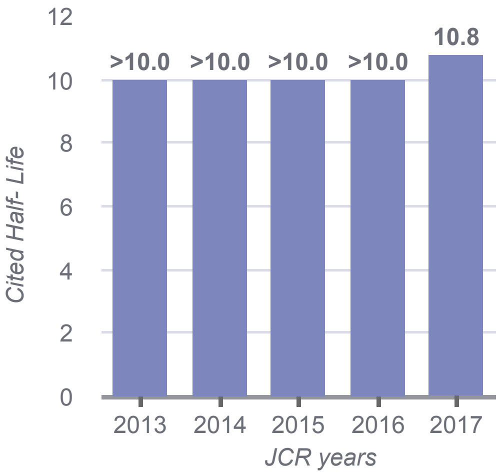

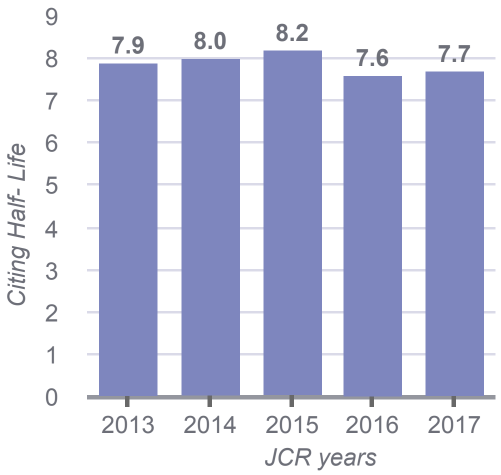

Downloaded on 2019/4/19 上午11:35:07 from Journal Citation Reports (©2019 Clarivate Analytics)

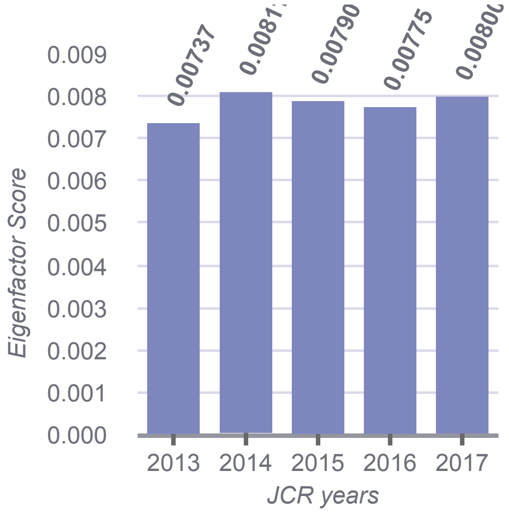

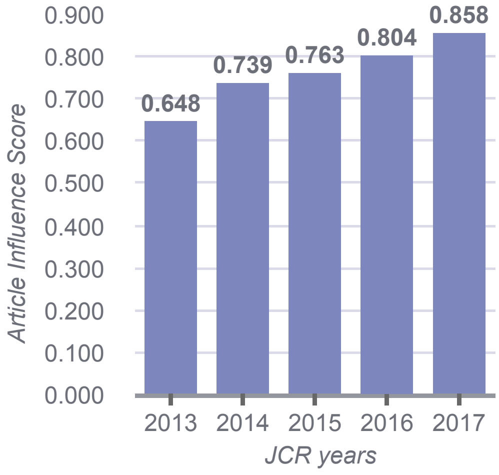

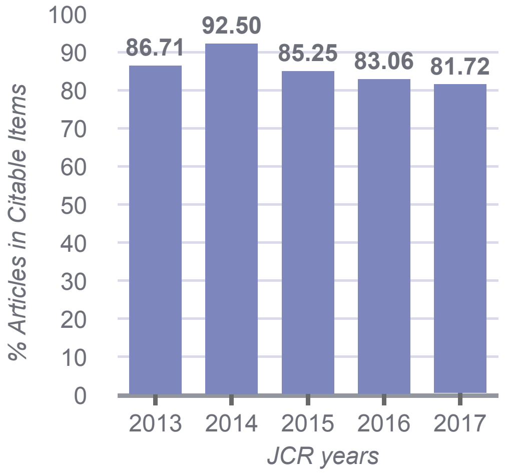

Downloaded on 2019/4/19 上午11:35:07 from Journal Citation Reports (©2019 Clarivate Analytics)

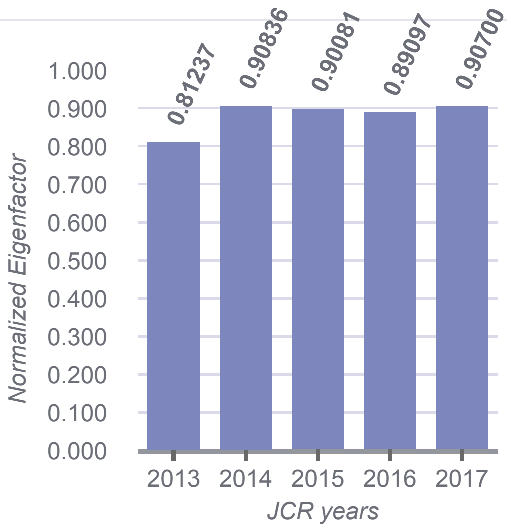

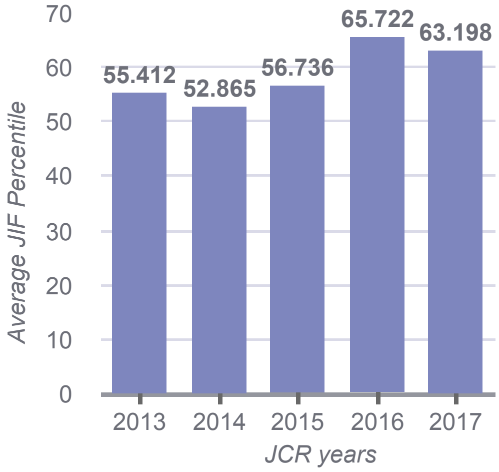

Downloaded on 2019/4/19 上午11:35:07 from Journal Citation Reports (©2019 Clarivate Analytics)

These data summarize the characteristics of the journal's published content for the most recent three years, that is, 2017 and the two prior years, combined. This information is based on all listed authors and addresses. It is meant to be descriptive rather than comparative.

Contributions by country/region

country	count
1. Sweden	69
2. USA	52
3. Italy	51
4. England	45
- GERMANY (FED REP GER)	45
6. Norway	40
7. Denmark	32
8. CHINA MAINLAND	31
9. Finland	23
10. Spain	21

Contributions by organizations

organization	count
1. UNIVERSITY OF GOTHENBURG	26
2. KAROLINSKA INSTITUTET	20
3. UNIVERSITY OF LONDON	18
- UNIVERSITY OF OSLO	18
- UNIVERSITY OF COPENHAGEN	18
6. UNIVERSITY OF TURKU	16
- UNIVERSITY OF BERGEN	16
8. LUND UNIVERSITY	15
9. STAVANGER UNIV HOSP	14
10. SAHLGRENSKA UNIVERSITY HOSPITAL	13
- UMEA UNIVERSITY	13

Downloaded on 2019/4/19 上午11:35:07 from Journal Citation Reports (©2019 Clarivate Analytics)
