# Supplementary material for: Emerging trends and knowledge structure of epilepsy during pregnancy research for 2000–2018: a bibliometric analysis
Source: PeerJ. 2019 Jun 7;7:e7115. doi: 10.7717/peerj.7115 (PMC6557303; doi:10.7717/peerj.7115)
Supplement: Supplemental Information 4 [file peerj-07-7115-s004.zip › 7/5. InCites Journal Citation Reports (BRAIN RESEARCH).pdf]

2017 Journal Performance Data for: BRAIN RESEARCH

ISSN: 0006-8993
eISSN: 1872-6240
ELSEVIER SCIENCE BV
PO BOX 211, 1000 AE AMSTERDAM, NETHERLANDS
NETHERLANDS

TITLES
ISO: Brain Res.
JCR Abbrev: BRAIN RES

LANGUAGES
English

CATEGORIES
NEUROSCIENCES - SCIE

PUBLICATION FREQUENCY
24 issues/year

Downloaded on 2019/4/19 上午11:36:28 from Journal Citation Reports (©2019 Clarivate Analytics)

Current Year

The data in the two graphs below and in the Journal Impact Factor calculation panels represent citation activity in 2017 to items published in the journal in the prior two years. They detail the components of the Journal Impact Factor. Use the "All Years" tab to access key metrics and additional data for the current year and all prior years for this journal.

2017 Journal Impact Factor & percentile rank in category for: BRAIN RESEARCH

3.125
2017 Journal Impact Factor

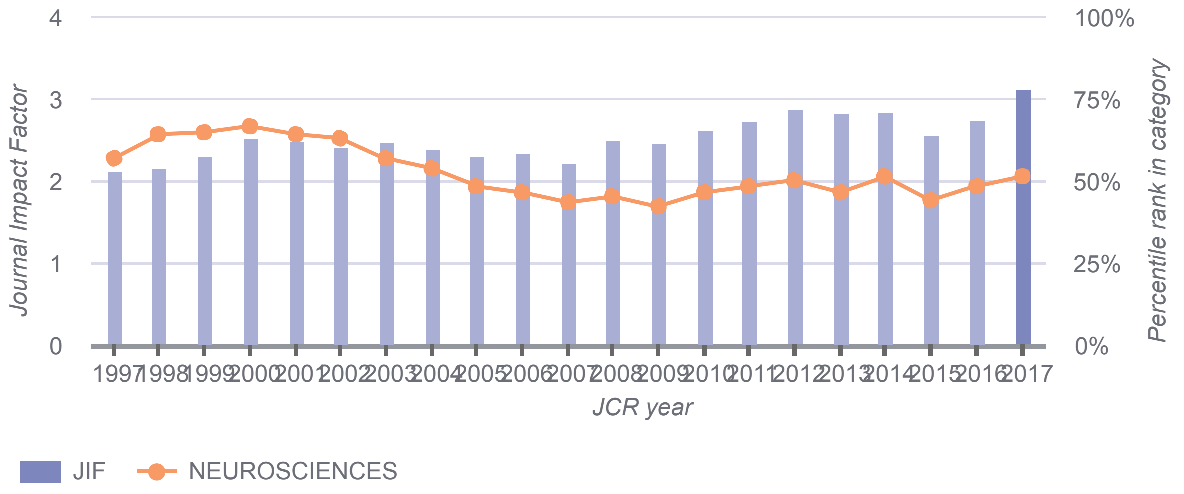

2017 JIF Citation Distribution for: BRAIN RESEARCH

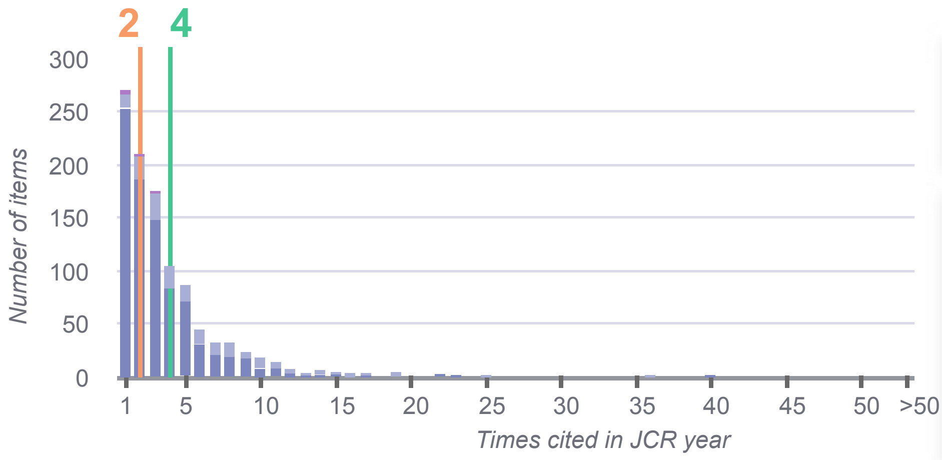

134
Unlinked citations

0
Times Cited

- **237** articles
- **18** reviews
- **19** other

Downloaded on 2019/4/19 上午11:36:28 from Journal Citation Reports (©2019 Clarivate Analytics)

Journal Impact Factor Calculation

$$\text{2017 Journal Impact Factor} = \frac{4{,}035}{1{,}291} = 3.125$$

How is Journal Impact Factor Calculated?

$$\text{JIF} = \frac{\text{Citations in 2017 to items published in } \mathbf{2015\ (2{,}293) + 2016\ (1{,}742)}}{\text{Number of citable items in } \mathbf{2015\ (675) + 2016\ (616)}} = \frac{4{,}035}{1{,}291}$$

Downloaded on 2019/4/19 上午11:36:28 from Journal Citation Reports (©2019 Clarivate Analytics)

Journal Impact Factor contributing items

Citable items in 2016 and 2015 (1,291)

TITLE	CITATIONS COUNTED TOWARDS JIF
Macrophage activation and its role in repair and pathology after spinal cord injury<br>By: Gensel, John C.; Zhang, Bei<br>**Volume: 1619 Page: 1-11 Accession number: WOS:000360776000001**<br>**Document Type: Article**	40
Regulation of hippocampal synaptic plasticity by BDNF<br>By: Leal, Graciano; Afonso, Pedro M.; Salazar, Ivan L.; Duarte, Carlos B.<br>**Volume: 1621 Page: 82-101 Accession number: WOS:000361403400009**<br>**Document Type: Review**	36
Prion-like domains as epigenetic regulators, scaffolds for subcellular organization, and drivers of neurodegenerative disease<br>By: March, Zachary M.; King, Oliver D.; Shorter, James<br>**Volume: 1647 Page: 9-18 Accession number: WOS:000383315200002**<br>**Document Type: Review**	25
How microglia kill neurons<br>By: Brown, Guy C.; Vilalta, Anna<br>**Volume: 1628 Page: 288-297 Accession number: WOS:000367412300006**<br>**Document Type: Article**	23
Long-term potentiation and the role of N-methyl-D-aspartate receptors<br>By: Volianskis, Arturas; France, Grace; Jensen, Morten S.; Bortolotto, Zuner A.; Jane, David E.; et al.<br>**Volume: 1621 Page: 5-16 Accession number: WOS:000361403400002**<br>**Document Type: Article**	22
Nestin overexpression promotes the embryonic development of heart and brain through the regulation of cell proliferation<br>By: Liu, Jinkai; Ji, Xiaozhen; Li, Zhenlin; Zheng, Hua; Zheng, Wenhong; et al.<br>**Volume: 1610 Page: 1-11 Accession number: WOS:000355714400001**<br>**Document Type: Article**	22
Matrix metalloproteinases as therapeutic targets for stroke<br>By: Yang, Yi; Rosenberg, Gary A.<br>**Volume: 1623 Page: 30-38 Accession number: WOS:000362144600004**<br>**Document Type: Review**	19

Downloaded on 2019/4/19 上午11:36:28 from Journal Citation Reports (©2019 Clarivate Analytics)

Citations in 2017 (4,035)

TITLE	CITATIONS COUNTED TOWARDS JIF
SCIENTIFIC REPORTS	137
PLOS ONE	71
BRAIN RESEARCH	65
NEUROSCIENCE	58
BEHAVIOURAL BRAIN RESEARCH	52
JOURNAL OF NEUROSCIENCE	51
FRONTIERS IN MOLECULAR NEUROSCIENCE	45
NEUROSCIENCE AND BIOBEHAVIORAL REVIEWS	43
NEUROSCIENCE LETTERS	43
ONCOTARGET	42

Downloaded on 2019/4/19 上午11:36:28 from Journal Citation Reports (©2019 Clarivate Analytics)

Key Indicators 2017

IMPACT METRICS		INFLUENCE METRICS		SOURCE METRICS	
**Total Cites**	55,731	**Eigenfactor Score**	0.03900	**Citable Items**	366
**Journal Impact Factor**	3.125	**Article Influence Score**	0.861	**% Articles in Citable Items**	91.80
**5 Year Impact Factor**	2.929	**Normalized Eigenfactor**	4.53000	**Average JIF Percentile**	51.916
**Immediacy Index**	0.664			**Cited Half-Life**	13.7
**Impact Factor Without Journal Self Cites**	3.075			**Citing Half-Life**	9.3

Source data

Journal source data 2017

	Articles	Reviews	Combined(C)	Other(O)	Percentage(C/(C+O))
**Number in JCR Year 2017 (A)**	336	30	366	5	98%
**Number of References (B)**	17,852	3,386	21,238	3	99%
**Ratio (B/A)**	53.1	112.9	58.0	0.6	

Downloaded on 2019/4/19 上午11:36:28 from Journal Citation Reports (©2019 Clarivate Analytics)

Box plot

Category Box Plot 2017

Category Box Plot

The category box plot depicts the distribution of Impact Factors for all journals in the category. The horizontal line that forms the top of the box is the 75th percentile (Q1). The horizontal line that forms the bottom is the 25th percentile (Q3). The horizontal line that intersects the box is the median Impact Factor for the category. Horizontal lines above and below the box, called whiskers, represent maximum and minimum values.
The top whisker is the smaller of the following two values:
the maximum Impact Factor (IF)
Q1 IF + 3.5(Q1 IF - Q3 IF)
The bottom whisker is the larger of the following two values:
the minimum Impact Factor (IF)
Q1 IF - 3.5(Q1 IF - Q3 IF)
Box Plots are provided for the current JCR year for each of the categories in which the journal is indexed.

BRAIN RES, IF: 3.125

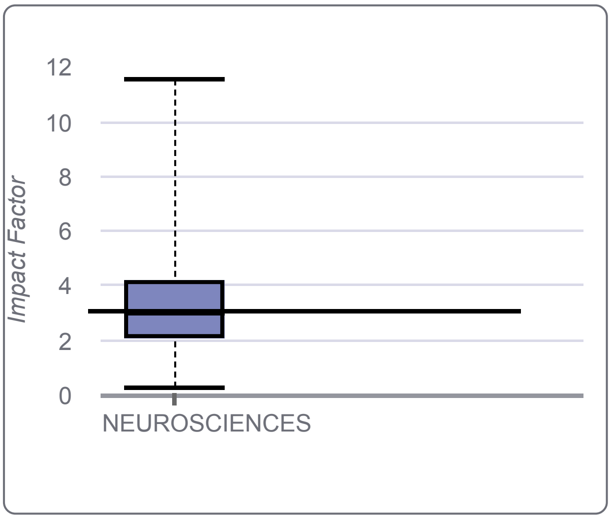

Downloaded on 2019/4/19 上午11:36:28 from Journal Citation Reports (©2019 Clarivate Analytics)

Rank

Rank 2017

JCR Impact Factor

JCR Year	NEUROSCIENCES		
	Rank	Quartile	JIF Percentile
2017	126/261	Q2	51.916
2016	134/259	Q3	48.456
2015	143/256	Q3	44.336
2014	122/252	Q2	51.786
2013	135/252	Q3	46.627
2012	126/252	Q2	50.198
2011	126/244	Q3	48.566
2010	128/239	Q3	46.653
2009	133/231	Q3	42.641
2008	121/221	Q3	45.475
2007	119/211	Q3	43.839
2006	107/200	Q3	46.750
2005	103/200	Q3	48.750
2004	91/198	Q2	54.293
2003	85/198	Q2	57.323
2002	73/197	Q2	63.198
2001	71/198	Q2	64.394
2000	67/203	Q2	67.241
1999	70/201	Q2	65.423
1998	72/202	Q2	64.604

Downloaded on 2019/4/19 上午11:36:28 from Journal Citation Reports (©2019 Clarivate Analytics)

Downloaded on 2019/4/19 上午11:36:28 from Journal Citation Reports (©2019 Clarivate Analytics)

ESI Total Citations 2017

Rank

JCR Year	NEUROSCIENCE & BEHAVIOR
2017	7/346-Q1
2016	6/345-Q1
2015	6/344-Q1
2014	6/337-Q1
2013	5/339-Q1

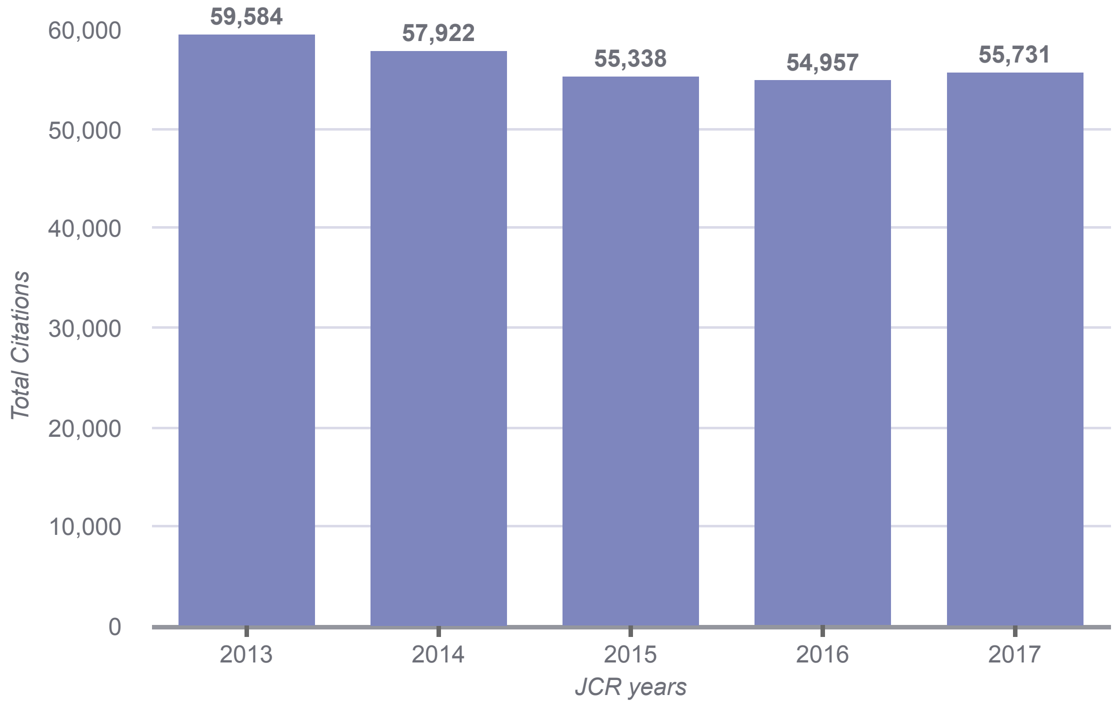

Downloaded on 2019/4/19 上午11:36:28 from Journal Citation Reports (©2019 Clarivate Analytics)

Cited Journal Data

Cited Half-Life Data

Customize columns

Cited Year	2017	2016	2015	2014	2013	2012	2011	2010	2009	2008	20(
#Cites from 2017	243	1,742	2,293	1,616	1,727	2,201	2,471	3,065	2,558	2,484	3
Cumulative %	0.44%	3.56%	7.68%	10.58%	13.67%	17.62%	22.06%	27.56%	32.15%	36.60%	10(

Cited Journal Graph 2017

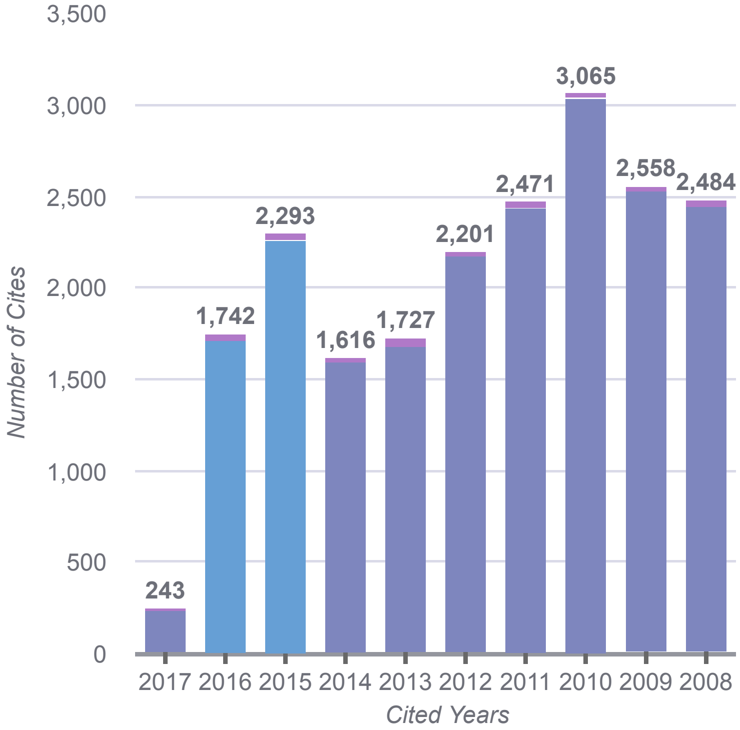

CITED JOURNAL GRAPH

The Cited Journal Graph shows the distribution (by cited year) of citations published in journals during the JCR year to items published in the Journal during the last 10 years.

The white/grey division indicates the cited half-life (if < 10.0). Half of the citations are to items that were published more recently than the cited half-life.

The two light-blue columns indicate citations used to calculate the Impact Factor (always the 2nd and 3rd columns).

Downloaded on 2019/4/19 上午11:36:28 from Journal Citation Reports (©2019 Clarivate Analytics)

Cited Journal Data

Customize columns

	Impact	Citing Journal	All Yrs	2017	2016	2015	2014	2013	2012	2011	2010
		ALL Journals	55,731	243	1,742	2,293	1,616	1,727	2,201	2,471	3,065
		ALL OTHERS (1318)	1,318	10	45	68	52	29	74	77	75
1	4.122	SCI REP-UK	1,470	6	64	73	42	46	68	74	93
2	2.766	PLOS ONE	1,119	5	31	40	36	38	49	53	78
3	3.382	NEUROSCIENCE	960	3	25	33	16	28	33	35	41
4	3.173	BEHAV BRAIN RES	807	1	22	30	17	24	29	37	56
5	5.971	J NEUROSCI	684	3	18	33	14	14	17	18	21
6	5.076	MOL NEUROBIOL	675	0	11	30	31	32	34	39	51
7	4.249	NEUROPHARMACOLOGY	623	2	11	21	16	14	11	23	35
8	3.125	BRAIN RES	621	9	30	35	22	42	22	34	27
9	8.037	NEUROSCI BIOBEHAV R	518	0	17	26	12	16	18	20	24
10	3.400	J COMP NEUROL	511	0	3	2	2	3	8	14	11
11	3.877	FRONT NEUROSCI-SWITZ	494	5	18	16	9	9	23	17	39
12	2.502	J NEUROPHYSIOL	457	1	11	9	3	6	2	2	8
13	3.687	INT J MOL SCI	454	4	21	21	16	18	26	19	29
14	2.159	NEUROSCI LETT	447	3	14	29	21	15	20	21	16
15	4.300	FRONT CELL NEUROSCI	446	5	19	9	9	10	16	18	17
16	4.231	BRAIN STRUCT FUNCT	420	1	6	11	4	4	13	11	16

Rows 1 - 18 of 2,513 *(use csv export to download the full table)*

Downloaded on 2019/4/19 上午11:36:28 from Journal Citation Reports (©2019 Clarivate Analytics)

Citing Journal Data

Citing Half-Life Data

Customize columns

Citing Year	2017	2016	2015	2014	2013	2012	2011	2010	2009	2008	20
#Cites from 2017	193	916	1,400	1,447	1,455	1,323	1,251	1,147	1,140	1,024	
Cumulative %	0.91%	5.22%	11.81%	18.62%	25.47%	31.70%	37.59%	42.99%	48.36%	53.18%	10

Citing Journal Graph 2017

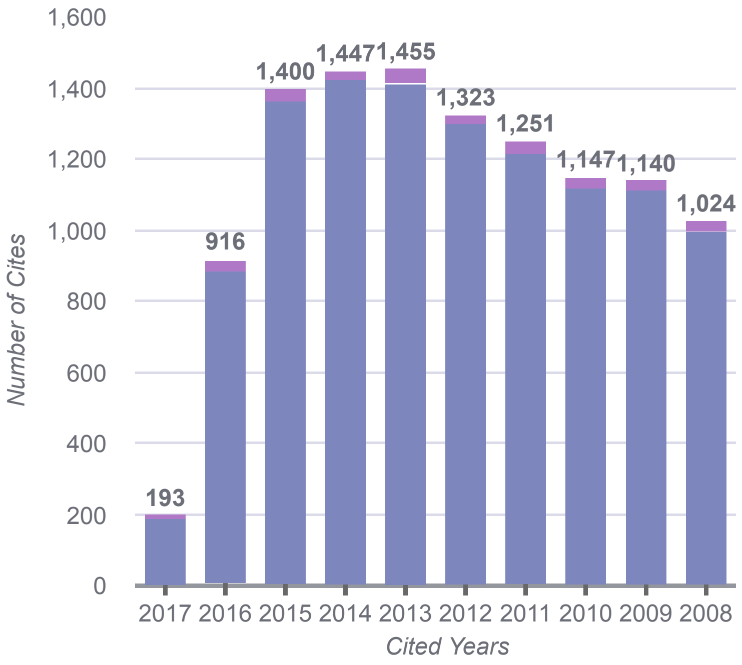

CITING JOURNAL GRAPH

The Citing Journal Graph shows the distribution (by cited year) of citations published in the Journal during the JCR year to items published in journals during the last 10 years.

The white/grey division indicates the citing half-life (if < 10.0). Half of the citations are to items that were published more recently than the citing half-life.

Downloaded on 2019/4/19 上午11:36:28 from Journal Citation Reports (©2019 Clarivate Analytics)

Citing Journal Data

Customize columns

	Impact	Cited Journal	All Yrs	2017	2016	2015	2014	2013	2012	2011	2010	2009	20
		ALL Journals	21,241	193	916	1,400	1,447	1,455	1,323	1,251	1,147	1,140	1,[illegible]
		ALL OTHERS (1383)	1,383	23	91	115	113	101	84	67	67	65	
1	5.971	J NEUROSCI	841	3	15	26	21	37	49	60	55	49	
2	3.125	BRAIN RES	621	9	30	35	22	42	22	34	27	26	
3	9.504	P NATL ACAD SCI USA	452	1	6	16	16	16	28	23	34	32	
4	3.382	NEUROSCIENCE	405	1	18	38	20	31	23	30	21	22	
5	2.766	PLOS ONE	349	0	19	37	65	78	58	40	27	12	
6	14.319	NEURON	333	1	7	19	23	31	14	25	26	14	
7	41.577	NATURE	316	0	8	13	15	16	15	30	15	12	
8	5.426	NEUROIMAGE	306	2	8	6	27	15	24	20	23	25	
9	41.058	SCIENCE	273	0	2	0	11	13	7	3	7	11	
10	3.400	J COMP NEUROL	257	0	3	5	7	6	5	3	16	9	
11	4.011	J BIOL CHEM	244	1	2	5	5	7	10	12	5	13	
12	2.159	NEUROSCI LETT	217	1	11	13	13	9	13	9	11	14	
13	2.832	EUR J NEUROSCI	211	1	2	8	13	9	5	13	9	14	
14	3.173	BEHAV BRAIN RES	209	3	9	12	21	10	20	18	9	20	

Rows 1 - 16 of 1,248 *(use csv export to download the full table)*

Downloaded on 2019/4/19 上午11:36:28 from Journal Citation Reports (©2019 Clarivate Analytics)

Metric trend

Metric Trend

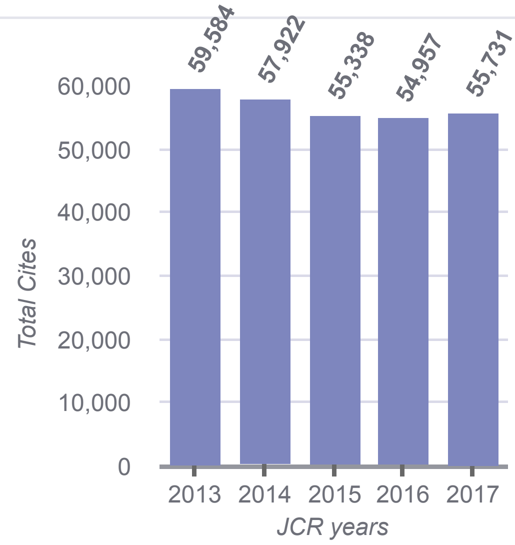

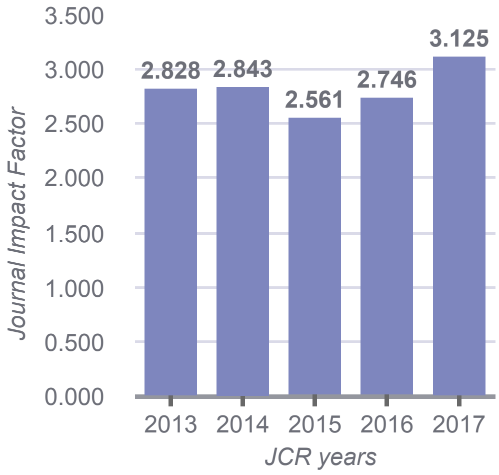

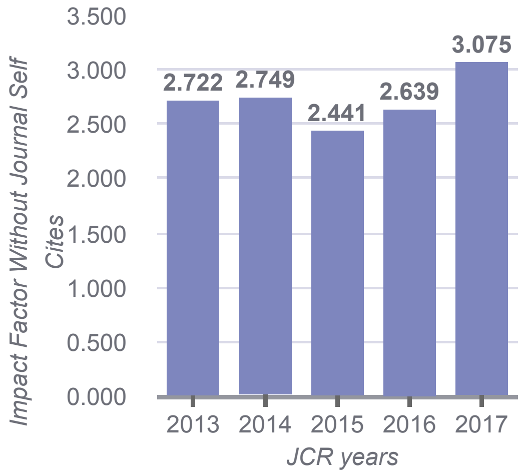

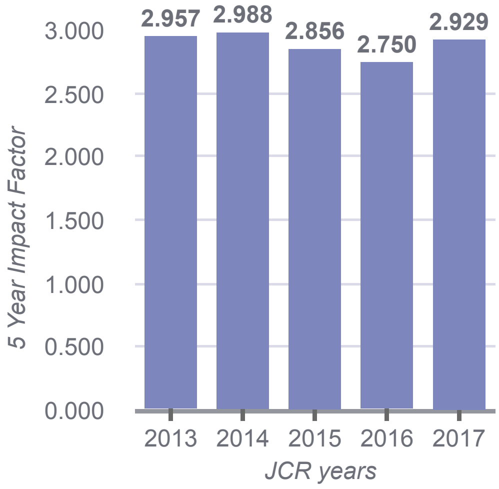

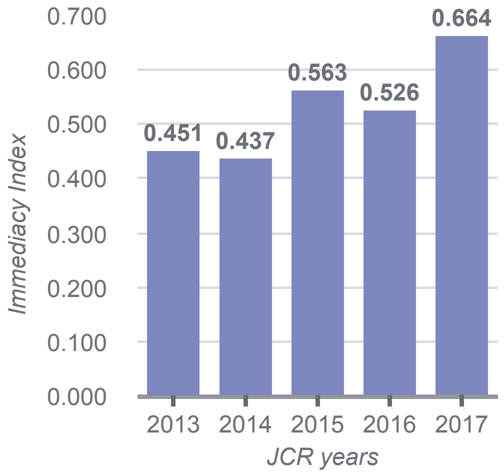

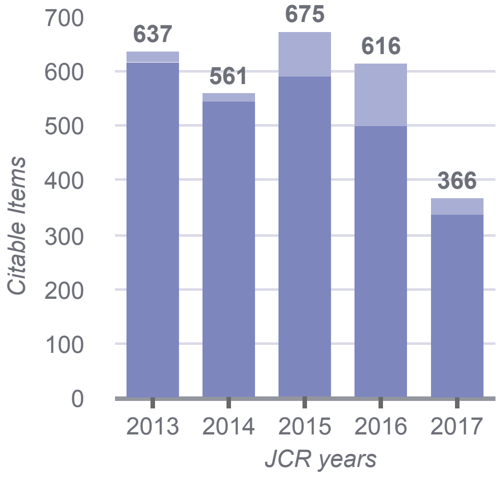

Downloaded on 2019/4/19 上午11:36:28 from Journal Citation Reports (©2019 Clarivate Analytics)

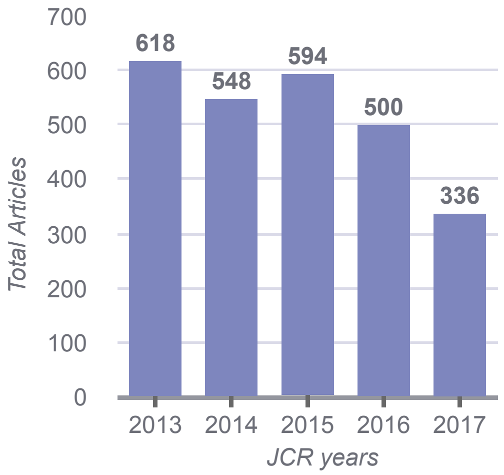

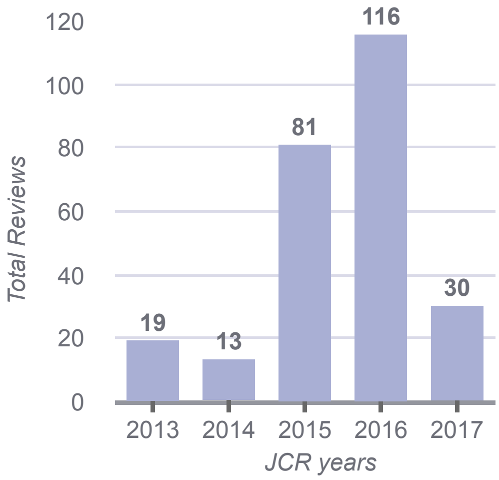

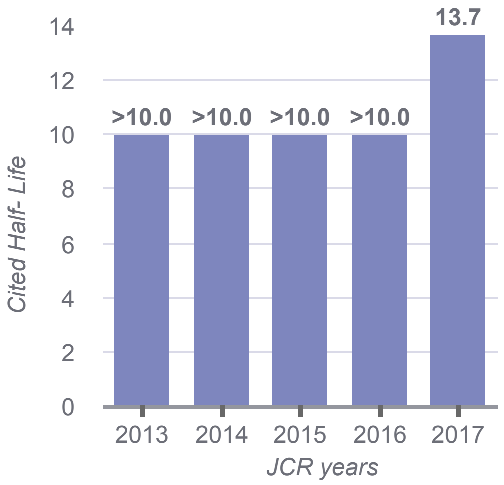

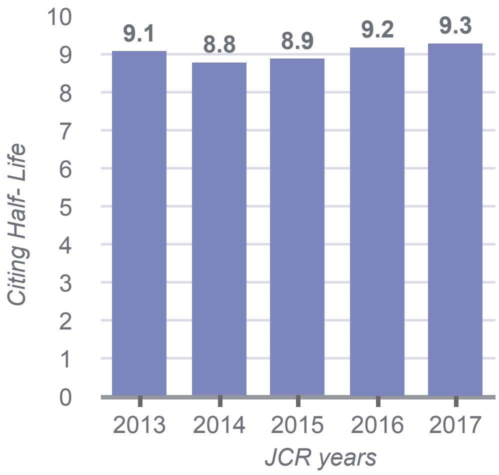

Downloaded on 2019/4/19 上午11:36:28 from Journal Citation Reports (©2019 Clarivate Analytics)

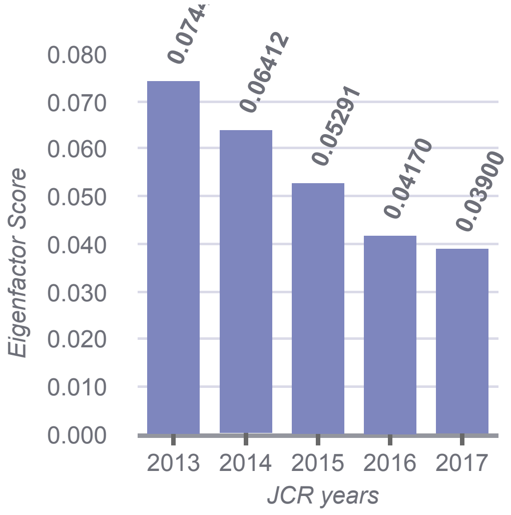

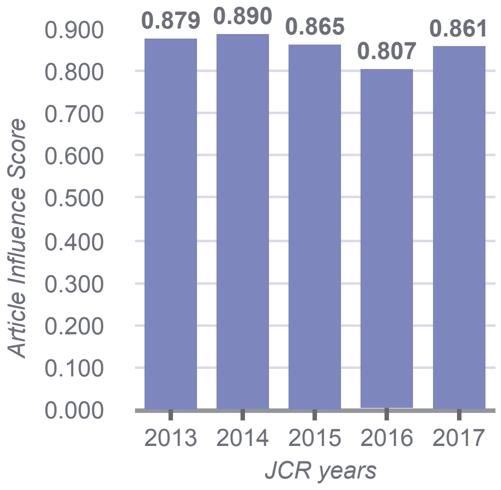

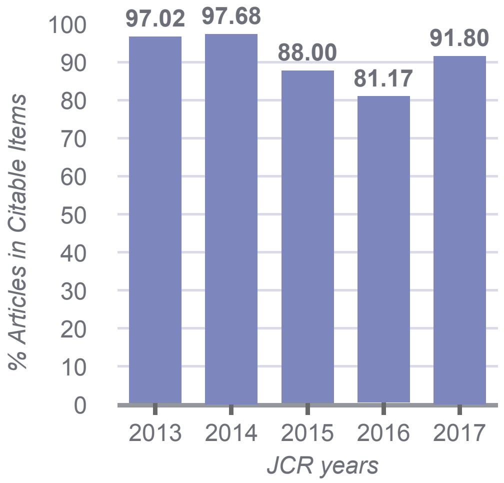

Downloaded on 2019/4/19 上午11:36:28 from Journal Citation Reports (©2019 Clarivate Analytics)

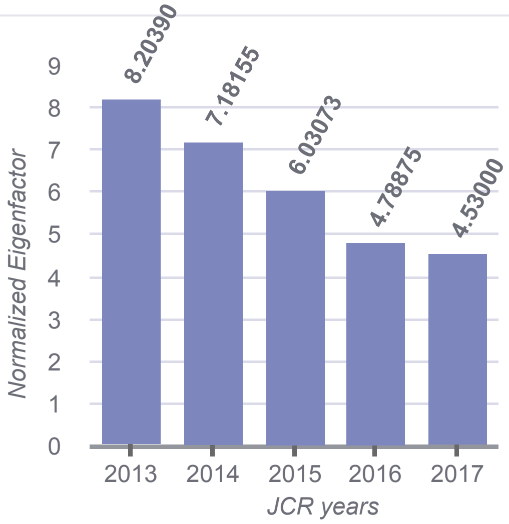

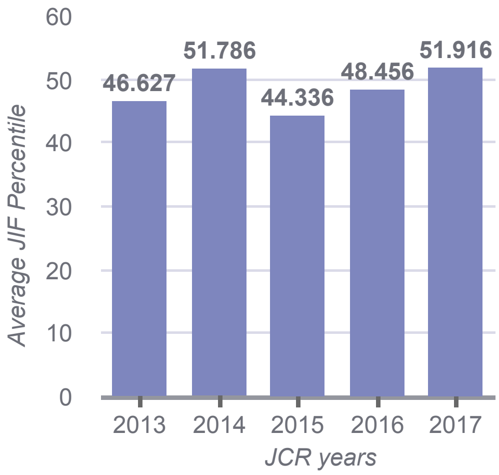

Downloaded on 2019/4/19 上午11:36:28 from Journal Citation Reports (©2019 Clarivate Analytics)

These data summarize the characteristics of the journal's published content for the most recent three years, that is, 2017 and the two prior years, combined. This information is based on all listed authors and addresses. It is meant to be descriptive rather than comparative.

Contributions by country/region

country	count
1. USA	606
2. CHINA MAINLAND	417
3. Japan	145
4. GERMANY (FED REP GER)	109
5. Canada	75
6. England	62
7. Brazil	60
8. South Korea	53
9. France	42
10. Australia	37
- Italy	37

Contributions by organizations

organization	count
1. UNIVERSITY OF CALIFORNIA SYSTEM	57
2. HARVARD UNIVERSITY	35
3. PENNSYLVANIA COMMONWEALTH SYSTEM OF HIGHER EDUCATION (PCSHE)	33
4. CAPITAL MEDICAL UNIVERSITY	28
5. UNIVERSITY OF TEXAS SYSTEM	24
6. UNIVERSIDADE DE SAO PAULO	22
7. UNIVERSITY OF LONDON	21
8. CENTRE NATIONAL DE LA RECHERCHE SCIENTIFIQUE (CNRS)	20
- JOHNS HOPKINS UNIVERSITY	20
- HUAZHONG UNIVERSITY OF SCIENCE & TECHNOLOGY	20
- VA BOSTON HEALTHCARE SYSTEM	20

Downloaded on 2019/4/19 上午11:36:28 from Journal Citation Reports (©2019 Clarivate Analytics)
