# Supplementary material for: Emerging trends and knowledge structure of epilepsy during pregnancy research for 2000–2018: a bibliometric analysis
Source: PeerJ. 2019 Jun 7;7:e7115. doi: 10.7717/peerj.7115 (PMC6557303; doi:10.7717/peerj.7115)
Supplement: Supplemental Information 4 [file peerj-07-7115-s004.zip › 7/6. InCites Journal Citation Reports(SEIZURE-EUROPEAN JOURNAL OF EPILEPSY).pdf]

2017 Journal Performance Data for: SEIZURE-EUROPEAN JOURNAL OF EPILEPSY

ISSN: 1059-1311
eISSN: 1532-2688
W B SAUNDERS CO LTD
32 JAMESTOWN RD, LONDON NW1 7BY, ENGLAND
ENGLAND

TITLES
ISO: Seizure
JCR Abbrev: SEIZURE-EUR J EPILEP

LANGUAGES
English

CATEGORIES
CLINICAL NEUROLOGY - SCIE

NEUROSCIENCES - SCIE

PUBLICATION FREQUENCY
10 issues/year

Downloaded on 2019/4/19 上午11:37:39 from Journal Citation Reports (©2019 Clarivate Analytics)

Current Year

The data in the two graphs below and in the Journal Impact Factor calculation panels represent citation activity in 2017 to items published in the journal in the prior two years. They detail the components of the Journal Impact Factor. Use the "All Years" tab to access key metrics and additional data for the current year and all prior years for this journal.

2017 Journal Impact Factor & percentile rank in category for: SEIZURE-EUROPEAN JOURNAL OF EPILEPSY

2.839
2017 Journal Impact Factor

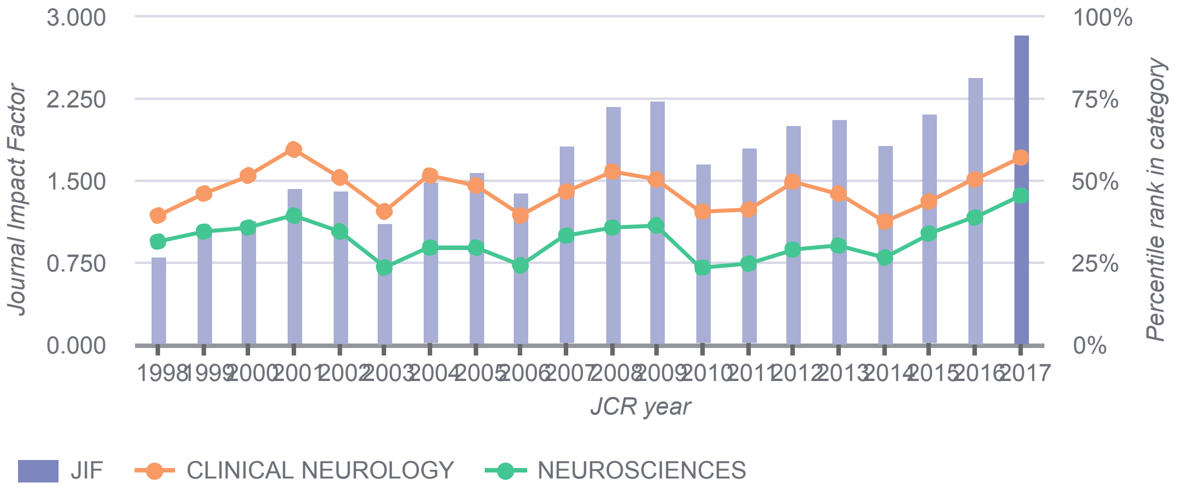

2017 JIF Citation Distribution for: SEIZURE-EUROPEAN JOURNAL OF EPILEPSY

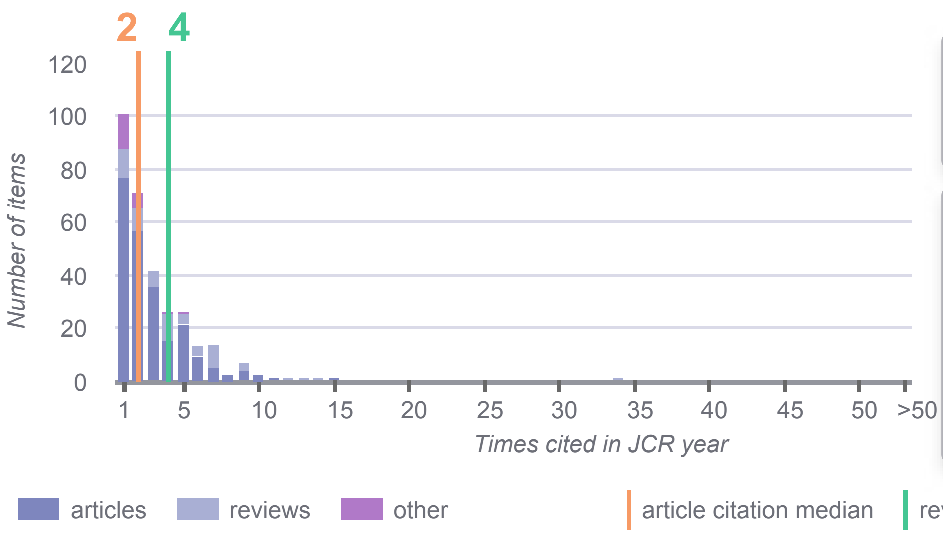

38
Unlinked citations

0
Times Cited

- **59** articles
- **7** reviews
- **39** other

Downloaded on 2019/4/19 上午11:37:39 from Journal Citation Reports (©2019 Clarivate Analytics)

Journal Impact Factor Calculation

$$\text{2017 Journal Impact Factor} = \frac{1{,}008}{355} = 2.839$$

How is Journal Impact Factor Calculated?

$$\text{JIF} = \frac{\text{Citations in 2017 to items published in } \mathbf{2015\ (649) + 2016\ (359)}}{\text{Number of citable items in } \mathbf{2015\ (208) + 2016\ (147)}} = \frac{1{,}008}{355}$$

Downloaded on 2019/4/19 上午11:37:39 from Journal Citation Reports (©2019 Clarivate Analytics)

Journal Impact Factor contributing items

Citable items in 2016 and 2015 (355)

TITLE	CITATIONS COUNTED TOWARDS JIF
Wavelet-based EEG processing for computer-aided seizure detection and epilepsy diagnosis<br>By: Faust, Oliver; Acharya, U. Rajendra; Adeli, Hojjat; Adeli, Amir<br>**Volume: 26 Page: 56-64 Accession number: WOS:000353003800011**<br>**Document Type: Review**	34
CBD-enriched medical cannabis for intractable pediatric epilepsy The current Israeli experience<br>By: Tzadok, Michal; Granot, Dorit; Dor, Michael; Lerman-Sagie, Tali; Ben-Zeev, Bruria; et al.<br>**Volume: 35 Page: 41-44 Accession number: WOS:000371839000007**<br>**Document Type: Article**	15
Prevalence of epilepsy among people with intellectual disabilities: A systematic review<br>By: Robertson, Janet; Hatton, Chris; Emerson, Eric; Baines, Susannah<br>**Volume: 29 Page: 46-62 Accession number: WOS:000357767800009**<br>**Document Type: Review**	14
Interactions between antiepileptic drugs and hormones<br>By: Svalheim, Sigrid; Sveberg, Line; Mochol, Monika; Tauboll, Erik<br>**Volume: 28 Page: 12-17 Accession number: WOS:000355705400003**<br>**Document Type: Review**	13
Intracranial evaluation and laser ablation for epilepsy with periventricular nodular heterotopia<br>By: Thompson, Stephen A.; Kalamangalam, Giridhar P.; Tandon, Nitin<br>**Volume: 41 Page: 211-216 Accession number: WOS:000384858200038**<br>**Document Type: Review**	12
Efficacy and safety of lacosamide as first add-on or later adjunctive treatment for uncontrolled partial-onset seizures: A multicentre open-label trial<br>By: Zadeh, Wendy Waldman; Escartin, Antonio; Byrnes, William; Tennigkeit, Frank; Borghs, Simon; et al.<br>**Volume: 31 Page: 72-79 Accession number: WOS:000361774200013**<br>**Document Type: Article**	11
Retrospective case series of the clinical features, management and outcomes of patients with autoimmune epilepsy<br>By: Dubey, Divyanshu; Samudra, Niyatee; Gupta, Puneet; Agostini, Mark; Ding, Kan; et al.<br>**Volume: 29 Page: 143-147 Accession number: WOS:000357767800023**<br>**Document Type: Article**	10

Downloaded on 2019/4/19 上午11:37:39 from Journal Citation Reports (©2019 Clarivate Analytics)

Citations in 2017 (1,008)

TITLE	CITATIONS COUNTED TOWARDS JIF
EPILEPSY & BEHAVIOR	119
SEIZURE-EUROPEAN JOURNAL OF EPILEPSY	106
EPILEPSIA	67
EPILEPSY RESEARCH	33
FRONTIERS IN NEUROLOGY	21
CURRENT PHARMACEUTICAL DESIGN	17
ZEITSCHRIFT FUR EPILEPTOLOGIE	12
CLINICAL NEUROPHYSIOLOGY	11
CURRENT OPINION IN NEUROLOGY	10
EXPERT REVIEW OF NEUROTHERAPEUTICS	10

Downloaded on 2019/4/19 上午11:37:39 from Journal Citation Reports (©2019 Clarivate Analytics)

Key Indicators 2017

IMPACT METRICS		INFLUENCE METRICS		SOURCE METRICS	
**Total Cites**	5,113	**Eigenfactor Score**	0.00800	**Citable Items**	248
**Journal Impact Factor**	2.839	**Article Influence Score**	0.708	**% Articles in Citable Items**	70.97
**5 Year Impact Factor**	2.609	**Normalized Eigenfactor**	0.96300	**Average JIF Percentile**	51.446
**Immediacy Index**	0.702			**Cited Half-Life**	6.4
**Impact Factor Without Journal Self Cites**	2.540			**Citing Half-Life**	8.1

Source data

Journal source data 2017

	Articles	Reviews	Combined(C)	Other(O)	Percentage(C/(C+O))
**Number in JCR Year 2017 (A)**	176	72	248	14	94%
**Number of References (B)**	4,514	4,847	9,361	111	98%
**Ratio (B/A)**	25.6	67.3	37.7	7.9	

Downloaded on 2019/4/19 上午11:37:39 from Journal Citation Reports (©2019 Clarivate Analytics)

Box plot

Category Box Plot 2017

Category Box Plot

The category box plot depicts the distribution of Impact Factors for all journals in the category. The horizontal line that forms the top of the box is the 75th percentile (Q1). The horizontal line that forms the bottom is the 25th percentile (Q3). The horizontal line that intersects the box is the median Impact Factor for the category. Horizontal lines above and below the box, called whiskers, represent maximum and minimum values.
The top whisker is the smaller of the following two values:
the maximum Impact Factor (IF)
Q1 IF + 3.5(Q1 IF - Q3 IF)
The bottom whisker is the larger of the following two values:
the minimum Impact Factor (IF)
Q1 IF - 3.5(Q1 IF - Q3 IF)
Box Plots are provided for the current JCR year for each of the categories in which the journal is indexed.

SEIZURE-EUR J EPILEP, IF: 2.839

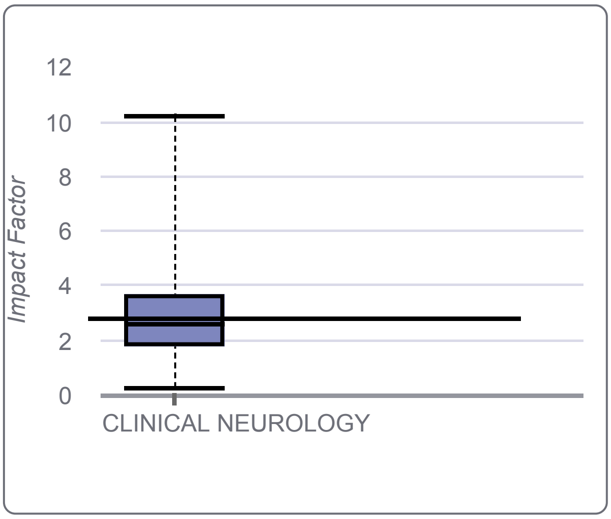

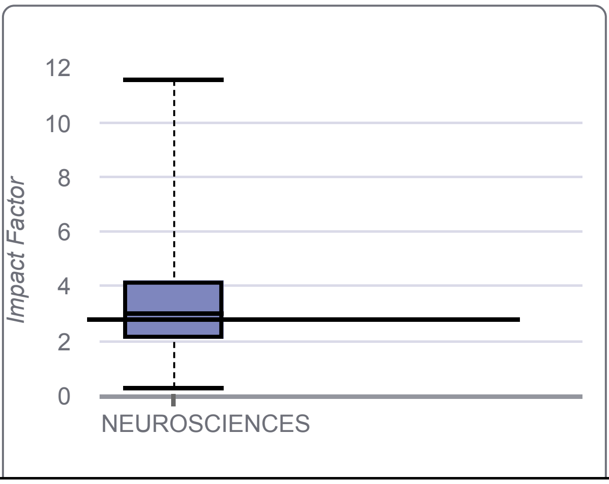

Downloaded on 2019/4/19 上午11:37:39 from Journal Citation Reports (©2019 Clarivate Analytics)

Rank

Rank 2017

JCR Impact Factor

JCR Year	CLINICAL NEUROLOGY			NEUROSCIENCES		
	Rank	Quartile	JIF Percentile	Rank	Quartile	JIF Percentile
2017	85/197	Q2	57.107	142/261	Q3	45.785
2016	97/194	Q2	50.258	159/259	Q3	38.803
2015	109/193	Q3	43.782	170/256	Q3	33.789
2014	120/192	Q3	37.760	186/252	Q3	26.389
2013	105/194	Q3	46.134	177/252	Q3	29.960
2012	97/193	Q3	50.000	179/252	Q3	29.167
2011	113/192	Q3	41.406	184/244	Q4	24.795
2010	110/185	Q3	40.811	184/239	Q4	23.222
2009	83/167	Q2	50.599	148/231	Q3	36.147
2008	74/156	Q2	52.885	143/221	Q3	35.520
2007	78/146	Q3	46.918	141/211	Q3	33.412
2006	90/147	Q3	39.116	153/200	Q4	23.750
2005	77/148	Q3	48.311	142/200	Q3	29.250
2004	68/140	Q2	51.786	140/198	Q3	29.545
2003	81/135	Q3	40.370	152/198	Q4	23.485
2002	68/138	Q2	51.087	130/197	Q3	34.264
2001	55/136	Q2	59.926	120/198	Q3	39.646
2000	67/137	Q2	51.460	131/203	Q3	35.714
1999	72/132	Q3	45.833	132/201	Q3	34.577
1998	76/125	Q3	39.600	139/202	Q3	31.436

Downloaded on 2019/4/19 上午11:37:39 from Journal Citation Reports (©2019 Clarivate Analytics)

ESI Total Citations 2017

Rank

JCR Year	NEUROSCIENCE & BEHAVIOR
2017	135/346-Q2
2016	149/345-Q2
2015	148/344-Q2
2014	154/337-Q2
2013	150/339-Q2

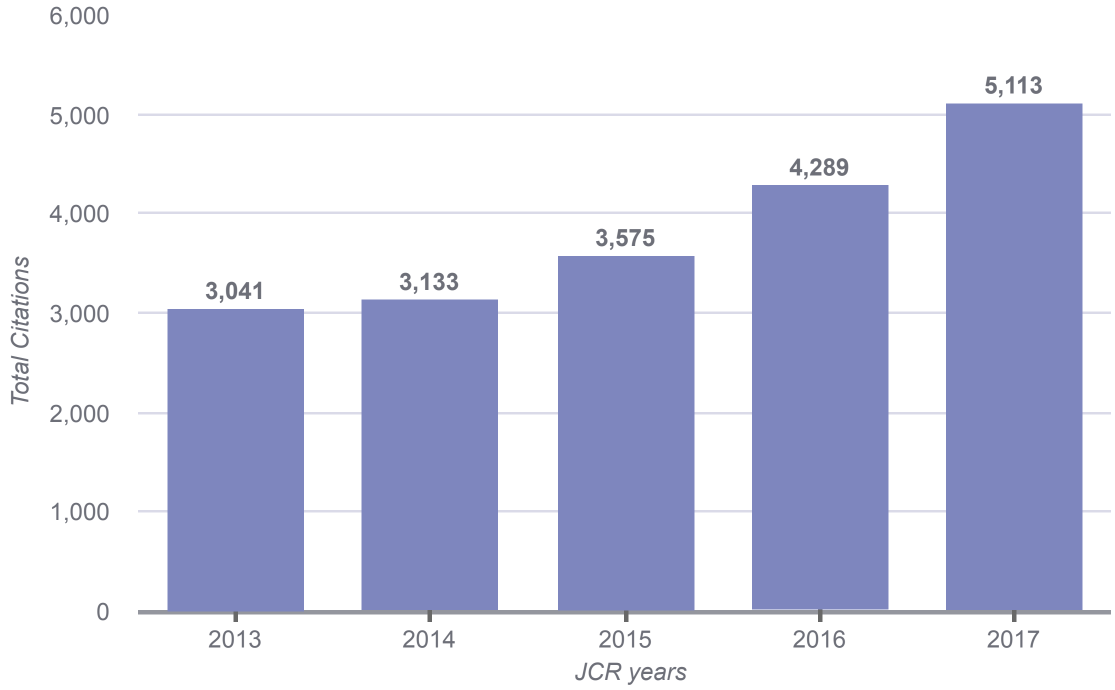

Downloaded on 2019/4/19 上午11:37:39 from Journal Citation Reports (©2019 Clarivate Analytics)

Cited Journal Data

Cited Half-Life Data

Customize columns

Cited Year	2017	2016	2015	2014	2013	2012	2011	2010	2009	2008	2
#Cites from 2017	174	359	649	407	414	378	429	270	281	205	
Cumulative %	3.40%	10.42%	23.12%	31.08%	39.17%	46.57%	54.96%	60.24%	65.73%	69.74%	1

Cited Journal Graph 2017

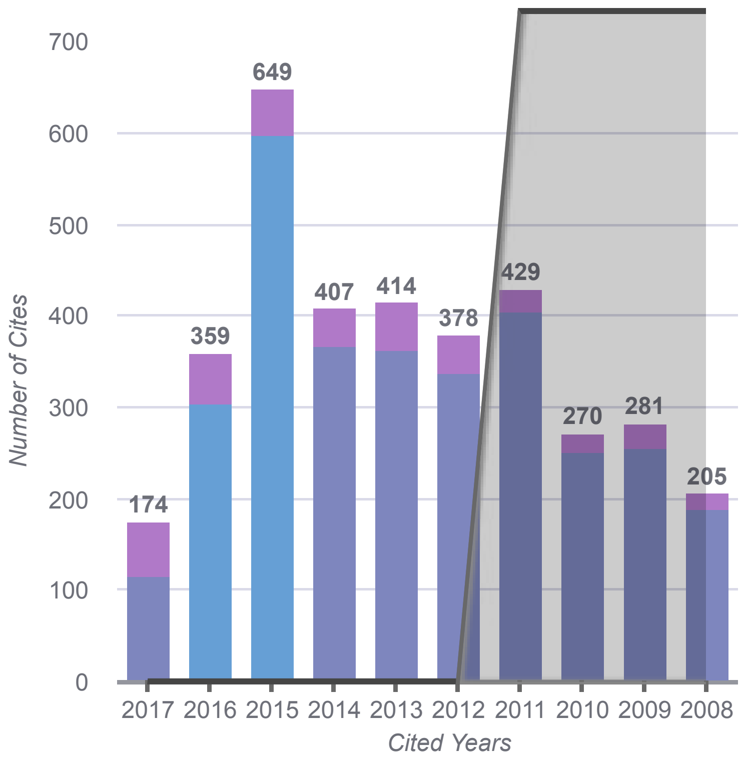

CITED JOURNAL GRAPH

The Cited Journal Graph shows the distribution (by cited year) of citations published in journals during the JCR year to items published in the Journal during the last 10 years.

The white/grey division indicates the cited half-life (if < 10.0). Half of the citations are to items that were published more recently than the cited half-life.

The two light-blue columns indicate citations used to calculate the Impact Factor (always the 2nd and 3rd columns).

Downloaded on 2019/4/19 上午11:37:39 from Journal Citation Reports (©2019 Clarivate Analytics)

Cited Journal Data

Customize columns

	Impact	Citing Journal	All Yrs	2017	2016	2015	2014	2013	2012	2011	2010	2009	2008	R
		ALL Journals	5,113	174	359	649	407	414	378	429	270	281	205	1,
		ALL OTHERS (477)	477	15	33	77	28	40	39	33	30	27	18	
1	2.600	EPILEPSY BEHAV	597	15	39	80	43	34	38	53	25	28	29	
2	2.839	SEIZURE-EUR J EPILEP	529	59	55	51	41	52	42	24	19	26	18	
3	5.067	EPILEPSIA	243	4	32	35	20	18	21	27	8	14	5	
4	2.491	EPILEPSY RES	167	7	8	25	17	8	12	6	7	8	11	
5	2.757	CURR PHARM DESIGN	110	14	11	6	12	9	9	4	9	5	12	
6	3.508	FRONT NEUROL	73	2	7	14	6	10	7	5	3	2	1	
7		J PEDIATR EPILEPSY	51	0	0	1	5	5	5	3	6	4	0	
8	1.500	EPILEPTIC DISORD	48	2	2	7	2	4	5	4	1	5	1	
9	2.766	PLOS ONE	48	2	0	6	6	2	5	4	1	6	3	
10	3.614	CLIN NEUROPHYSIOL	47	2	3	8	6	3	5	1	0	4	1	
11	3.126	ACTA NEUROL SCAND	45	0	0	1	5	6	5	5	4	4	1	
12	1.878	SEMIN PEDIATR NEUROL	44	1	1	7	3	4	1	3	3	3	1	

Rows 1 - 14 of 485 *(use csv export to download the full table)*

Downloaded on 2019/4/19 上午11:37:39 from Journal Citation Reports (©2019 Clarivate Analytics)

Citing Journal Data

Citing Half-Life Data

Customize columns

Citing Year	2017	2016	2015	2014	2013	2012	2011	2010	2009	2008	20
#Cites from 2017	184	664	748	680	704	639	554	472	439	405	
Cumulative %	1.94%	8.95%	16.85%	24.03%	31.46%	38.21%	44.06%	49.04%	53.67%	57.95%	10

Citing Journal Graph 2017

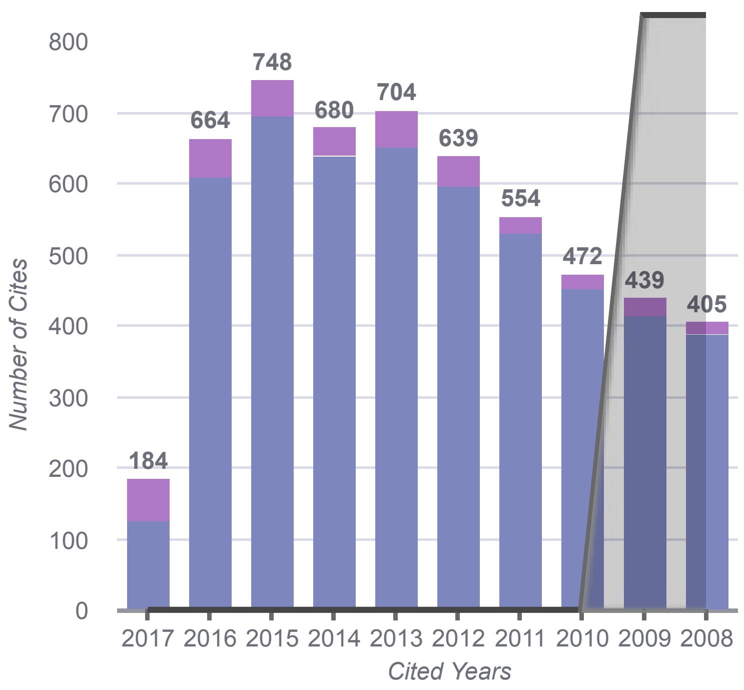

CITING JOURNAL GRAPH

The Citing Journal Graph shows the distribution (by cited year) of citations published in the Journal during the JCR year to items published in journals during the last 10 years.

The white/grey division indicates the citing half-life (if < 10.0). Half of the citations are to items that were published more recently than the citing half-life.

Downloaded on 2019/4/19 上午11:37:39 from Journal Citation Reports (©2019 Clarivate Analytics)

Citing Journal Data

Customize columns

	Impact	Cited Journal	All Yrs	2017	2016	2015	2014	2013	2012	2011	2010	2009	2008	R
		ALL Journals	9,472	184	664	748	680	704	639	554	472	439	405	3,
		ALL OTHERS (1079)	1,079	22	96	83	76	66	64	65	47	42	48	
1	5.067	EPILEPSIA	1,508	25	60	92	94	132	90	100	107	82	67	
2	2.600	EPILEPSY BEHAV	633	19	58	85	71	69	47	47	29	55	37	
3	8.055	NEUROLOGY	563	7	21	42	26	30	30	25	23	19	24	
4	2.839	SEIZURE-EUR J EPILEP	529	59	55	51	41	52	42	24	19	26	18	
5	2.491	EPILEPSY RES	319	1	25	28	34	23	21	20	29	9	18	
6	10.848	BRAIN	186	0	7	9	10	4	14	16	5	11	7	
7	10.250	ANN NEUROL	165	2	10	7	14	5	6	11	4	7	4	
8	7.144	J NEUROL NEUROSUR PS	126	0	11	3	8	7	4	4	4	5	2	
9	27.144	LANCET NEUROL	124	1	16	6	3	16	5	11	3	3	15	
10	53.254	LANCET	93	0	3	5	0	5	5	4	0	2	1	
11	3.126	ACTA NEUROL SCAND	88	1	11	3	7	7	7	4	3	3	2	
12	79.260	NEW ENGL J MED	85	0	1	0	0	0	3	6	7	2	2	
13	1.500	EPILEPTIC DISORD	73	0	9	6	9	5	10	7	1	1	1	

Rows 1 - 15 of 594 *(use csv export to download the full table)*

Downloaded on 2019/4/19 上午11:37:39 from Journal Citation Reports (©2019 Clarivate Analytics)

Clarivate Analytics

Metric trend

Metric Trend

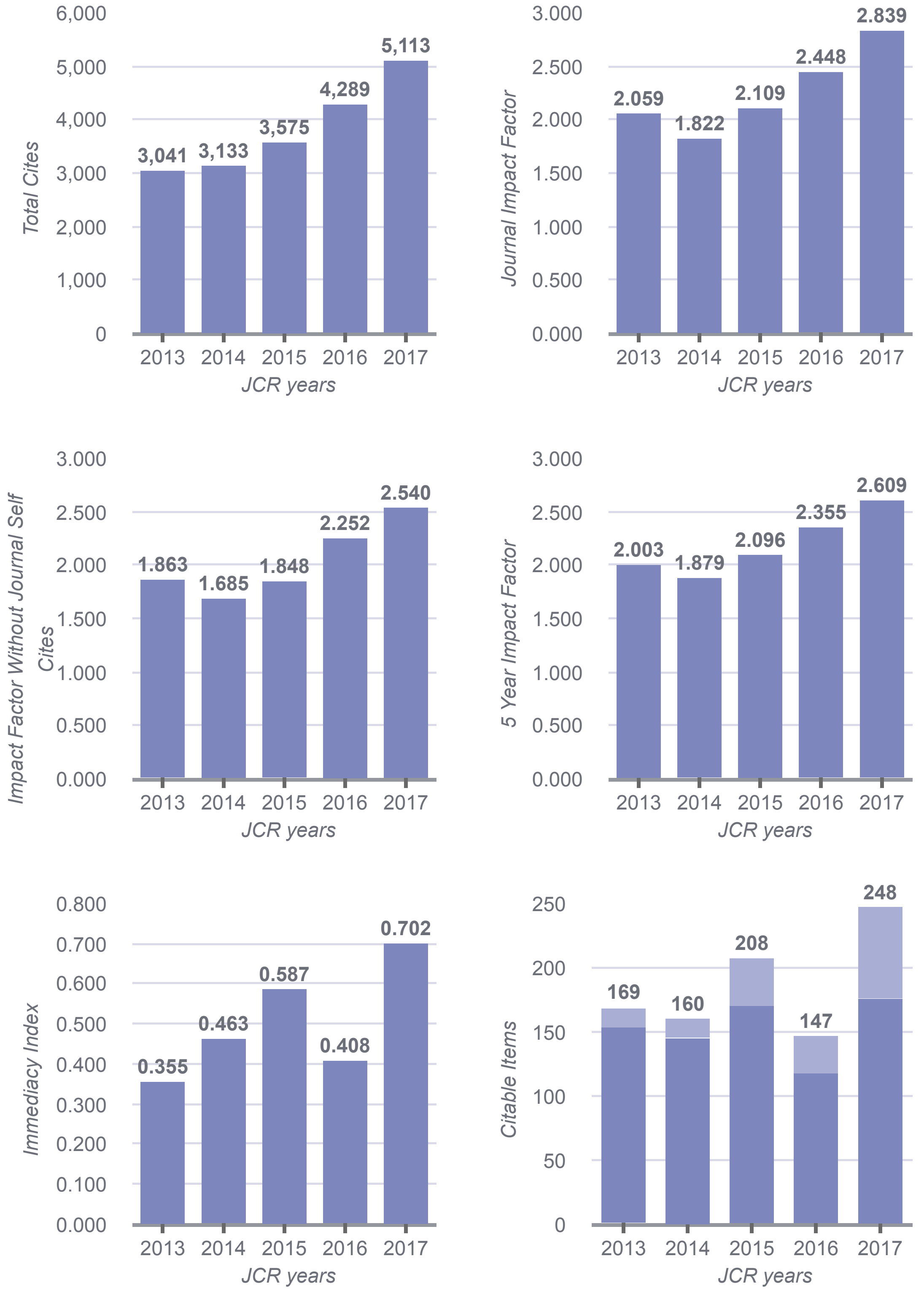

Downloaded on 2019/4/19 上午11:37:39 from Journal Citation Reports (©2019 Clarivate Analytics)

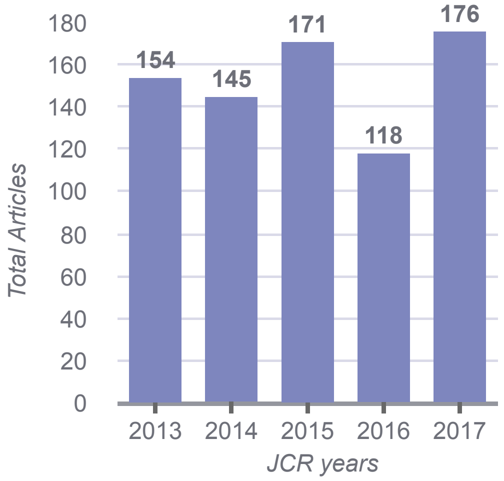

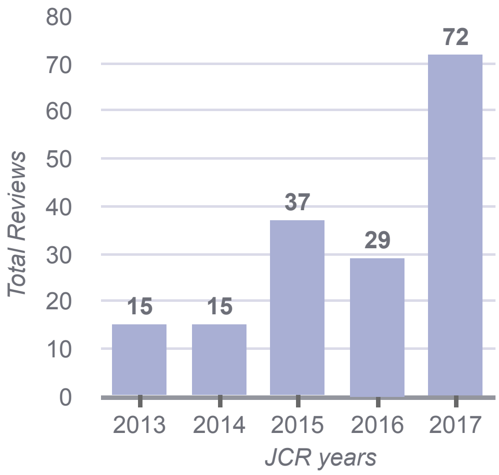

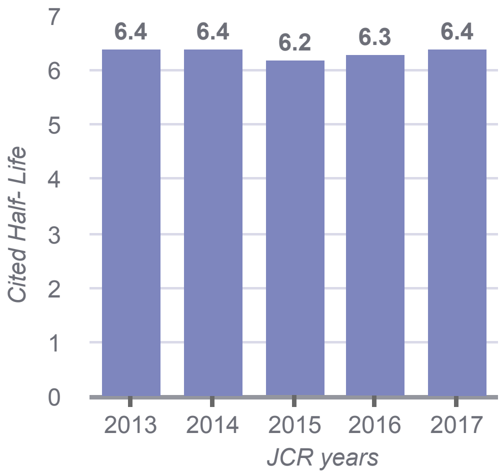

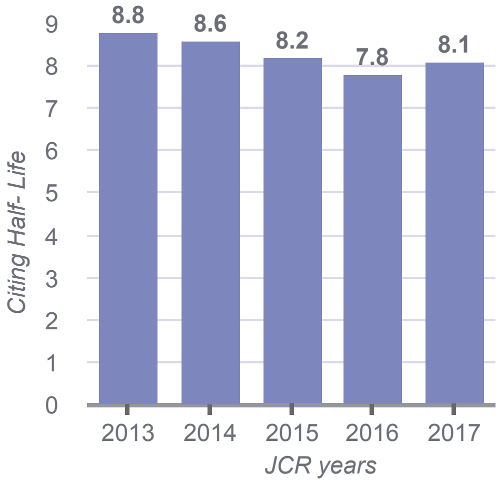

Downloaded on 2019/4/19 上午11:37:39 from Journal Citation Reports (©2019 Clarivate Analytics)

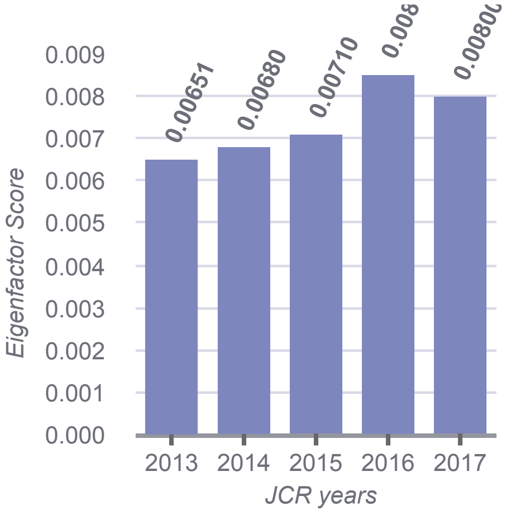

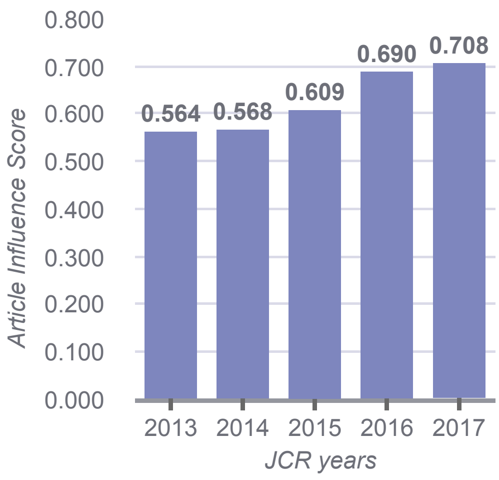

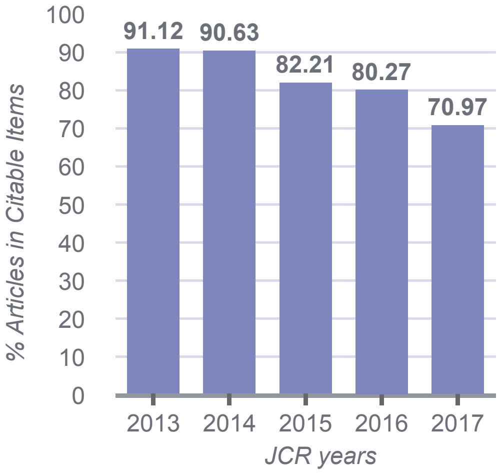

Downloaded on 2019/4/19 上午11:37:39 from Journal Citation Reports (©2019 Clarivate Analytics)

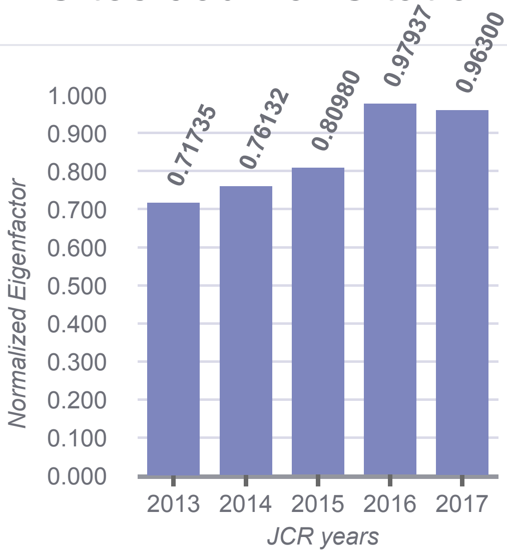

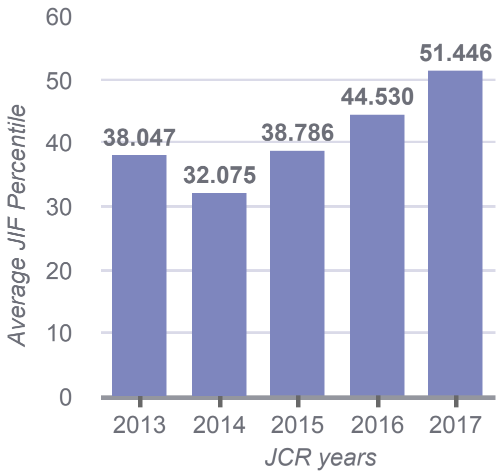

Downloaded on 2019/4/19 上午11:37:39 from Journal Citation Reports (©2019 Clarivate Analytics)

These data summarize the characteristics of the journal's published content for the most recent three years, that is, 2017 and the two prior years, combined. This information is based on all listed authors and addresses. It is meant to be descriptive rather than comparative.

Contributions by country/region

country	count
1. USA	117
2. England	89
3. GERMANY (FED REP GER)	72
4. CHINA MAINLAND	64
5. Italy	59
6. Canada	35
7. Turkey	32
8. Denmark	29
9. Norway	26
10. South Korea	25

Contributions by organizations

organization	count
1. UNIVERSITY OF LONDON	35
2. HARVARD UNIVERSITY	25
3. UNIVERSITY OF BONN	23
4. UNIVERSITY OF SHEFFIELD	20
5. DANISH EPILEPSY CTR	17
- SICHUAN UNIVERSITY	17
7. VA BOSTON HEALTHCARE SYSTEM	16
8. AARHUS UNIVERSITY	14
9. UNIVERSITY OF OSLO	13
- UNIVERSITY OF GENOA	13

Downloaded on 2019/4/19 上午11:37:39 from Journal Citation Reports (©2019 Clarivate Analytics)
