# Supplementary material for: Emerging trends and knowledge structure of epilepsy during pregnancy research for 2000–2018: a bibliometric analysis
Source: PeerJ. 2019 Jun 7;7:e7115. doi: 10.7717/peerj.7115 (PMC6557303; doi:10.7717/peerj.7115)
Supplement: Supplemental Information 4 [file peerj-07-7115-s004.zip › 7/7. InCites Journal Citation Reports(PLoS One).pdf]

2017 Journal Performance Data for: PLoS One

ISSN: 1932-6203
eISSN: 1932-6203
PUBLIC LIBRARY SCIENCE
1160 BATTERY STREET, STE 100, SAN FRANCISCO, CA 94111
USA

TITLES
ISO: PLoS One
JCR Abbrev: PLOS ONE

LANGUAGES
English

CATEGORIES
MULTIDISCIPLINARY SCIENCES - SCIE

Open Access from 2006

Downloaded on 2019/4/19 上午11:40:26 from Journal Citation Reports (©2019 Clarivate Analytics)

Current Year

The data in the two graphs below and in the Journal Impact Factor calculation panels represent citation activity in 2017 to items published in the journal in the prior two years. They detail the components of the Journal Impact Factor. Use the "All Years" tab to access key metrics and additional data for the current year and all prior years for this journal.

2017 Journal Impact Factor & percentile rank in category for: PLoS One

2.766
2017 Journal Impact Factor

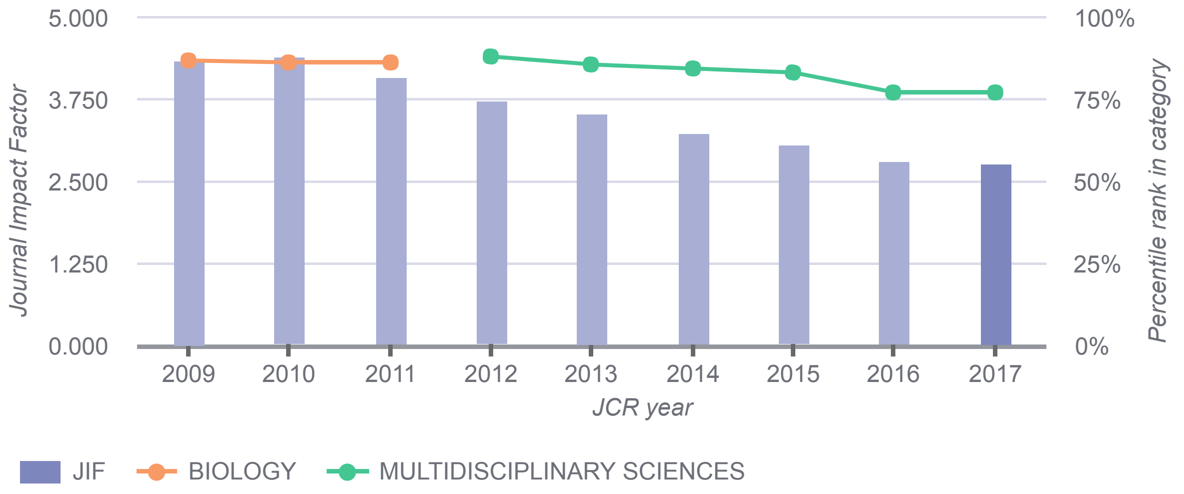

2017 JIF Citation Distribution for: PLoS One

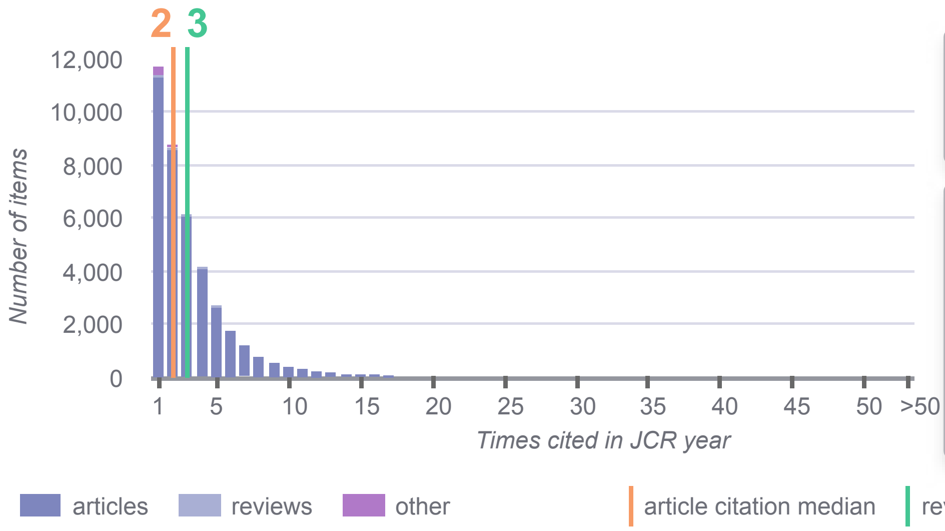

7,855
Unlinked citations

0
Times Cited

- **11,068** articles
- **71** reviews
- **2,289** other

Downloaded on 2019/4/19 上午11:40:26 from Journal Citation Reports (©2019 Clarivate Analytics)

Journal Impact Factor Calculation

$$2017\ \text{Journal Impact Factor} = \frac{138{,}835}{50{,}188} = 2.766$$

How is Journal Impact Factor Calculated?

$$\text{JIF} = \frac{\text{Citations in 2017 to items published in } \mathbf{2015\ (90{,}319)\ +\ 2016\ (48{,}516)}}{\text{Number of citable items in } \mathbf{2015\ (28{,}110)\ +\ 2016\ (22{,}078)}} = \frac{138{,}835}{50{,}188}$$

Downloaded on 2019/4/19 上午11:40:26 from Journal Citation Reports (©2019 Clarivate Analytics)

Journal Impact Factor contributing items

Citable items in 2016 and 2015 (50,188)

TITLE	CITATIONS COUNTED TOWARDS JIF
Global Estimates of the Prevalence and Incidence of Four Curable Sexually Transmitted Infections in 2012 Based on Systematic Review and Global Reporting<br>By: Newman, Lori; Temmerman, Marleen; Rowley, Jane; Vander Hoorn, Stephen; Wijesooriya, Nalinka Saman; et al.<br>**Volume: 10** **Accession number: WOS:000366903100002** **Document Type: Article**	122
A Compartmental Comparison of Major Lipid Species in a Coral-Symbiodinium Endosymbiosis: Evidence that the Coral Host Regulates Lipogenesis of Its Cytosolic Lipid Bodies<br>By: Chen, Hung-Kai; Song, Shin-Ni; Wang, Li-Hsueh; Mayfield, Anderson B.; Chen, Yi-Jyun; et al.<br>**Volume: 10** **Accession number: WOS:000358595900009** **Document Type: Article**	85
Dose-Response Analysis Using R<br>By: Ritz, Christian; Baty, Florent; Streibig, Jens C.; Gerhard, Daniel<br>**Volume: 10** **Accession number: WOS:000367510500116** **Document Type: Article**	74
The Global Burden of Mental, Neurological and Substance Use Disorders: An Analysis from the Global Burden of Disease Study 2010<br>By: Whiteford, Harvey A.; Ferrari, Alize J.; Degenhardt, Louisa; Feigin, Valery; Vos, Theo<br>**Volume: 10** **Accession number: WOS:000349444900070** **Document Type: Article**	72
Analytical and Clinical Validation of a Digital Sequencing Panel for Quantitative, Highly Accurate Evaluation of Cell-Free Circulating Tumor DNA<br>By: Lanman, Richard B.; Kopetz, E. Scott; Lee, Jeeyun; Nikolinakos, Petros G.; Baca, Arthur M.; et al.<br>**Volume: 10** **Accession number: WOS:000363185500089** **Document Type: Article**	69
Identification of Suitable Reference Genes for Gene Expression Normalization in the Quantitative Real-Time PCR Analysis of Sweet Osmanthus (Osmanthus fragrans Lour.)<br>By: Zhang, Chao; Fu, Jianxin; Wang, Yiguang; Bao, Zhiyi; Zhao, Hongbo<br>**Volume: 10** **Accession number: WOS:000359951900062** **Document Type: Article**	68
Global Prevalence of Chronic Kidney Disease - A Systematic Review and Meta-Analysis<br>By: Hill, Nathan R.; Fatoba, Samuel T.; Oke, Jason L.; Hirst, Jennifer A.; O'Callaghan, Christopher A.; et al.<br>**Volume: 11** **Accession number: WOS:000379809400083** **Document Type: Review**	66

Downloaded on 2019/4/19 上午11:40:26 from Journal Citation Reports (©2019 Clarivate Analytics)

Citations in 2017 (138,835)

TITLE	CITATIONS COUNTED TOWARDS JIF
PLOS ONE	8370
SCIENTIFIC REPORTS	5422
ONCOTARGET	2264
FRONTIERS IN MICROBIOLOGY	1038
INTERNATIONAL JOURNAL OF MOLECULAR SCIENCES	980
FRONTIERS IN PLANT SCIENCE	928
FRONTIERS IN IMMUNOLOGY	694
BMJ OPEN	506
PEERJ	483
PROCEEDINGS OF THE NATIONAL ACADEMY OF SCIENCES OF THE UNITED STATES OF AMERICA	474

Downloaded on 2019/4/19 上午11:40:26 from Journal Citation Reports (©2019 Clarivate Analytics)

Key Indicators 2017

IMPACT METRICS		INFLUENCE METRICS		SOURCE METRICS	
Total Cites	582,878	Eigenfactor Score	1.86200	Citable Items	20,328
Journal Impact Factor	2.766	Article Influence Score	1.000	% Articles in Citable Items	97.99
5 Year Impact Factor	3.352	Normalized Eigenfactor	217.45100	Average JIF Percentile	77.344
Immediacy Index	0.405			Cited Half-Life	4.3
Impact Factor Without Journal Self Cites	2.599			Citing Half-Life	8.2

Source data

Journal source data 2017

	Articles	Reviews	Combined(C)	Other(O)	Percentage(C/(C+O))
Number in JCR Year 2017 (A)	19,920	408	20,328	752	96%
Number of References (B)	939,992	26,497	966,489	1,024	99%
Ratio (B/A)	47.2	64.9	47.5	1.4	

Downloaded on 2019/4/19 上午11:40:26 from Journal Citation Reports (©2019 Clarivate Analytics)

Box plot

Category Box Plot 2017

Category Box Plot

The category box plot depicts the distribution of Impact Factors for all journals in the category. The horizontal line that forms the top of the box is the 75th percentile (Q1). The horizontal line that forms the bottom is the 25th percentile (Q3). The horizontal line that intersects the box is the median Impact Factor for the category. Horizontal lines above and below the box, called whiskers, represent maximum and minimum values.
The top whisker is the smaller of the following two values:
the maximum Impact Factor (IF)
Q1 IF + 3.5(Q1 IF - Q3 IF)
The bottom whisker is the larger of the following two values:
the minimum Impact Factor (IF)
Q1 IF - 3.5(Q1 IF - Q3 IF)
Box Plots are provided for the current JCR year for each of the categories in which the journal is indexed.

PLOS ONE, IF: 2.766

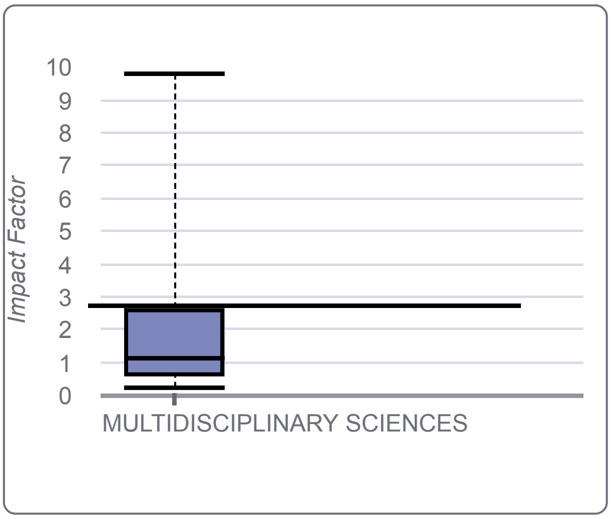

Downloaded on 2019/4/19 上午11:40:26 from Journal Citation Reports (©2019 Clarivate Analytics)

Rank

Rank 2017

JCR Impact Factor

JCR Year	MULTIDISCIPLINARY SCIENCES			BIOLOGY		
	Rank	Quartile	JIF Percentile	Rank	Quartile	JIF Percentile
2017	15/64	Q1	77.344	N/A	N/A	N/A
2016	15/64	Q1	77.344	N/A	N/A	N/A
2015	11/63	Q1	83.333	N/A	N/A	N/A
2014	9/57	Q1	85.088	N/A	N/A	N/A
2013	8/55	Q1	86.364	N/A	N/A	N/A
2012	7/56	Q1	88.393	N/A	N/A	N/A
2011	N/A	N/A	N/A	12/85	Q1	86.471
2010	N/A	N/A	N/A	12/86	Q1	86.628
2009	N/A	N/A	N/A	10/76	Q1	87.500

Downloaded on 2019/4/19 上午11:40:26 from Journal Citation Reports (©2019 Clarivate Analytics)

ESI Total Citations 2017

Rank

JCR Year	CLINICAL MEDICINE	MULTIDISCIPLINARY
2017	n/a	4/52-Q1
2016	n/a	4/48-Q1
2015	n/a	4/49-Q1
2014	1/1975-Q1	4/42-Q1
2013	2/1955-Q1	n/a

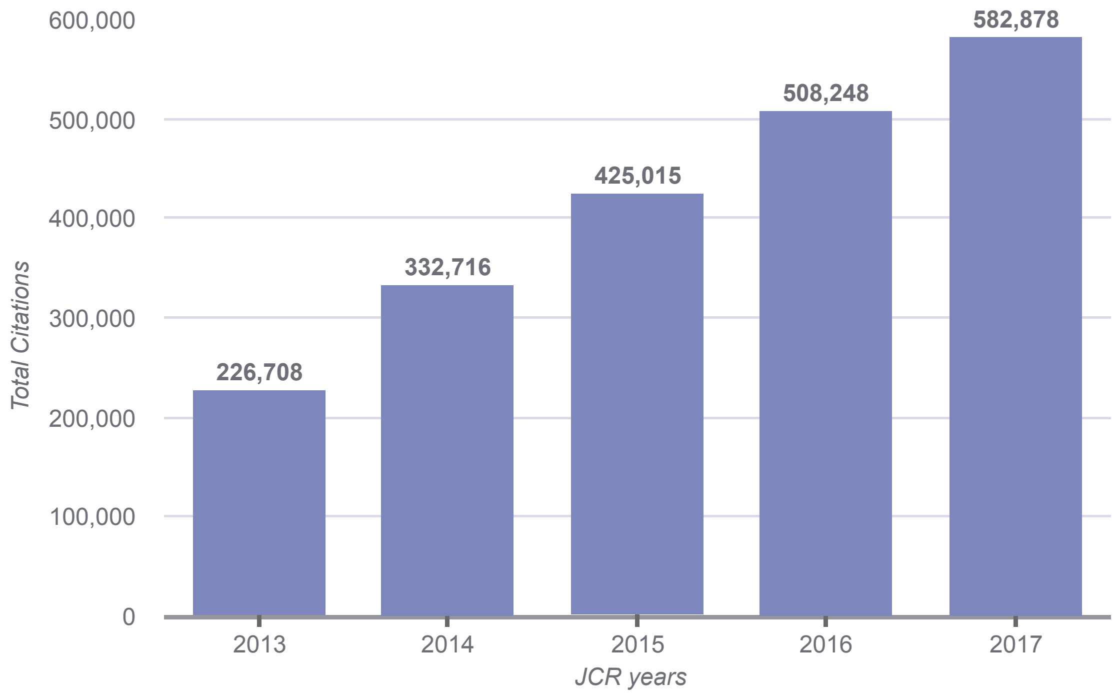

Downloaded on 2019/4/19 上午11:40:26 from Journal Citation Reports (©2019 Clarivate Analytics)

Cited Journal Data

Cited Half-Life Data

Customize columns

Cited Year	2017	2016	2015	2014	2013	2012	2011	2010	2009	2008	2
#Cites from 2017	8,223	48,516	90,319	107,550	116,895	89,692	55,563	29,008	17,723	11,917	
Cumulative %	1.41%	9.73%	25.23%	43.68%	63.74%	79.12%	88.66%	93.63%	96.67%	98.72%	1

Cited Journal Graph 2017

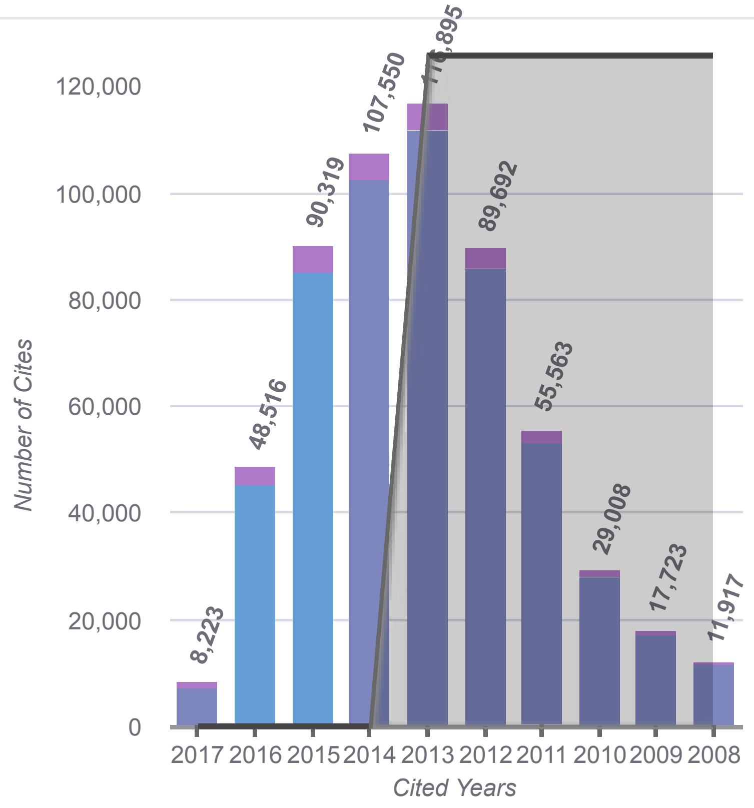

CITED JOURNAL GRAPH

The Cited Journal Graph shows the distribution (by cited year) of citations published in journals during the JCR year to items published in the Journal during the last 10 years.

The white/grey division indicates the cited half-life (if < 10.0). Half of the citations are to items that were published more recently than the cited half-life.

The two light-blue columns indicate citations used to calculate the Impact Factor (always the 2nd and 3rd columns).

Downloaded on 2019/4/19 上午11:40:26 from Journal Citation Reports (©2019 Clarivate Analytics)

Cited Journal Data

Customize columns

	Impact	Citing Journal	All Yrs	2017	2016	2015	2014	2013	2012	2011	201[illegible]
		ALL Journals	582,878	8,223	48,516	90,319	107,550	116,895	89,692	55,563	29,00[illegible]
		ALL OTHERS (2200)	2,200	43	232	415	444	455	251	170	6[illegible]
1	2.766	PLOS ONE	28,491	970	3,352	5,018	5,021	5,059	3,864	2,301	1,25[illegible]
2	4.122	SCI REP-UK	24,313	306	1,961	3,461	4,271	4,873	3,951	2,532	1,29[illegible]
3		ONCOTARGET	9,848	152	729	1,535	1,908	2,097	1,490	891	45[illegible]
4	4.019	FRONT MICROBIOL	4,511	119	422	616	778	889	698	448	26[illegible]
5	3.687	INT J MOL SCI	4,153	131	406	574	689	793	651	397	23[illegible]
6	3.677	FRONT PLANT SCI	3,279	59	343	585	590	684	486	276	9[illegible]
7	5.511	FRONT IMMUNOL	3,143	75	281	413	485	558	508	345	23[illegible]
8	12.353	NAT COMMUN	2,304	20	100	268	353	471	399	269	18[illegible]
9	9.504	P NATL ACAD SCI USA	2,211	23	179	295	287	410	374	273	14[illegible]
10	3.730	BMC GENOMICS	2,096	20	152	259	367	425	362	239	12[illegible]
11	2.118	PEERJ	2,088	35	180	303	358	416	312	207	11[illegible]
12	1.922	MOL MED REP	1,644	7	79	228	372	354	282	163	8[illegible]
13	2.413	BMJ OPEN	1,521	34	189	317	335	299	172	92	3[illegible]
14	2.583	BIOMED RES INT	1,468	30	151	261	277	307	199	126	6[illegible]

Rows 1 - 16 of 10,680 *(use csv export to download the full table)*

Downloaded on 2019/4/19 上午11:40:26 from Journal Citation Reports (©2019 Clarivate Analytics)

Citing Journal Data

Downloaded on 2019/4/19 上午11:40:26 from Journal Citation Reports (©2019 Clarivate Analytics)

Metric trend

Metric Trend

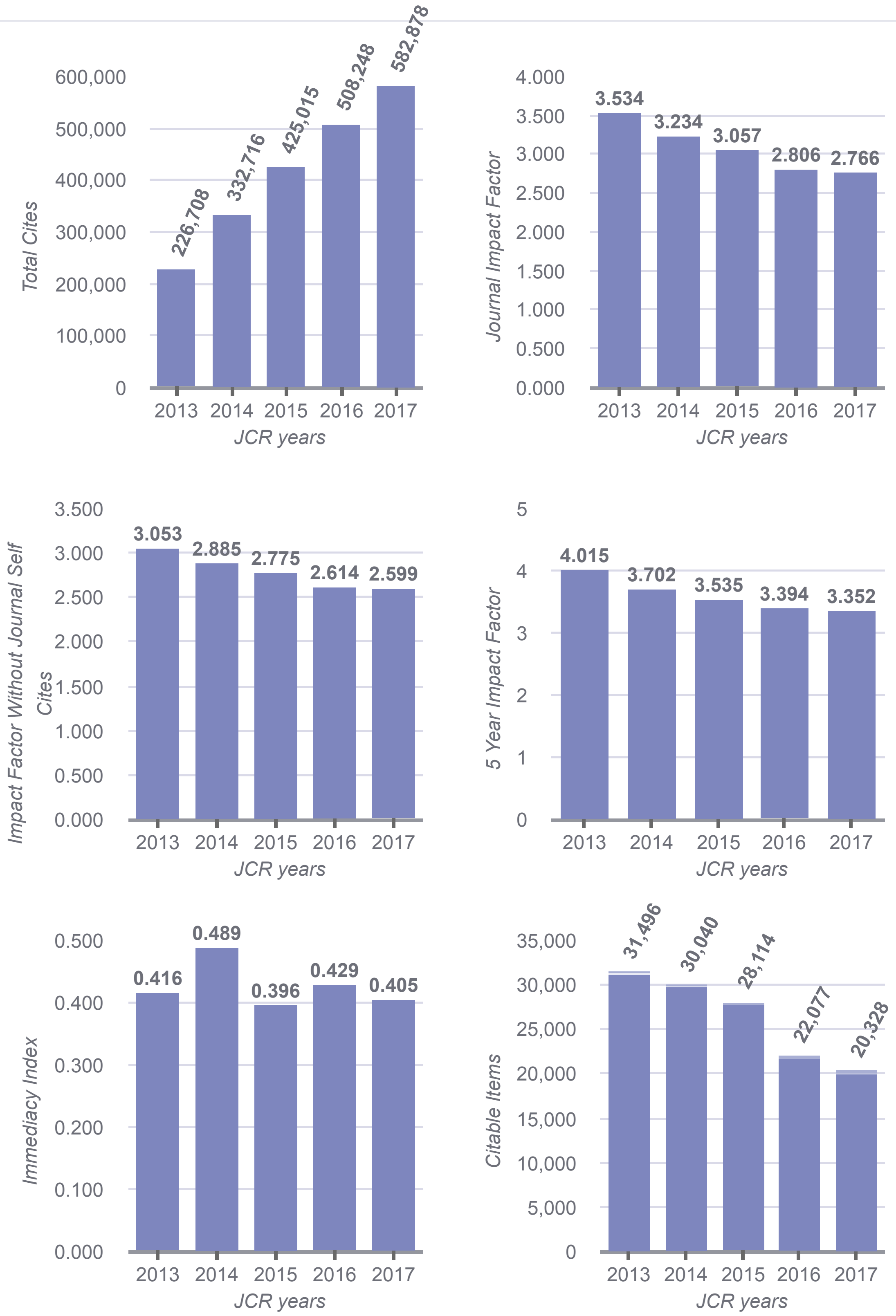

Downloaded on 2019/4/19 上午11:40:26 from Journal Citation Reports (©2019 Clarivate Analytics)

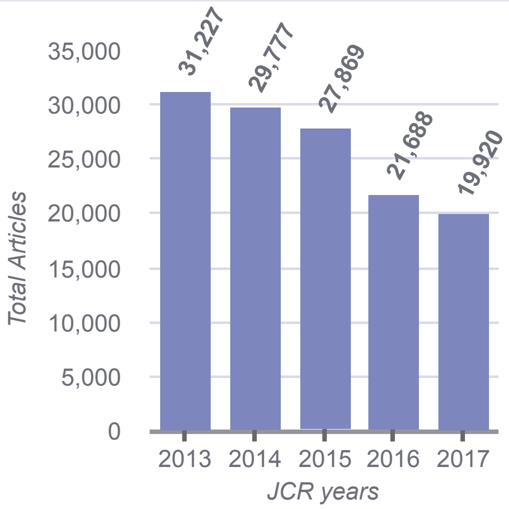

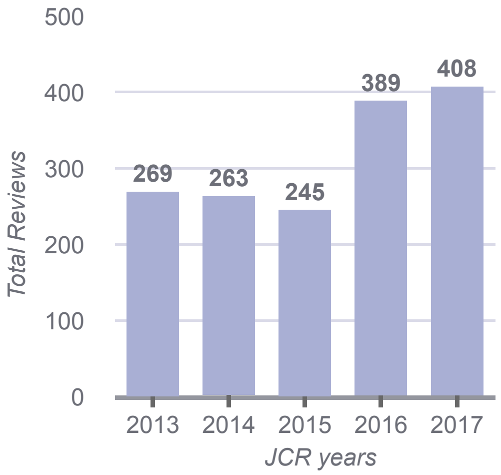

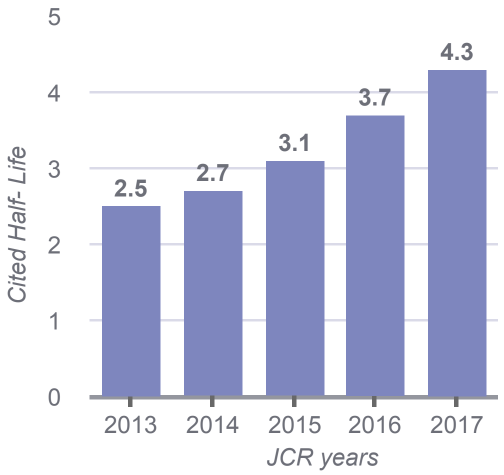

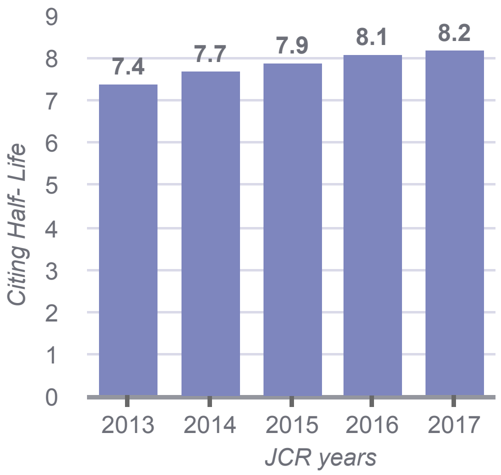

Downloaded on 2019/4/19 上午11:40:26 from Journal Citation Reports (©2019 Clarivate Analytics)

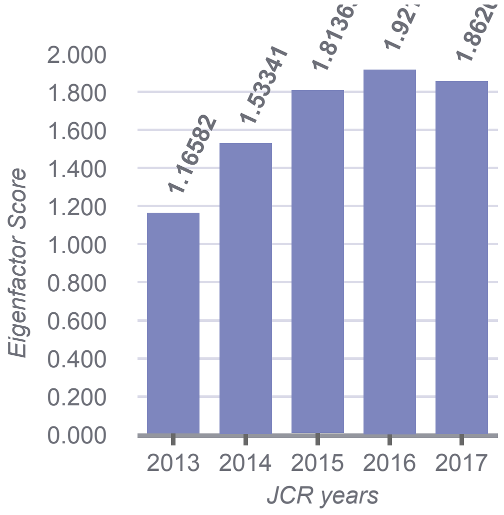

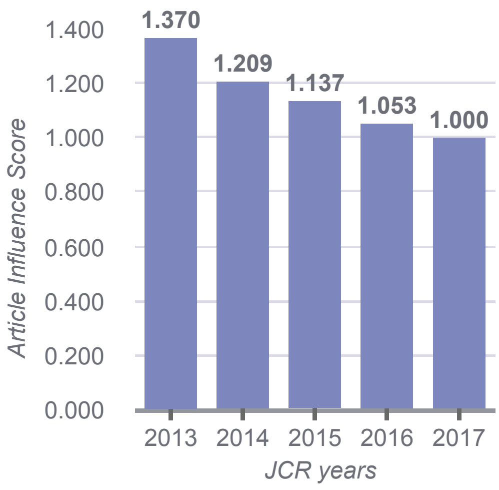

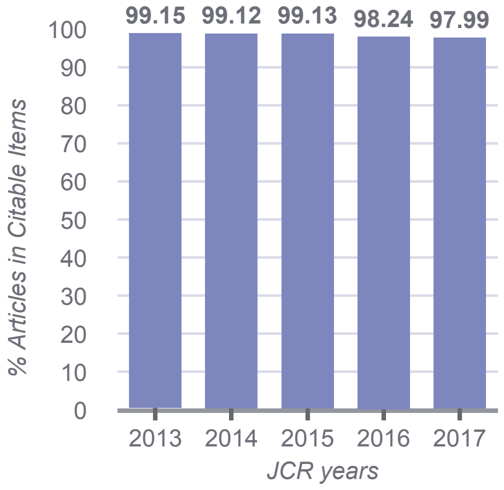

Downloaded on 2019/4/19 上午11:40:26 from Journal Citation Reports (©2019 Clarivate Analytics)

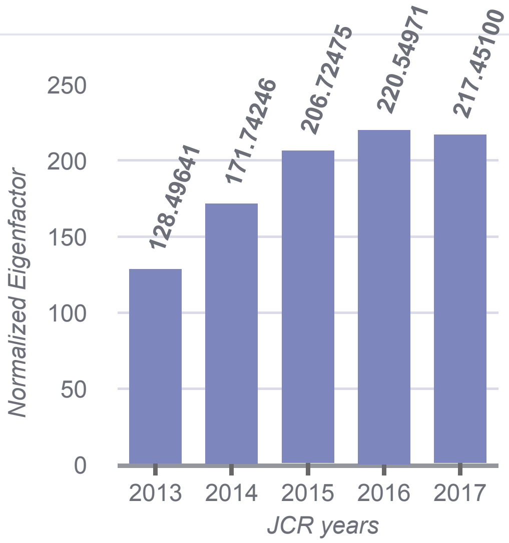

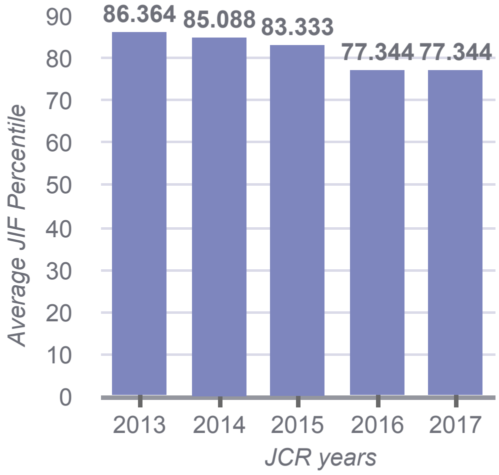

Downloaded on 2019/4/19 上午11:40:26 from Journal Citation Reports (©2019 Clarivate Analytics)

These data summarize the characteristics of the journal's published content for the most recent three years, that is, 2017 and the two prior years, combined. This information is based on all listed authors and addresses. It is meant to be descriptive rather than comparative.

Contributions by country/region

country	count
1. USA	20,935
2. CHINA MAINLAND	12,321
3. GERMANY (FED REP GER)	6,072
4. England	5,852
5. Japan	4,384
6. Canada	3,694
7. France	3,651
8. Australia	3,569
9. Netherlands	3,098
10. Spain	3,011

Contributions by organizations

organization	count
1. UNIVERSITY OF CALIFORNIA SYSTEM	2,326
2. UNIVERSITY OF LONDON	1,684
3. CENTRE NATIONAL DE LA RECHERCHE SCIENTIFIQUE (CNRS)	1,421
4. HARVARD UNIVERSITY	1,382
5. CHINESE ACADEMY OF SCIENCES	1,171
6. INSTITUT NATIONAL DE LA SANTE ET DE LA RECHERCHE MEDICALE (INSERM)	1,157
7. UNIVERSITY OF TEXAS SYSTEM	824
8. JOHNS HOPKINS UNIVERSITY	760
9. STATE UNIVERSITY SYSTEM OF FLORIDA	745
10. HELMHOLTZ ASSOCIATION	737

Downloaded on 2019/4/19 上午11:40:26 from Journal Citation Reports (©2019 Clarivate Analytics)
