# Supplementary material for: Emerging trends and knowledge structure of epilepsy during pregnancy research for 2000–2018: a bibliometric analysis
Source: PeerJ. 2019 Jun 7;7:e7115. doi: 10.7717/peerj.7115 (PMC6557303; doi:10.7717/peerj.7115)
Supplement: Supplemental Information 4 [file peerj-07-7115-s004.zip › 7/8. InCites Journal Citation Reports(EPILEPSY & BEHAVIOR).pdf]

2017 Journal Performance Data for: EPILEPSY & BEHAVIOR

ISSN: 1525-5050
eISSN: 1525-5069
ACADEMIC PRESS INC ELSEVIER SCIENCE
525 B ST, STE 1900, SAN DIEGO, CA 92101-4495
USA

TITLES
ISO: Epilepsy Behav.
JCR Abbrev: EPILEPSY BEHAV

LANGUAGES
English

CATEGORIES
BEHAVIORAL SCIENCES - SCIE

CLINICAL NEUROLOGY - SCIE

PSYCHIATRY - SCIE

PUBLICATION FREQUENCY
12 issues/year

Downloaded on 2019/4/19 上午11:42:06 from Journal Citation Reports (©2019 Clarivate Analytics)

Current Year

The data in the two graphs below and in the Journal Impact Factor calculation panels represent citation activity in 2017 to items published in the journal in the prior two years. They detail the components of the Journal Impact Factor. Use the "All Years" tab to access key metrics and additional data for the current year and all prior years for this journal.

2017 Journal Impact Factor & percentile rank in category for: EPILEPSY & BEHAVIOR

2.600
2017 Journal Impact Factor

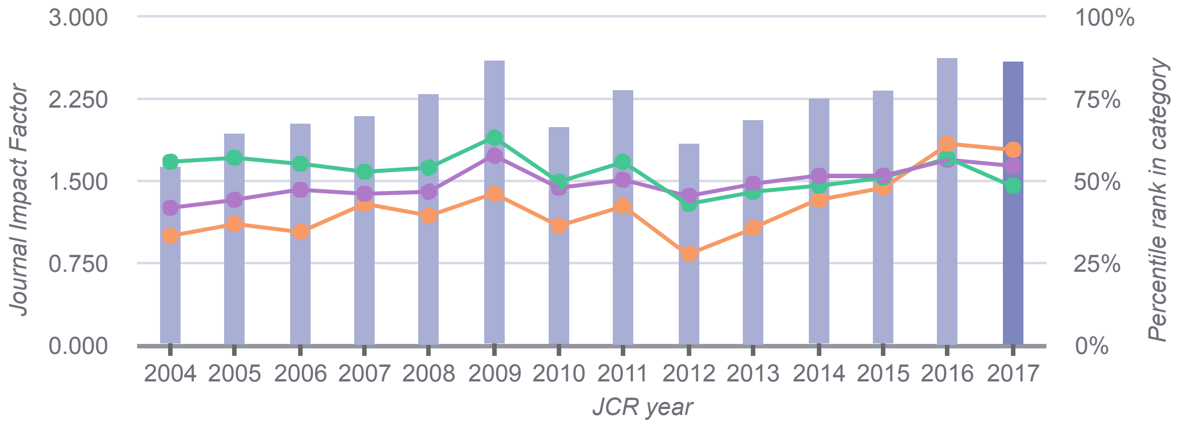

2017 JIF Citation Distribution for: EPILEPSY & BEHAVIOR

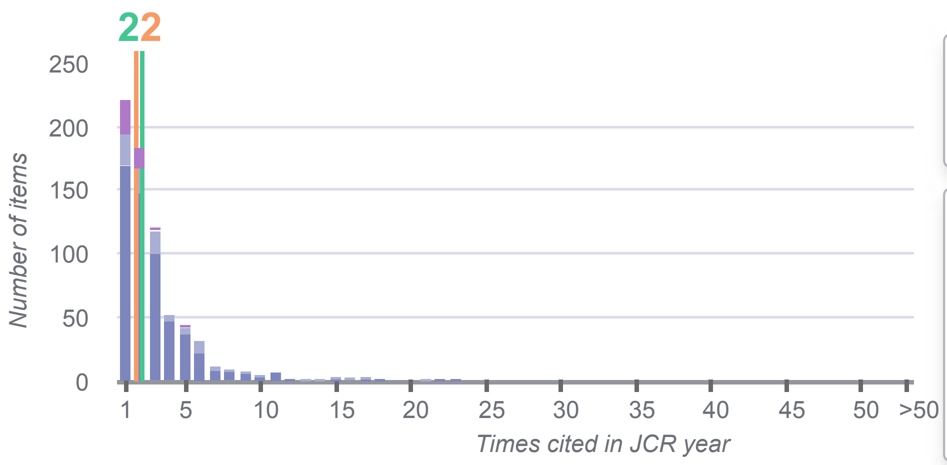

39
Unlinked citations

0
Times Cited

- **147** articles
- **18** reviews
- **77** other

articles | reviews | other | article citation median | review citation median

Downloaded on 2019/4/19 上午11:42:06 from Journal Citation Reports (©2019 Clarivate Analytics)

Journal Impact Factor Calculation

$$\text{2017 Journal Impact Factor} = \frac{2{,}135}{821} = 2.600$$

How is Journal Impact Factor Calculated?

$$\text{JIF} = \frac{\text{Citations in 2017 to items published in } \mathbf{2015\ (1{,}338) + 2016\ (797)}}{\text{Number of citable items in } \mathbf{2015\ (434) + 2016\ (387)}} = \frac{2{,}135}{821}$$

Downloaded on 2019/4/19 上午11:42:06 from Journal Citation Reports (©2019 Clarivate Analytics)

Journal Impact Factor contributing items

Citable items in 2016 and 2015 (821)

TITLE	CITATIONS COUNTED TOWARDS JIF
Parental reporting of response to oral cannabis extracts for treatment of refractory epilepsy<br>By: Press, Craig A.; Knupp, Kelly G.; Chapman, Kevin E.<br>**Volume: 45 Page: 49-52 Accession number: WOS:000353830700009**<br>**Document Type: Article**	23
Perceived efficacy of cannabidiol-enriched cannabis extracts for treatment of pediatric epilepsy: A potential role for infantile spasms and Lennox-Gastaut syndrome<br>By: Hussain, Shaun A.; Zhou, Raymond; Jacobson, Catherine; Weng, Julius; Cheng, Emily; et al.<br>**Volume: 47 Page: 138-141 Accession number: WOS:000356366900025**<br>**Document Type: Article**	22
Network analysis for a network disorder: The emerging role of graph theory in the study of epilepsy<br>By: Bernhardt, Boris C.; Bonilha, Leonardo; Gross, Donald W.<br>**Volume: 50 Page: 162-170 Accession number: WOS:000361186300029**<br>**Document Type: Review**	21
An open-label, prospective, exploratory study of patients with epilepsy switching from levetiracetam to brivaracetam<br>By: Yates, Stephen L.; Fakhoury, Toufic; Liang, Wei; Eckhardt, Klaus; Borghs, Simon; et al.<br>**Volume: 52 Page: 165-168 Accession number: WOS:000364990000029**<br>**Document Type: Article**	18
Clinical characteristics of children and young adults with co-occurring autism spectrum disorder and epilepsy<br>By: El Achkar, Christelle M.; Spence, Sarah J.<br>**Volume: 47 Page: 183-190 Accession number: WOS:000356366900032**<br>**Document Type: Review**	17
Laser ablation therapy: An alternative treatment for medically resistant mesial temporal lobe epilepsy after age 50<br>By: Waseem, Hena; Osborn, Katie E.; Schoenberg, Mike R.; Kelley, Valerie; Bozorg, Ali; et al.<br>**Volume: 51 Page: 152-157 Accession number: WOS:000362290200023**<br>**Document Type: Article**	17
Status epilepticus, blood-brain barrier disruption, inflammation, and epileptogenesis<br>By: Gorter, Jan A.; van Vliet, Erwin A.; Aronica, Eleonora<br>**Volume: 49 Page: 13-16 Accession number: WOS:000359314900004**<br>**Document Type: Review**	16

Downloaded on 2019/4/19 上午11:42:06 from Journal Citation Reports (©2019 Clarivate Analytics)

Citations in 2017 (2,135)

TITLE	CITATIONS COUNTED TOWARDS JIF
EPILEPSY & BEHAVIOR	440
SEIZURE-EUROPEAN JOURNAL OF EPILEPSY	143
EPILEPSIA	118
EPILEPSY RESEARCH	68
CURRENT PHARMACEUTICAL DESIGN	47
CURRENT OPINION IN NEUROLOGY	24
FRONTIERS IN NEUROLOGY	22
ACTA NEUROLOGICA SCANDINAVICA	21
SCIENTIFIC REPORTS	20
CLINICAL NEUROPHYSIOLOGY	18

Downloaded on 2019/4/19 上午11:42:06 from Journal Citation Reports (©2019 Clarivate Analytics)

Key Indicators 2017

IMPACT METRICS		INFLUENCE METRICS		SOURCE METRICS	
**Total Cites**	9,684	**Eigenfactor Score**	0.01600	**Citable Items**	397
**Journal Impact Factor**	2.600	**Article Influence Score**	0.663	**% Articles in Citable Items**	88.16
**5 Year Impact Factor**	2.655	**Normalized Eigenfactor**	1.90600	**Average JIF Percentile**	54.286
**Immediacy Index**	0.587			**Cited Half-Life**	5.8
**Impact Factor Without Journal Self Cites**	2.064			**Citing Half-Life**	8.9

Source data

Journal source data 2017

	Articles	Reviews	Combined(C)	Other(O)	Percentage(C/(C+O))
**Number in JCR Year 2017 (A)**	350	47	397	62	86%
**Number of References (B)**	12,634	3,204	15,838	633	96%
**Ratio (B/A)**	36.1	68.2	39.9	10.2	

Downloaded on 2019/4/19 上午11:42:06 from Journal Citation Reports (©2019 Clarivate Analytics)

Box plot

Category Box Plot 2017

Category Box Plot

The category box plot depicts the distribution of Impact Factors for all journals in the category. The horizontal line that forms the top of the box is the 75th percentile (Q1). The horizontal line that forms the bottom is the 25th percentile (Q3). The horizontal line that intersects the box is the median Impact Factor for the category. Horizontal lines above and below the box, called whiskers, represent maximum and minimum values.
The top whisker is the smaller of the following two values:
the maximum Impact Factor (IF)
Q1 IF + 3.5(Q1 IF - Q3 IF)
The bottom whisker is the larger of the following two values:
the minimum Impact Factor (IF)
Q1 IF - 3.5(Q1 IF - Q3 IF)
Box Plots are provided for the current JCR year for each of the categories in which the journal is indexed.

EPILEPSY BEHAV, IF: 2.600

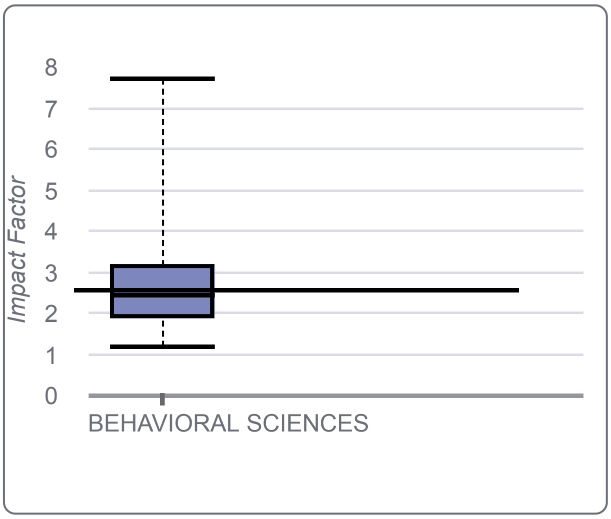

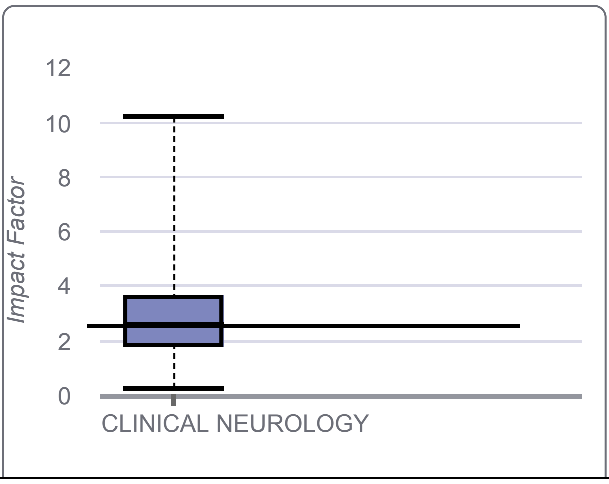

Downloaded on 2019/4/19 上午11:42:06 from Journal Citation Reports (©2019 Clarivate Analytics)

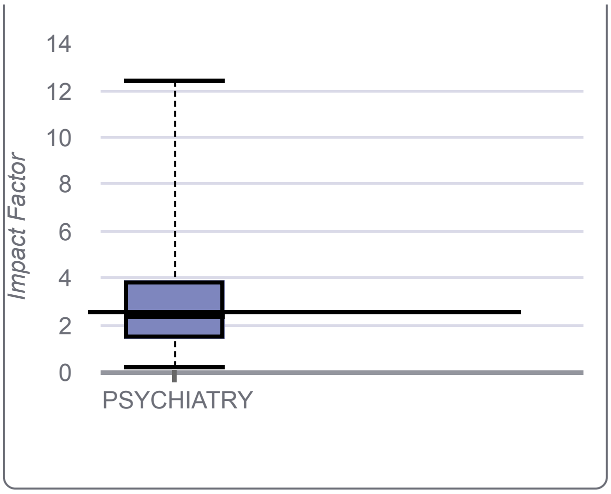

Downloaded on 2019/4/19 上午11:42:06 from Journal Citation Reports (©2019 Clarivate Analytics)

Rank

Rank 2017

JCR Impact Factor

JCR Year	BEHAVIORAL SCIENCES			CLINICAL NEUROLOGY			PSYCHIATRY		
	Rank	Quartile	JIF Percentile	Rank	Quartile	JIF Percentile	Rank	Quartile	JIF Percentil[illegible]
2017	21/51	Q2	59.804	102/197	Q3	48.477	65/142	Q2	54.57[illegible]
2016	20/51	Q2	61.765	84/194	Q2	56.959	62/142	Q2	56.69[illegible]
2015	27/51	Q3	48.039	95/193	Q2	51.036	69/142	Q2	51.76[illegible]
2014	29/51	Q3	44.118	99/192	Q3	48.698	68/140	Q2	51.78[illegible]
2013	32/49	Q3	35.714	104/194	Q3	46.649	70/136	Q3	48.89[illegible]
2012	36/49	Q3	27.551	110/193	Q3	43.264	74/135	Q3	45.55[illegible]
2011	28/48	Q3	42.708	85/192	Q2	55.990	65/130	Q2	50.38[illegible]
2010	31/48	Q3	36.458	93/185	Q3	50.000	67/128	Q3	48.04[illegible]
2009	27/49	Q3	45.918	62/167	Q2	63.174	50/117	Q2	57.69[illegible]
2008	29/47	Q3	39.362	72/156	Q2	54.167	54/101	Q3	47.03[illegible]
2007	26/45	Q3	43.333	69/146	Q2	53.082	51/94	Q3	46.27[illegible]
2006	28/42	Q3	34.524	66/147	Q2	55.442	50/94	Q3	47.34[illegible]
2005	27/42	Q3	36.905	64/148	Q2	57.095	53/94	Q3	44.14[illegible]
2004	28/41	Q3	32.927	62/140	Q2	56.071	53/90	Q3	41.66[illegible]

Downloaded on 2019/4/19 上午11:42:06 from Journal Citation Reports (©2019 Clarivate Analytics)

ESI Total Citations 2017

Rank

JCR Year	NEUROSCIENCE & BEHAVIOR
2017	77/346-Q1
2016	76/345-Q1
2015	82/344-Q1
2014	85/337-Q1
2013	95/339-Q2

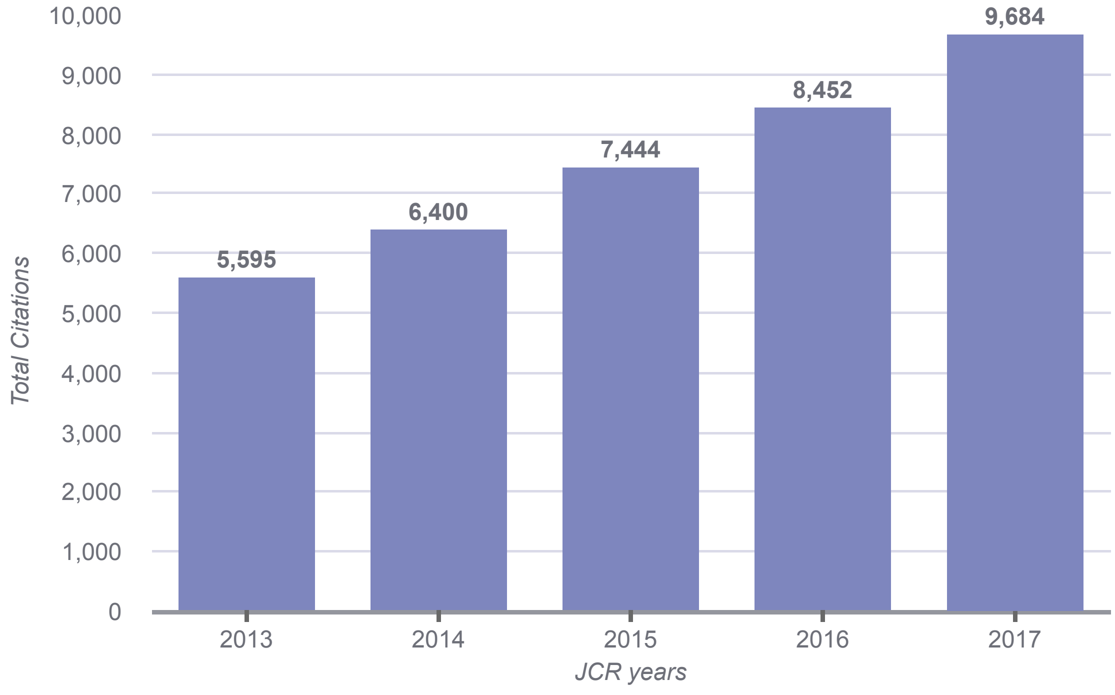

Downloaded on 2019/4/19 上午11:42:06 from Journal Citation Reports (©2019 Clarivate Analytics)

Cited Journal Data

Cited Half-Life Data

Customize columns

Cited Year	2017	2016	2015	2014	2013	2012	2011	2010	2009	2008	2
#Cites from 2017	233	797	1,338	1,007	846	759	824	617	678	539	
Cumulative %	2.41%	10.64%	24.45%	34.85%	43.59%	51.43%	59.93%	66.31%	73.31%	78.87%	1

Cited Journal Graph 2017

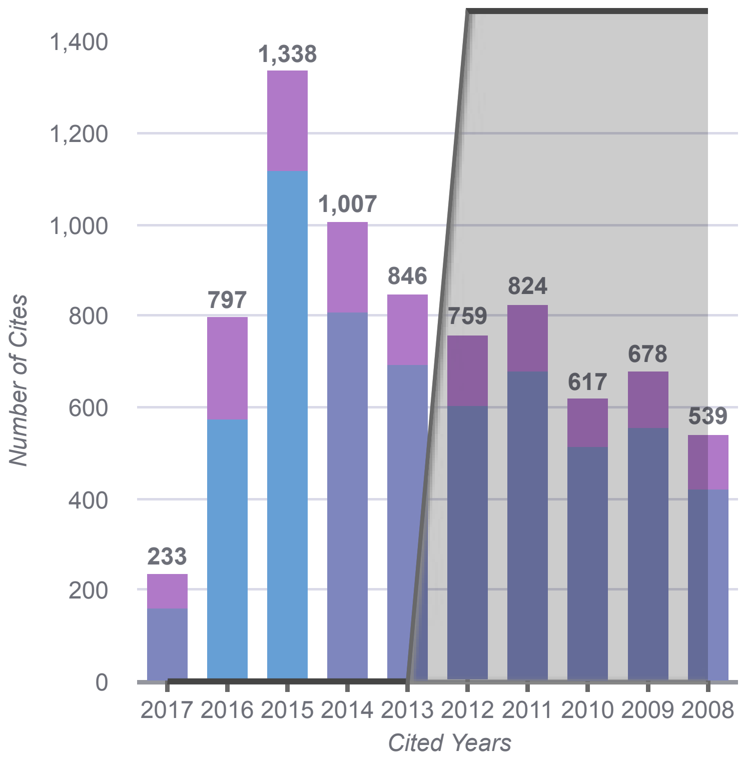

CITED JOURNAL GRAPH

The Cited Journal Graph shows the distribution (by cited year) of citations published in journals during the JCR year to items published in the Journal during the last 10 years.

The white/grey division indicates the cited half-life (if < 10.0). Half of the citations are to items that were published more recently than the cited half-life.

The two light-blue columns indicate citations used to calculate the Impact Factor (always the 2nd and 3rd columns).

Downloaded on 2019/4/19 上午11:42:06 from Journal Citation Reports (©2019 Clarivate Analytics)

Cited Journal Data

Customize columns

	Impact	Citing Journal	All Yrs	2017	2016	2015	2014	2013	2012	2011	2010	2009	2008
		ALL Journals	9,684	233	797	1,338	1,007	846	759	824	617	678	539
		ALL OTHERS (587)	587	12	48	81	67	48	40	54	41	33	39
1	2.600	EPILEPSY BEHAV	1,939	75	222	218	198	153	155	143	104	124	117
2	2.839	SEIZURE-EUR J EPILEP	633	19	58	85	71	69	47	47	29	55	37
3	5.067	EPILEPSIA	482	11	36	82	43	38	53	32	27	30	25
4	2.491	EPILEPSY RES	267	7	32	36	20	35	12	22	27	21	9
5	2.757	CURR PHARM DESIGN	179	5	18	29	13	18	12	10	13	14	5
6		J PEDIATR EPILEPSY	106	0	0	9	8	6	10	14	7	8	4
7	1.878	SEMIN PEDIATR NEUROL	100	7	11	7	8	7	8	11	6	8	7
8	2.766	PLOS ONE	95	0	2	8	12	20	5	6	2	11	7
9	3.126	ACTA NEUROL SCAND	93	1	2	19	16	8	5	7	4	16	2
10	3.508	FRONT NEUROL	88	3	7	15	8	9	9	8	5	4	4
11	4.122	SCI REP-UK	78	3	8	12	12	2	5	6	5	10	8
12	6.754	COCHRANE DB SYST REV	77	0	3	12	4	1	8	6	5	10	4

Rows 1 - 14 of 707 *(use csv export to download the full table)*

Downloaded on 2019/4/19 上午11:42:06 from Journal Citation Reports (©2019 Clarivate Analytics)

Citing Journal Data

Citing Half-Life Data

Customize columns

Citing Year	2017	2016	2015	2014	2013	2012	2011	2010	2009	2008	20
#Cites from 2017	241	1,086	1,222	1,110	1,043	1,023	997	796	785	703	
Cumulative %	1.46%	8.06%	15.48%	22.21%	28.55%	34.76%	40.81%	45.64%	50.41%	54.68%	10

Citing Journal Graph 2017

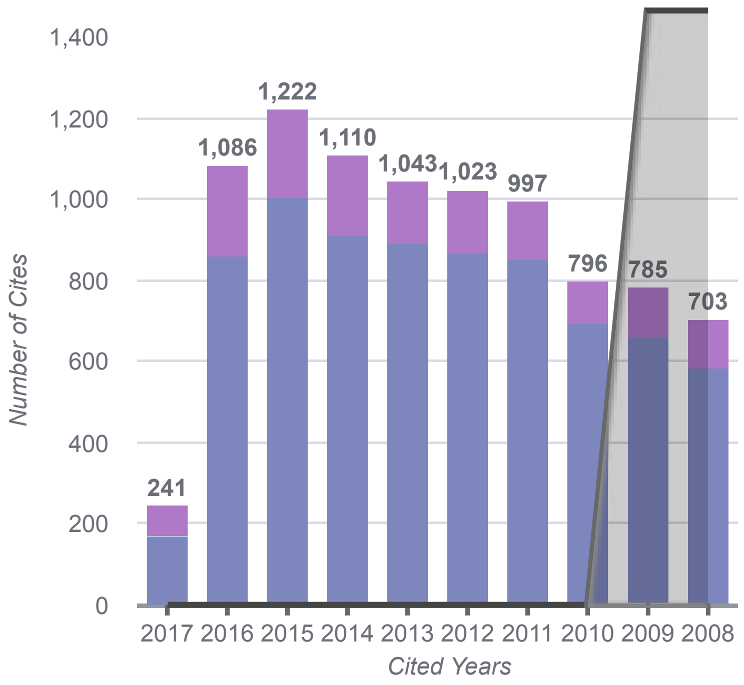

CITING JOURNAL GRAPH

The Citing Journal Graph shows the distribution (by cited year) of citations published in the Journal during the JCR year to items published in journals during the last 10 years.

The white/grey division indicates the citing half-life (if < 10.0). Half of the citations are to items that were published more recently than the citing half-life.

Downloaded on 2019/4/19 上午11:42:06 from Journal Citation Reports (©2019 Clarivate Analytics)

Citing Journal Data

Customize columns

	Impact	Cited Journal	All Yrs	2017	2016	2015	2014	2013	2012	2011	2010	2009	2008
		ALL Journals	16,471	241	1,086	1,222	1,110	1,043	1,023	997	796	785	703
		ALL OTHERS (1931)	1,931	33	122	140	134	124	124	105	77	96	61
1	5.067	EPILEPSIA	2,093	24	80	124	118	154	115	148	162	103	68
2	2.600	EPILEPSY BEHAV	1,939	75	222	218	198	153	155	143	104	124	117
3	8.055	NEUROLOGY	604	5	29	30	25	26	44	28	33	24	25
4	2.839	SEIZURE-EUR J EPILEP	597	15	39	80	43	34	38	53	25	28	29
5	2.491	EPILEPSY RES	513	1	33	38	45	45	22	30	21	27	22
6	10.848	BRAIN	190	1	9	12	10	4	8	14	9	13	14
7	27.144	LANCET NEUROL	164	1	29	7	9	15	13	7	2	8	20
8	10.250	ANN NEUROL	145	1	2	9	6	3	10	8	8	1	2
9	3.125	BRAIN RES	145	0	5	2	0	2	3	9	3	9	5
10	7.144	J NEUROL NEUROSUR PS	145	2	3	5	5	5	4	4	3	4	3
11	4.483	EXP NEUROL	113	0	3	3	5	3	5	2	3	3	3
12	5.971	J NEUROSCI	111	0	4	3	4	6	5	11	4	5	6
13	3.289	DEV MED CHILD NEUROL	98	0	2	7	4	4	4	7	4	2	4

Rows 1 - 15 of 1,076 *(use csv export to download the full table)*

Downloaded on 2019/4/19 上午11:42:06 from Journal Citation Reports (©2019 Clarivate Analytics)

Metric trend

Metric Trend

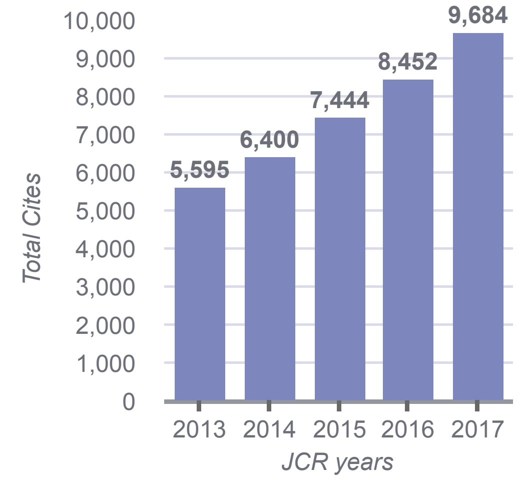

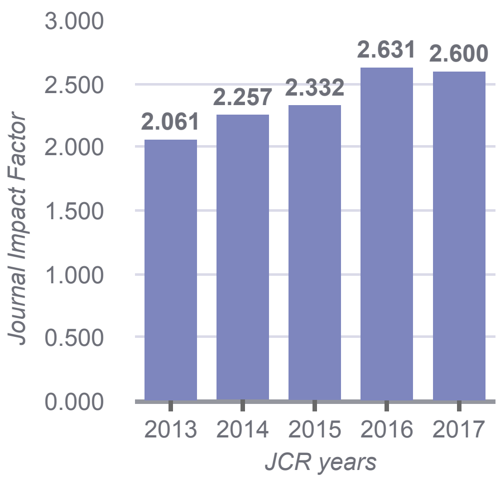

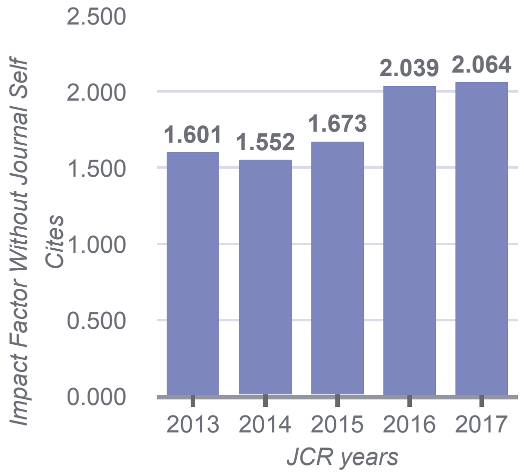

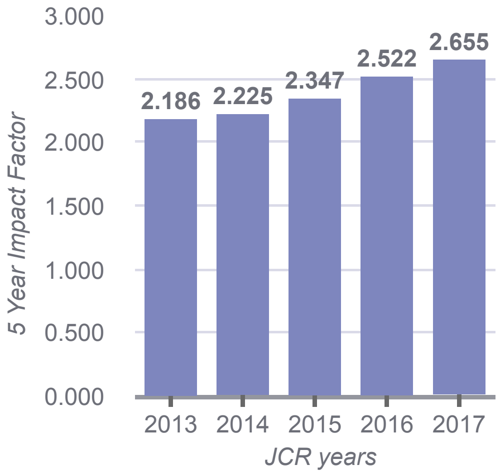

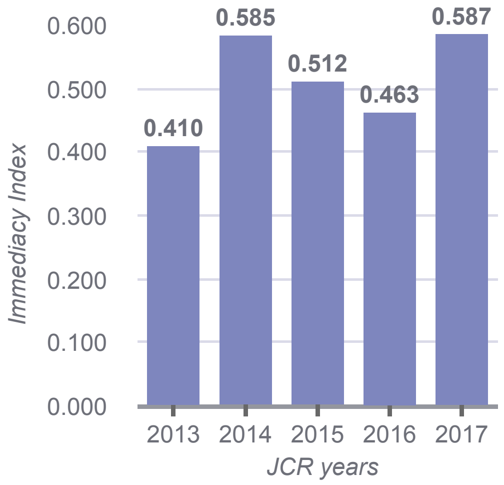

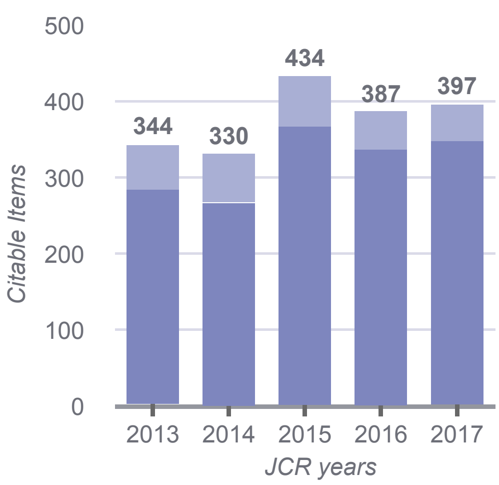

Downloaded on 2019/4/19 上午11:42:06 from Journal Citation Reports (©2019 Clarivate Analytics)

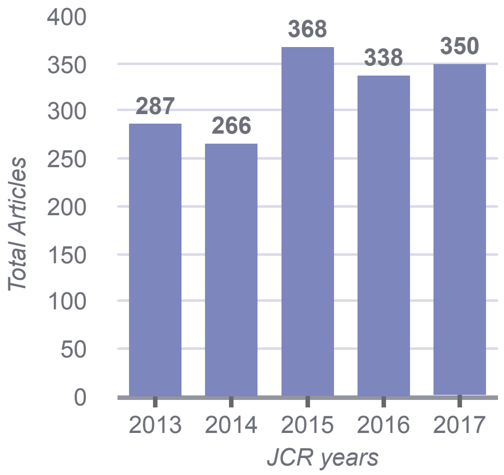

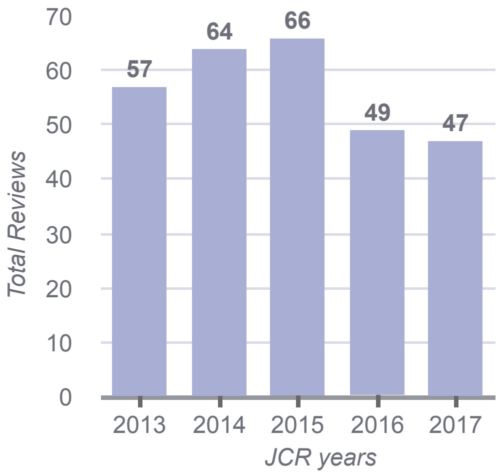

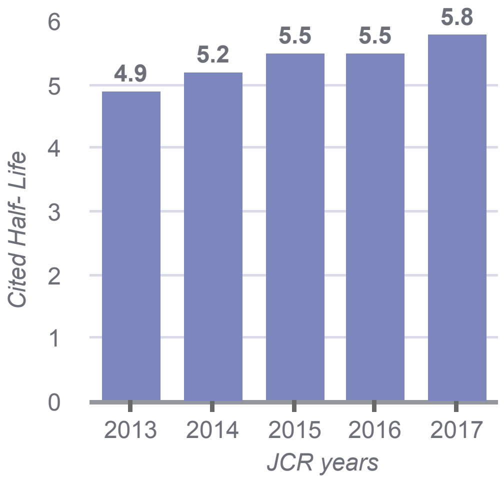

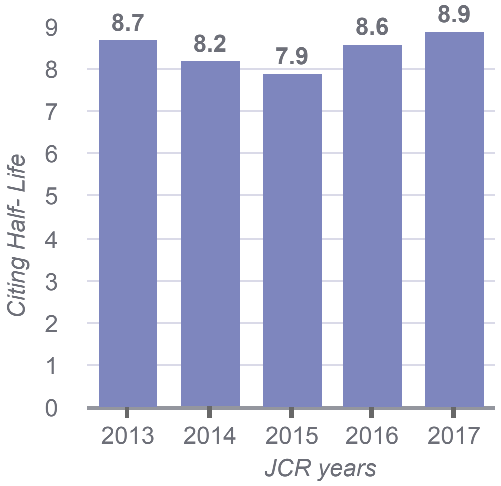

Downloaded on 2019/4/19 上午11:42:06 from Journal Citation Reports (©2019 Clarivate Analytics)

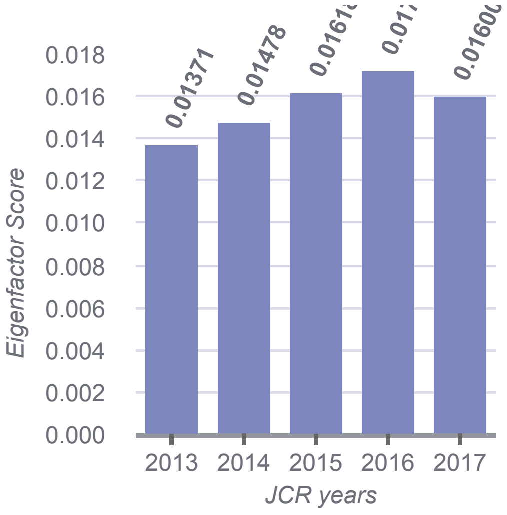

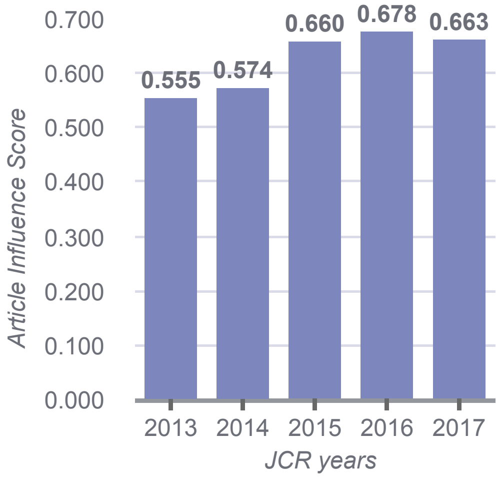

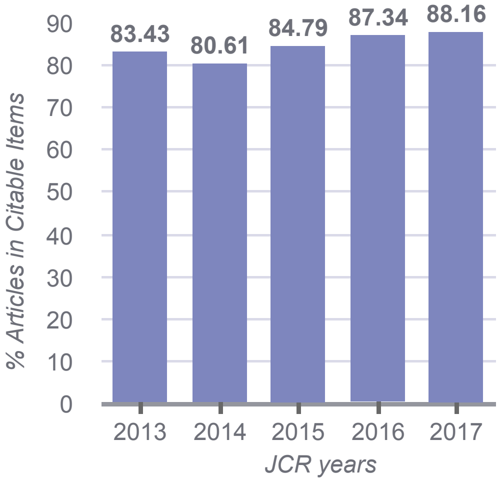

Downloaded on 2019/4/19 上午11:42:06 from Journal Citation Reports (©2019 Clarivate Analytics)

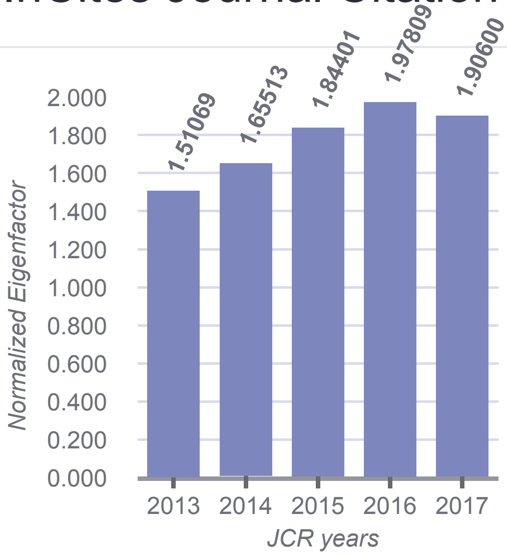

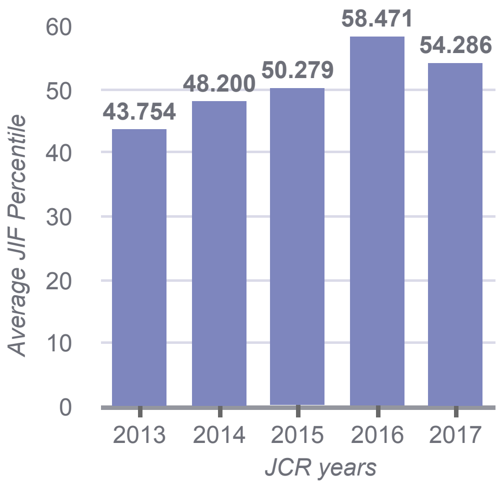

Downloaded on 2019/4/19 上午11:42:06 from Journal Citation Reports (©2019 Clarivate Analytics)

These data summarize the characteristics of the journal's published content for the most recent three years, that is, 2017 and the two prior years, combined. This information is based on all listed authors and addresses. It is meant to be descriptive rather than comparative.

Contributions by country/region

country	count
1. USA	518
2. England	156
3. GERMANY (FED REP GER)	97
4. Brazil	94
- Italy	94
6. Canada	92
7. CHINA MAINLAND	74
8. Netherlands	58
9. France	52
10. Australia	45

Contributions by organizations

organization	count
1. UNIVERSITY OF LONDON	63
2. HARVARD UNIVERSITY	52
3. UNIVERSITY OF CALIFORNIA SYSTEM	46
4. UNIVERSIDADE FEDERAL DE SAO PAULO (UNIFESP)	36
5. VA BOSTON HEALTHCARE SYSTEM	32
- NEW YORK UNIVERSITY	32
7. INSTITUT NATIONAL DE LA SANTE ET DE LA RECHERCHE MEDICALE (INSERM)	31
8. UNIVERSITY OF TORONTO	30
9. UNIVERSIDADE DE SAO PAULO	25
10. COLUMBIA UNIVERSITY	23
- EMORY UNIVERSITY	23

Downloaded on 2019/4/19 上午11:42:06 from Journal Citation Reports (©2019 Clarivate Analytics)
