# Supplementary material for: Emerging trends and knowledge structure of epilepsy during pregnancy research for 2000–2018: a bibliometric analysis
Source: PeerJ. 2019 Jun 7;7:e7115. doi: 10.7717/peerj.7115 (PMC6557303; doi:10.7717/peerj.7115)
Supplement: Supplemental Information 4 [file peerj-07-7115-s004.zip › 7/9. InCites Journal Citation Reports(REPRODUCTIVE TOXICOLOGY).pdf]

2017 Journal Performance Data for: REPRODUCTIVE TOXICOLOGY

ISSN: 0890-6238
PERGAMON-ELSEVIER SCIENCE LTD
THE BOULEVARD, LANGFORD LANE, KIDLINGTON, OXFORD OX5 1GB, ENGLAND
USA

TITLES
ISO: Reprod. Toxicol.
JCR Abbrev: REPROD TOXICOL

LANGUAGES
English

CATEGORIES
REPRODUCTIVE BIOLOGY - SCIE

TOXICOLOGY - SCIE

PUBLICATION FREQUENCY
8 issues/year

Downloaded on 2019/4/19 上午11:43:01 from Journal Citation Reports (©2019 Clarivate Analytics)

Current Year

The data in the two graphs below and in the Journal Impact Factor calculation panels represent citation activity in 2017 to items published in the journal in the prior two years. They detail the components of the Journal Impact Factor. Use the "All Years" tab to access key metrics and additional data for the current year and all prior years for this journal.

2017 Journal Impact Factor & percentile rank in category for: REPRODUCTIVE TOXICOLOGY

2.580
2017 Journal Impact Factor

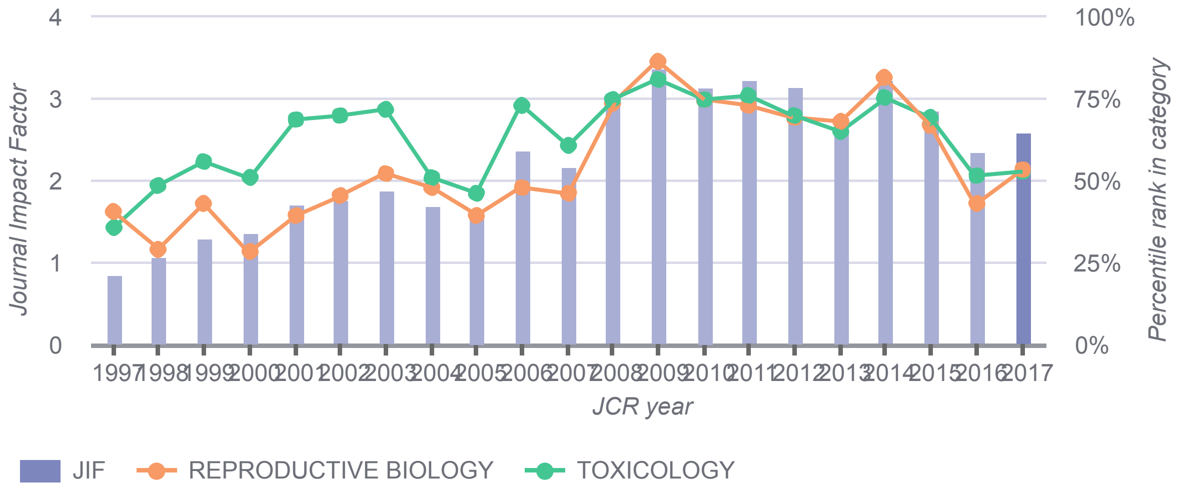

2017 JIF Citation Distribution for: REPRODUCTIVE TOXICOLOGY

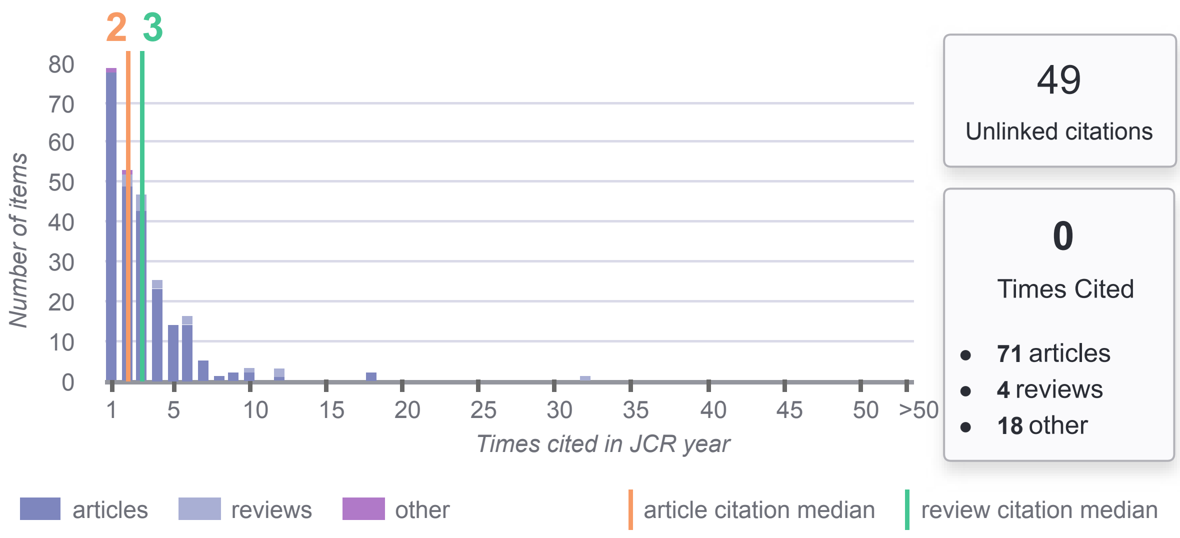

49
Unlinked citations

0
Times Cited

- **71** articles
- **4** reviews
- **18** other

Downloaded on 2019/4/19 上午11:43:01 from Journal Citation Reports (©2019 Clarivate Analytics)

Journal Impact Factor Calculation

$$\text{2017 Journal Impact Factor} = \frac{836}{324} = 2.580$$

How is Journal Impact Factor Calculated?

$$\text{JIF} = \frac{\text{Citations in 2017 to items published in } \textbf{2015 (412) + 2016 (424)}}{\text{Number of citable items in } \textbf{2015 (154) + 2016 (170)}} = \frac{836}{324}$$

Downloaded on 2019/4/19 上午11:43:01 from Journal Citation Reports (©2019 Clarivate Analytics)

Journal Impact Factor contributing items

Citable items in 2016 and 2015 (324)

TITLE	CITATIONS COUNTED TOWARDS JIF
A review of the carcinogenic potential of bisphenol A<br>By: Seachrist, Darcie D.; Bonk, Kristen W.; Ho, Shuk-Mei; Prins, Gail S.; Soto, Ana M.; et al.<br>**Volume: 59 Page: 167-182 Accession number: WOS:000372770300021**<br>**Document Type: Review**	32
A perspective on the developmental toxicity of inhaled nanoparticles<br>By: Hougaard, Karin Sorig; Piersma, Aldert H.; Ross, Bryony L.; Hutchison, Gary R.; Hansen, Jitka Stilund; et al.<br>**Volume: 56 Page: 118-140 Accession number: WOS:000359183400013**<br>**Document Type: Article**	18
Concentrations of environmental phenols and parabens in milk, urine and serum of lactating North Carolina women<br>By: Hines, Erin P.; Mendola, Pauline; von Ehrenstein, Ondine S.; Ye, Xiaoyun; Calafat, Antonia M.; et al.<br>**Volume: 54 Page: 120-128 Accession number: WOS:000356401300015**<br>**Document Type: Article**	18
Autism spectrum disorder and prenatal exposure to selective serotonin reuptake inhibitors: A systematic review and meta-analysis<br>By: Kobayashi, Tohru; Matsuyama, Tasuku; Takeuchi, Masanobu; Ito, Shinya<br>**Volume: 65 Page: 170-178 Accession number: WOS:000385990900020**<br>**Document Type: Review**	12
Prenatal factors associated with autism spectrum disorder (ASD)<br>By: Ornoy, A.; Weinstein-Fudim, L.; Ergaz, Z.<br>**Volume: 56 Page: 155-169 Accession number: WOS:000359183400016**<br>**Document Type: Review**	12
Estrogens in the wrong place at the wrong time: Fetal BPA exposure and mammary cancer<br>By: Paulose, Tessie; Speroni, Lucia; Sonnenschein, Carlos; Soto, Ana M.<br>**Volume: 54 Page: 58-65 Accession number: WOS:000356401300008**<br>**Document Type: Article**	12
The potential of AOP networks for reproductive and developmental toxicity assay development<br>By: Knapen, Dries; Vergauwen, Lucia; Villeneuve, Daniel L.; Ankley, Gerald T.<br>**Volume: 56 Page: 52-55 Accession number: WOS:000359183400005**<br>**Document Type: Article**	10

Downloaded on 2019/4/19 上午11:43:01 from Journal Citation Reports (©2019 Clarivate Analytics)

Citations in 2017 (836)

TITLE	CITATIONS COUNTED TOWARDS JIF
REPRODUCTIVE TOXICOLOGY	57
ENVIRONMENTAL HEALTH PERSPECTIVES	21
TOXICOLOGY AND APPLIED PHARMACOLOGY	20
ENVIRONMENT INTERNATIONAL	16
TOXICOLOGICAL SCIENCES	16
CHEMOSPHERE	15
INTERNATIONAL JOURNAL OF MOLECULAR SCIENCES	14
SCIENTIFIC REPORTS	14
ENVIRONMENTAL SCIENCE & TECHNOLOGY	12
TOXICOLOGY IN VITRO	12

Downloaded on 2019/4/19 上午11:43:01 from Journal Citation Reports (©2019 Clarivate Analytics)

Key Indicators 2017

IMPACT METRICS	
Total Cites	6,095
Journal Impact Factor	2.580
5 Year Impact Factor	3.132
Immediacy Index	0.869
Impact Factor Without Journal Self Cites	2.404

INFLUENCE METRICS	
Eigenfactor Score	0.00700
Article Influence Score	0.752
Normalized Eigenfactor	0.87200

SOURCE METRICS	
Citable Items	160
% Articles in Citable Items	89.38
Average JIF Percentile	53.054
Cited Half-Life	7.6
Citing Half-Life	8.1

Source data

Journal source data 2017

	Articles	Reviews	Combined(C)	Other(O)	Percentage(C/(C+O))
Number in JCR Year 2017 (A)	143	17	160	13	92%
Number of References (B)	8,661	1,600	10,261	113	98%
Ratio (B/A)	60.6	94.1	64.1	8.7	

Downloaded on 2019/4/19 上午11:43:01 from Journal Citation Reports (©2019 Clarivate Analytics)

Box plot

Category Box Plot 2017

Category Box Plot

The category box plot depicts the distribution of Impact Factors for all journals in the category. The horizontal line that forms the top of the box is the 75th percentile (Q1). The horizontal line that forms the bottom is the 25th percentile (Q3). The horizontal line that intersects the box is the median Impact Factor for the category. Horizontal lines above and below the box, called whiskers, represent maximum and minimum values.
The top whisker is the smaller of the following two values:
the maximum Impact Factor (IF)
Q1 IF + 3.5(Q1 IF - Q3 IF)
The bottom whisker is the larger of the following two values:
the minimum Impact Factor (IF)
Q1 IF - 3.5(Q1 IF - Q3 IF)
Box Plots are provided for the current JCR year for each of the categories in which the journal is indexed.

REPROD TOXICOL, IF: 2.580

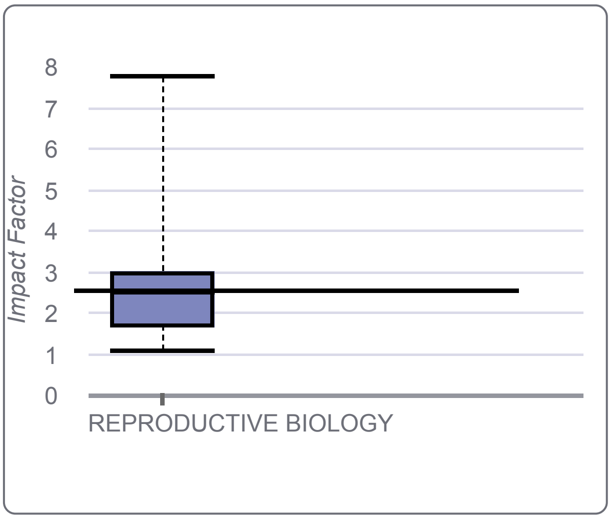

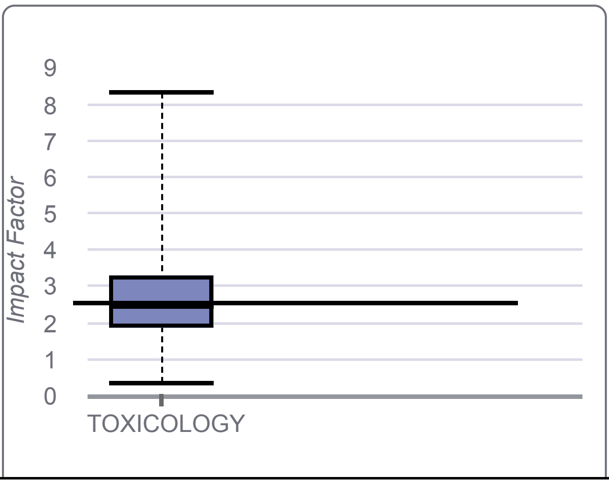

Downloaded on 2019/4/19 上午11:43:01 from Journal Citation Reports (©2019 Clarivate Analytics)

Rank

Rank 2017

JCR Impact Factor

JCR Year	REPRODUCTIVE BIOLOGY			TOXICOLOGY		
	Rank	Quartile	JIF Percentile	Rank	Quartile	JIF Percentile
2017	14/29	Q2	53.448	45/94	Q2	52.660
2016	17/29	Q3	43.103	45/92	Q2	51.630
2015	10/29	Q2	67.241	28/90	Q2	69.444
2014	6/30	Q1	81.667	22/88	Q1	75.568
2013	10/30	Q2	68.333	31/87	Q2	64.943
2012	9/28	Q2	69.643	26/85	Q2	70.000
2011	8/28	Q2	73.214	20/83	Q1	76.506
2010	7/26	Q2	75.000	21/83	Q2	75.301
2009	4/26	Q1	86.538	15/77	Q1	81.169
2008	7/25	Q2	74.000	19/75	Q2	75.333
2007	14/25	Q3	46.000	29/73	Q2	60.959
2006	13/24	Q3	47.917	21/76	Q2	73.026
2005	15/24	Q3	39.583	41/75	Q3	46.000
2004	13/24	Q3	47.917	37/75	Q2	51.333
2003	11/22	Q2	52.273	22/77	Q2	72.078
2002	13/23	Q3	45.652	23/76	Q2	70.395
2001	15/24	Q3	39.583	25/78	Q2	68.590
2000	17/23	Q3	28.261	38/77	Q2	51.299
1999	13/22	Q3	43.182	33/74	Q2	56.081
1998	14/19	Q3	28.947	37/71	Q3	48.592

Downloaded on 2019/4/19 上午11:43:01 from Journal Citation Reports (©2019 Clarivate Analytics)

Downloaded on 2019/4/19 上午11:43:01 from Journal Citation Reports (©2019 Clarivate Analytics)

ESI Total Citations 2017

Rank

JCR Year	PHARMACOLOGY & TOXICOLOGY
2017	70/274-Q2
2016	69/270-Q2
2015	64/266-Q1
2014	63/264-Q1
2013	70/267-Q2

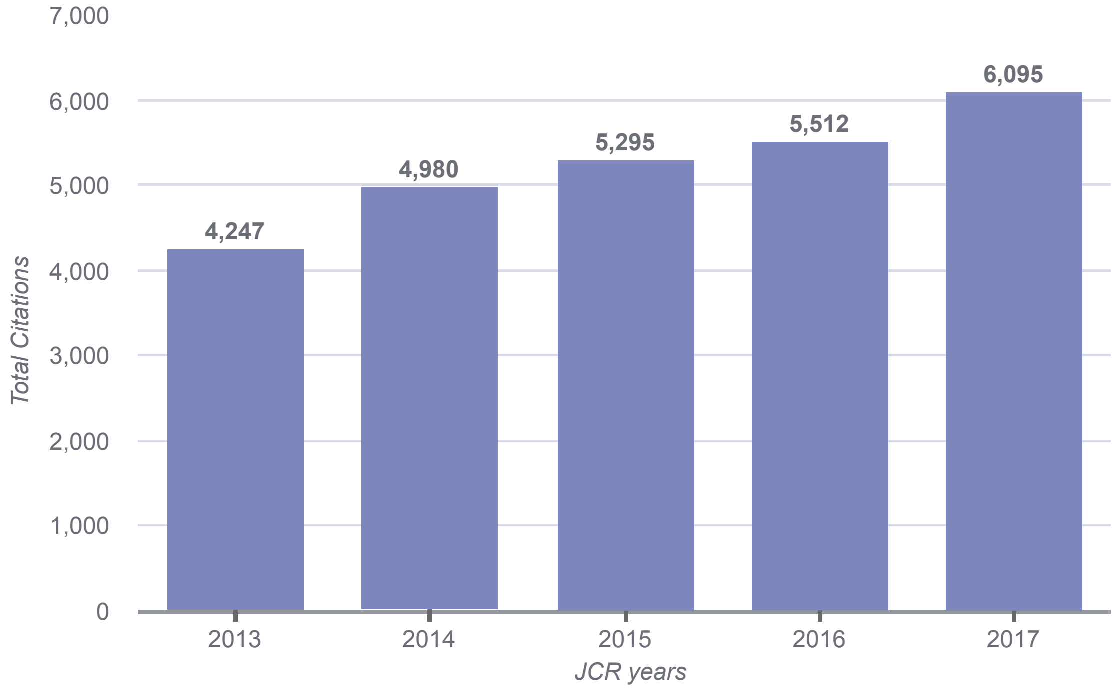

Downloaded on 2019/4/19 上午11:43:01 from Journal Citation Reports (©2019 Clarivate Analytics)

Cited Journal Data

Cited Half-Life Data

Customize columns

Cited Year	2017	2016	2015	2014	2013	2012	2011	2010	2009	2008	20
#Cites from 2017	139	424	412	351	506	565	434	365	350	308	
Cumulative %	2.28%	9.24%	16.00%	21.76%	30.06%	39.33%	46.45%	52.44%	58.18%	63.23%	10

Cited Journal Graph 2017

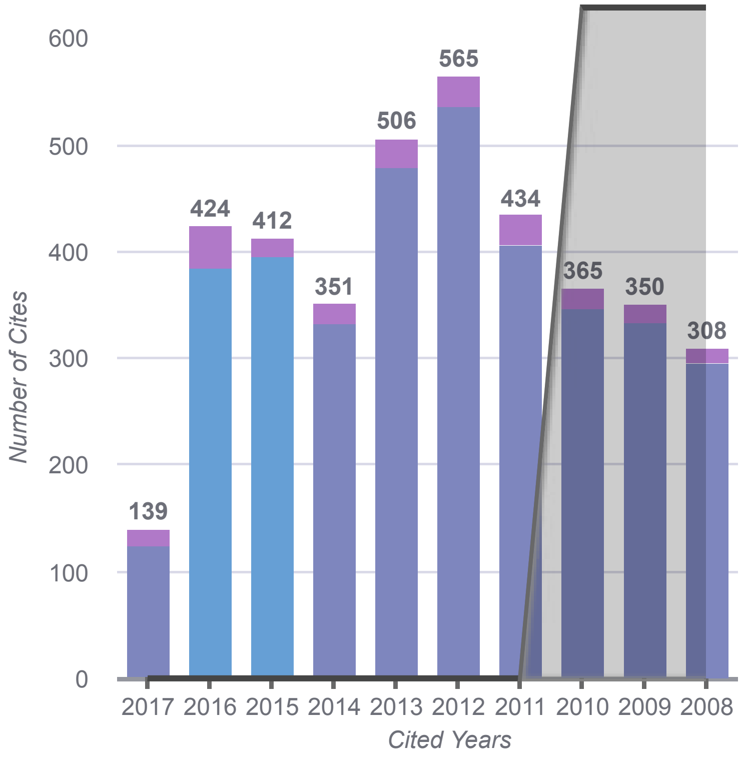

CITED JOURNAL GRAPH

The Cited Journal Graph shows the distribution (by cited year) of citations published in journals during the JCR year to items published in the Journal during the last 10 years.

The white/grey division indicates the cited half-life (if < 10.0). Half of the citations are to items that were published more recently than the cited half-life.

The two light-blue columns indicate citations used to calculate the Impact Factor (always the 2nd and 3rd columns).

Downloaded on 2019/4/19 上午11:43:01 from Journal Citation Reports (©2019 Clarivate Analytics)

Cited Journal Data

Customize columns

	Impact	Citing Journal	All Yrs	2017	2016	2015	2014	2013	2012	2011	2010	2009	2008	R
		ALL Journals	6,095	139	424	412	351	506	565	434	365	350	308	2,
		ALL OTHERS (682)	682	18	40	55	46	34	66	52	25	32	37	
1	2.580	REPROD TOXICOL	321	16	40	17	18	27	28	28	19	17	13	
2	4.122	SCI REP-UK	120	5	10	4	6	10	17	5	8	4	9	
3	4.427	CHEMOSPHERE	110	1	6	9	6	12	12	6	5	12	7	
4	3.616	TOXICOL APPL PHARM	96	1	11	9	3	8	17	5	6	6	5	
5	2.766	PLOS ONE	93	2	4	2	9	10	15	8	5	4	1	
6	4.610	SCI TOTAL ENVIRON	93	4	6	5	9	13	9	4	4	5	5	
7	4.181	TOXICOL SCI	90	3	10	6	4	11	20	2	3	7	3	
8	4.358	ENVIRON POLLUT	88	0	3	5	6	5	8	3	4	9	5	
9	2.800	ENVIRON SCI POLLUT R	88	3	6	5	1	6	10	2	10	3	8	
10	7.297	ENVIRON INT	84	3	9	7	3	7	16	4	5	12	4	
11	2.491	ENVIRON TOXICOL	84	0	1	5	3	7	6	8	9	5	2	
12	8.440	ENVIRON HEALTH PERSP	82	2	10	11	5	8	9	4	6	5	2	

Rows 1 - 14 of 648 *(use csv export to download the full table)*

Downloaded on 2019/4/19 上午11:43:01 from Journal Citation Reports (©2019 Clarivate Analytics)

Citing Journal Data

Citing Half-Life Data

Customize columns

Citing Year	2017	2016	2015	2014	2013	2012	2011	2010	2009	2008	20
#Cites from 2017	139	615	838	900	738	664	654	539	528	491	
Cumulative %	1.34%	7.27%	15.35%	24.02%	31.14%	37.54%	43.84%	49.04%	54.13%	58.86%	10

Citing Journal Graph 2017

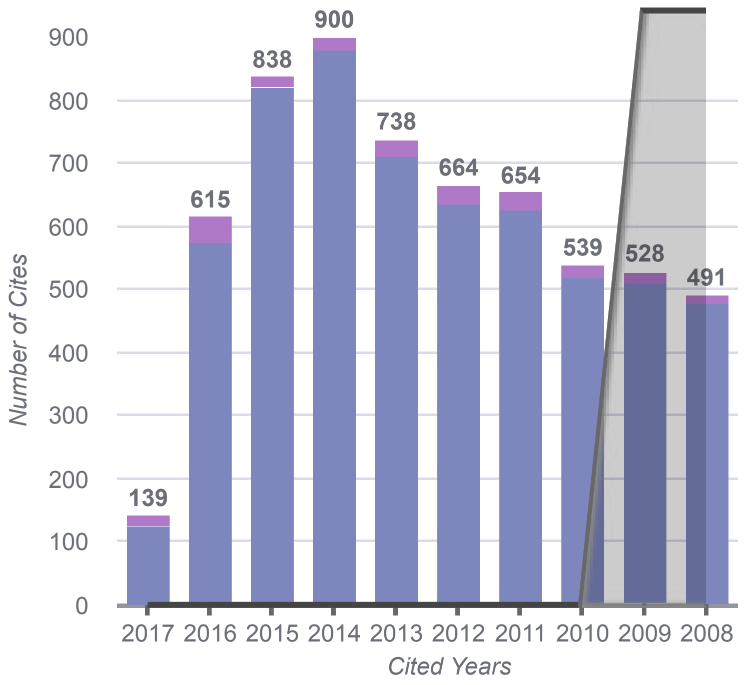

CITING JOURNAL GRAPH

The Citing Journal Graph shows the distribution (by cited year) of citations published in the Journal during the JCR year to items published in journals during the last 10 years.

The white/grey division indicates the citing half-life (if < 10.0). Half of the citations are to items that were published more recently than the citing half-life.

Downloaded on 2019/4/19 上午11:43:01 from Journal Citation Reports (©2019 Clarivate Analytics)

Citing Journal Data

Customize columns

	Impact	Cited Journal	All Yrs	2017	2016	2015	2014	2013	2012	2011	2010	2009	2008
		ALL Journals	10,374	139	615	838	900	738	664	654	539	528	491
		ALL OTHERS (1303)	1,303	24	103	116	135	94	72	88	61	47	54
1	8.440	ENVIRON HEALTH PERSP	422	4	25	33	33	29	31	37	32	27	27
2	2.580	REPROD TOXICOL	321	16	40	17	18	27	28	28	19	17	13
3	3.961	ENDOCRINOLOGY	200	3	10	24	11	6	13	11	5	5	8
4	4.181	TOXICOL SCI	187	0	7	7	20	11	11	15	15	13	8
5	2.766	PLOS ONE	179	3	11	21	47	36	28	17	5	6	3
6	3.616	TOXICOL APPL PHARM	169	0	7	15	17	7	15	6	7	14	9
7	3.184	BIOL REPROD	150	0	4	12	10	9	9	9	5	9	2
8	4.990	HUM REPROD	150	1	9	8	9	8	16	8	7	9	5
9	4.803	FERTIL STERIL	139	2	6	15	8	11	11	11	12	2	14
10	9.504	P NATL ACAD SCI USA	110	1	2	6	1	11	5	4	8	10	4
11	41.577	NATURE	93	0	2	4	1	4	2	4	4	1	4
12	4.732	ENVIRON RES	85	1	7	17	14	4	4	4	0	1	12
13	3.166	TOXICOL LETT	82	2	4	4	14	10	6	7	7	6	2
14	3.265	TOXICOLOGY	78	3	1	9	12	1	2	7	0	6	1

Rows 1 - 16 of 963 *(use csv export to download the full table)*

Downloaded on 2019/4/19 上午11:43:01 from Journal Citation Reports (©2019 Clarivate Analytics)

Metric trend

Metric Trend

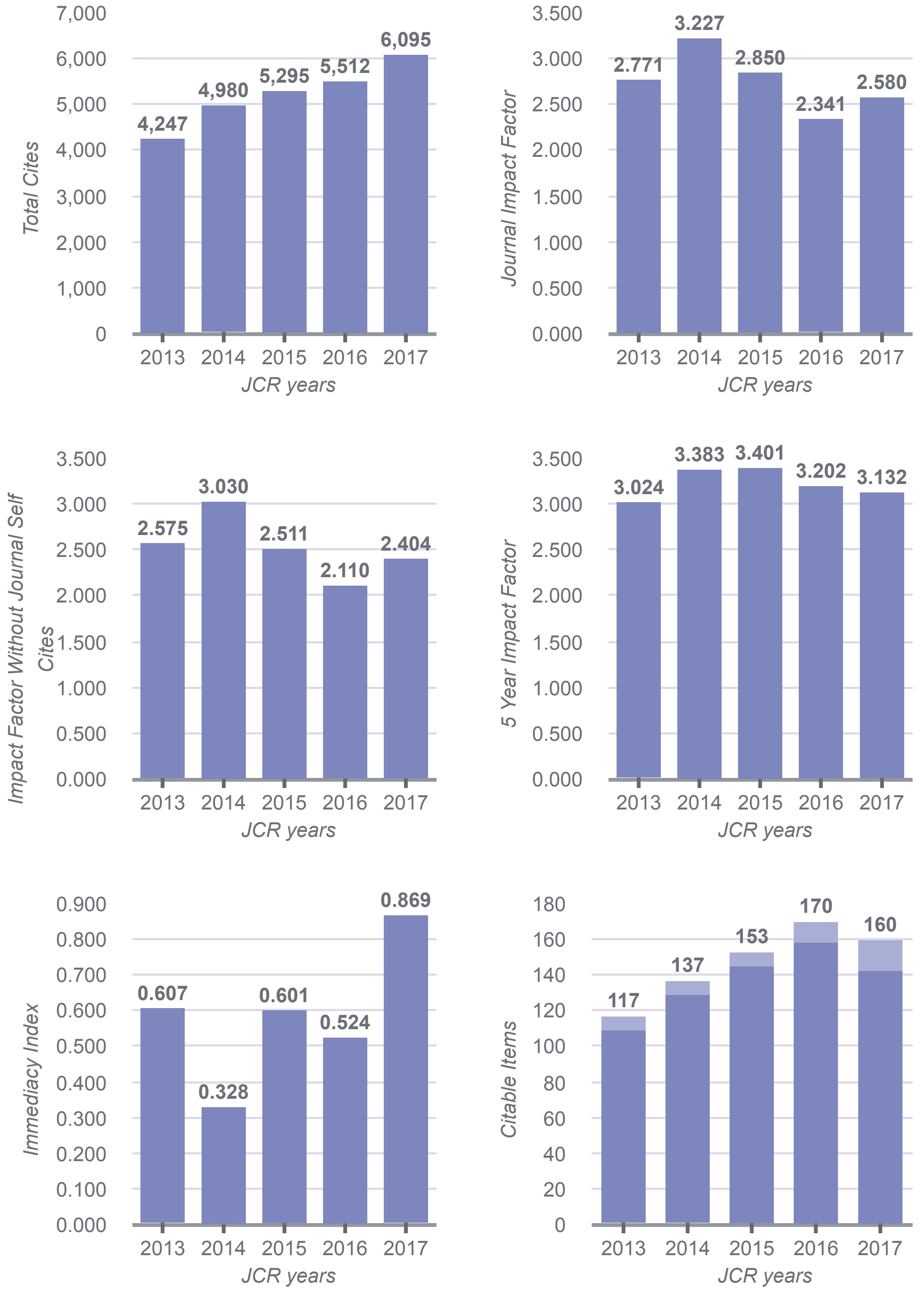

Downloaded on 2019/4/19 上午11:43:01 from Journal Citation Reports (©2019 Clarivate Analytics)

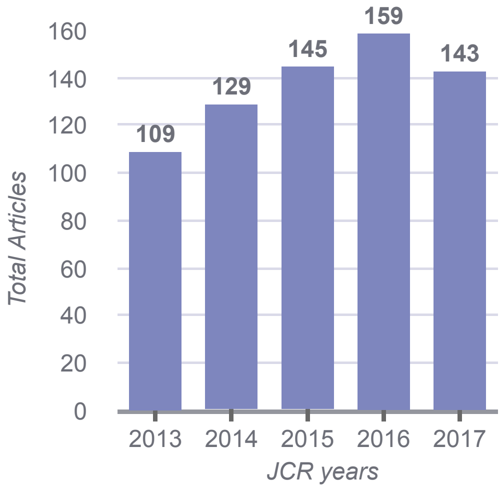

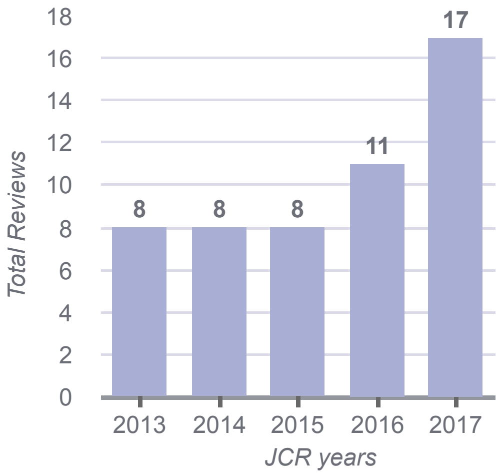

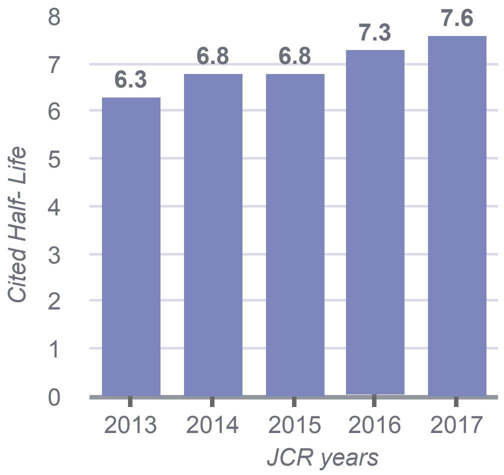

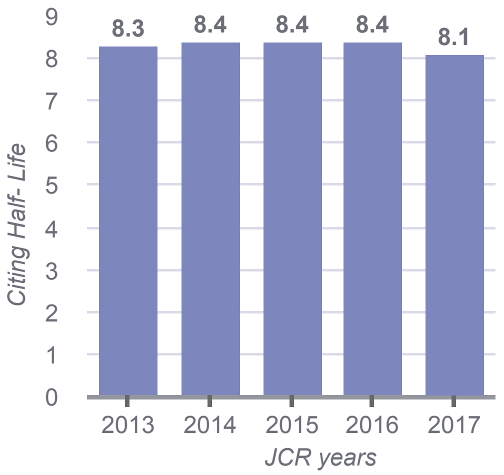

Downloaded on 2019/4/19 上午11:43:01 from Journal Citation Reports (©2019 Clarivate Analytics)

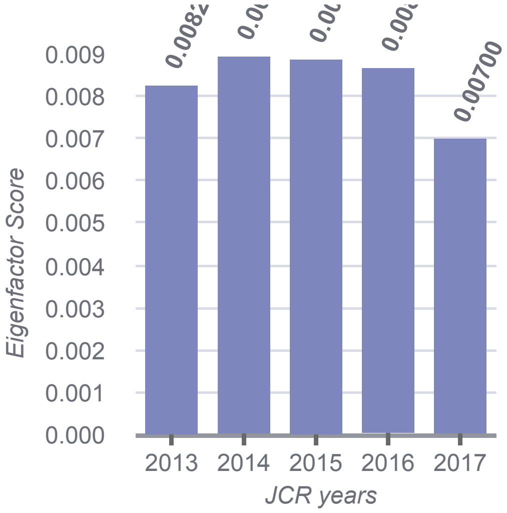

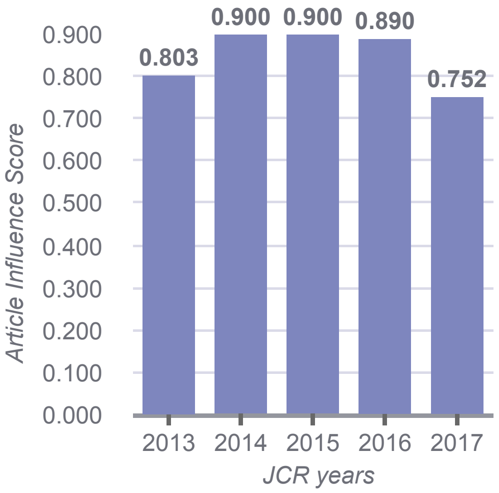

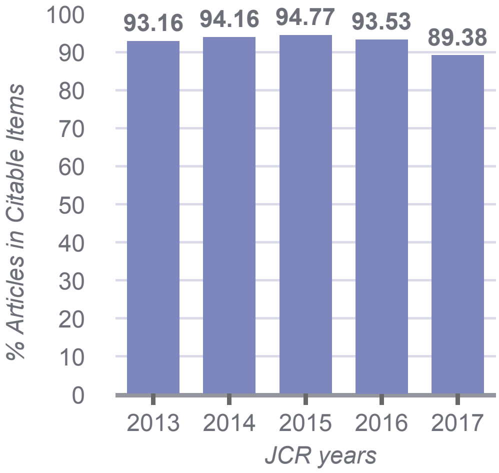

Downloaded on 2019/4/19 上午11:43:01 from Journal Citation Reports (©2019 Clarivate Analytics)

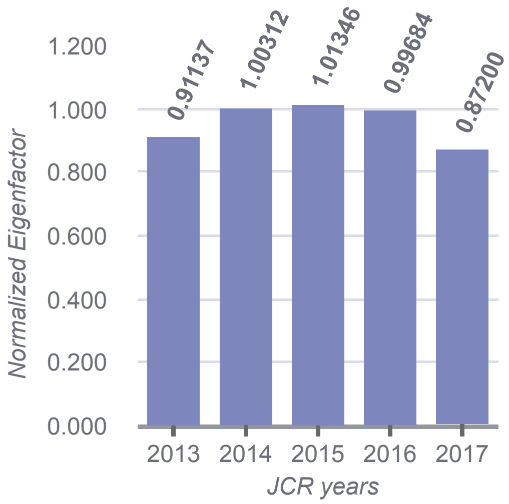

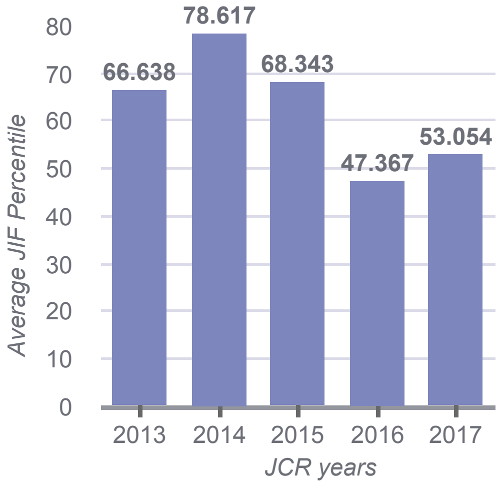

Downloaded on 2019/4/19 上午11:43:01 from Journal Citation Reports (©2019 Clarivate Analytics)

These data summarize the characteristics of the journal's published content for the most recent three years, that is, 2017 and the two prior years, combined. This information is based on all listed authors and addresses. It is meant to be descriptive rather than comparative.

Contributions by country/region

country	count
1. USA	207
2. CHINA MAINLAND	64
3. Brazil	38
4. GERMANY (FED REP GER)	35
5. Netherlands	30
6. Denmark	27
7. Canada	26
8. Japan	24
9. Italy	23
10. England	21

Contributions by organizations

organization	count
1. UNIVERSITY OF CALIFORNIA SYSTEM	18
2. UNITED STATES ENVIRONMENTAL PROTECTION AGENCY	17
3. UNIVERSIDADE ESTADUAL PAULISTA	13
4. UNIVERSITY OF ILLINOIS SYSTEM	12
- HUMBOLDT UNIVERSITY OF BERLIN	12
- FREE UNIVERSITY OF BERLIN	12
7. UNIVERSITY OF COPENHAGEN	11
- UTRECHT UNIVERSITY	11
9. HARVARD UNIVERSITY	10
- NETHERLANDS NATIONAL INSTITUTE FOR PUBLIC HEALTH & THE ENVIRONMENT	10

Downloaded on 2019/4/19 上午11:43:01 from Journal Citation Reports (©2019 Clarivate Analytics)
