# Supplementary figures and images for: Emerging trends and knowledge structure of epilepsy during pregnancy research for 2000–2018: a bibliometric analysis
Source: PeerJ. 2019 Jun 7;7:e7115. doi: 10.7717/peerj.7115 (PMC6557303; doi:10.7717/peerj.7115)

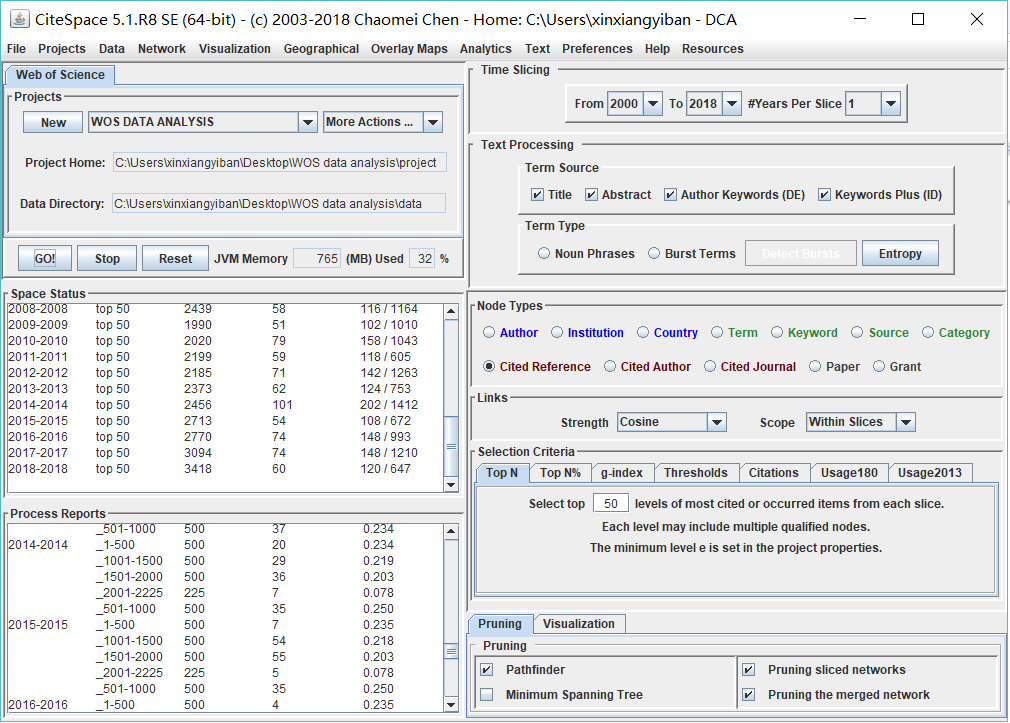

Supplement: Supplemental Information 9 [file peerj-07-7115-s009.png]

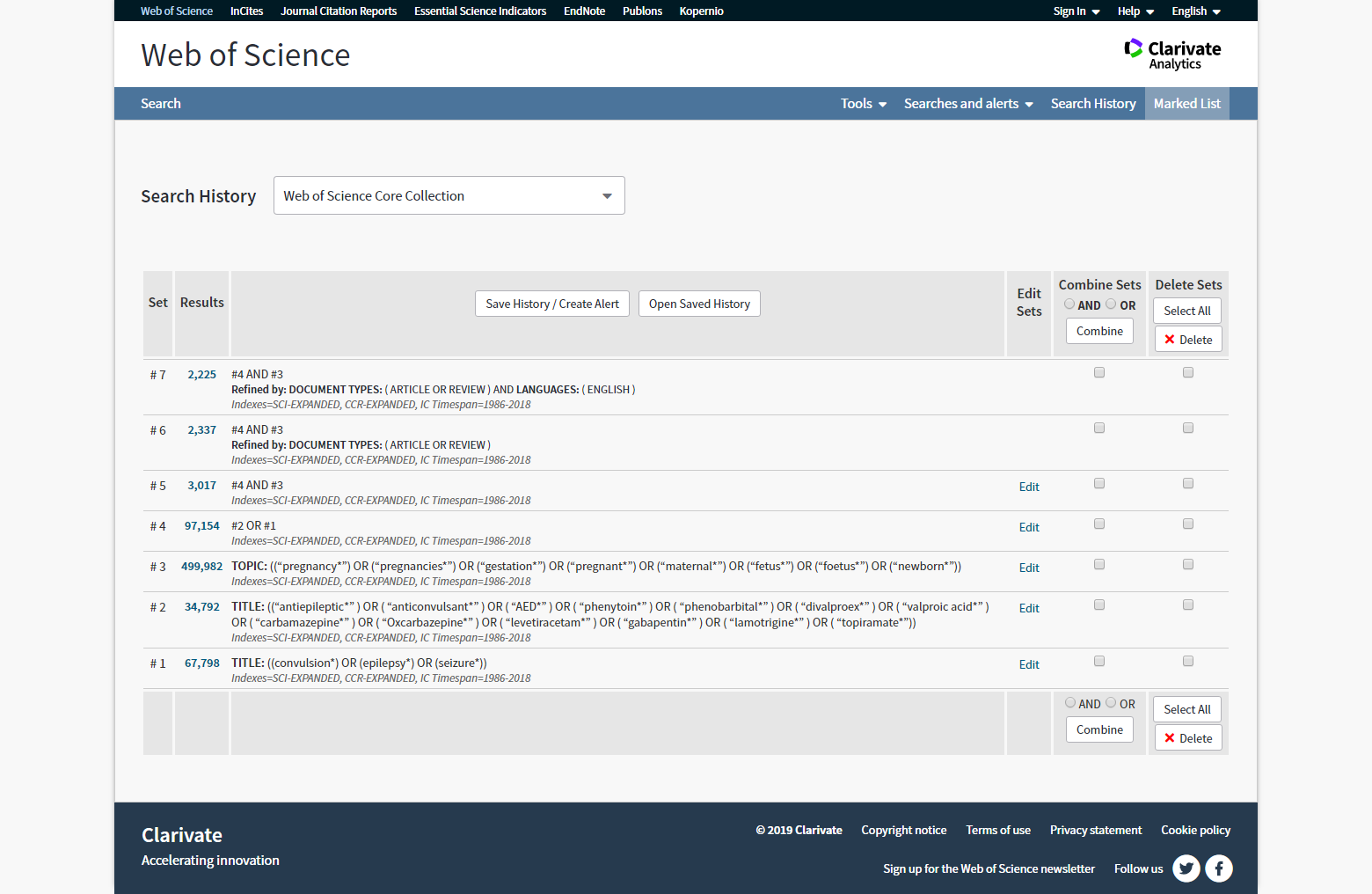

Supplement: Supplemental Information 10 [file peerj-07-7115-s010.png]

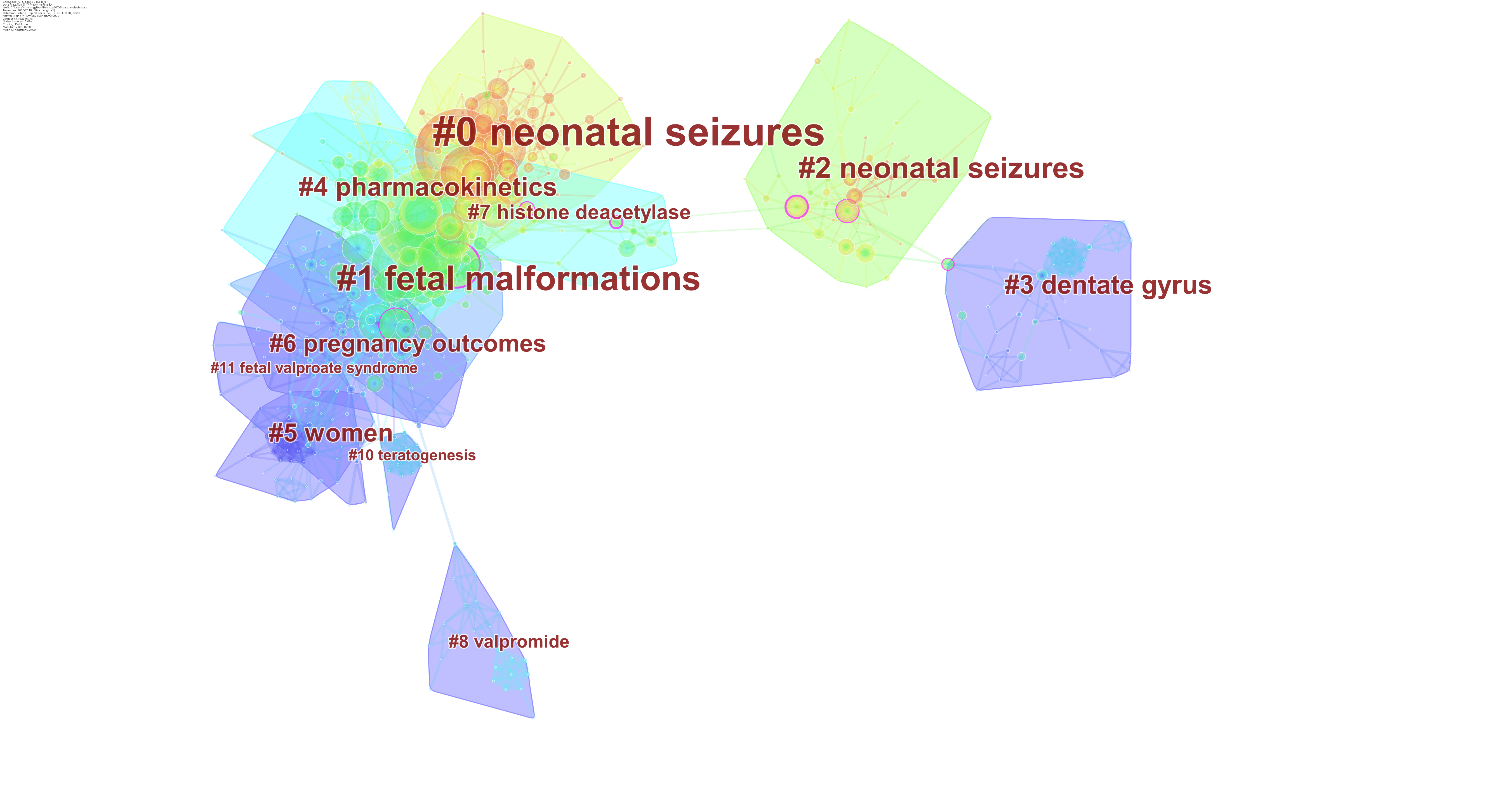

Supplement: Supplemental Information 11 [file peerj-07-7115-s011.png]

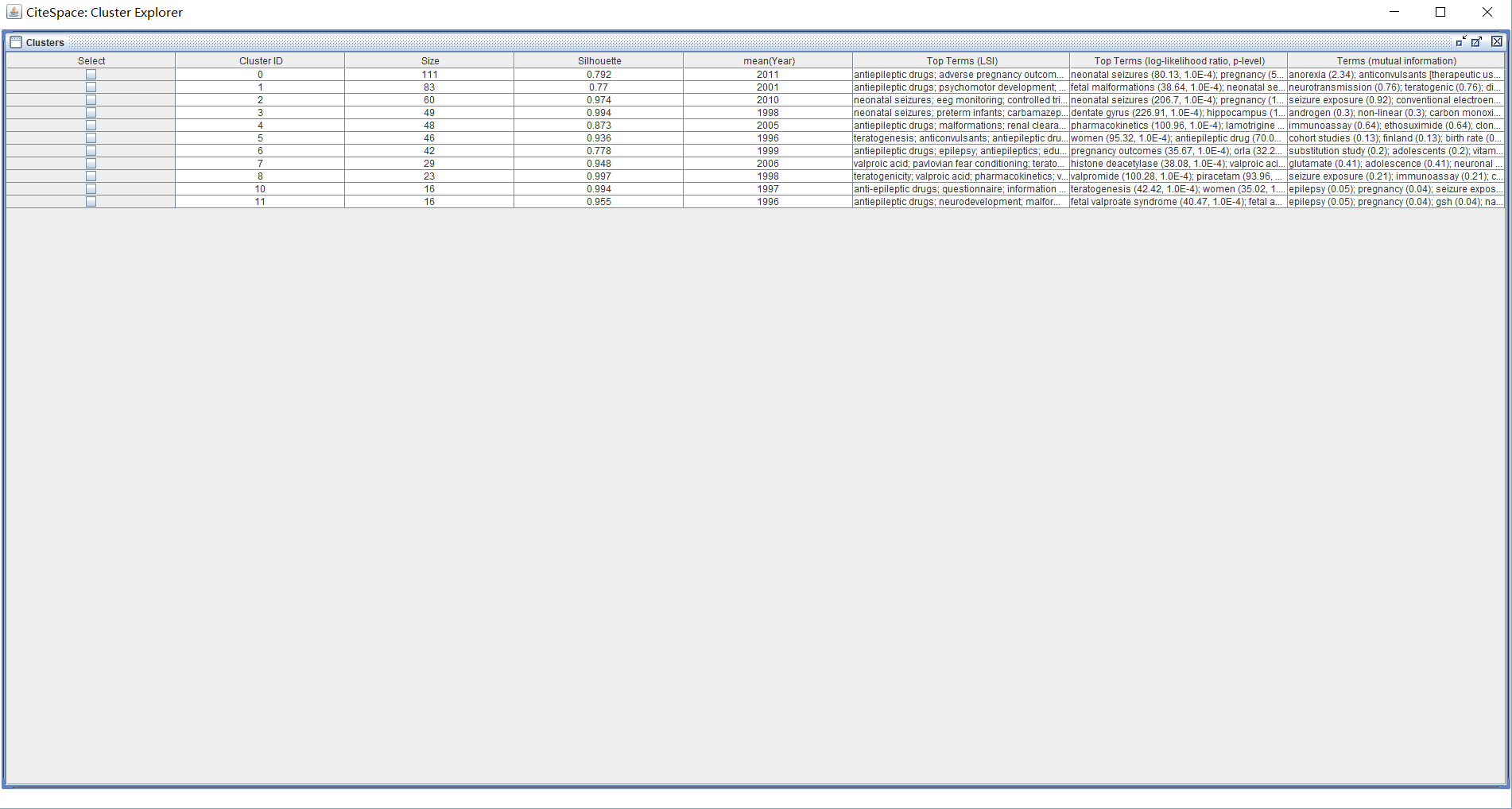

Supplement: Supplemental Information 12 [file peerj-07-7115-s012.png]

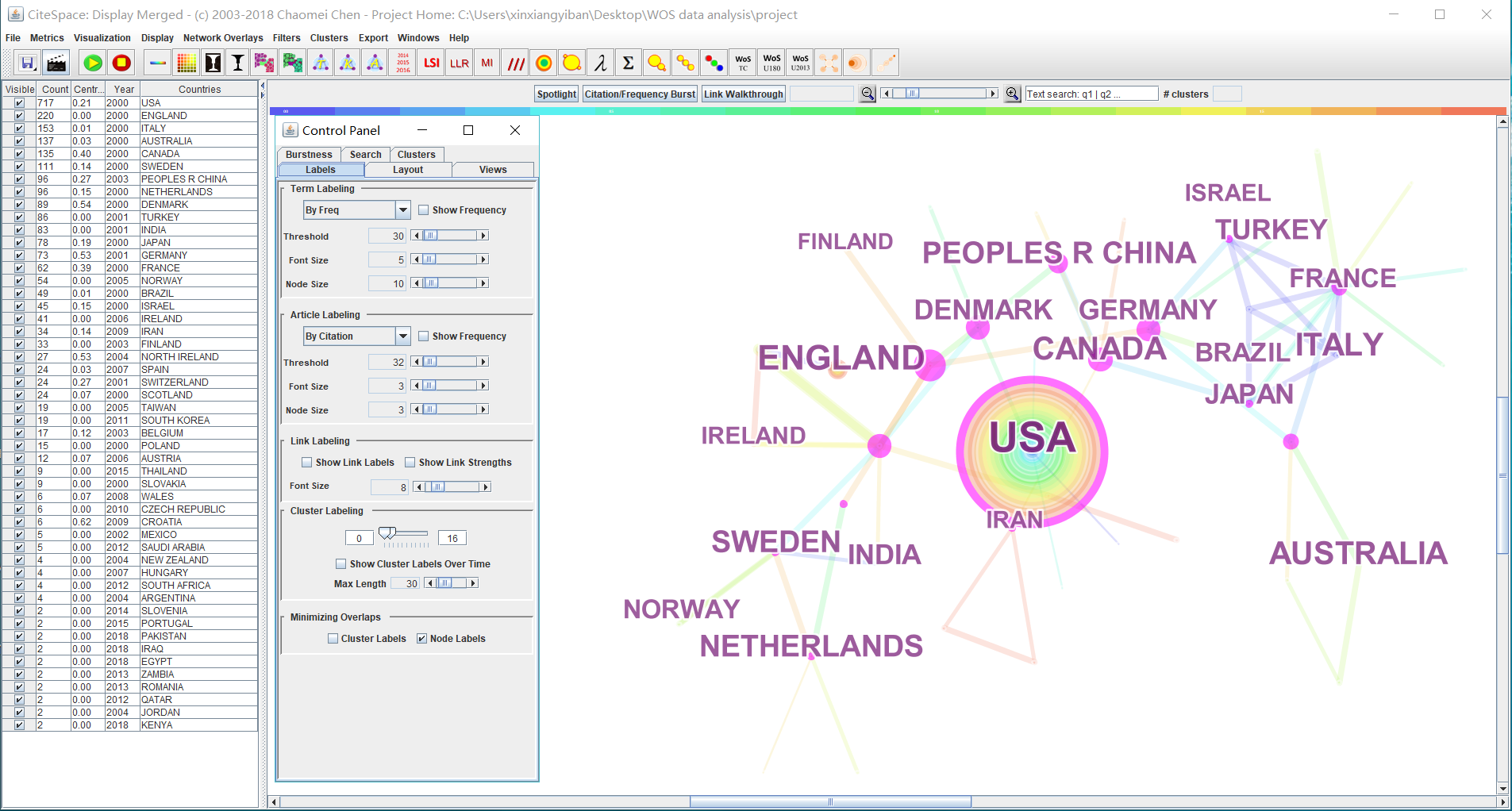

Supplement: Supplemental Information 13 [file peerj-07-7115-s013.png]

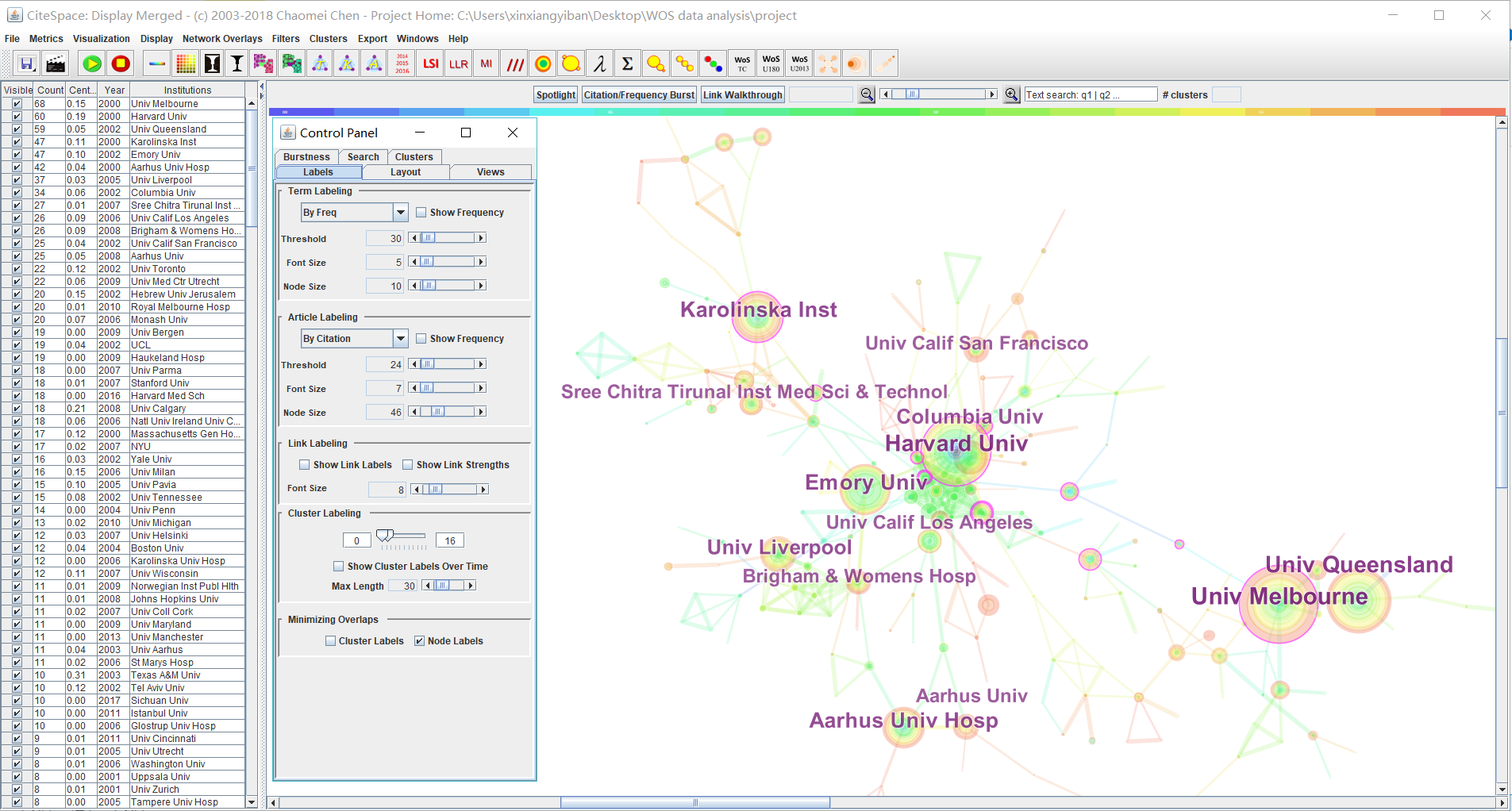

Supplement: Supplemental Information 14 [file peerj-07-7115-s014.png]

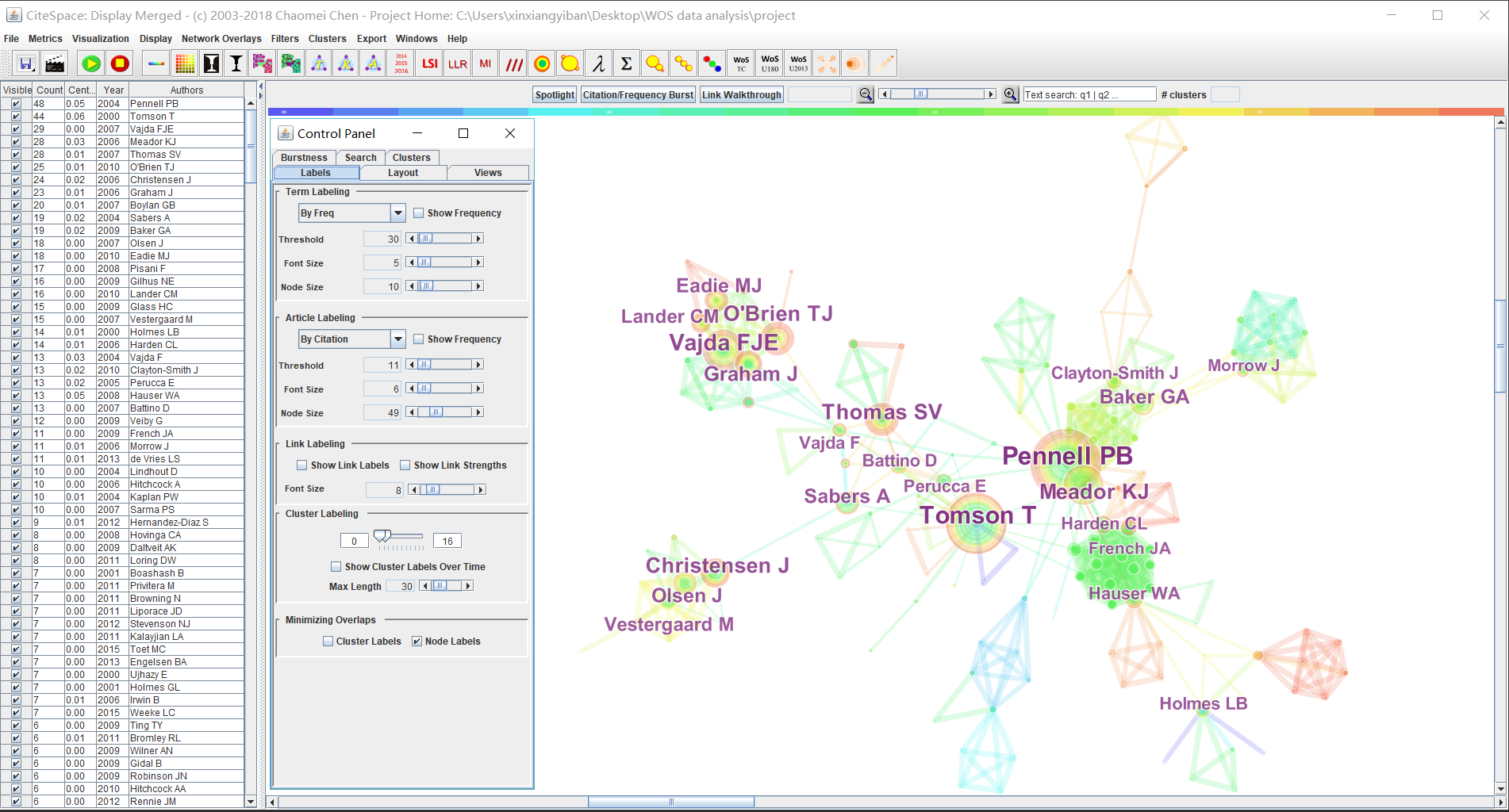

Supplement: Supplemental Information 15 [file peerj-07-7115-s015.png]

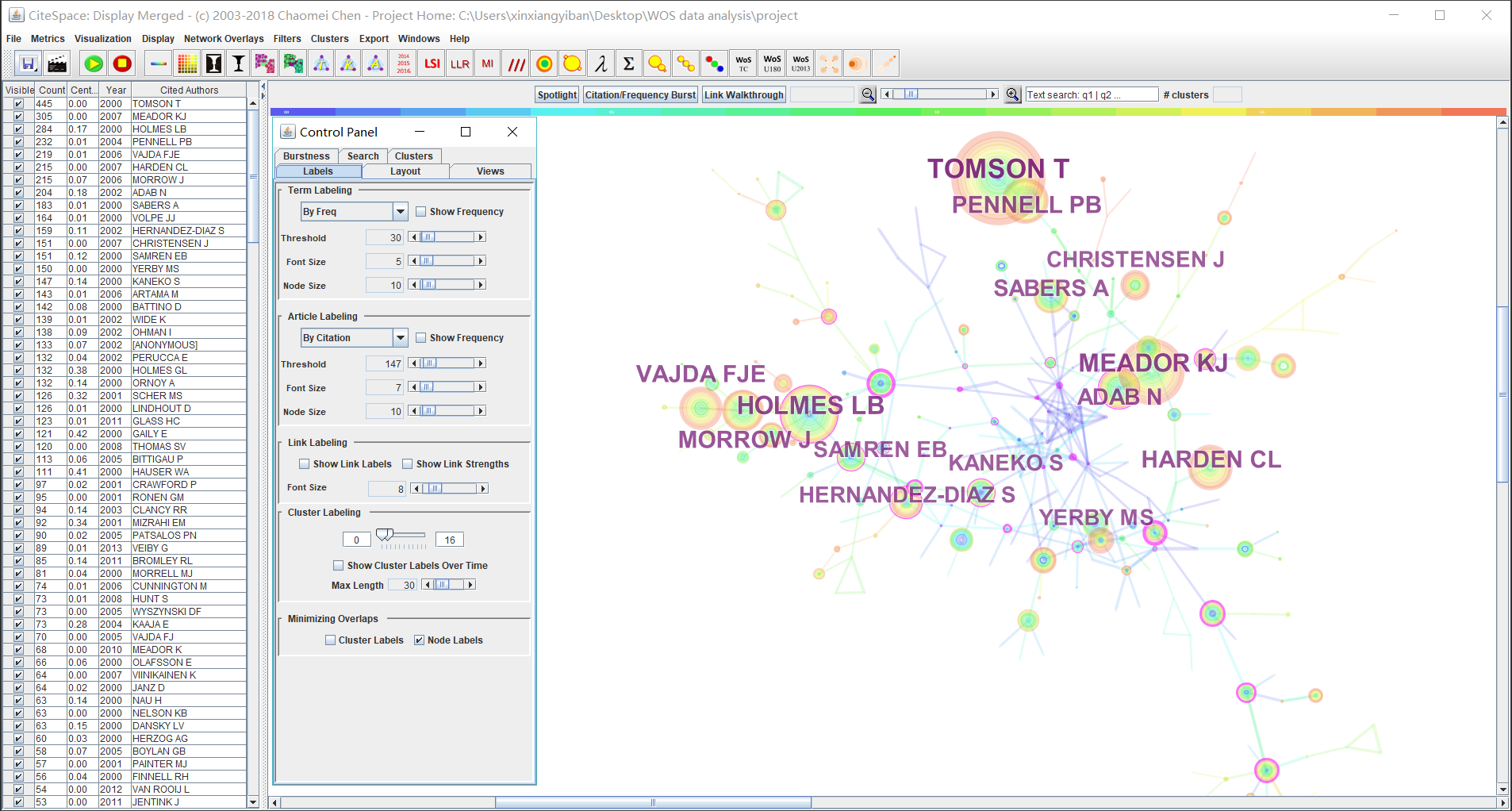

Supplement: Supplemental Information 16 [file peerj-07-7115-s016.png]

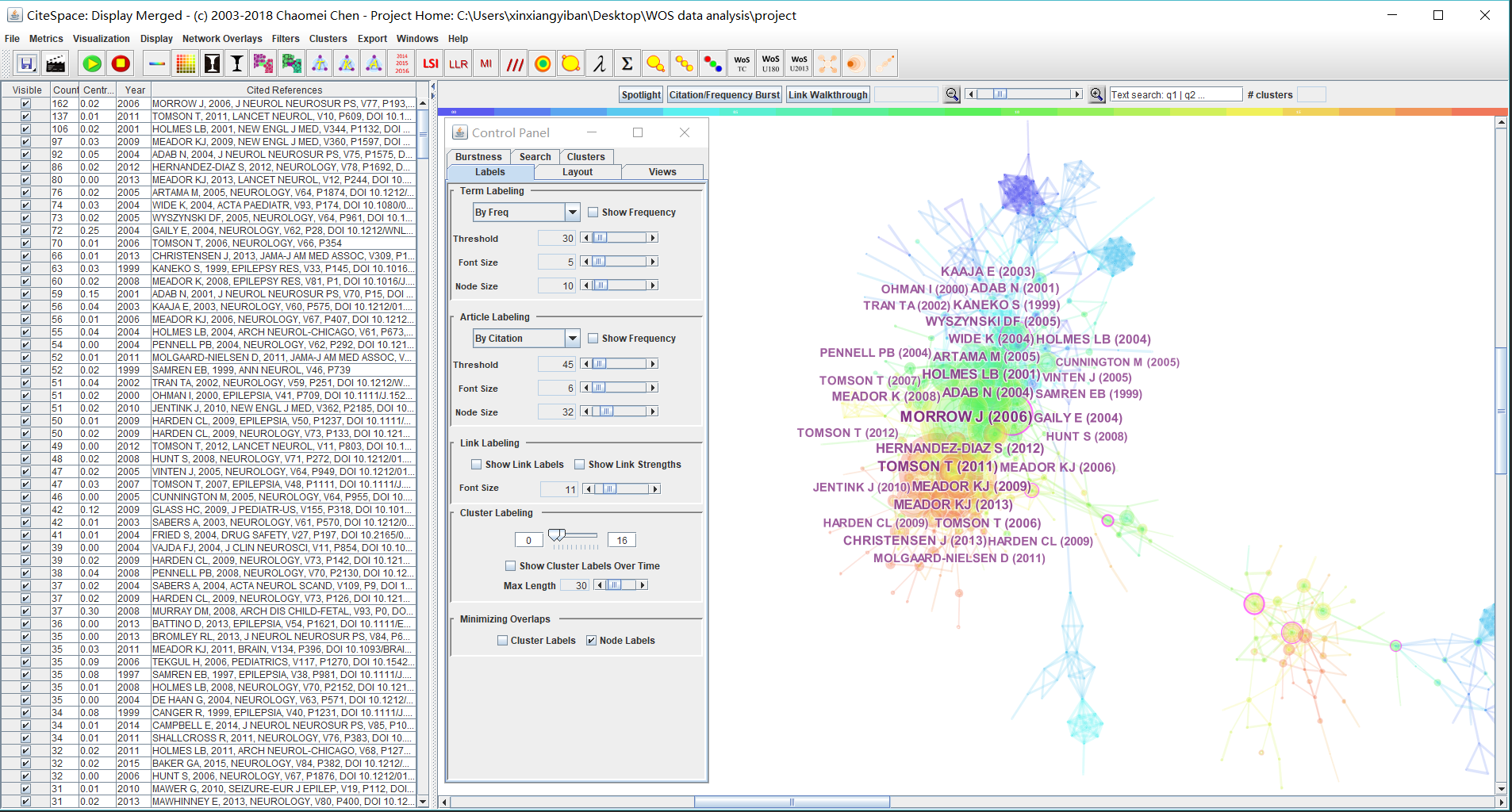

Supplement: Supplemental Information 17 [file peerj-07-7115-s017.png]

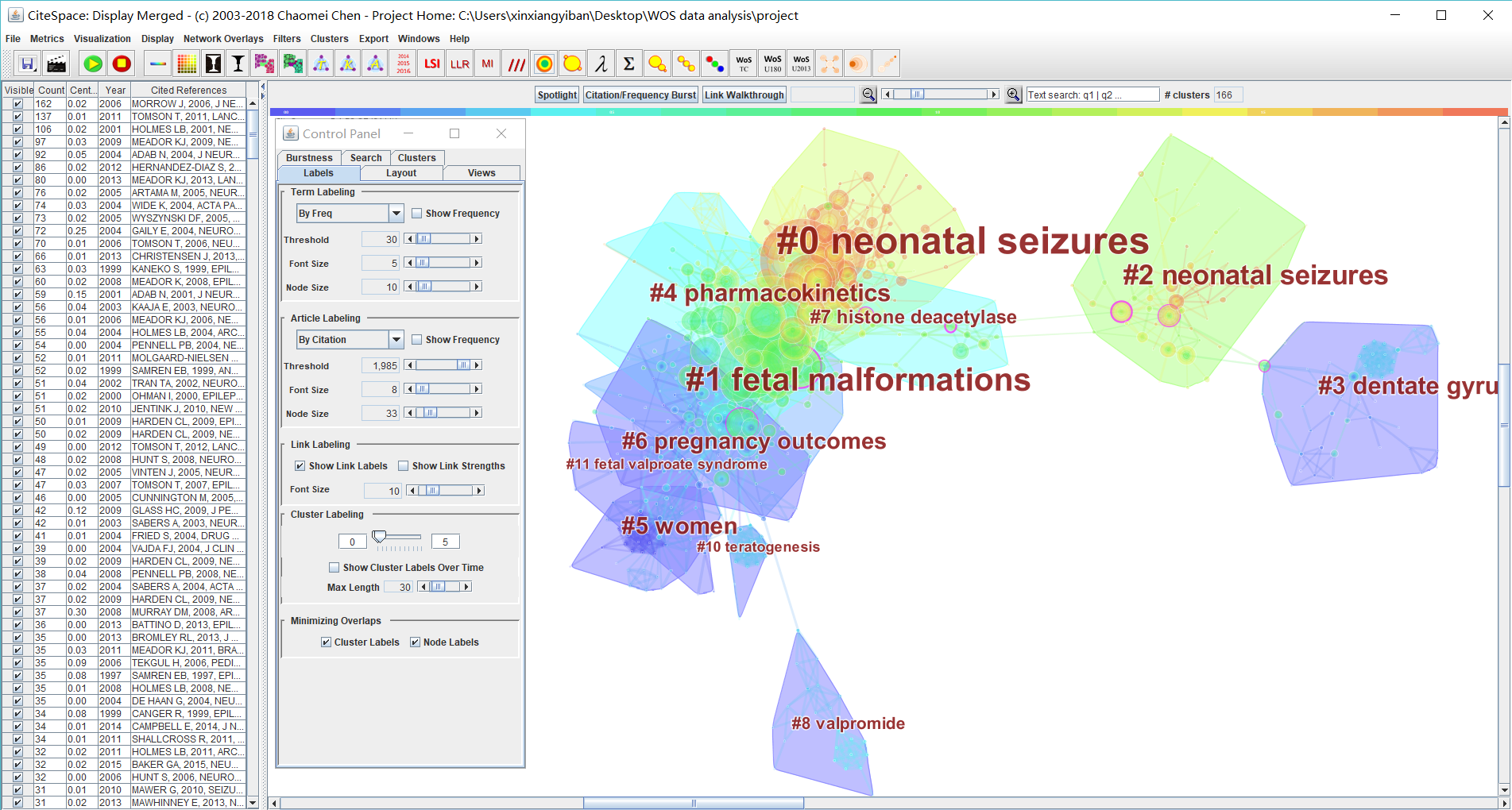

Supplement: Supplemental Information 18 [file peerj-07-7115-s018.png]

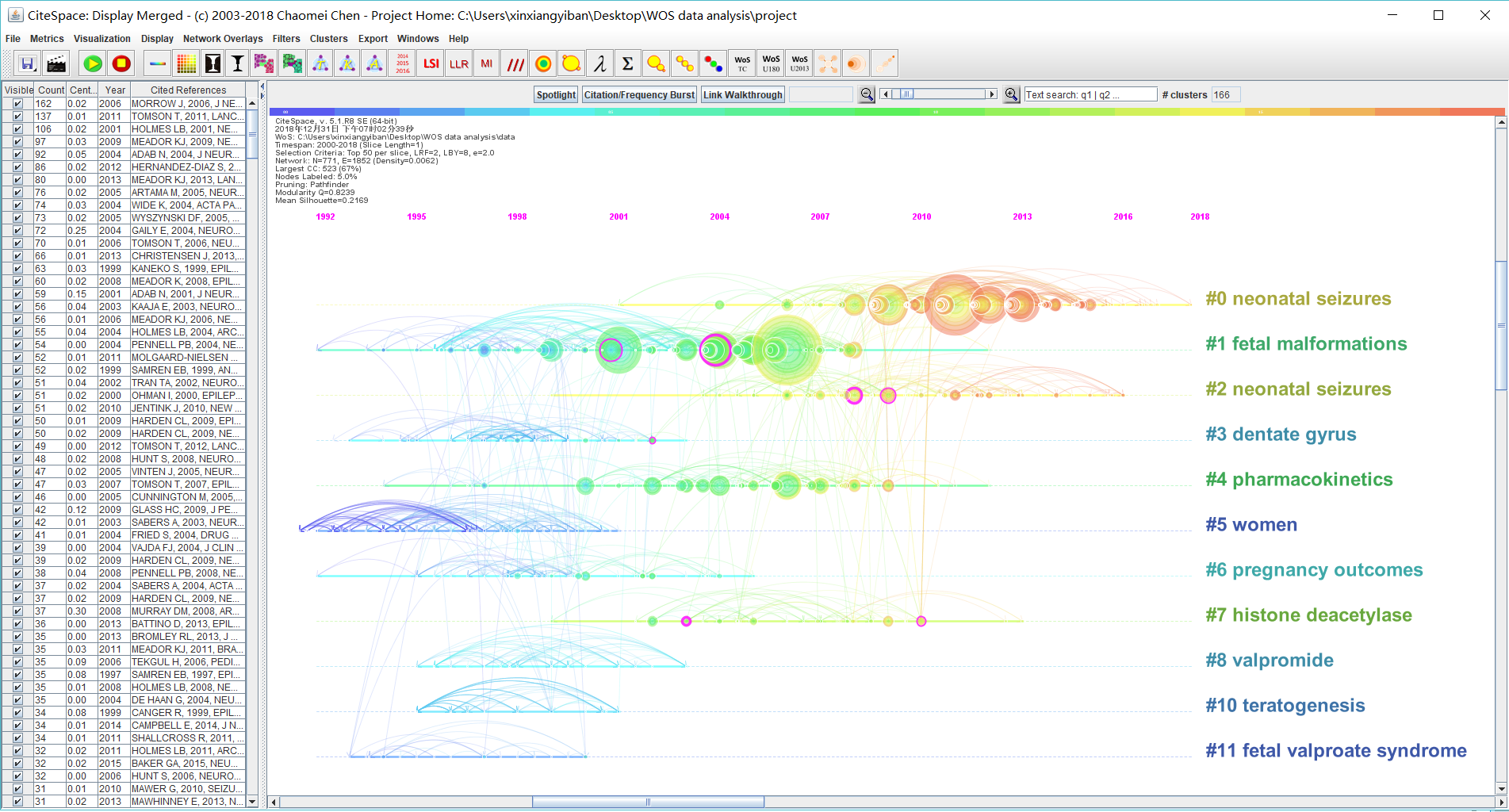

Supplement: Supplemental Information 19 [file peerj-07-7115-s019.png]
